# Whole-genome resequencing reveals signatures of selection and timing of duck domestication

**DOI:** 10.1093/gigascience/giy027

**Published:** 2018-04-09

**Authors:** Zebin Zhang, Yaxiong Jia, Pedro Almeida, Judith E Mank, Marcel van Tuinen, Qiong Wang, Zhihua Jiang, Yu Chen, Kai Zhan, Shuisheng Hou, Zhengkui Zhou, Huifang Li, Fangxi Yang, Yong He, Zhonghua Ning, Ning Yang, Lujiang Qu

**Affiliations:** 1 State Key Laboratory of Animal Nutrition, Department of Animal Genetics and Breeding, National Engineering Laboratory for Animal Breeding, College of Animal Science and Technology, China Agricultural University, Beijing, China; 2 Institute of Animal Science, Chinese Academy of Agricultural Sciences, Beijing, China; 3 Department of Genetics, Evolution and Environment, University College London, London, UK; 4 Department of Organismal Biology, Evolutionary Biology Centre, Uppsala University, Uppsala, Sweden; 5 Centre of Evolutionary and Ecological Studies, Marine Evolution and Conservation Group, University of Groningen, Groningen, The Netherlands; 6 Department of Animal Sciences, Center for Reproductive Biology, Veterinary and Biomedical Research Building, Washington State University, Pullman, United States; 7 Beijing Municipal General Station of Animal Science, Beijing, China; 8 Institute of Animal Husbandry and Veterinary Medicine, Anhui Academy of Agricultural Sciences, Hefei, China; 9 Poultry Institute, Chinese Academy of Agriculture Science, Yangzhou, China; 10 Institute of Pekin Duck, Beijing, China; 11 Cherry Valley farms (xianghe) Co., Ltd, Langfang, China

**Keywords:** duck, domestication, intensive selection, neuronal development, energy metabolism, plumage colouration

## Abstract

**Background:**

The genetic basis of animal domestication remains poorly understood, and systems with substantial phenotypic differences between wild and domestic populations are useful for elucidating the genetic basis of adaptation to new environments as well as the genetic basis of rapid phenotypic change. Here, we sequenced the whole genome of 78 individual ducks, from two wild and seven domesticated populations, with an average sequencing depth of 6.42X per individual.

**Results:**

Our population and demographic analyses indicate a complex history of domestication, with early selection for separate meat and egg lineages. Genomic comparison of wild to domesticated populations suggests that genes that affect brain and neuronal development have undergone strong positive selection during domestication. Our F_ST_ analysis also indicates that the duck white plumage is the result of selection at the melanogenesis-associated transcription factor locus.

**Conclusions:**

Our results advance the understanding of animal domestication and selection for complex phenotypic traits.

## Background

Animal domestication was one of the major contributory factors to the agricultural revolution during the Neolithic period, which resulted in a shift in human lifestyle from hunting to farming [1]. Compared with their wild progenitors, domesticated animals showed notable changes in behavior, morphology, physiology, and reproduction [2]. Detecting domestication-mediated selective signatures is important for understanding the genetic basis of both adaptation to new environments and rapid phenotype change [3, 4]. In recent years, to characterize signatures of domestication, whole-genome resequencing studies have been performed on a wide range of agricultural animals, including pig [5], sheep [6], rabbit [7], and chicken [8, 9].

Mallards (*Anas platyrhynchos*) are the world's most widely distributed and agriculturally important waterfowl species and are of particular economic importance in Asia [10]. Southeast Asia, particularly southern China, is the major center of duck domestication, with records indicating duck farming in the region dating at least 2,000 years [11, 12], particularly in wet environments [13] associated with rice crops [14]. In the absence of archaeological evidence, the exact timing of domestication and the time of meat and egg type ducks split remains unknown, with the first written records of domestic ducks in central China shortly after 500 BC [15].

It is clear that the domesticated duck originated from mallards [16], and domestic ducks can be classified as those produced primarily for meat (similar to chicken broilers) or eggs (similar to chicken layer lines). Together with the timing of duck domestication, the relative separation of duck meat and egg lines is also unknown. It is unclear whether ducks were domesticated once and subsequently selected for divergent meat and egg production traits or whether meat and egg populations were derived independently in two domestication events from wild mallards.

Moreover, domesticated mallards show many important behavioral [17] and morphological [18–20] differences from their wild ancestors, particularly related to plumage and neuroanatomy. However, the genetic basis of these phenotypic differences is still poorly understood.

## Data Description

In order to determine the timing of duck domestication in China, as well as identify the genomic regions under selection during domestication, we performed whole-genome resequencing from 78 individuals belonging to seven duck breeds (three for meat breeds, three for egg breeds, and one dual-purpose breed) and two geographically distinct wild populations. Using the large number of single nucleotide polymorphisms (SNPs) as well as small insertions and deletions (INDELs), we tested for population structure between domesticated and wild populations and we assessed the genome for signatures of selection associated with domestication. We tested alternative demographic scenarios with the pairwise sequential Markovian coalescent method combined with the diffusion approximation method.

## Analyses

### Genetic variation

**Figure 1:**
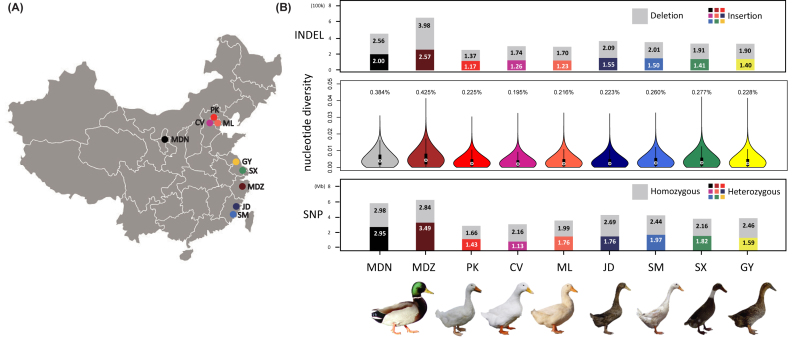
Experimental design and variants statistics. A)null Sampling sites in this study. A total of 78 ducks from two wild populations (mallard Ningxia [MDN] n = 8; mallard Zhejiang [MDZ] n = 14), three meat breeds (Pekin [PK] n = 8; Cherry Valley [CV] n = 8; maple leaf [ML] n = 8), three egg breeds (Jin Ding [JD] n = 8; Shan Ma [SM] n = 8; Shao Xing [SX] n = 8), and one dual-purpose breed (Gao You [GY] n = 8) were selected. B) Genomic variation of nine populations. Mean number of SNPs and heterozygous and homozygous SNP ratio in the nine populations are shown at the bottom. Nucleotide diversity ratios of the nine populations are shown at the middle. The nucleotide diversity ratios in wild mallards are dramatically higher than ratios in domesticated ducks. Number of insertions and deletions in the nine populations are shown at the top. The number of deletions was higher than the number of insertions in all nine populations.

We individually sequenced 22 wild and 56 domestic ducks from two wild populations and seven domestic breeds (three meat breeds, three egg breeds, and one dual-purpose breed) from across China (Fig.1A) to an average of 6.42X coverage per individual (613.37 Gb of high-quality paired-end sequence data) after filtering and quality control, resulting in 535 billion mappable reads across 78 ducks (Supplemental Table S1).

Across samples, we identified 39.2 million variants, consisting of 36.1 million SNPs (average per sample = 4.5 m SNPs; range = 2.34 – 9.52 M SNPs) and 3.1 million INDELs (average per sample = 0.4 million INDELs; range = 0.21 – 0.89 million INDELs) (Fig.1B, Supplemental Figs. S1 and S2, Supplemental Table S2). Single base-pair INDELs were the most common, accounting for 38.63% of all detected INDELs (Supplemental Table S3). Our dataset covers 96.2% of the duck dbSNP database deposited in the Genome Variation Map (GVM) [21]. In general, domesticated populations showed lower number of SNPs (*t* test, *P* = 3.13 × 10^−12^) and nucleotide diversity (*t* test, *P* = 2.20 × 10^−16^) compared to wild mallards (Fig.1B). Moreover, homozygosity in domesticated ducks was significantly higher than ratios in wild mallards (*t* test, *P* = 1.35 × 10^−10^) consistent with the larger panmictic wild population or with the higher artificial selection and inbreeding within domesticated stocks.

### Population structure and domestication

**Figure 2:**
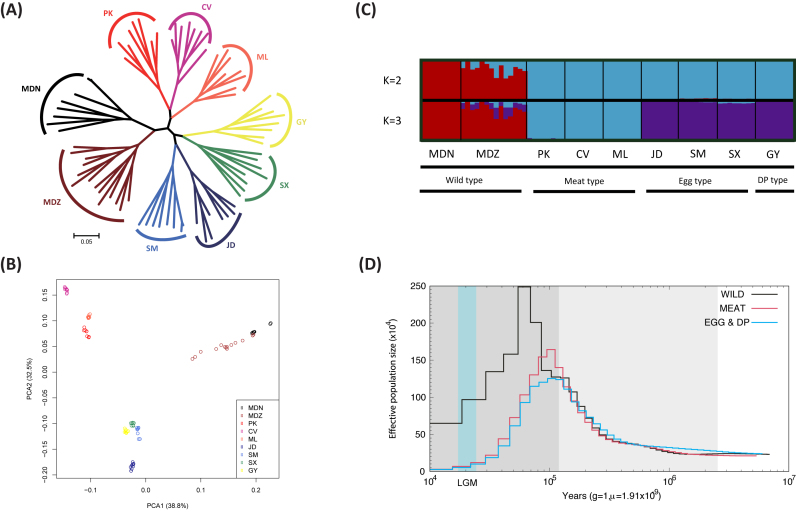
Population genetic structure and demographic history of nine duck populations. A) Neighbor-joining phylogenetic tree of nine duck populations. The scale bar is proportional to genetic differentiation (p distance). B) PCA plot of duck populations. Eigenvector 1 and 2 explained 38.8% and 32.5% of the observed variance, respectively. C) Population genetic structure of 78 ducks. The length of each colored segment represents the proportion of the individual genome inferred from ancestral populations (K = 2–3). The population names and production type are at the bottom. DP type means dual-purpose type. D) Demographic history of duck populations. Examples of PSMC estimate changes in the effective population size over time, representing variation in inferred Ne dynamics. The lines represent inferred population sizes and the gray shaded areas indicate the Pleistocene period, with Last Glacial Period (LGP) shown in darker gray, and Last Glacial Maximum (LGM) shown in light blue areas.

Phylogenetic relationships, based on a neighbor-joining of pairwise genetic distances of whole-genome SNPs (Fig.2A) and principal component analysis (PCA; Fig.2B), revealed strong clustering into three distinct genetic groups. In general, we observed separate clusters corresponding to wild ducks (MDN and MDZ), ducks domesticated for meat production (PK, CV, and ML), and ducks domesticated for egg production (JD, SM, and SX). The dual-purpose domesticate (GY) clustered with ducks domesticated for egg production (Fig.2B and C).

We further performed population structure analysis using FRAPPE [22], which estimates individual ancestry and admixture proportions assuming K ancestral populations (Fig. 2C). With K = 2, a clear division was found between wild-type ducks (MDN and MDZ) and domesticated ducks (PK, CV, ML, JD, SM, SX, and GY). With K = 3, a clear division was found between meat type ducks (PK, CV, and ML) and egg type ducks mixed with dual-purpose type ducks (JD, SM, SX, and GY).

Next, we explored the demographic history of our samples to differentiate whether domestication of meat- and egg-producing ducks was the result of one or multiple events. First, we estimated changes in effective population size (N_e_) in our three genetic clusters in a pairwise sequentially Markovian coalescent (PSMC) framework [23]. The meat type ducks (PK, CV, and ML) showed concordant demographic trajectories with egg and mixture dual-purpose type populations (JD, SM, SX, and GY), with one apparent expansion around the Penultimate Glaciation Period (0.30-0.13 million years ago) [4, 24] and Last Glacial Period (110–12 thousand years ago) [25, 26], followed by a subsequent contraction (Fig. 2D). Next, we tested multiple demographic scenarios related to domestication using a diffusion approximation method for the allele frequency spectrum (∂a∂i) (Supplemental Figs. S3 and S4). Among the four isolation models tested (models 1 – 4), the model of a single domestication with subsequent divergence of the domesticated breeds (model 2) was both consistent with our population structure results (Fig.2) and had the lowest Akaike information criteria (AIC) value, indicating a better overall fit to the data (log-likelihood = –33 388.43; AIC = 66 788) (Supplemental Fig. S3).

Demographic parameters estimated from the single domestication model (model 2) indicated that domestication occurred 2,228 years ago, with 95% confidence interval (CI) ± 441 years ago, followed by a rapid subsequent divergence of the meat breed from the egg/dual-purpose breeds roughly 100 years after the initial domestication event (Table1). Our results suggest that following an initial bottleneck associated with domestication, with an estimated N_e_ of 320 (95% CI ± 3) individuals for the ancestral domesticated population, the population has expanded to the current N_e_ of 5,597 (95% CI ± 1,195) and 12,988 (95% CI ± 2,877) in the meat type and egg/dual purpose breeds, respectively. N_e_ estimates for domesticated breeds are lower than the Ne of 88,842 (95% CI ±18,065) in wild mallards, consistent with the large panmictic wild population.

**Table 1:** Maximum likelihood population demographic parameters

Parameter	ML estimate	95% CI
N_e_ of ancestral population after size change	663,439	644,726–682,152
N_e_ of the wild population	88,842	70-778–106-907
N_e_ of the ancestral domesticated population	320	316–323
N_e_ of the meat breed	5,597	4,402–6,792
N_e_ of the egg/dual-purpose	12,988	10,111–15,865
Time of size change in the ancestral population	249,944	227,912–267,518
Time of domestication	2,228	1,787–2,669
Time of breed divergence	2,126	1,686–2,567
Migration _wild ← meat_	1.12	1.00–1.24
Migration _wild ← egg/dp_	3.92	3.11–4.73

Best fit parameter estimates for the model of a single domestication event followed by divergence of the domesticated breeds, including changes in population size. The 95% confidence intervals were obtained from 100 bootstrap datasets. Time estimates are given in years and migration are in units of number of migrants per generation.

Gene flow estimates were relatively high, with 1 and 4 migrants per generation from the meat and egg/dual-purpose breeds, respectively, into the wild population. Our results suggest duck domestication was a recent single domestication event followed by rapid subsequent selection for separate meat and egg/dual-purpose breeds.

### Selection for plumage color

**Figure 3:**
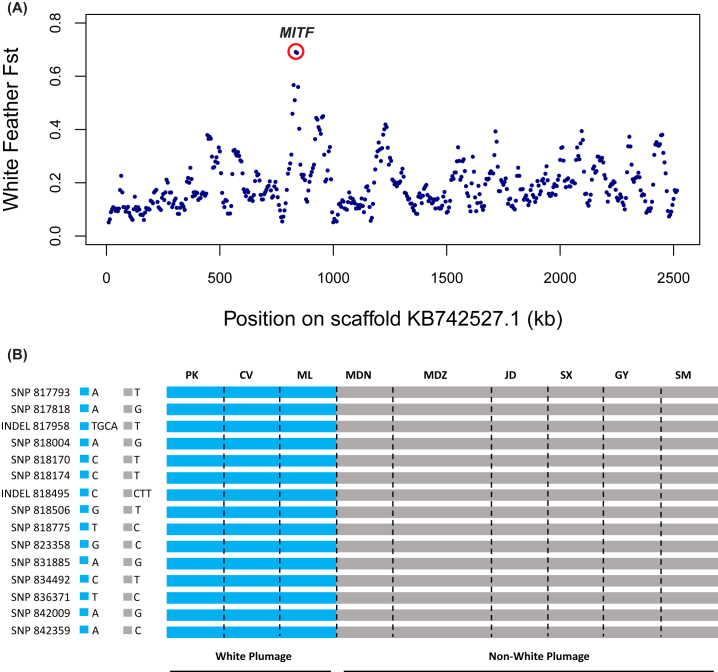
MITF shows different genetic signatures between white-plumage and non-white-plumage ducks. A) FST plot around the MITF locus. The F_ST_ value of MITF is highest for scaffold KB742527.1, circled in red. Each plot represent a 10-kb window. B) The 13 homozygous SNPs and 2 homozygous INDELs were identified in white-plumage ducks and absent in non-white-plumage ducks. SNPs and INDELs were named according to their position on the scaffold.

Derived traits in domesticated animals tend to evolve in a predictable order, with color variation appearing in the earliest stages of domestication, followed by coat or plumage and structural (skeletal and soft tissue) variation, and finally behavioral differences [27, 28]. One of the simplest and most visible derived traits of ducks is white plumage color. In order to detect the signature of selection associated with white feathers, we searched the duck genome for regions with high F_ST_ between the populations of white-feather (PK, CV, and ML) and non-white-feather (MDN, MDZ, JD, SX, and GY) birds based on sliding 10-kbwindows. We identified a region of high differentiation between white-plumage and non-white-plumage ducks overlapping the melanogenesis associated transcription factor (*MITF*; F_ST_ = 0.69) (Fig. 3A). In the intronic region of *MITF*, we identified 13 homozygous SNPs and 2 homozygous INDELs present in all white-plumage breeds (n = 24) and absent in all non-white-plumage breeds (n = 54) (Fig.3B). These mutations were completely associated with the white-plumage phenotype, suggesting a causative mutation at the *MITF* locus. Moreover, to validate the reliability of variants detected in MITF gene, we amplified the first three SNPs (SNP817793, SNP817818, and SNP818004) and all INDELs by diagnostic polymerase chain reaction (PCR) combined with Sanger sequencing in the 78 white- and non-white-plumage ducks. The results show that the three SNPs and INDEL817958 completely match our NGS analysis (Supplemental Fig. S5). For INDEL818495, we were unable to design a suitable PCR primer to amplify this region.

### Selection for other domestication traits

**Figure 4:**
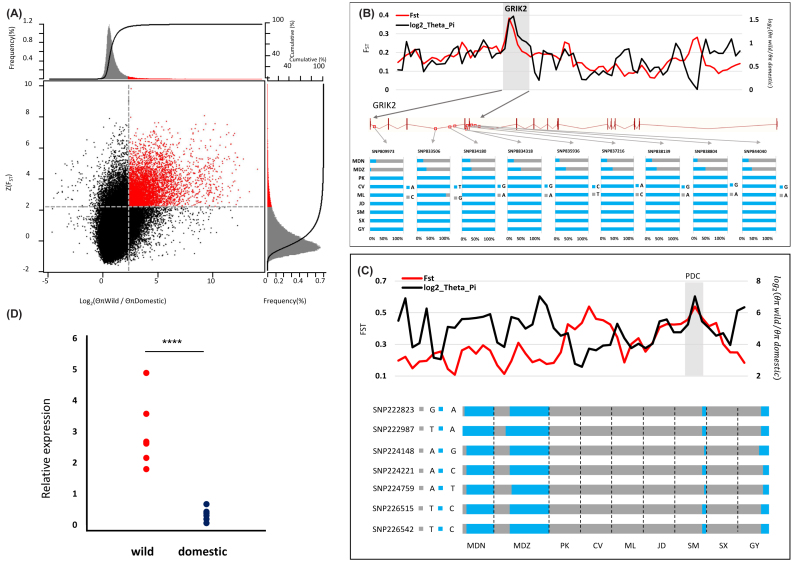
Genomic regions with strong selective sweep signals in wild population ducks and domesticated population ducks. A) Distribution of θπ ratios θπ (wild/domesticated) and Z(F_ST_) values, which are calculated by 10-kbwindows with 5-kbsteps. Only scaffolds >10 kbwere used for our calculation, as F_ST_ results calculated on a small scaffold are unlikely to be accurate. Red data points located to the top-right regions correspond to the 5% right tails of empirical *log*_2_ (*θπ wild*/*θπ domestic*) ratio distribution, and the top 5% empirical Z(F_ST_) distribution are genomic regions under selection during duck domestication. The two horizontal and vertical gray lines represent the top 5% value of Z(F_ST_) (2.216) and *log*_2_ (*θπ wild*/*θπ domestic*) (2.375), respectively. B) The *log*_2_ (*θπ*) ratios and F_ST_ values around the *GRIK2* locus and allele frequencies of nine SNPs within the *GRIK2* gene across nine duck populations. The black and red lines represent *log*_2_ (*θπ wild*/*θπ domestic*) ratios and F_ST_ values, respectively. The gray bar shows the region under strong selection in *GRIK2* gene. The nine red rectangular frames correspond to the locus on gene of nine SNPs. The SNPs were named according to their position on the scaffold. C) The PDC gene showed a different genetic signature in domesticated and wild ducks. *The log*_2_ (*θπ*) ratios and F_ST_ values around the *PDC* locus. The *PDC* gene region is shown in gray. Allele frequencies of seven SNPs within the *PDC* gene across nine duck populations. The SNPs are named according to their scaffold position. D) The PDC gene expression level differs between domesticated and wild ducks. PDC mRNA expression levels in brain of wild (MDN, n = 3; MDZ, n = 4) and domesticated (PK, n = 1; CV, n = 1; ML, n = 1; JD, n = 1; SM, n = 1; SX, n = 1; GY, n = 1) ducks. ****P value from *t* test (*P* < 0.0001).

In order to detect the signature of selection for other traits associated with duck domestication, we scanned the duck genome for regions with a high coefficient of nucleotide differentiation (F_ST_) among the populations of wild (MDN and MDZ) and domesticated (PK, CV, ML, JD, SM, SX, and GY) ducks based on 10-kbsliding windows, as well as global F_ST_ between each population (Supplemental Table S4). Owing to the complex and partly unresolved demographic history of these populations, it is difficult to define a strict threshold that distinguishes true sweeps from regions of homozygosity caused by drift. We therefore also calculated the pairwise diversity ratio (*θ*_π_(wild/domesticated)). We identified 292 genes in the top 5% of both F_ST_ and *θ*_π_ scores, putatively under positive selection during domestication (Fig. 4A, Supplemental Table S5).

All 292 genes located in the top 5% F_ST_ regions were used for the gene ontology GO (The framework for the model of biology, which provides the most comprehensive rescoure currently available for computable knowledge regarding the functions of genes and gene products) analysis, resulting in a total of 57 GO enrichment terms (Supplementary Table S6). Because domesticated ducks are known to differ from wild ducks in body size, body fat percentage, behavior, egg productivity, growth speed, and flight capability, we focused our analysis on GO annotations of neural-related processes, lipid metabolism and energy metabolism, reproduction, and skeletal muscle contraction for our 292 putative positive selection genes. In this reduced data set, the neuro-synapse-axon and lipid-energy metabolism pathways were overrepresented (Supplemental Table S7) in our list of genes under selection.

From the highlighted GO terms, 25 neuro-synapse-axon genes were identified as being under positive selection, with six (*ADGRB3*, *EFNA5*, *GRIN3A*, *GRIK2*, *SYNGAP1*, and *HOMER1*) in the top 1% of F_ST_ and *θ*_π_ (Supplemental Table S8). In particular, *GRIK2* (glutamate receptor, ionotropic kainate 2) and *GRIN3A* (glutamate receptor, subunit 3A) both showed high F_ST_ and *θ*_π_ value compared to neighboring regions, suggesting functional importance (Fig. 3B, Supplemental Tables S5, S8).

Beyond the neuronal-synapse-axon genes, 115 genes were identified in the four lipid- and energy-related pathways with high F_ST_ and *θ*_π_ values, particularly related to fatty acid metabolism. Among these genes, 37 were found with both parameters yielding top 1% ranked values (Supplemental Table S8) such as phosphatidylinositol 3-kinase catalytic subunit type 3 (*PIK3C3*) and patatin-like phospholipase domain containing 8 (*PNPLA8*).

To infer whether selection extends beyond allelic variation and also affects gene expression, we compared individual gene expression in the brain, liver, and breast muscle between seven wild mallards and seven domesticated ducks in natural states with RNA-sequencing (RNA-seq) (Supplemental Table S9). We detected three genes (*PDC*, *MLPH*, and *NID2*) in the brain, two genes (*MAPK12* and *BST1*) in the liver, and no genes in breast muscle with significantly different expression between wild and domesticated ducks. Of the five differentially expressed genes, *PDC* was the only gene that also showed evidence of a selective sweep at the genomic level (Supplemental Table S5, Fig.3C and D). The results suggest that the *PDC* gene is of substantial functional importance in phenotypic differentiation among wild and domestic ducks.

## Discussion

Domesticated animals have contributed greatly to human society and human population growth by providing a stable source of animal protein, fat, and accessory products such as leather and feathers (including down). To illuminate the genetic trajectories of duck domestication, we performed whole-genome sequencing of 78 ducks including seven domesticate breeds and two wild populations. This is the first study to characterize the genetic architecture, phylogenetic relationships, and domestication history of domesticated ducks and wild mallards.

Using this powerful dataset and a suite of cutting-edge population genomic and functional genetic analyses, we observed higher mean variant numbers and nucleotide diversity for the wild mallard populations compared to the domestics, consistent with both a greater panmictic mallard population as well as recent sweeps associated with domestication.

### Population structure and domestication

We observed a large expansion of the duck population at the interglacial period, which could be the result of beneficial climatic changes including rising temperatures and sea levels. In contrast, the glacial maximum coincided with a reduction in population size, consistent with harsher conditions and limited access to arctic breeding grounds [4, 29–31]. The demographic pattern we observe in wild ducks is similar to that observed in wild boars [5], wild yaks [32], and wild horses [33]. However, it is worth noting that although PSMC is a powerful method to infer changes in N_e_ over time, it is also sensitive to deviations from a neutral model. The effects of genetic drift and/or selection could lead to time-dependent estimates of mutation rate and could bias our estimates of population expansion [26].

We observed three genetic clusters, with wild mallard, meat breeds, and egg/dual purpose breeds each representing unique groups. These results suggest either a single domestication event followed by subsequent breed-specific selection or two separate domestication events. In order to distinguish alternative models of domestication, we modeled population demographics and found strong support for a single domestication event roughly 2,200 years ago, with the rapid subsequent selection for separate meat and egg/dual-purpose breeds roughly 100 generations later. Difficulty in differentiating between very recent divergence and high migration rates in the frequency spectrum prevented convergence between independent runs when trying to fit other migration parameters to our model. We note that the evolutionary history of wild mallards and domesticated duck breeds is likely to be more complex than the simple demographic scenarios modeled here, and further studies may be needed to fully capture the evolutionary dynamics of duck domestication. Given the recent origin of wild ducks, as well as the high levels of diversity we observe in the wild and domestic duck genomes, it is not possible to differentiate recent admixture from incomplete lineage sorting with our current data. This issue has important conservation implications and represents an interesting area for future study. Nevertheless, the time estimates obtained with our model are compatible with previous written records from 500 BC [15].

### Selection for white plumage

Plumage color is an important domestication trait, and we compared breeds with white plumage to those with colored plumage. We identified high levels of divergence in the intronic region of the *MITF* gene, an important developmental locus with a complex regulation implicated in pigmentation and melanocyte development in several vertebrate species [34–36], including Japanese quail [37], dog [38], and duck [39, 40].

### Selection for other domestication traits

In order to identify those genomic regions that have been the target of selection during domestication, we used estimates of diversity between wild and domestic samples, retaining those 292 genes in the top 5% of both F_ST_ and *θ_π_* values for further analysis. These genes were overrepresented for both neural developmental and lipid metabolism, suggesting that these functionalities were under strong selection during domestication. Two loci, *GRIK2 and GRIN3A*, showed particularly strong signs of selective sweeps presumably associated with domestication. *GRIK2* encodes a subunit of a glutamate receptor that has a role in synaptic plasticity and is important for learning and memory. *GRIN3A* encodes a subunit of the N-methyl-D-aspartate receptors, which are expressed abundantly in the human cerebral cortex [41] and are involved in the development of synaptic elements.

We also identified five genes with significantly different expression in the brain and liver of domesticated ducks compared to their wild ancestor. One of these, *PDC* also showed evidence of selective sweeps at the genomic level. *PDC* encodes phosducin, a photoreceptor-specific protein that is highly expressed in the retina and the pineal gland [42], as well as the brain [43].

Our results suggest that *PDC*, *GRIK2*, and *GRIN3A* may have played a crucial role in duck domestication by altering functional regulation of the developing brain and nervous system. This finding is consistent with theories that behavioral traits are the most critical in the initial steps of animal domestication, allowing animals to tolerate humans and captivity [44, 45]. Indeed, compared to wild mallards, domestic ducks are more docile, less vigilant, and show important differences in brain morphology [17, 18]. Interestingly, differences between wild and domesticated animals in brain and nervous system functions due to directional selection were also observed in domestication studies of rabbits [7], dogs [46], and chickens [8]. In particular, *GRIK2* was also found to play a crucial role during rabbit domestication [7].

In addition to brain- and nervous system-related genes, we also identified several genes that play an important function in lipid and energy metabolism. For example, *PIK3C3* plays an important role in ATP binding but also regulates brain development and axons of cortical neurons [47–51]. *PNPLA8* is involved in facilitating lipid storage in adipocyte tissue energy mobilization and maintains mitochondrial integrity [52, 53], as well as plays a role in lipid metabolism associated with neurodegenerative diseases [54–56]. *PRKAR2B* is associated with body weight regulation, hyperphagia, and other energy metabolism [57, 58].

Taken together, our results show that duck domestication was a relatively recent and complex process, and the genetic basis of domestication traits show many striking overlaps with other vertebrate domestication events. The whole-genome resequencing data and SNP and INDEL variant datasets are valuable resources for researchers studying evolution, domestication, and trait discovery and for breeders of *Anas platyrhynchos*. Furthermore, the data represent a foundation for development of new, ultrahigh-density variant screening arrays for duck population level trait analysis and genomic selection.

## Methods

### Sample selection

A total of 78 ducks were chosen for sequencing, seven populations of domesticated ducks and two populations of mallards from different geographic regions. The domesticated ducks include three meat type populations, i.e., Pekin duck (PK; n = 8), Cherry Valley duck (CV; n = 8), and maple leaf duck (ML; n = 8); three egg type populations, i.e., Jin Ding duck (JD; n = 8), Shao Xing duck (SX; n = 8), and Shan Ma duck (SM; n = 8); one egg and meat dual-purpose type (DP type) population, i.e., Gao You duck (GY; n = 8); and two wild populations come from two provinces in China separated by nearly 2,000 km, i.e., mallard from Ningxia Province (MDN; n = 8) and mallardform Zhejiang Province (MDZ; n = 14). The classification of production types follow the description of Animal Genetic Resources in China Poultry [59]. PK, CV, and ML ducks originated from Beijing; JD and SM ducks originated from Fujian Province; and SX and GY ducks originated from Jiangsu Province. Whole blood samples were collected from brachial veins of ducks by standard venipuncture.

In addition, 14 male ducks (MDNM, n = 3; MDZM, n = 4; PKM, n = 1; CVM, n = 1; MLM, n = 1; JDM, n = 1; SMM, n = 1; SXM, n = 1; GYM, n = 1) were chosen for RNA-seq.

Sequencing and mapping statistic of individual ducks in genome and transcriptome analyses are detailed in the Supplementary files (Supplemental Tables S1, S7).

### Sequencing and library preparation

Genomic DNA was extracted using the standard phenol/chloroform extraction method. For each sample, two paired-end libraries (500 bp) were constructed according to the manufacturer's protocols (Illumina) and sequenced on the Illumina Hiseq 2500 sequencing platform. We sequenced each sample at 5X depth in order to reduce the false-negative rate of variants due to our strict filter criteria. We randomly selected one individual for 10X coverage, except for the MDN population, where we sequenced seven individuals at 5X coverage and random one at 20X coverage and the MDZ population, where we sequenced all individuals at 10X coverage. We generated 628.37 Gb of paired-end reads of 100 bp (or 150 bp; MDZ) length (Supplemental Table S1).

The mRNA from brain, liver, and breast muscle of 14 ducks were extracted using the standard trizol extraction methods. For each sample, two paired-end libraries (500 bp) were constructed according to the manufacturer's instruction (Illumina). All samples were sequenced using Illumina Hiseq 4000 sequencing platform with the coverage of 6X. We generated 278.62 Gb of paired-end reads of 150 bp length (Supplemental Table S9).

### Read alignment and variant calling

To avoid low-quality reads, mainly the result of base-calling duplicates and adapter contamination, we filtered out sequences according to the default parameters of NGS QC Toolkit (v2.3.3) [60]. Those paired reads that passed Illumina's quality control filter were aligned using BWA-MEM (v0.7.12) to version 1.0 of the *Anas platyrhynchos* genome (BGI_duck_1.0) [10]. Duplicate reads were removed from individual sample alignments using Picard tools MarkDuplicates, and reads were merged using MergeSamFiles [61].

The Genome Analysis Toolkit v3.5 (GATK, RRID:SCR_001876), RealignerTargetCreator, and IndelRealigner protocol were used for global realignment of reads around INDELs before variant calling [62, 63]. SNPs and small INDELs (1–50 bp) were called using the GATK UnifiedGenotyper set for diploids with the parameter of a minimum quality score of 20 for both mapped reads and bases to call variants, similar to previous studies [64–68]. We filtered variants both per population and per individual using GATK according to the stringent filtering criteria. For SNPs of population filter: a) QUAL >30.0; b) QD >5.0; c) FS <60.0; d) MQ >40.0; e) MQRankSum >-12.5; and f) ReadPosRankSum >-8.0.Additionally, if there were more than 3 SNPs clustered in a 10-bpwindow, all three SNPs were considered as false positives and removed [69].

We used the following population criteria to identify INDELs: QUAL >30.0, QD >5.0, FS <200.0, ReadPosRankSum >-20.0. Of individual filters, we also removed all INDELs and SNPs where the depth of derived variants was less than half the depth of the sequence. All SNPs and INDELs were assigned to specific genomic regions and genes using SnpEff v4.0 (SnpEff, RRID:SCR_005191) [70] based on the Ensembl duck annotations. After filtering, 36,107,949 SNPs and 3,082,731 INDELs were identified (Supplemental Table S2).

### SNP validation

In order to evaluate the reliability of our data, we compared our SNPs to the duck dbSNP database deposited in the GVM at the Big Data Center at the Beijing Institute of Genomics, Chinese Academy of Science [71]. A total of 7,908,722 SNPs were validated in the duck dbSNP database, which covered 96.2% of the database (Supplemental Table S2). For the 28,199,227 SNPs not confirmed by dbSNPs, 390 randomly selected nucleotide sites were further validated using diagnostic PCR combined with Sanger sequence method described in previous researchM [8, 72, 73]. The result showed 100% accuracy, indicating the high reliability of the called SNP variation identified in this study.

### Population structure

We removed all SNPs with a minor allele frequency < = 0.1 and kept only SNPs that occurred in more than 90% of individuals. Vcf files were converted to hapmap format with custom perl scripts and to PLINK format file by GLU v1.0b3 [74] and PLINK v1.90 (PLINK, RRID:SCR_001757) [75, 76], when appropriate. We used GCTA (v1.25) [77] for PCA, first by generating the genetic relationship matrix from which the first 20 eigenvectors were extracted.

To estimate individual admixture assuming different numbers of clusters, the population structure was investigated using FRAPPE v1.1 [22] base on all high-quality SNPs information, with a maximum likelihood method. We increased the coancestry clusters spanning from 2 to 4 (Supplemental Fig. S6), because there are four duck types (wild, meat, egg, and dual-purpose) across the nine duck populations, with 10,000 iterations per run.

A distance matrix was generated by calculating the pairwise allele sharing distance for each pair of all high-quality SNPs. Multiple alignment of the sequences was performed with MUSCLE v3.8 (MUSCLE, RRID:SCR_011812) [78]. A neighbor-joining maximum likelihood phylogenetic tree was constructed with the DNAML program in the PHYLIP package v3.69 (PHYLIP, RRID:SCR_006244) [79] and MEGA7 [80, 81]. All implementation was performed according to the recommended manipulations of SNPhylo [82].

### Demographic history reconstruction

The demographic history of both wild and domesticated ducks was inferred using a hidden Markov model approach as implemented in pairwise sequentially Markovian coalescence based on SNP distributions [23]. In order to determine which PSMC (v0.6.5) settings were most appropriate for each population, we reset the number of free atomic time intervals (-p option), upper limit of time to most recent common ancestor (-t option), and initial value of r = θ/ρ (-r option) according to previous research [26] and online suggestions by Li and Durbin [83]. Based on estimates from the chicken genome, an average mutation rate (μ) of 1.191 × 10^-9^ per base per generation and a generation time (g) of 1 year were used for analysis [84].

Three-population demographic inference was performed using a diffusion-based approach as implemented in the program ∂a∂i (v1.7) [85]. To minimize potential effects of selection that could interfere with demographic inference, these analyses were performed using the subset of noncoding regions across the whole genome and spanning 750,939,264 bp in length. Noncoding SNPs were then thinned to 1% to alleviate potential linkage between the markers. The final dataset consisted of 95,181 SNPs with an average distance of 7,112 bp (± 18,810 bp) between neighboring SNPs. To account for missing data, the folded allele frequency spectrum for the three populations (wild, meat, and egg/dual-purpose breeds) was projected down in ∂a∂i to the projection that maximized the number of segregating SNPs, resulting in 92,966 SNPs.

We tested four scenarios to reconstruct the demographic history of the domesticated breeds of mallards: simultaneous domestication of the meat and egg and dual-purpose breeds (model 1); a single domestication event followed by divergence of the meat and egg and dual-purpose breeds (model 2); two independent domestication events, with the meat type breed being domesticated first (model 3); and two independent domestication events, with the egg and dual-purpose breeds being domesticated first (model 4). Using the “backbone” of the best model, we then used a step-wise strategy to add parameters related with variation in population sizes and population growth, keeping a new parameter only if the AIC and log likelihood improved considerably over the previous model with fewer parameters. In cases where additional parameters resulted in negligibly improved AIC and likelihood, we retained the simpler, less parameterized model. Gene flow was modeled as continuous migration events after population divergence. Each model was run at least 10 times from independent starting values to ensure convergence to the same parameter estimates. We rejected models where we failed to obtain convergence across the replicate runs. Scaled parameters for the best-supported model were transformed into real values using the same average mutation rate (*μ*) and (*g*) as described above for the PSMC analysis. Parameter uncertainty was obtained using the Godambe information matrix [86] from 100 nonparametric bootstraps.

### Selective-sweep analysis

In order to define candidate regions that have undergone directional selection during duck domestication, we calculated the coefficient of nucleotide differentiation (F_ST_) between mallards and domesticated ducks described by Weir and Cockerham [87]. We calculated the average F_ST_ in 10-kbwindows with a 5-kbshift for all seven domesticated duck populations combined and two mallard populations combined. Only scaffolds longer than 10 kb, 2,368 of 78,488 scaffolds, were chosen for the analysis. We transformed observed F_ST_ values to Z transformation (Z(F_ST_)) with μ = 0.1154 and σ = 0.0678 according to previously described methods [88].

To estimate levels of nucleotide diversity (π) across all sampled populations, we used the VCFtools software (v0.1.13) [89] to calculate θπ(wild/domesticated) [90], computing the average difference per locus over each pair of accessions. As the measurement of F_ST_, averaged π ratio (θπ(wild/domesticated)) was calculated for each scaffold in 10–kbsliding windows.

Functional classification of GO categories was performed in Database for Annotation, Visualization and Integrated Discovery (DAVID, v6.8) [91]. Statistical significance was accessed by using a modified Fisher exact test and Benjamini correction for multiple testing.

### RNA-seq and data processing

To infer whether novel allelic variants located in the top 5% F_ST_ regions of genome comparison between wild mallards and domesticated ducks could also affect gene expression, we compared gene expression in brain, liver, and breast muscle between wild mallards and domesticated ducks. To make our result more universal, seven male mallards and seven male domesticated ducks were choose for RNA-seq. All samples were individually sequenced using the Illumina Highseq 4000 sequencing platform.

For each sample, adapters and primers of paired-end reads were removed using the NGSQC Tool kit (v2.3.3) [60]. For each paired-end read pair, if one of two reads had an average base quality less than 20 (PHRED quality score), then both reads were removed. If one end of a paired-end read had a percentage of high-quality base less than 70%, the two paired reads were also removed. After that, high-quality reads were mapped to the reference genome using STAR (v.2.5.3a) [92]. The *featureCounts* function of the *Rsubread* (v.1.5.2) [93, 94] was used to output the counts of reads aligning to each gene. We detected the differential expression genes with edgeR (v3.6) [95–98] using a p_adj_ <0.05 threshold.

## Availability of supporting data

The 78 ducks used in whole-genome resequencing analysis and the 14 ducks used in RNA–seq analysis are accessible at the National Center for Biotechnology Information (NCBI) under BioProject accession numbers PRJNA419832 and PRJNA419583, respectively. The unassembled sequencing reads of 78 ducks and RNA-seq reads of 14 ducks have been deposited in NCBI Sequence Read Archive under accession numbers SRP125660 and SRP125529, respectively. All VCF files of SNPs and INDELs and other supporting data, such as scripts, alignments for phylogenetic trees, and sweep regions, are available via the *GigaScience* database *Giga*DB [99].

## Additional file

Supplemental Figure S1: Distribution of variants in functional regions. SNPs distribution were showed on the left, and INDELs were showed on right. Most variants were synonymous mutations both in SNPs and in INDELs at genome wide across all populations.

Supplemental Figure S2: INDELs statistics of 9 population ducks. The largest INDEL detected in this study was 50 bp, and the majority of INDELs were less than 10 bp. Single base-pair INDEL was the predominant form and accounted for 38.63% of all detected INDELs. Both count and percentage were mean value of 9 population ducks.

Supplemental Figure S3: Comparison of four demographic models for the domestication of meat and egg/dual purpose breeds of mallards using ∂a∂i. The top panel shows the distribution of the log-likelihood for each one of the tested models and the middle panel the distribution of the Akaike information criterion (AIC) with outliers excluded. *Model 1:* simultaneous domestication of the meat and egg and dual purpose breeds; *Model 2:* a single domestication event followed by divergence of the meat and egg and dual purpose breeds; *Model 3:* two independent domestication events, with the meat type breed being domesticated first; *Model 4:* two independent domestication events, with the egg and dual purpose breed being domesticated first.

Supplemental Figure S4: Demographic history of meat and egg/dual purpose breed domestication using the best fit model inferred by ∂a∂i. (A) Model of single domestication event with changes in population sizes and migration. Time units are in years before present and migration are in units of number of migrants per generation. (B) Site frequency spectrum for the three populations of domesticated and wild mallards. The frequency spectrum is shown for the data (first row) and for the best fit model (second row). The last two rows show the normalized difference (i.e., residuals) between model and data for each bin in the spectrum.

Supplemental Figure S5: White plumage related variants of MITF validation by Sanger sequence in 78 ducks. Three SNPs and one INDEL of MITF was amplified by diagnostic PCR and sequenced by Sanger method, resulted completely matched with the analysis result of NGS. White plumage ducks contains PK, CV, and ML; non-white plumage ducks contains MDN, MDZ, JD, SM, SX, and GY.

Supplemental Figure S6: Population genetic structure of 78 ducks. The length of each colored segment represents the proportion of the individual genome inferred from ancestral populations (K = 2–4). The population names and production type are at the bottom. DP type means dual-purpose type. With K = 2, a clear division was found between wild type ducks (MDN and MDZ) and domesticated ducks (PK, CV, ML, JD, SM, SX, and GY). With K = 3, a clear division was found between meat type ducks (PK, CV, and ML) and egg type ducks mixed with dual-purpose type ducks (JD, SM, SX, and GY). With K=4, a clear division was found between egg type ducks (JD, SM, and SX) and dual-purpose type ducks (GY).

Supplemental Table S1: Summary of genome sequencing and mapping statistic.

Supplemental Table S2: Summary of SNPs and INDELs.

Supplemental Table S3: INDELs statistics of 9 population ducks.

Supplemental Table S4: global Fst between each population.

Supplemental Table S5: gene name in top 5% sweep regions.

Supplemental Table S6: Total GO terms of genes located in top 5% FST and θπ regions.

Supplemental Table S7: Summary of results from enrichment analysis of neuronal and lipid related in regions of top 5% FST and θπ.

Supplemental Table S8: gene name in top 1% sweep regions.

Supplemental Table S9: summary of transcriptome sequencing and mapping statistic.

## Abbreviations

AIC: Akaike information criteria; CI: confidence interval; GO: gene ontology; GVM: Genome Variation Map; INDEL: insertion and deletion; MITF: melanogenesis-associated transcription factor; NCBI: National Center for Biotechnology Information; PCA: principle component analysis; PCR: polymerase chain reaction; PSMC: pairwise sequentially Markovian coalescent; RNA-seq: RNA sequencing; SNP: single-nucleotide polymorphism.

## Ethics statement

The entire procedure was carried out in strict accordance with the protocol approved by the Animal Welfare Committee of China Agricultural University (permit XK622).

## Funding

This work was supported by the Earmarked fund for the Beijing Innovation Team of the Modern Agro-industry Technology Research System (BAIC04–2017), European Research Council (grant agreement 680951), and Wolfson Merit Award. We gratefully acknowledge our colleagues in the Poultry Team at the National Engineering Laboratory for Animal Breeding of China Agricultural University for their assistance with sample collection and helpful comments on the manuscript.

## Author contributions

Conceived and designed the experiments: L.Q. Wrote the paper: Z.Z. Revised the paper: L.Q., J.E.M., M.vanT. Analyzed the data: Z.Z., P.A., Q.W., Y.J. Performed the experiments: Z.Z., Y.J. Contributed reagents/materials: Z.J., Y.C., K.Z., S.H., Z.Zhou, H.L., F.Y., Y.H., Z.N., and N.Y.

## Supplementary Material

GIGA-D-17-00301_Original_Submission.pdf

GIGA-D-17-00301_Revision_1.pdf

GIGA-D-17-00301_Revision_2.pdf

Response_to_Reviewer_Comments_Original_Submission.pdf

Response_to_Reviewer_Comments_Revision_1.pdf

Reviewer_1_Report_(Original_Submission) -- Martin Johnsson 07 Dec 2017 Reviewed

Reviewer_1_Report_(Revision_1) -- Martin Johnsson 06 Feb 2018 Reviewed

Reviewer_2_Report_(Original_Submission) -- Philip Lavretsky 11 Dec 2017 Reviewed

Reviewer_2_Report_(Revision_1) -- Philip Lavretsky 12 Feb 2018 Reviewed

Reviewer_3_Report_(Original_Submission) -- Henner Simianer, Prof. 20 Dec 2017 Reviewed

Supplemental material
