# Supplementary material for: Whole-genome resequencing reveals signatures of selection and timing of duck domestication
Source: Gigascience. 2018 Apr 9;7(4):giy027. doi: 10.1093/gigascience/giy027 (PMC6007426; doi:10.1093/gigascience/giy027)
Supplement: GIGA-D-17-00301_Original_Submission.pdf [file giy027_giga-d-17-00301_original_submission.pdf]

GigaScience

Whole-genome resequencing reveals signatures of selection and timing of duck domestication

--Manuscript Draft--

Manuscript Number:	GIGA-D-17-00301
Full Title:	Whole-genome resequencing reveals signatures of selection and timing of duck domestication
Article Type:	Research
Funding Information:	Beijing Innovation Team of the Modern Agro-industry Technology Research System (BAIC04-2017) — Prof. Lujiang Qu<br>European Research Council (680951) — Dr. Judith E Mank<br>Wolfson Merit Award — Dr. Judith E Mank
Abstract:	The genetic basis of animal domestication remains poorly understood, and systems with substantial phenotypic differences between wild and domestic populations are useful for elucidating the genetic basis of adaptation to new environments as well as the genetic basis of rapid phenotypic change. Here, we sequenced the whole genome of 78 individual ducks, from two wild populations and seven domesticated populations, with an average sequencing depth > 45X for each population. Our population and demographic analysis indicates a complex history of domestication, with early selection for separate meat and egg lineages. Genomic comparison of wild to domesticated populations suggest that genes affecting brain and neuronal development have undergone strong positive selection during domestication. Our FST analysis also first indicates the duck white plumage associated with selection at the melanogenesis associated transcription factor locus. Our results advance the understanding of animal domestication and selection for complex phenotypic traits.
Corresponding Author:	Lujiang Qu, Ph.D.<br>China Agricultural University<br>Beijing, CHINA
Corresponding Author Secondary Information:	
Corresponding Author's Institution:	China Agricultural University
Corresponding Author's Secondary Institution:	
First Author:	Zebin Zhang
First Author Secondary Information:	
Order of Authors:	Zebin Zhang<br>Yaxiong Jia<br>Pedro Almeida<br>Judith E Mank<br>Marcel van Tuinen<br>Qiong Wang<br>Zhihua Jiang<br>Yu Chen<br>Kai Zhan<br>Shuisheng Hou

Powered by Editorial Manager® and ProduXion Manager® from Aries Systems Corporation

	Zhengkui Zhou
	Huifang Li
	Fangxi Yang
	Yong He
	Lujiang Qu, Ph.D.
**Order of Authors Secondary Information:**	
**Opposed Reviewers:**	Yinhua Huang<br>yinhuahuang@126.com<br>scientific interest conflict
	Erik Axelsson<br>erik.axelsson@imbim.uu.se<br>scientific interest conflict
	Matthew Webster<br>mattew.webster@imbim.uu.se<br>scientific interest conflict
	Zhuocheng Hou<br>zchou@cau.edu.cn<br>scientific interest conflict
**Additional Information:**	
**Question**	**Response**
Are you submitting this manuscript to a special series or article collection?	No
**Experimental design and statistics**<br><br>Full details of the experimental design and statistical methods used should be given in the Methods section, as detailed in our Minimum Standards Reporting Checklist. Information essential to interpreting the data presented should be made available in the figure legends.<br><br>Have you included all the information requested in your manuscript?	Yes
**Resources**<br><br>A description of all resources used, including antibodies, cell lines, animals and software tools, with enough information to allow them to be uniquely identified, should be included in the Methods section. Authors are strongly encouraged to cite Research Resource Identifiers (RRIDs) for antibodies, model organisms and tools, where possible.<br><br>Have you included the information requested as detailed in our Minimum Standards Reporting Checklist?	Yes

Powered by Editorial Manager® and ProduXion Manager® from Aries Systems Corporation

**Availability of data and materials**<br><br>All datasets and code on which the conclusions of the paper rely must be either included in your submission or deposited in publicly available repositories (where available and ethically appropriate), referencing such data using a unique identifier in the references and in the "Availability of Data and Materials" section of your manuscript.<br><br>Have you have met the above requirement as detailed in our Minimum Standards Reporting Checklist?	Yes

Powered by Editorial Manager® and ProduXion Manager® from Aries Systems Corporation

Manuscript
Click here to download Manuscript Manuscript_of_Duck_Domestication.docx

Whole-genome resequencing reveals signatures of selection and timing of duck domestication

Zebin Zhang †,[1], Yaxiong Jia†,[2], Pedro Almeida[3], Judith E Mank[3,4], Marcel van Tuinen[5], Qiong Wang[1], Zhihua Jiang[6], Yu Chen[7], Kai Zhan[8], Shuisheng Hou[2], Zhengkui Zhou[2], Huifang Li[9] Fangxi Yang[10], Yong He[11], and Lujiang Qu[1*]

[1]Department of Animal Genetics and Breeding, National Engineering Laboratory for Animal Breeding, College of Animal Science and Technology, China Agricultural University, Beijing, China

[2]Institute of Animal Science, Chinese Academy of Agricultural Sciences, Beijing, China

[3]Department of Genetics, Evolution and Environment, University College London, London, UK

[4]Department of Organismal Biology, Evolutionary Biology Centre, Uppsala University, Uppsala, Sweden

[5]Centre of Evolutionary and Ecological Studies, Marine Evolution and Conservation Group, University of Groningen, Groningen, The Netherlands

[6]Department of Animal Sciences, Center for Reproductive Biology, Veterinary and Biomedical Research Building, Washington State University, Pullman, United States

[7]Beijing Municipal General Station of Animal Science, Beijing, China

[8]Institute of Animal Husbandry and Veterinary Medicine, Anhui Academy of

Agricultural Sciences, Hefei, China

[9]Poultry Institute, Chinese Academy of Agriculture Science, Yangzhou, China

[10]Institute of Pekin Duck，Beijing, China

[11]Cherry Valley farms (xianghe) Co.,Ltd, Langfang, China

[†]These authors contributed equally to this work.

*Corresponding authors: quluj@163.com

Abstract

The genetic basis of animal domestication remains poorly understood, and systems with substantial phenotypic differences between wild and domestic populations are useful for elucidating the genetic basis of adaptation to new environments as well as the genetic basis of rapid phenotypic change. Here, we sequenced the whole genome of 78 individual ducks, from two wild populations and seven domesticated populations, with an average sequencing depth > 45X for each population. Our population and demographic analysis indicates a complex history of domestication, with early selection for separate meat and egg lineages. Genomic comparison of wild to domesticated populations suggest that genes affecting brain and neuronal development have undergone strong positive selection during domestication. Our F_{ST} analysis also first indicates the duck white plumage associated with selection at the *melanogenesis associated transcription factor* locus. Our results advance the understanding of animal domestication and selection for complex phenotypic traits.

Keywords: duck, domestication, intensive selection, neuronal development, energy metabolism, plumage colouration.

Introduction

Anas platyrhynchos (ducks or mallards) are the world's most widely distributed and agriculturally important waterfowl, and are of particular economic and importance in Asia [1]. Although forms of the mallard have been farmed in Asia for over a thousand years, the exact timing of domestication remains unknown, with written records indicating domestic ducks in central China shortly after 500 BC [2]. Moreover, domesticated mallards show many important behavioral [3] and morphological [4-6] differences from their wild ancestors, particularly related to plumage and neuroanatomy, offering an important opportunity to understand the genetic basis of these phenotypic differences.

In order to determine the timing of duck domestication in China, as well as identify the genomic regions under selection during domestication, we performed whole genome resequencing from 78 individuals belonging to seven different duck breeds (three for meat breeds, three for egg breeds, and one dual-purpose breed) and two geographically distinct wild populations. Using the 36.1 million single nucleotide polymorphisms (SNPs) and 3.1 million small insertions and deletions (INDELs), we analyzed the structure of these populations and signatures of selection associated with domestication. We identified two distinct domesticated populations, originating from a single domestication event roughly 2000 years ago. We also identified signatures of selection on genes associated with neuronal development, energy metabolism,

Results

Genetic variation

We individually sequenced 16 wild and 62 domestic ducks, from two wild populations and seven domestic breeds (three meat breeds, three egg breeds and one dual-purpose breed), from across China (Fig. 1A) to an average of 6.42X coverage per individual after filtering and quality control, resulting in total 535 billion mappable reads(Supplemental Table S1).

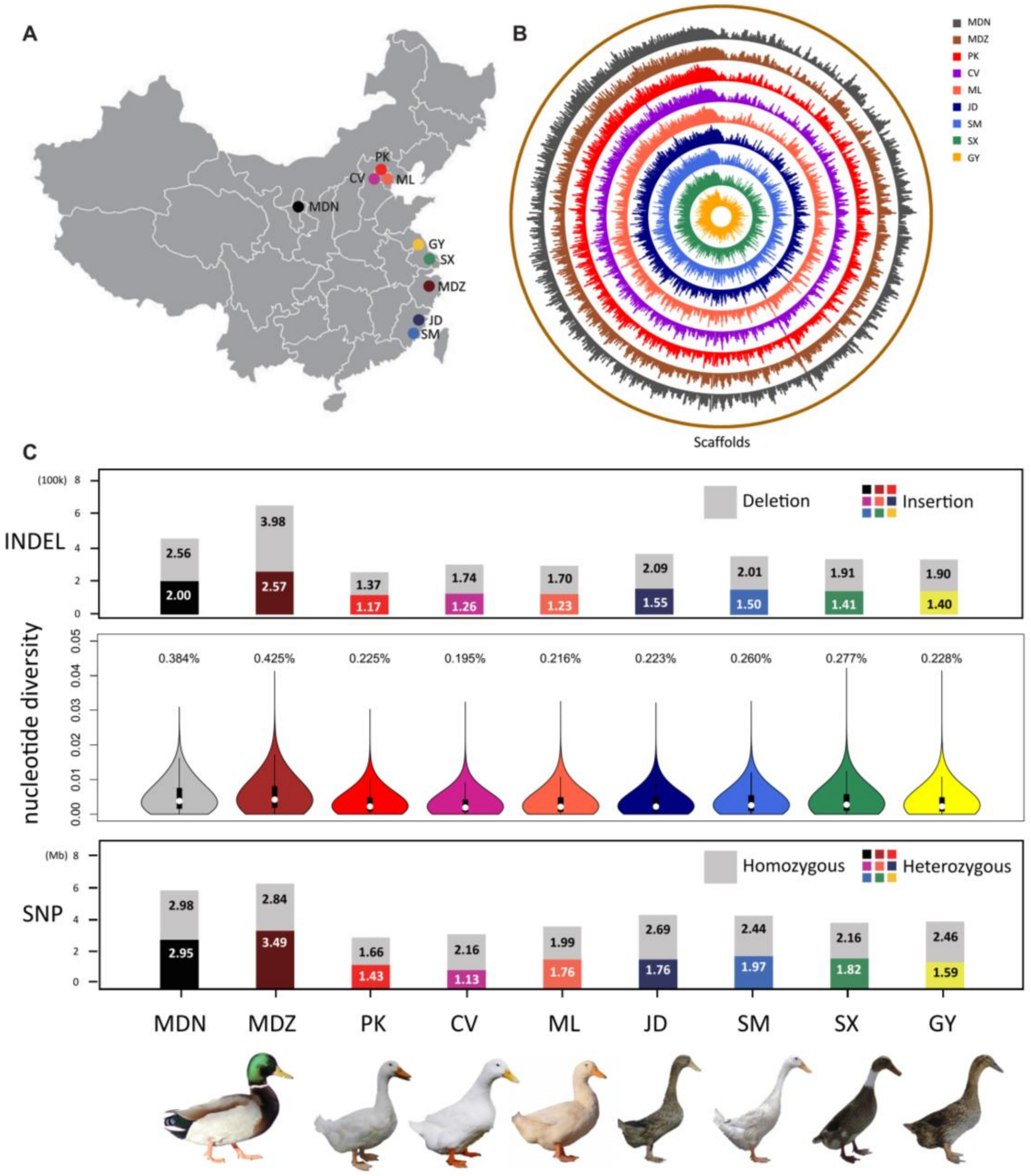

Figure. 1 Experimental design and variants statistics

(A) Sampling sites in this study. A total of 78 ducks from two wild populations (Mallard Ningxia (MDN) n=8; Mallard Zhejiang (MDZ) n=14), three meat breeds (Pekin (PK) n=8; Cherry Valley (CV) n=8; Maple Leaf (ML) n=8), three egg breeds (Jin Ding (JD) n=8; Shan Ma (SM) n=8; Shao Xing (SX) n=8), and one dual breed (Gao You (GY) n=8) were selected.

(B) Circos plot of SNP distribution and density of seven domestic breeds and two wild

populations across the genome. The duck whole genome reference is shown in the outermost circle (non-overlapping, window size = 1 Mb).

(C) Genomic variation of nine population ducks. Mean number of SNPs, heterozygous and homozygous SNP ratio in the nine populations as shown at the bottom. Homozygous SNP ratios in domesticated ducks are significantly higher than ratios in wild mallards ($p = 1.35 \times 10^{-10}$). Nucleotide diversity ratio in the nine populations are shown at the middle. The nucleotide diversity ratio in wild mallards are dramatically higher than ratios in domesticated ducks ($p = 2.20 \times 10^{-16}$). Number of insertions and deletions in the nine populations are shown at the top. The number of deletion was higher than insertion in all nine populations.

We detected 36.1 million (M) SNPs in total, with an average for each individual of 4.5M (range = 2.34 – 9.52M), which covered 96.2% of the duck dbSNP database deposited in the Genome Variation Map (GVM) (http://bigd.big.ac.cn/gvm/). We also identified 3.1M INDELs, with an average of 0.4M (range = 0.21 – 0.89M) (Fig. 1C, Supplemental Figs. S1 - S2, Supplemental Table S2). Both the number of SNPs (t test, $p = 3.13 \times 10^{-12}$) and nucleotide diversity (t test, $p = 2.20 \times 10^{-16}$) are lower in domesticated compared to wild mallards (Fig. 1B - C), consistent with the larger panmictic wild population. Single base-pair INDELs were the predominant form, accounting for 38.63% of all detected INDELs (Supplemental Table S3).

Population structure and domestication

Phylogenetic relationships, based on a neighbor-joining (NJ) of pairwise genetic distances of whole genome SNPs (Fig. 2A) and Principal Component Analysis (PCA, Fig. 2B) revealed strong clustering into three distinct genetic groups. The two wild populations (MDN and MDZ) clustered together, with the three meat type population ducks (PK, CV, and ML) clustered together into a second group, and the three egg type populations (JD, SM, and SX) clustered with the dual-purpose type ducks (GY) into a third group.

We further performed population structure analysis using FRAPPE [7], which estimates individual ancestry and admixture proportions assuming K ancestral populations (Fig. 2C). With K = 2, a clear division was found between wild type ducks (MDN and MDZ) and domesticated ducks (PK, CV, ML, JD, SM, SX, and GY). With K = 3, a clear division was found between meat type ducks (PK, CV, and ML) and egg type ducks mixed with dual-purpose type ducks (JD, SM, SX, and GY).

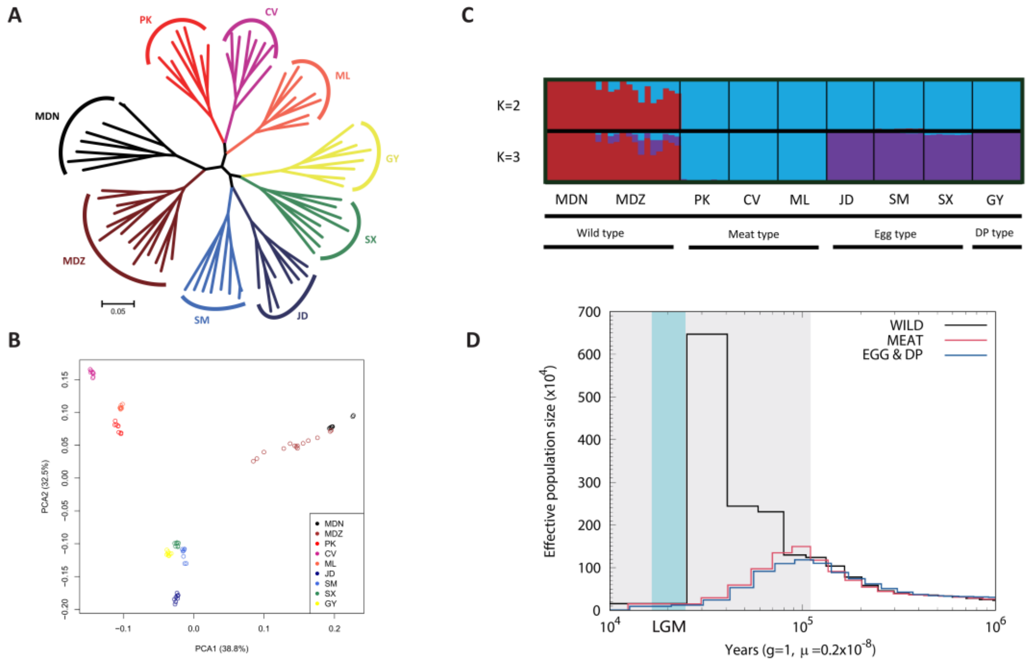

Figure. 2 Population genetic structure and demographic history of nine duck populations

(A) Neighbor-joining phylogenetic tree of nine duck populations. The scale bar is proportional to genetic differentiation (p dist ance).

(B) PCA plot of duck populations. Eigenvector 1 and 2 explained 38.8% and 32.5% of the observed variance, respectively.

(C) Population genetic structure of 78 ducks. The length of each colored segment represents the proportion of the individual genome inferred from ancestral populations (K = 2-3). The population names and production type are at the bottom. DP type means dual-purpose type.

(D) Demographic history of duck populations. Examples of PSMC estimate changes in the effective population size over time, representing variation in inferred Ne dynamics. The lines represent inferred population sizes and the gray shaded areas indicate the Pleistocene period, with Last Glacial Period (LGP) shown in darker gray, and Last Glacial Maximum (LGM) shown

in light blue areas.

Together, these results indicate two genetic clusters of domesticated breeds, either domesticated once with subsequent subdivision due to divergent selection, or domesticated twice independently. In order to differentiate these alternatives, we explored the demographic history of our samples, first estimating changes in effective population size (N_e) in our three genetic clusters in a pairwise sequentially Markovian coalescent (PSMC) framework [8]. The meat type ducks (PK, CV, and ML) showed concordant demographic trajectories with egg and mixture type populations (JD, SM, SX, and GY) with one apparent expansion around the Penultimate Glaciation Period (PGP, 0.30-0.13 Mya) [9,10] and Last Glacial Period (LGP, 110-12 kya) [11,12], followed by a subsequent contraction (Fig. 2D).

We tested multiple demographic scenarios related to domestication using a diffusion approximation method for the allele frequency spectrum (∂a∂i) (Supplemental Fig. S3 and S4). Among the four isolation models tested (models 1 - 4), the model of a single domestication with subsequent divergence of the domesticated breeds (Model 2) was both consistent with our population structure results (Fig. 2) and had the lowest Akaike Information Criteria (AIC), indicating a better overall fit to the data (log-likelihood = -33,388.43; AIC = 66,788) (Supplemental Fig. S3).

Demographic parameters estimated from the single domestication model

(Model 2) indicated that domestication occurred approximately 2,200 years ago, followed by a rapid subsequent divergence of the meat breed from the egg/dual purpose breeds roughly 100 years after the initial domestication event (Table 1). Our results suggest that following an initial bottleneck associated with domestication, with an estimated N_e of 305 individuals for the ancestral domesticated population, the population has expanded to the current N_e of 5,345 and 12,404 in the meat type and egg/dual purpose breeds respectively. N_e estimates for domesticated breeds are lower than that in wild mallards, consistent with the large panmictic wild population.

Table 1. Maximum likelihood population demographic parameters. Best fit parameter estimates for the model of a single domestication event followed by divergence of the domesticated breeds, including changes in population size. Time estimates are given in years and migration are in units of number of migrants per generation.

**Parameter**	**ML estimate**
N_e of ancestral population after size change	633,584
N_e of the wild population	84,845
N_e of the ancestral domesticated population	305
N_e of the meat breed	5,345
N_e of the egg/dual purpose	12,404
Time of size change in the ancestral population	238,696
Time of domestication	2,128
Time of breed divergence	2,030
Migration $_{wild \leftarrow meat}$	1.21
Migration $_{wild \leftarrow egg/dp}$	3.92

Gene flow estimates were relatively high, and were 1 and 4 migrants per generation from the meat and egg/dual purpose breeds, respectively, into the

wild population. Difficulty in differentiating between very recent divergence and high migration rates in the frequency spectrum prevented convergence between independent runs when trying to fit other migration parameters to our model.

Selection for plumage color

Derived traits in domesticated animals tend to evolve in a predictable order, with color variation appearing in the earliest stages of domestication, followed by coat or plumage and structural (skeletal and soft tissue) variation, and finally behavioral differences [13,14]. One of the simplest and most visible derived traits of ducks is white plumage color. In order to detect the signature of selection associated with white feathers, we searched the duck genome for regions with high F_{ST} among the populations of white feather (PK, CV, and ML) and non-white feather (MDN, MDZ, JD, SX, and GY) based on sliding windows of 10kb windows. We identified a region of high differentiation between white plumage and non-white plumage ducks overlapping the *melanogenesis associated transcription factor* (*MITF*; F_{ST}=0.69) (Fig. 3A). In the intronic region of *MITF*, we identified 13 homozygous SNPs and 2 homozygous INDELs present in all white plumage breeds (n=24). These SNPS were absent in all non-white plumage breeds (n=46) (Fig. 3B). These mutations were completely consistent with the white plumage phenotype suggesting as causative mutation. Our result first indicates the duck white plumage associated with selection at the *MITF* locus.

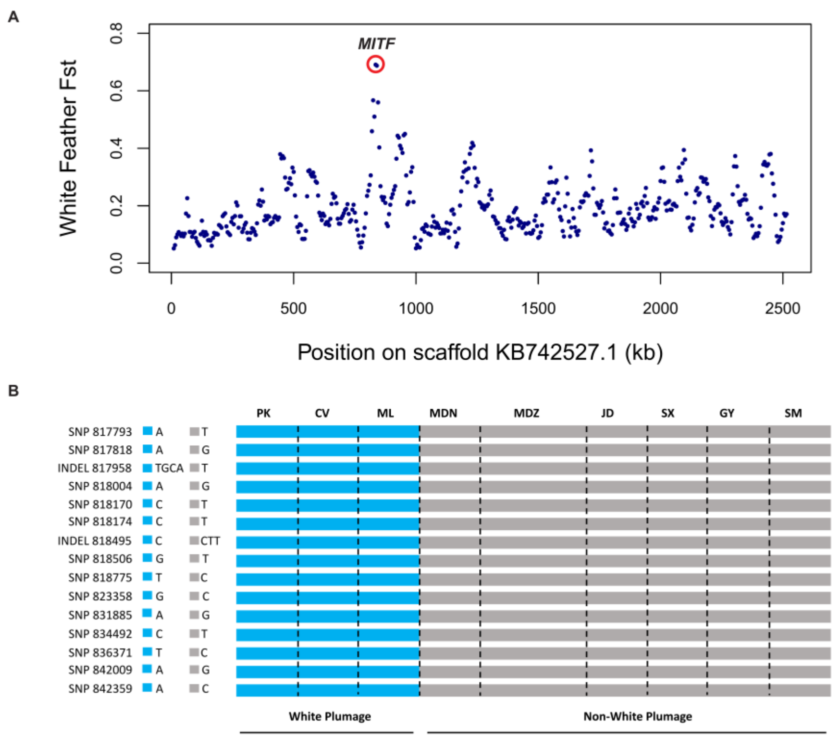

Figure. 3 MITF shows different genetic signature between white plumage and non-white plumage ducks.

(A) FST plot around the MITF locus. The FST value of MITF is highest for scaffold KB742527.1, circled in red. Each plot represent a 10 kb windows.

(B) 13 homozygous SNPs and 2 homozygous INDELs were identified in white plumage ducks and absent in non-white plumage ducks. SNPs and INDELs were named according to their position on scaffold.

Selection for other domestication traits

In order to detect the signature of selection for other traits associated with duck domestication, we scanned the duck genome for regions with a high coefficient of nucleotide differentiation (F_{ST}) among the populations of wild types (MDN and MDZ) and domesticated types (PK, CV, ML, JD, SM, SX, and GY)

based on sliding windows of 10kb windows. Owing to the complex and partly unresolved demography of these populations, it is difficult to define a strict threshold that distinguishes true sweeps from regions of homozygosity caused by drift. We therefore also calculated pairwise diversity ratio (θ_π(wild/domesticated)). We identified 292 genes in the top 5% of both F_{ST} and θ_π scores, putatively under positive selection during domestication (Fig. 4A, Supplemental Tables S4).

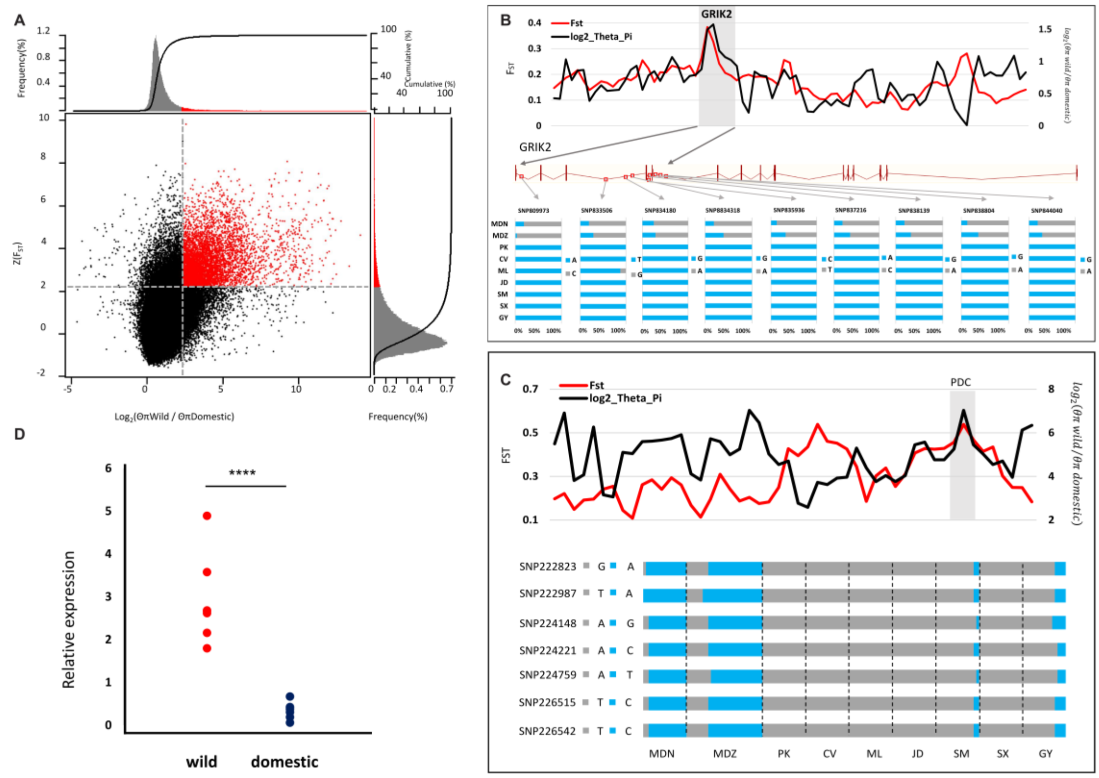

Figure. 4 Genomic regions with strong selective sweep signals in wild population ducks and domesticated population ducks.

(A) Distribution of θπ ratios θπ(wild/domesticated)) and Z(F_{ST}) values, which are calculated using scaffolds longer than 10-kb by 10-kb windows with 5-kb steps. Red data points located to the top-right regions correspond to the 5% right tails of empirical $log_2(\theta\pi\ wild/\theta\pi\ domestic)$ ratio distribution and the top 5% empirical Z(F_{ST}) distribution are

genomic regions under selection during duck domestication. The two horizontal and vertical gray lines represented the top 5% value of Z(F_{ST}) (2.216) and $log_2(\theta\pi\ wild/\theta\pi\ domestic)$ (2.375), respectively.

(B) $log_2(\theta\pi)$ ratios and F_{ST} values around the *GRIK2* locus and allele frequencies of nine SNPs within the *GRIK2* gene across nine duck populations. The black and red lines represent $log_2(\theta\pi\ wild/\theta\pi\ domestic)$ ratios and F_{ST} values, respectively. The gray bar showed the region of under strong selection in *GRIK2* gene. The nine red rectangular frame corresponding to the locus on gene of nine SNPs. The SNPs were named according to their position on scaffold.

(C) The PDC gene showed different genetic signature in domesticated and wild duck. $log_2(\theta\pi)$ ratios and F_{ST} values around the *PDC* locus. The *PDC* gene region was showed in gray par. Allele frequencies of seven SNPs within the *PDC* gene across nine duck populations. The SNPs were named according to their position on scaffold.

(D) The PDC gene expression level different in domesticated and wild duck. PDC mRNA expression levels in brain of wild (MDN, n=3; MDZ, n=4) and domesticated (PK, n=1; CV, n=1; ML, n=1; JD, n=1; SM, n=1; SX, n=1; GY, n=1) ducks. ****P value from t-test (P<0.0001).

Because domesticated ducks are known to differ from wild ducks in body size, body fat percentage, behavior, egg productivity, growth speed, and flight capability, we focused our analysis on GO annotations of neural related processes, lipid metabolism and energy metabolism, reproduction, and skeletal muscle contraction for our 292 putative positively selection genes. In this reduced data set, the neuro-synapse-axon and lipid-energy metabolism

pathways were over-represented (Supplemental Table S5) in our list of genes under selection.

From the highlighted GO terms, a total of 25 neuro-synapse-axon genes were identified as under positive selection, with six (*ADGRB3*, *EFNA5*, *GRIN3A*, *GRIK2*, *SYNGAP1*, and *HOMER1*) in the top 1% of F_{ST} and θ_{π} (Supplemental Tables S6). In particular, *GRIK2* (glutamate receptor, ionotropic kainate 2) and *GRIN3A* (glutamate receptor, subunit 3A) both showed high F_{ST} and θ_{π} value compared to neighboring regions, suggesting functional importance (Fig. 3B, Supplemental Table S4, S6).

Beyond the neuronal genes, 115 genes were identified in the four lipid and energy related pathways with high F_{ST} and θ_{π} values, particularly related to gatty acid metabolism. Among these genes, 37 genes were found with both parameters yielding top 1% ranked values (Supplemental Tables S6), such as phosphatidylinositol 3-kinase catalytic subunit type 3 (*PIK3C3*), and patatin like phospholipase domain containing 8 (*PNPLA8*).

To infer whether selection extends beyond yielding novel allelic variation by also affecting gene expression, we compared individual gene expression in the brain, liver, and in breast muscle between seven wild mallards and seven domesticated ducks with RNA-seq (Supplemental Tables S7). We detected three genes (*PDC*, *MLPH*, and *NID2*) in the brain, two genes (*MAPK12* and *BST1*) in the liver, and zero genes in breast muscle with significantly different expression between wild and domesticated ducks. Of the five differentially

expressed genes, *PDC* was the only gene which also showed evidence of a selective sweep at the genomic level (Supplemental Tables S4, Fig. 3C - D). The results imply that the *PDC* gene is of substantial functional importance in phenotypic differentiation among wild and domestic ducks through both allelic and expression differences.

Discussion

Animal domestication was one of the major contributory factors of the agricultural revolution during the Neolithic period, which resulted in a shift in human lifestyle from hunting to farming [15]. Since this transition, domesticated animals have contributed greatly to human society and human population growth by provision of stable animal protein, fat, and accessory products such as leather and feathers (including down). Whole genome sequencing has made it possible to illuminate the genetic trajectories of animal domestication such as those observed in pig [16], sheep [17], rabbit [18] and chicken [19,20].

In this study, we performed whole-genome sequencing of 78 ducks including seven domesticate breeds and two wild populations. This is the first study to characterize the genetic architecture, phylogenetic relationships and domestication history of domesticated ducks and wild mallards. We first catalogued millions of 36.1M SNPs and 3.1M INDELs, and in both cases, we observed higher mean variant numbers and nucleotide diversity for the wild mallard populations compared to the domestics, consistent with both a greater

panmictic mallard population as well as recent sweeps associated with domestication.

Population structure and domestication

We observed a large expansion of the duck population at the interglacial period, which could be the result of beneficial climatic changes, including rising temperatures and sea levels. In contrast, the glacial maximum coincided with a much reduced duck population size, consistent with harsher conditions and limited access to arctic breeding grounds [10,21-23]. The demographic pattern we observe in wild ducks is similar to that observed in wild boars [16], wild yaks [24], and wild horses [25]. However, it is worth noting that although PSMC is a powerful method to infer changes in N_e over time, it is also sensitive to deviations from a neutral model. The effects of genetic drift and/or selection could lead to time-dependent estimates of mutation rate, and bias our estimates of population expansion [12].

We observed three genetic clusters, with wild mallard, meat breeds, and egg/dual purpose breeds each representing unique groups. These results suggest either a single domestication event followed by subsequent breed-specific selection, or two separate domestication events. In order to distinguish alternative models of domestication, we modeled population demographics and found strong support for a single domestication event roughly 2,100 years ago, with the rapid subsequent selection for separate meat and egg/dual purpose breeds roughly 100 generations later. We note that the evolutionary history of

wild mallards and domesticated duck breeds is likely to be more complex than the simple demographic scenarios modelled here, and further studies may be needed to fully capture the evolutionary dynamics of duck domestication. Nevertheless, time estimates obtained with our model are compatible with previous written records from 500 BC [2].

Selection for white plumage

Plumage color is an important domestication trait, and we compared breeds with white plumage to those with colored plumage. We identified high levels of divergence in the intronic region of the *MITF* gene, an important developmental locus with a complex regulation implicated in pigmentation and melanocyte development in severval vertebrate species [26-28], including Japanese quail [29] and dog [30].

Selection for other domestication traits

In order to identify those genomic regions which have been the target of selection during domestication, we used estimates of diversity between wild and domestic samples, retaining those 292 genes in the top 5% of both F_{ST} and θ_π values for further analysis. These genes were over-represented for both neural developmental and lipid metabolism, suggesting that these functionalities were under strong selection during domestication. Two loci, *GRIK2 and GRIN3A*, showed particularly strong signatures of genetic sweep associated with domestication. *GRIK2* encodes a subunit of a glutamate

receptor that has a role in synaptic plasticity and is important for learning and memory. *GRIN3A* encodes a subunit of the N-methyl-D-aspartate (NMDAR) receptors, which is expressed abundantly in the human cerebral cortex [31] and is involved in the development of synaptic elements

We also identified five genes with significantly different expression in the brain and liver of domestics compared to their wild ancestor. One of these, *PDC*, also showed evidence of selective sweeps at the genomic level. *PDC* encodes phosducin, a photoreceptor-specific protein highly expressed in retina and pineal gland [32], as well as the brain [33].

Our results suggest that *PDC*, *GRIK2* and *GRIN3A* may have played a crucial role in duck domestication by altering functional regulation of the developing brain and nervous system. This finding is consistent with theories that behavioral traits are the most critical in the initial steps of animal domestication, allowing animals to tolerate humans and captivity [34,35]. Indeed, compared to wild mallards, domestic ducks are more docile, less vigilant, and show important differences in brain morphology [3,4]. Interestingly, differential selection on brain and nervous system functions was also observed in domestication studies of rabbits [18], dogs [36], chickens [19]. In particular, *GRIK2* was also found to play a crucial role during rabbit domestication [18].

Besides brain and nervous system related genes, we also identified several genes that play an important function in lipid and energy metabolism. For example, *PIK3C3* plays an important role in ATP binding but also regulates

brain development and axons of cortical neurons [37-41]. *PNPLA8* is involved in facilitating lipid storage in adipocyte tissue energy mobilization and maintains mitochondrial integrity [42,43], as well as plays a role in lipid metabolism associated with neurodegenerative diseases [44-46]. *PRKAR2B* is associated with body weight regulation, hyperphagia, and other energy metabolism [47,48].

Taken together, our results show that duck domestication was recent and complex process, and the genetic basis of domestication traits show many striking overlaps with other vertebrate domestication events.

Methods

Ethics statement

The entire procedure was carried out in strict accordance with the protocol approved by the Animal Welfare Committee of China Agricultural University (Permit Number: XK622).

Sample selection

78 ducks were chosen for sequencing, seven different populations of domesticated ducks and two population of mallards from different geographic regions. The domesticated ducks include three meat type populations *i.e.*, Pekin duck (PK; n=8); Cherry Valley duck (CV; n=8); Maple Leaf duck (ML; n=8), three egg type populations *i.e.*, Jin Ding duck (JD; n=8); Shao Xing duck (SX; n=8); Shan Ma duck (SM; n=8), one egg and meat dual-purpose type (DP type)

population *i.e.*, Gao You duck (GY; n=8), and two wild populations come from two different provinces in China with separated by nearly 2,000 km distance *i.e.*, Mallard from Ningxia province (MDN; n=8); Mallard form Zhejiang province (MDZ; n=14). The classification of production types follow the description of Animal Genetic Resources in China Poultry [49]. PK, CV, and ML ducks originated from Beijing; JD and SM ducks originated from Fujian province while SX and GY ducks originated from Jiangsu province. Whole blood samples were collected from brachial veins of ducks by standard venipuncture.

In addition, 14 male ducks (MDNM, n=3; MDZM, n=4; PKM, n=1; CVM, n=1; MLM, n=1; JDM, n=1; SMM, n=1; SXM, n=1; GYM, n=1) were chosen for RNA-seq.

Sequencing and mapping statistic of individual ducks in genome and transcriptome analysis were detailed in supplementary files (Supplemental Table S1, S7).

Sequencing and library preparation

Genomic DNA was extracted using standard phenol/chloroform extraction method. For each sample, two paired-end libraries (500 bp) were constructed according to manufacturer protocols (Illumina), and sequenced on the Illumina Hiseq 2500 sequencing platform. From each populations, we sequenced seven samples at 5X depth and one at 10X coverage, except for the MDN population, where we sequenced seven individuals at 5X coverage and one at 20X

coverage and the MDZ population, where we sequenced all individuals at 10X coverage. We generated a total of 628.37 Gb of paired-end reads of 100 bp (or 150 bp; MDZ) length (Supplemental Table S1).

mRNA from brain, liver, and breast muscle of 14 individual ducks were extracted using standard trizol extraction methods. Two paired-end libraries (500 bp) were constructed according to manufacturer instruction (Illumina). All samples were sequenced by Illumina Hiseq 4000 sequencing platform, with 32M paired-end 150 bp mapped reads per sample after QC (Supplemental Table S7).

Read alignment and variant calling

To avoid low quality reads, mainly the result of base-calling duplicates and adapter contamination, we filtered out sequences according to the default parameters of NGS QC Toolkit [50]. Those paired reads which passed Illumina's quality control filter were aligned using BWA-MEM (v0.7.12) to version 1.0 of the *Anas platyrhynchos* genome (BGI_duck_1.0) [1]. Duplicate reads were removed from individual samples alignments using Picard tools MarkDuplicates, and reads were merged using MergeSamFiles (http://broadinstitute.github.io/picard/).

The Genome Analysis Toolkit (GATK, v3.5) RealignerTargetCreator and IndelRealigner protocol were used for global realignment of reads around INDELs before variant calling [51,52]. SNPs and small indels (1-50 bp) were

called used the GATK UnifiedGenotyper set for diploids with the parameter of minimum quality score of 20 for both mapped reads and bases to call variants, similarly to previous studies [53-57]. We filtered variants both per population and per individual using GATK according to the stringent filtering criteria. For SNPs of population filter: a.) QUAL > 30.0; b.) QD > 5.0; c.) FS < 60.0; d.) MQ > 40.0; e.) MQRankSum > -12.5; f.) ReadPosRankSum > -8.0; Additionally, if there were more than 3 SNPs clustered in a 10 bp window, all three SNPs were considered as false positives and removed [58].

We used the following population criteria to identify INDELs: QUAL > 30.0, QD > 5.0, FS < 200.0, ReadPosRankSum > -20.0. Of individual filter, we also removed all INDELs and SNPs where the depth of derived variants was less than half the depth of the sequence. All SNPs and INDELs were assigned to specific genomic regions and genes using SnpEff [59] based on the Ensembl duck annotations. After filtering a total of 36,107,949 SNPs and 3,082,731 INDELs were identified (Supplemental Table S2).

SNP validation

In order to evaluate the reliability of our data, we compared our SNPs to the duck dbSNP database deposited in the Genome Variation Map (GVM) at the Big Data Center in the Beijing Institute of Genomics, Chinese Academy of Science (http://bigd.big.ac.cn/gvm/). 7,908,722 SNPs were validated in the duck dbSNP database, which covered 96.2% of the database (Supplemental

Table S2). For the 28,199,227 SNPs not confirmed by dbSNPs, 390 nucleotide sites were further validated by PCR with 100% accuracy, indicating the high reliability of the called SNP variation identified in this study.

Population structure

We removed all SNPs with a minor allele frequency (MAF) <= 0.1 and kept only SNPs that occurred in more than 90% of individuals. Vcf files were converted to hapmap format with custom perl scripts, and to PLINK format file by GLU v1.0b3 (https://code.google.com/archive/p/glu-genetics/) and PLINK v1.90 [60,61] when appropriate. We used GCTA [62] for Principle Component Analysis (PCA), first by generating the genetic relationship matrix (GRM) followed by the first 20 eigenvectors.

We used all high quality SNPs to infer population structure using FRAPPE 1.1 [7], with 10,000 iterations per run.

A distance matrix was generated by calculating the pairwise allele sharing distance for each pair of all high quality SNPs. Multiple alignment of the sequences was performed with MUSCLE [63]. A neighbor-joining maximum likelihood phylogenetic tree was constructed with the DNAML program in the PHYLIP package v3.69 [64] and MEGA7 [65,66]. All implementation was performed according to the recommended manipulations of SNPhylo [67].

Demographic history reconstruction

The demographic history of both wild and domesticated ducks was inferred using a hidden Markov model approach as implemented in Pairwise Sequentially Markovian Coalescence based on SNP distributions [8]. In order to determine which PSMC settings were most appropriate for each population, we reset the number of free atomic time intervals (-p option), upper limit of time to most recent common ancestor (TMRCA) (-t option), and initial value of $r = \theta/\rho$ (-r option) according to previous research [12] and online suggestions by Li and Durbin (https://github.com/lh3/psmc). Based on estimated from the zebra finch genome, an average mutation rate (μ) of 2.95×10^{-9} per base per generation and a generation time (g) of 1 year were used for analysis [68,69].

Three-population demographic inference was performed using a diffusion-based approach as implemented in the program *∂a∂i* [70]. To minimize potential effects of selection that could interfere with demographic inference, these analyses were performed using the subset of noncoding regions across the whole genome and spanning 750,939,264 bp in length. Noncoding SNPs were then thinned to 1% to alleviate potential linkage between the markers. The final dataset consisted of 95,181 SNPs with an average distance of 7,112 bp (± 18,810 bp) between neighbouring SNPs. To account for missing data, the folded allele frequency spectrum for the three populations (wild, meat and egg/dual purpose breeds) was projected down in *∂a∂i* to the projection that maximized the number of segregating SNPs, resulting in 92,966 SNPs.

We tested four different scenarios to reconstruct the demographic history of the domesticated breeds of mallards: simultaneous domestication of the meat and egg and dual purpose breeds (Model 1); a single domestication event followed by divergence of the meat and egg and dual purpose breeds (Model 2); two independent domestication events, with the meat type breed being domesticated first (Model 3); and two independent domestication events, with the egg and dual purpose breed being domesticated first (Model 4). Using the “backbone” of the best model, we then used a step-wise strategy to add parameters related with variation in population sizes and population growth, keeping a new parameter only if the Akaike information criterion (AIC) and log likelihood improved considerably over the previous model with less parameters. In cases where additional parameters resulted in negligibly improved AIC and likelihood, we retained the simpler, less parameterized model. Gene flow was modelled as continuous migration events after population divergence. Each model was run at least ten times from independent starting values to ensure convergence to the same parameter estimates. We rejected models where we failed to obtain convergence across the replicate runs. Scaled parameters for the best-supported model were transformed into real values using the same average mutation rate (μ) and (g) as described above for the PSMC analysis. Parameter uncertainty was obtained using the Godambe Information Matrix (GIM) [71] from 100 non-parametric bootstraps.

Selective-sweep analysis

In order to define candidate regions having undergone directional selection during duck domestication we calculated the coefficient of nucleotide differentiation (F_{ST}) between mallards and domesticated ducks described by Weir & Cockerham [72]. We calculated the average F_{ST} in 10kb windows with a 5 kb shift for all seven domesticated duck populations combined, and two mallard populations combined. Only scaffolds longer than 10 kb, 2368 of 78488 scaffolds, were chosen for the analysis. We transformed observed F_{ST} values to Z transformation ($Z(F_{ST})$) with $\mu = 0.1154$ and $\sigma = 0.0678$ according to previously described methods [73].

To estimate levels of nucleotide diversity (π) across all sampled populations we used the VCFtools software [74] to calculate $\theta\pi(\mathrm{wild}/\mathrm{domesticated})$ [75], computing the average difference per locus over each pair of accessions. As the measurement of F_{ST}, averaged π ratio ($\theta\pi(\mathrm{wild}/\mathrm{domesticated})$) was calculated for each scaffold in 10kb sliding windows.

Functional classification of GO categories was performed in Database for Annotation, Visualization and Integrated Discovery (DAVID, ver 6.8) [76]. Statistical significance was accessed by using a modified Fisher's exact test and Benjamini correction for multiple testing.

RNA-seq and data processing

High-quality reads were mapped to reference genome using STAR

(v.2.5.3a) [77]. The *featureCounts* function of the *Rsubread* (v.1.5.2) [78,79] was used to output the counts of reads aligning to each gene. We detected the differential expression genes with edgeR [80-83] using a $p_{adj} < 0.05$ threshold.

Data Access

All duck sequence data had been submitted to Genome Sequence Archive (GSA) database of BIG Data Center in Beijing Institute of Genomics (BIGD) with accession number of CRA000523.

Acknowledgments

This work was supported by the earmarked fund for the Beijing Innovation Team of the Modern Agro-industry Technology Research System (BAIC04-2017), European Research Council (grant agreement 680951) and Wolfson Merit Award. We gratefully acknowledge our colleagues in the Poultry Team at the National Engineering Laboratory for Animal Breeding of China Agricultural University, for their assistance on sample collection and helpful comments on the manuscript.

Author contributions

Conceived and designed the experiments: Lujiang Qu. Wrote the paper: Zebin Zhang. Revised the paper: Lujiang Qu, Judith E Mank, Marcel van Tuinen. Analyzed the data: Zebin Zhang, Pedro Almeida, Qiong Wang, Yaxiong Jia. Performed the experiments: Zebin Zhang, Yaxiong Jia. Contributed

reagents/materials: Zhihua Jiang, Yu Chen, Kai Zhan, Shuisheng Hou, Zhengkui Zhou, Huifang Li, Fangxi Yang, and Yong He.

References

1. Huang Y, Li Y, Burt DW, Chen H, Zhang Y, et al. (2013) The duck genome and transcriptome provide insight into an avian influenza virus reservoir species. Nat Genet 45: 776-783.
2. Kiple KF (2000) The Cambridge world history of food. 2: Cambridge University Press.
3. Miller DB (1977) Social displays of Mallard Ducks (Anas platyrhynchos): effects of domestication. Journal of Comparative and Physiological Psychology 91: 221.
4. Ebinger P (1995) Domestication and plasticity of brain organization in mallards (Anas platyrhynchos). Brain, behavior and evolution 45: 286-300.
5. Frahm H, Rehkämper G, Werner C (2001) Brain alterations in crested versus non-crested breeds of domestic ducks (Anas platyrhynchos fd). Poultry science 80: 1249-1257.
6. Duggan BM, Hocking PM, Schwarz T, Clements DN (2015) Differences in hindlimb morphology of ducks and chickens: effects of domestication and selection. Genetics Selection Evolution 47: 88.
7. Tang H, Peng J, Wang P, Risch NJ (2005) Estimation of individual admixture: analytical and study design considerations. Genet Epidemiol 28: 289-301.
8. Li H, Durbin R (2011) Inference of human population history from individual whole-genome sequences. Nature 475: 493-496.
9. Ehlers J, Gibbard PL (2007) The extent and chronology of Cenozoic global glaciation. Quaternary International 164: 6-20.
10. Yang J, Li WR, Lv FH, He SG, Tian SL, et al. (2016) Whole-Genome Sequencing of Native Sheep Provides Insights into Rapid Adaptations to Extreme Environments. Mol Biol Evol 33: 2576-2592.
11. Williams MAJ, Dunkerley D, De Deckker P, Kershaw AP, Stokes T (1997) Quaternary environments: Science Press.
12. Nadachowska-Brzyska K, Li C, Smeds L, Zhang G, Ellegren H (2015) Temporal Dynamics of Avian Populations during Pleistocene Revealed by Whole-Genome Sequences. Curr Biol 25: 1375-1380.
13. Shapiro MD, Kronenberg Z, Li C, Domyan ET, Pan H, et al. (2013) Genomic diversity and evolution of the head crest in the rock pigeon. Science 339: 1063-1067.
14. Price TD (2002) Domesticated birds as a model for the genetics of speciation by sexual selection. Genetica 116: 311-327.

15. Li J, Zhang Y (2009) Advances in research of the origin and domestication of domestic animals. Biodiversity Science 17: 319-329.

16. Li M, Tian S, Jin L, Zhou G, Li Y, et al. (2013) Genomic analyses identify distinct patterns of selection in domesticated pigs and Tibetan wild boars. Nat Genet 45: 1431-1438.

17. Jiang Y, Xie M, Chen W, Talbot R, Maddox JF, et al. (2014) The sheep genome illuminates biology of the rumen and lipid metabolism. Science 344: 1168-1173.

18. Carneiro M, Rubin CJ, Di Palma F, Albert FW, Alfoldi J, et al. (2014) Rabbit genome analysis reveals a polygenic basis for phenotypic change during domestication. Science 345: 1074-1079.

19. Wang MS, Zhang RW, Su LY, Li Y, Peng MS, et al. (2016) Positive selection rather than relaxation of functional constraint drives the evolution of vision during chicken domestication. Cell Res 26: 556-573.

20. Rubin CJ, Zody MC, Eriksson J, Meadows JR, Sherwood E, et al. (2010) Whole-genome resequencing reveals loci under selection during chicken domestication. Nature 464: 587-591.

21. Lorenzen ED, Nogués-Bravo D, Orlando L, Weinstock J, Binladen J, et al. (2011) Species-specific responses of Late Quaternary megafauna to climate and humans. Nature 479: 359-364.

22. Hewitt G (2000) The genetic legacy of the Quaternary ice ages. Nature 405: 907-913.

23. Hewitt G (2004) Genetic consequences of climatic oscillations in the Quaternary. Philosophical Transactions of the Royal Society of London B: Biological Sciences 359: 183-195.

24. Qiu Q, Wang L, Wang K, Yang Y, Ma T, et al. (2015) Yak whole-genome resequencing reveals domestication signatures and prehistoric population expansions. Nat Commun 6: 10283.

25. Orlando L, Ginolhac A, Zhang G, Froese D, Albrechtsen A, et al. (2013) Recalibrating Equus evolution using the genome sequence of an early Middle Pleistocene horse. Nature 499: 74-78.

26. Steingrimsson E, Copeland NG, Jenkins NA (2004) Melanocytes and the microphthalmia transcription factor network. Annu Rev Genet 38: 365-411.

27. Hallsson JH, Haflidadottir BS, Schepsky A, Arnheiter H, Steingrimsson E (2007) Evolutionary sequence comparison of the Mitf gene reveals novel conserved domains. Pigment Cell Res 20: 185-200.

28. Levy C, Khaled M, Fisher DE (2006) MITF: master regulator of melanocyte development and melanoma oncogene. Trends Mol Med 12: 406-414.

29. Minvielle F, Bed'hom B, Coville JL, Ito S, Inoue-Murayama M, et al. (2010) The "silver" Japanese quail and the MITF gene: causal mutation, associated traits and homology with the "blue" chicken

plumage. BMC Genet 11: 15.

30. Karlsson EK, Baranowska I, Wade CM, Salmon Hillbertz NH, Zody MC, et al. (2007) Efficient mapping of mendelian traits in dogs through genome-wide association. Nat Genet 39: 1321-1328.

31. Eriksson M, Nilsson A, Samuelsson H, Samuelsson EB, Mo L, et al. (2007) On the role of NR3A in human NMDA receptors. Physiol Behav 92: 54-59.

32. Bauer PH, Muller S, Puzicha M, Pippig S, Obermaier B, et al. (1992) Phosducin Is a Protein Kinase-a-Regulated G-Protein Regulator. Nature 358: 73-76.

33. Sunayashiki-Kusuzaki K, Kikuchi T, Wawrousek EF, Shinohara T (1997) Arrestin and phosducin are expressed in a small number of brain cells. Brain Res Mol Brain Res 52: 112-120.

34. Mignon-Grasteau S, Boissy A, Bouix J, Faure J-M, Fisher AD, et al. (2005) Genetics of adaptation and domestication in livestock. Livestock Production Science 93: 3-14.

35. Dugatkin LA, Trut L (2017) How to Tame a Fox (and Build a Dog): Visionary Scientists and a Siberian Tale of Jump-Started Evolution: University of Chicago Press.

36. Axelsson E, Ratnakumar A, Arendt ML, Maqbool K, Webster MT, et al. (2013) The genomic signature of dog domestication reveals adaptation to a starch-rich diet. Nature 495: 360-364.

37. Volinia S, Dhand R, Vanhaesebroeck B, MacDougall L, Stein R, et al. (1995) A human phosphatidylinositol 3-kinase complex related to the yeast Vps34p-Vps15p protein sorting system. The EMBO Journal 14: 3339.

38. Inaguma Y, Ito H, Iwamoto I, Matsumoto A, Yamagata T, et al. (2016) Morphological characterization of Class III phosphoinositide 3-kinase during mouse brain development. Med Mol Morphol 49: 28-33.

39. Stopkova P, Saito T, Papolos DF, Vevera J, Paclt I, et al. (2004) Identification of PIK3C3 promoter variant associated with bipolar disorder and schizophrenia. Biol Psychiatry 55: 981-988.

40. Tang R, Zhao X, Fang C, Tang W, Huang K, et al. (2008) Investigation of variants in the promoter region of PIK3C3 in schizophrenia. Neurosci Lett 437: 42-44.

41. Zhou X, Wang L, Hasegawa H, Amin P, Han B-X, et al. (2010) Deletion of PIK3C3/Vps34 in sensory neurons causes rapid neurodegeneration by disrupting the endosomal but not the autophagic pathway. Proceedings of the National Academy of Sciences 107: 9424-9429.

42. Wilson PA, Gardner SD, Lambie NM, Commans SA, Crowther DJ (2006) Characterization of the human patatin-like phospholipase family. Journal of lipid research 47: 1940-1949.

43. Kienesberger PC, Oberer M, Lass A, Zechner R (2009)

Mammalian patatin domain containing proteins: a family with diverse lipolytic activities involved in multiple biological functions. Journal of lipid research 50: S63-S68.

44. Tesson C, Nawara M, Salih MA, Rossignol R, Zaki MS, et al. (2012) Alteration of fatty-acid-metabolizing enzymes affects mitochondrial form and function in hereditary spastic paraplegia. The American Journal of Human Genetics 91: 1051-1064.

45. Schuurs-Hoeijmakers JH, Oh EC, Vissers LE, Swinkels ME, Gilissen C, et al. (2012) Recurrent de novo mutations in PACS1 cause defective cranial-neural-crest migration and define a recognizable intellectual-disability syndrome. The American Journal of Human Genetics 91: 1122-1127.

46. Martin E, Schüle R, Smets K, Rastetter A, Boukhris A, et al. (2013) Loss of function of glucocerebrosidase GBA2 is responsible for motor neuron defects in hereditary spastic paraplegia. The American Journal of Human Genetics 92: 238-244.

47. Gagliano SA, Tiwari AK, Freeman N, Lieberman JA, Meltzer HY, et al. (2014) Protein kinase cAMP-dependent regulatory type II beta (PRKAR2B) gene variants in antipsychotic-induced weight gain. Hum Psychopharmacol 29: 330-335.

48. Czyzyk TA, Sikorski MA, Yang L, McKnight GS (2008) Disruption of the RIIβ subunit of PKA reverses the obesity syndrome of agouti lethal yellow mice. Proceedings of the National Academy of Sciences 105: 276-281.

49. Resources CNCoAG (2010) Animal genetic resources in China poultry. Beijing: China Agriculture Press.

50. Patel RK, Jain M (2012) NGS QC Toolkit: a toolkit for quality control of next generation sequencing data. PLoS One 7: e30619.

51. McKenna A, Hanna M, Banks E, Sivachenko A, Cibulskis K, et al. (2010) The Genome Analysis Toolkit: a MapReduce framework for analyzing next-generation DNA sequencing data. Genome Res 20: 1297-1303.

52. DePristo MA, Banks E, Poplin R, Garimella KV, Maguire JR, et al. (2011) A framework for variation discovery and genotyping using next-generation DNA sequencing data. Nat Genet 43: 491-498.

53. Yan Y, Yi G, Sun C, Qu L, Yang N (2014) Genome-wide characterization of insertion and deletion variation in chicken using next generation sequencing. PLoS One 9: e104652.

54. Qu Y, Tian S, Han N, Zhao H, Gao B, et al. (2015) Genetic responses to seasonal variation in altitudinal stress: whole-genome resequencing of great tit in eastern Himalayas. Sci Rep 5: 14256.

55. Meyer RS, Choi JY, Sanches M, Plessis A, Flowers JM, et al. (2016) Domestication history and geographical adaptation inferred from a SNP map of African rice. Nat Genet 48: 1083-1088.

56. Russell J, Mascher M, Dawson IK, Kyriakidis S, Calixto C, et al. (2016) Exome sequencing of geographically diverse barley landraces and wild relatives gives insights into environmental adaptation. Nat Genet 48: 1024-1030.

57. Mascher M, Schuenemann VJ, Davidovich U, Marom N, Himmelbach A, et al. (2016) Genomic analysis of 6,000-year-old cultivated grain illuminates the domestication history of barley. Nat Genet 48: 1089-1093.

58. Li H, Ruan J, Durbin R (2008) Mapping short DNA sequencing reads and calling variants using mapping quality scores. Genome Res 18: 1851-1858.

59. Cingolani P, Platts A, Wang LL, Coon M, Nguyen T, et al. (2012) A program for annotating and predicting the effects of single nucleotide polymorphisms, SnpEff: SNPs in the genome of Drosophila melanogaster strain w1118; iso-2; iso-3. Fly 6: 80-92.

60. Purcell S, Neale B, Todd-Brown K, Thomas L, Ferreira MA, et al. (2007) PLINK: a tool set for whole-genome association and population-based linkage analyses. The American Journal of Human Genetics 81: 559-575.

61. Chang CC, Chow CC, Tellier LC, Vattikuti S, Purcell SM, et al. (2015) Second-generation PLINK: rising to the challenge of larger and richer datasets. Gigascience 4: 7.

62. Yang J, Lee SH, Goddard ME, Visscher PM (2011) GCTA: a tool for genome-wide complex trait analysis. The American Journal of Human Genetics 88: 76-82.

63. Edgar RC (2004) MUSCLE: multiple sequence alignment with high accuracy and high throughput. Nucleic acids research 32: 1792-1797.

64. Plotree D, Plotgram D (1989) PHYLIP-phylogeny inference package (version 3.2). cladistics 5: 6.

65. Tamura K, Dudley J, Nei M, Kumar S (2007) MEGA4: molecular evolutionary genetics analysis (MEGA) software version 4.0. Molecular biology and evolution 24: 1596-1599.

66. Kumar S, Stecher G, Tamura K (2016) MEGA7: Molecular Evolutionary Genetics Analysis Version 7.0 for Bigger Datasets. Mol Biol Evol 33: 1870-1874.

67. Lee TH, Guo H, Wang X, Kim C, Paterson AH (2014) SNPhylo: a pipeline to construct a phylogenetic tree from huge SNP data. BMC Genomics 15: 162.

68. Zhan X, Pan S, Wang J, Dixon A, He J, et al. (2013) Peregrine and saker falcon genome sequences provide insights into evolution of a predatory lifestyle. Nat Genet 45: 563-566.

69. Balakrishnan CN, Edwards SV (2009) Nucleotide variation, linkage disequilibrium and founder-facilitated speciation in wild

populations of the zebra finch (Taeniopygia guttata). Genetics 181: 645-660.

70. Gutenkunst RN, Hernandez RD, Williamson SH, Bustamante CD (2009) Inferring the joint demographic history of multiple populations from multidimensional SNP frequency data. PLoS Genet 5: e1000695.

71. Coffman AJ, Hsieh PH, Gravel S, Gutenkunst RN (2016) Computationally Efficient Composite Likelihood Statistics for Demographic Inference. Molecular Biology and Evolution 33: 591-593.

72. Weir BS, Cockerham CC (1984) Estimating F-Statistics for the Analysis of Population-Structure. Evolution 38: 1358-1370.

73. Kreyszig E (2007) Advanced engineering mathematics: John Wiley & Sons.

74. Danecek P, Auton A, Abecasis G, Albers CA, Banks E, et al. (2011) The variant call format and VCFtools. Bioinformatics 27: 2156-2158.

75. Tajima F (1983) Evolutionary relationship of DNA sequences in finite populations. Genetics 105: 437-460.

76. Huang da W, Sherman BT, Lempicki RA (2009) Systematic and integrative analysis of large gene lists using DAVID bioinformatics resources. Nat Protoc 4: 44-57.

77. Dobin A, Davis CA, Schlesinger F, Drenkow J, Zaleski C, et al. (2013) STAR: ultrafast universal RNA-seq aligner. Bioinformatics 29: 15-21.

78. Liao Y, Smyth GK, Shi W (2013) The Subread aligner: fast, accurate and scalable read mapping by seed-and-vote. Nucleic acids research 41: e108-e108.

79. Liao Y, Smyth GK, Shi W (2014) featureCounts: an efficient general purpose program for assigning sequence reads to genomic features. Bioinformatics 30: 923-930.

80. Robinson MD, Smyth GK (2007) Moderated statistical tests for assessing differences in tag abundance. Bioinformatics 23: 2881-2887.

81. Robinson MD, McCarthy DJ, Smyth GK (2010) edgeR: a Bioconductor package for differential expression analysis of digital gene expression data. Bioinformatics 26: 139-140.

82. McCarthy DJ, Chen Y, Smyth GK (2012) Differential expression analysis of multifactor RNA-Seq experiments with respect to biological variation. Nucleic Acids Res 40: 4288-4297.

83. Lun AT, Chen Y, Smyth GK (2016) It's DE-licious: a recipe for differential expression analyses of RNA-seq experiments using quasi-likelihood methods in edgeR. Statistical Genomics: Methods and Protocols: 391-416.

Figure 1 Click here to download Figure Fig. 1.pdf

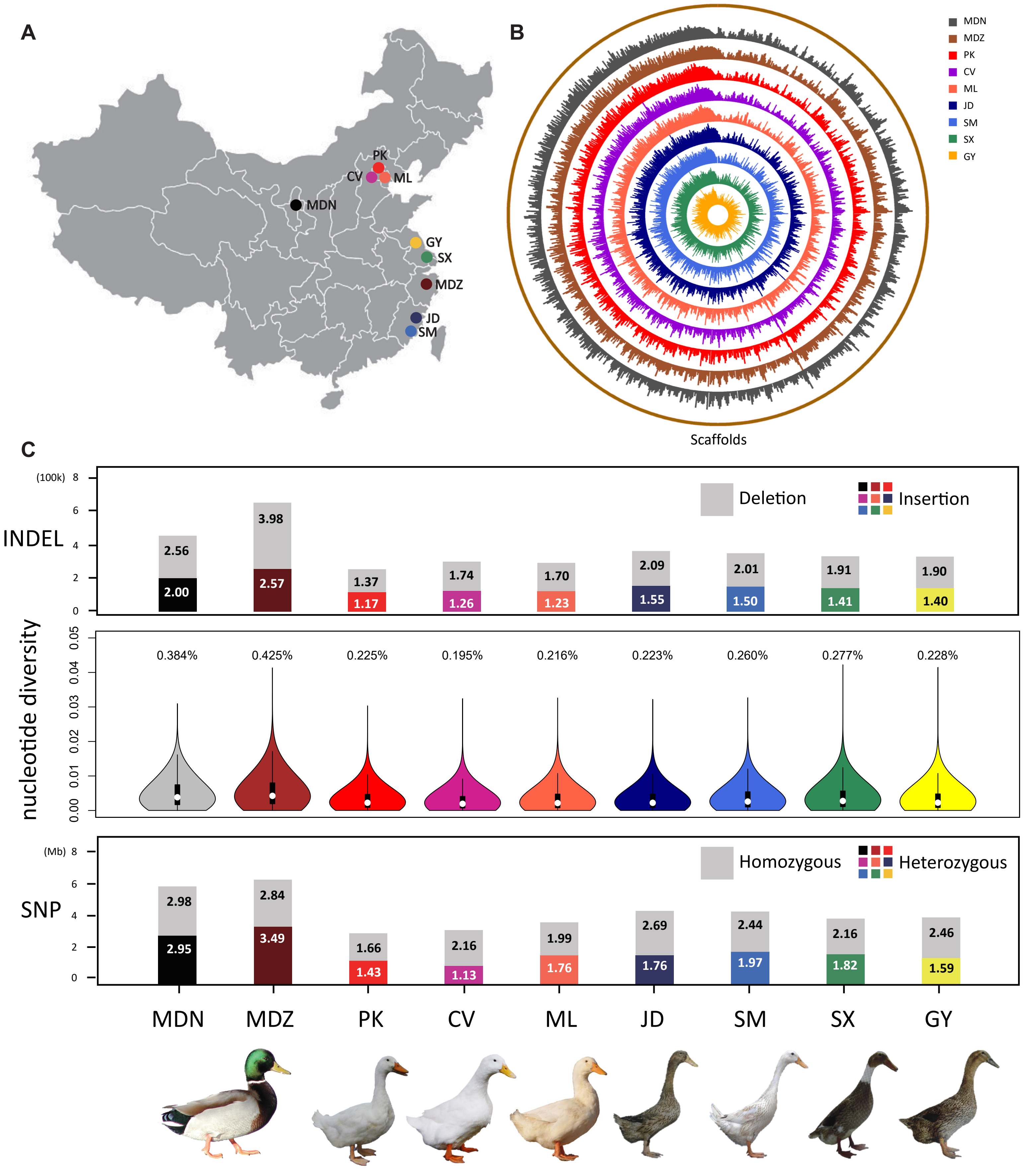

Figure 2

Click here to download Figure Fig. 2.pdf

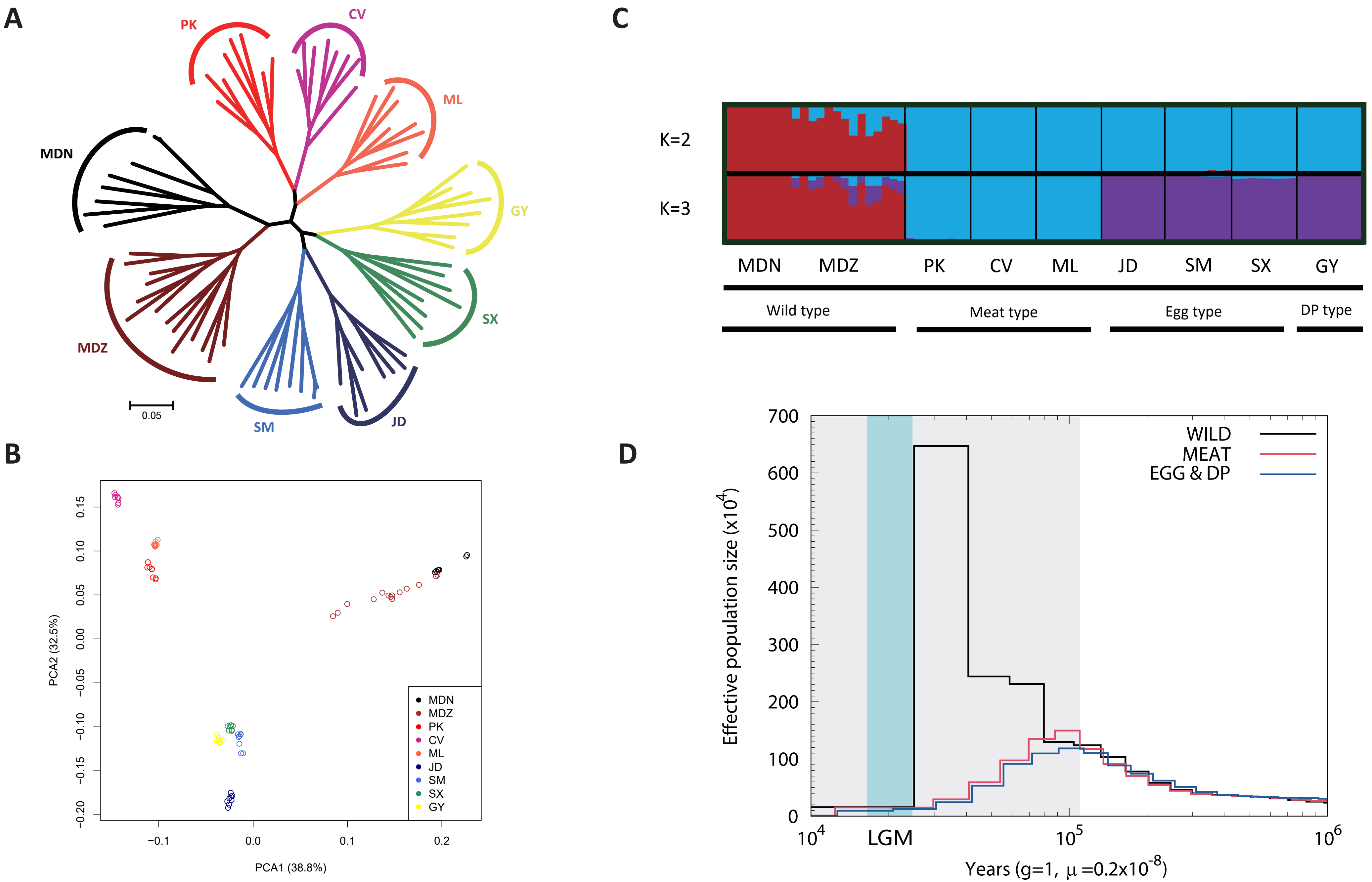

Figure 3 Click here to download Figure Fig. 3.pdf

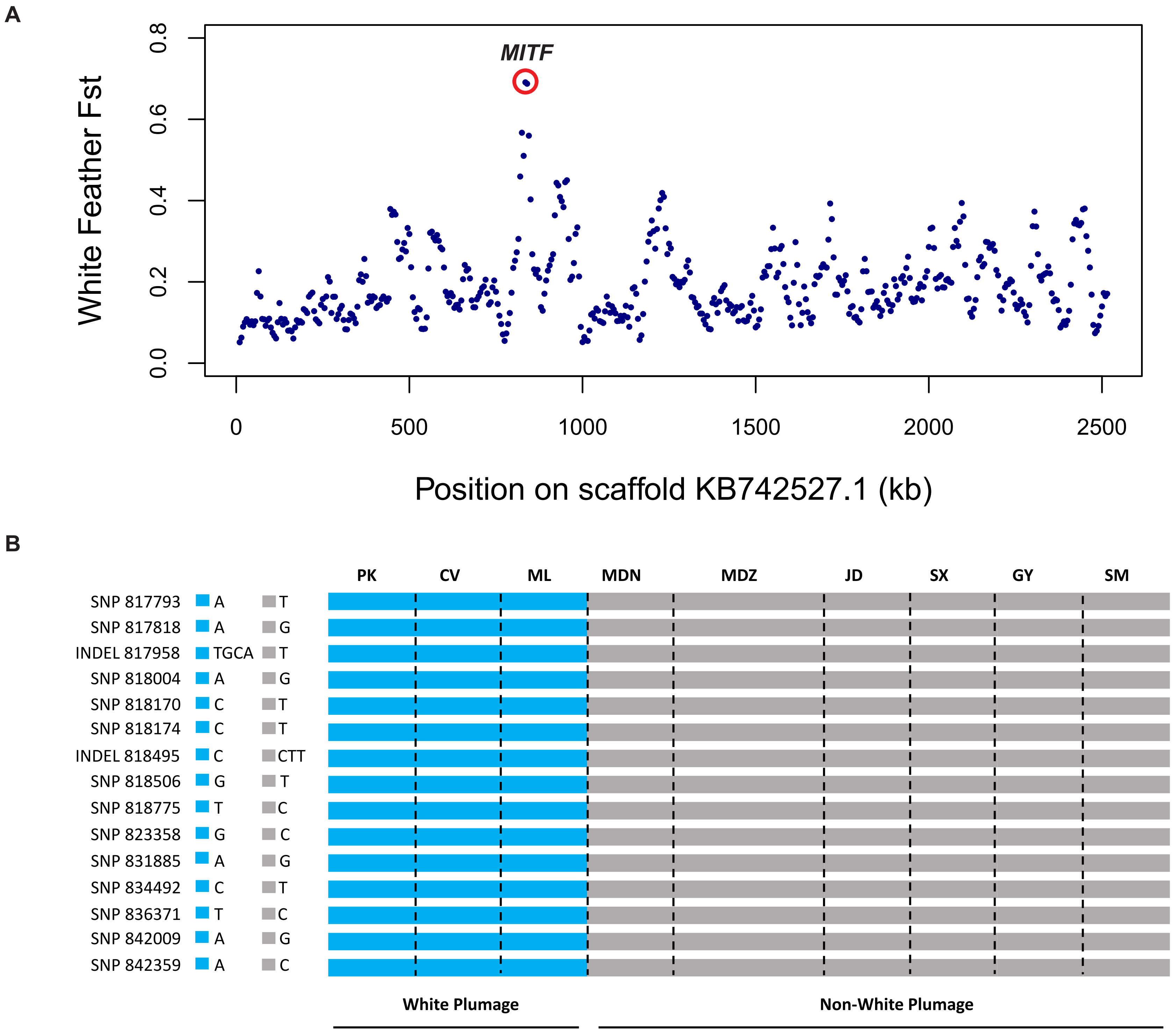

Figure 4

Click here to download Figure fig. 4.pdf

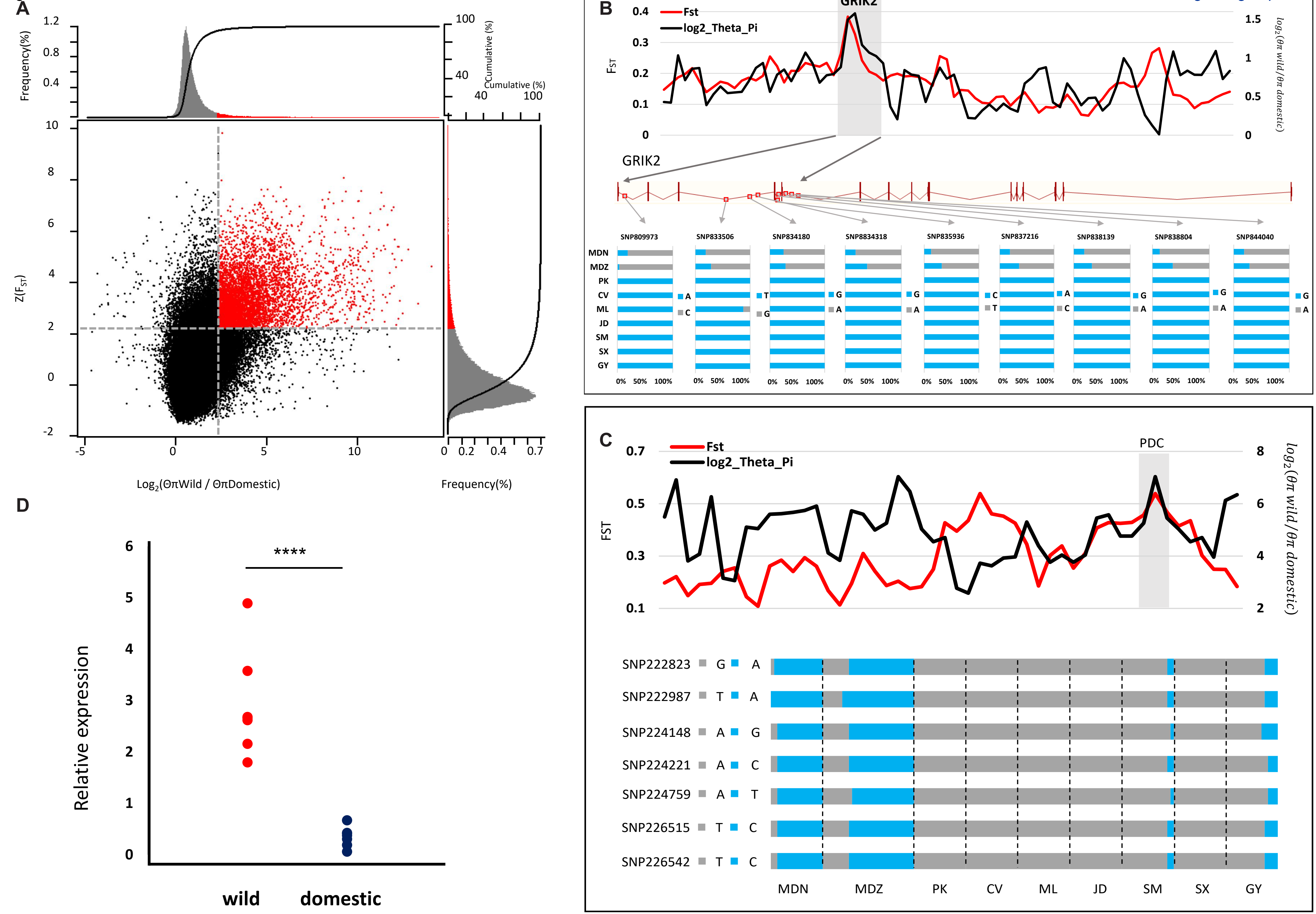

Supplementary Figure S1

Click here to access/download
Supplementary Material
supplemental Figure S1.pdf

Supplementary Figure S2

Click here to access/download
Supplementary Material
supplemental Figure S2.pdf

Supplementary Figure S3

Click here to access/download
Supplementary Material
Supplemental Figure S3.pdf

Supplementary Figure S4

Click here to access/download
Supplementary Material
Supplemental Figure S4.pdf

Supplementary Tables

Click here to access/download
Supplementary Material
Supplementary Tables.xlsx
