# Supplementary material for: Whole-genome resequencing reveals signatures of selection and timing of duck domestication
Source: Gigascience. 2018 Apr 9;7(4):giy027. doi: 10.1093/gigascience/giy027 (PMC6007426; doi:10.1093/gigascience/giy027)
Supplement: GIGA-D-17-00301_Revision_1.pdf [file giy027_giga-d-17-00301_revision_1.pdf]

GigaScience

Whole-genome resequencing reveals signatures of selection and timing of duck domestication

--Manuscript Draft--

**Manuscript Number:**	GIGA-D-17-00301R1
**Full Title:**	Whole-genome resequencing reveals signatures of selection and timing of duck domestication
**Article Type:**	Research
**Funding Information:**	Beijing Innovation Team of the Modern Agro-industry Technology Research System (BAIC04-2017) — Prof. Lujiang Qu<br>European Research Council (680951) — Dr. Judith E Mank<br>Wolfson Merit Award — Dr. Judith E Mank
**Abstract:**	Background: The genetic basis of animal domestication remains poorly understood, and systems with substantial phenotypic differences between wild and domestic populations are useful for elucidating the genetic basis of adaptation to new environments as well as the genetic basis of rapid phenotypic change. Here, we sequenced the whole genome of 78 individual ducks, from two wild and seven domesticated populations, with an average sequencing depth of 6.42X per individual.<br>Results: Our population and demographic analyses indicate a complex history of domestication, with early selection for separate meat and egg lineages. Genomic comparison of wild to domesticated populations suggest that genes affecting brain and neuronal development have undergone strong positive selection during domestication. Our FST analysis also indicates that the duck white plumage is the result of selection at the melanogenesis associated transcription factor locus.<br>Conclusions: Our results advance the understanding of animal domestication and selection for complex phenotypic traits.
**Corresponding Author:**	Lujiang Qu, Ph.D.<br>China Agricultural University<br>Beijing, CHINA
**Corresponding Author Secondary Information:**	
**Corresponding Author's Institution:**	China Agricultural University
**Corresponding Author's Secondary Institution:**	
**First Author:**	Zebin Zhang
**First Author Secondary Information:**	
**Order of Authors:**	Zebin Zhang
	Yaxiong Jia
	Pedro Almeida
	Judith E Mank
	Marcel van Tuinen
	Qiong Wang
	Zhihua Jiang
	Yu Chen
	Kai Zhan
	Shuisheng Hou

Powered by Editorial Manager® and ProduXion Manager® from Aries Systems Corporation

	Zhengkui Zhou
	Huifang Li
	Fangxi Yang
	Yong He
	Zhonghua Ning
	Ning Yang
	Lujiang Qu, Ph.D.
**Order of Authors Secondary Information:**	

Response to Reviewers:

Dear Dr Zauner,

Many thanks for your positive comments about our manuscript, “Whole-genome resequencing reveals signatures of selection and timing of duck domestication” (manuscript number GIGA-D-17-00301). We also thank the reviewers for their thoughtful and constructive suggestions. We have addressed all these comments, detailed below, in our revised manuscript, which we hope is now suitable for publication in GigaScience.

Sincerely,
Lujiang Qu, Ph.D., on behalf of all co-authors.
Email: quluj@163.com
Department of Animal Genetics and Breeding, College of Animal Science and Technology, China Agricultural University, Beijing 100193, China

GIGA-D-17-00301
Whole-genome resequencing reveals signatures of selection and timing of duck domestication
Zebin Zhang; Yaxiong Jia; Pedro Almeida; Judith E Mank; Marcel van Tuinen; Qiong Wang; Zhihua Jiang; Yu Chen; Kai Zhan; Shuisheng Hou; Zhengkui Zhou; Huifang Li; Fangxi Yang; Yong He; Lujiang Qu, Ph.D.
GigaScience

Dear Prof. Qu,

Your manuscript "Whole-genome resequencing reveals signatures of selection and timing of duck domestication" (GIGA-D-17-00301) has been assessed by our reviewers. Although it is of interest, we are unable to consider it for publication in its current form. The reviewers have raised a number of points which we believe would improve the manuscript and may allow a revised version to be published in GigaScience.

Their reports are below.

Comment: All reviewers, but reviewer 2 in particular, provide some suggestions how the submission can be improved, for example by explaining the hypotheses more clearly in the introduction, and also by some additional analyses that may make the paper even stronger.

Reply: Many thanks for your comments. We have more clearly articulated our hypotheses in introduction section according to your and reviewer2’s suggestion, please see lines 75-79. Meanwhile, we have done the additional analyses according to your and reviewer2’s suggestion, such as FRAPPE analyses by K=4, PSMC and δaδi analyses based on chicken mutation rate, global FST between each duck population, and FST recalculated by BayeScan, please see the specific reply to reviewer2.

Comment: An absolutely crucial point for publication in GigaScience is the remark #6 by reviewer 1, regarding sharing of data, code and protocols. GigaScience embraces the FAIR principles (https://www.force11.org/group/fairgroup/fairprinciples) and we ask our authors to document their work according to these principles, to allow full reproducibility and maximum reuse potential of the data, protocols and scripts.

Please include supporting data such as custom scripts, full population genetic statistics and location of sweeps, any software output files, alignments, phylogenetic tree files etc.

Reply: Thank you for this suggestion. The 78 ducks used in our whole genome resequencing analysis and the 14 ducks used in RNA-seq analysis have been submitted to NCBI BioProject (http://www.ncbi.nlm.nih.gov/bioproject) under accession numbers PRJNA419832 and PRJNA419583, respectively. The unassembled sequencing reads of 78 ducks and RNA-seq reads of 14 ducks have been deposited in NCBI Sequence Read Archive (SRA: http://www.ncbi.nlm.nih.gov/sra) under accession numbers SRP125660 and SRP125529, respectively.

VCF files of SNPs and INDELs, as well as other supporting data, have been submitted to GigaDB as suggested. Please check the GigaDB servers.

Meanwhile, we also replied to reviewer 1 and have added these description to our current manuscript, please see lines 618-628.

To share your supporting data and scripts, our data curators will be able to help you to make them available via our data repository GigaDB. You can contact them via email: database@gigasciencejournal.com.

We are encouraging our submitters to make use of protocols.io , if you provide your methods (both wet-lab and dry-lab) in the SOP tab on the data spreadsheet we can import those into protocols.io on your behalf.

To share your raw sequencing data, please note that the BIG data repository is not part of the International Nucleotide Sequence Database Collaboration. Please choose a database that is an INSDC member (http://www.insdc.org/) and report accession numbers of the INSDC database in the manuscript.

If you are able to fully address points of our reviewers, we would encourage you to submit a revised manuscript to GigaScience. Once you have made the necessary corrections, please submit online at:

http://giga.edmgr.com/

If you have forgotten your username or password please use the "Send Login Details" link to get your login information. For security reasons, your password will be reset.

Please include a point-by-point within the 'Response to Reviewers' box in the submission system.
Please ensure you describe additional experiments that were carried out and include a detailed rebuttal of any criticisms or requested revisions that you disagreed with.
Please also ensure that your revised manuscript conforms to the journal style, which can be found in the Instructions for Authors on the journal homepage.

The due date for submitting the revised version of your article is 20 Mar 2018.

I look forward to receiving your revised manuscript soon.

Best wishes,

Hans Zauner
GigaScience
www.gigasciencejournal.com

Reviewer reports:

Reviewer #1:
This paper reports sequencing, population history inferences, and selective sweep mapping in ducks using whole genome sequence data of multiple populations.

This is a good paper. It presents a large-scale population genomic dataset of ducks, uses standard methods that seem appropriate to the task, and it is well written.

Despite this, I have a few criticisms and questions:

Comment: 1. The paper repeatedly states that this is the first time MITF is associated with colour in the duck. This seems not to be entirely true (see Li et al 2012, http://journals.plos.org/plosone/article?id=10.1371/journal.pone.0036592, and Sultana et al 2017, https://www.ncbi.nlm.nih.gov/pubmed/28823136, but maybe the latter was not published when the manuscript was written). This study presents a whole-genome scan, which should provide stronger evidence than candidate gene associations. Comparing to other papers would be interesting. Can that help filter the candidate variants?

Reply: Thank you very much for your positive comments and for the two very helpful citations. Li et al (2012) identified that M isoform of MITF as expressed in black feather ducks, rather than white feather ducks or other colorful ducks. Sultana et al (2017) showed several SNPs and INDEL of MITF with different allele frequency in black and white ducks (table 2 - 5), but did not distinguish the correlation of MITF to white or other feather colors.
Due linkage effects, it is notoriously difficult to determine which variant is the real causative mutation of white plumage. Thus, we used the strictest variant filter criteria, namely those with fixed genotype differences in white and non-white ducks. We would very much like to implement the reviewer's suggestion of using the variants identified in these two previous studies, however the variants reported in Li et al (2012) and Sultana et al (2017) do not in fact pass our strict filter criteria.

We have however added these citations to our manuscript and revised the discussion accordingly (please see line 390). Most importantly, in order to distinguish our result from these previous studies, we revised our statement to say that "Our results show that white plumage in the duck is completely associated with selection at the MITF locus" in our current manuscript, please see line 42 and line 246-247.

Comment: 2. It would be useful to see the population history results put more into context. In the light of what is known about duck breed history, is it reasonable that meat and egg type ducks split 2100 years ago? In the Discussion, this number is said to be "compatible with previous written records from 500 BC". The reference is to a book with no page numbers given. Would it be possible to be more specific? Given convergence problems with alternative models, how sure are you that the balance between migration and split time is right? I will admit that I am not really the person to evaluate the pairwise sequential Markov coalescent and δaδi results.

Reply: Many thanks for your comments. As we state in the manuscript, written records note domestic ducks in China as early as 500 BC. Due to the lack of archaeological evidence, we must focus on textual evidence, which indicates duck domestication occurred approximately 2,000 - 2,500 years ago. We have added these historical references regarding duck domestication to our current manuscript, please see lines 63-71, and have added page numbers to the book citations, and below, please see lines 697-700. Meanwhile, we also reran the PSMC and δaδi analyses based on the mutation rate estimate in chicken (1.91 x 10-9 per base per generation, Nam et al. 2010). The chicken is phylogenetically closer to the duck than zebra finch, the source of our previous mutation rate estimate (Jarvis et al. 2014), however the mutation rate estimates in both chicken and duck are qualitatively similar. As a result, our results are similar, and indicate duck domestication occurred 2228 (441) years ago. We revised the PSMC and δaδi results of our current manuscript, please see Fig 2D, Table 1, and lines 204-219, 546-548.

It is true that the recent divergent time and the high level of diversity in both the domestic and wild populations makes it difficult to differentiate recent admixture from incomplete lineage sorting, however our genetic analysis is largely consistent with these written records, and does not indicate domestication much earlier than this time.

Luff R. 2000. Ducks. In Cambridge World History of Food, ed. KF Kiple, KC Ornelas, pp. 517–24. Cambridge, UK: Cambridge University Press

Jarvis, E. D., et al. (2014). "Whole-genome analyses resolve early branches in the tree of life of modern birds." Science 346(6215): 1320-1331.
Nam, K., et al. (2010). "Molecular evolution of genes in avian genomes." Genome Biol 11(6): R68.

Comment: 3. It is nice to see the high overlap between SNPs detected here and those in dbSNP. How many of the indels were already in databases? Was PCR validation only for SNPs? Given that indel detection is harder than SNP detection, are you convinced that the MITF indels are real?

Reply: Thank you for your comments. Initially, we validated our INDELs in dbINDEL, following a similar protocol to our SNP validation. However, there has been less focus on INDEL annotation in the database, which contains nearly 70 fold fewer INDELs than we detected. As we used extremely strict filter criteria for INDELs as well as SNPs, we suggest that the difference in variation is due to our greater focus on INDEL annotation please lines 497 – 500.
For the two MITF INDELs discussed, we used diagnostic PCR combined with Sanger sequencing to validate these sites in the 78 white and non-white ducks, as well as the first three SNPs (SNP817793, SNP817818, and SNP818004). The Sanger sequencing results of the three SNPs and INDEL817958 completely match our NGS analysis, please see figure below and supplemental figure S5 in our current manuscript. For INDEL818495, we were unable to identify a suitable PCR primer. We have added this to our revised manuscript, please see lines 247-253.

Comment: 4. A protocol for PCR validation seems to be missing (L440-442). It is hard to interpret the 100% accuracy in SNP validation when it is not clear how validation was performed or the accuracy evaluated.

Reply: Apologies, and many thanks for pointing this out. The SNP validation was performed by diagnostic PCR combined with Sanger sequencing method. We have added this description to our revised manuscript, please see lines 510-513.

Comment: 5. The paper is well written, but the GigaScience author guidelines prescribe a somewhat different structure. It specifies an abstract divided into Background, Results, and Conclusions. The Data Description section is missing and other sections are have different names.

Reply: Thank you very much for this helpful suggestion. We had separated the abstract section accordingly, please see lines 30-44. We have also added the Data Description section, please see lines 86-109. We also renamed the Results as Analyses, please see line 111, and revised the Availability of Supporting Data and Materials (lines 618-628), and the Declarations section (lines 632, 633, and 641).

Comment:6. It seems to me that the data and source code availability may not be in line with the journal policies. I am not certain how to interpret the policies, but the editors will know better. Overall, the methods are described in text, but protocols and scripts are not provided. The raw sequence data is published in a repository, but little else, not even the full population genetic statistics or location of sweeps, as far as I can tell.

Reply: Apologies for our previous raw data and source code status. The data from the 78 ducks used in whole genome resequencing and the 14 ducks used in RNA-seq analysis have been submitted to NCBI BioProject (http://www.ncbi.nlm.nih.gov/bioproject) under accession numbers PRJNA419832 and PRJNA419583, respectively. The unassessembled sequencing reads of 78 ducks and RNA-seq reads of 14 ducks have been deposited in the NCBI Sequence Read Archive (SRA: http://www.ncbi.nlm.nih.gov/sra) under accession numbers SRP125660 and SRP125529, respectively. VCF files of SNPs and INDELs, as well as other supporting data, have been submitted to GigaDB as you suggest, please check the GigaDB servers. And, we add these description to our current manuscript, please see lines 618-628.

Minor comments

Comment: Line 35: The important numbers are the number of individuals sampled and the coverage per individual. Average coverage per breed seems less interesting.

Reply: Many thanks for your comment, we had revised this to per individual coverage information, please see line 36.

Comment: Lines 97-101: What do the average numbers of variants detected per individual mean? Are they variants that differ from reference genome, heterozygous variants, or something else?

Reply: Many thanks for your questions. The number of variants between the reference genome and each individual are different, especially in wild mallard and domesticated ducks, (please see supplementary table S2). The average value is the mean variant count of an individual, which includes both heterozygous variants and homozygous variants.

Comment: Lines 243-250: Which GO terms were these, and how were they chosen? It seems odd to me to first select a subset of genes based on GO and then perform enrichment analysis on that set. Will this not bias the analysis?

Reply: Apologies for any confusion. In fact, we observed 292 genes in the top 5% Fst regions, please see supplementary table S5. Our enrichment analysis is based on these 292 genes, and we identified a subset of GO terms for further analyses based on significant GO term P-values, please see supplementary table S7. Moreover, we add the full GO terms to our current manuscript, please see supplementary table S6.

Comment: Lines 393-400: Is there a reason for this mix of sequencing coverage?

Reply: We aimed to sequence each individual at 5X coverage. Additionally, in order to reduce the false negative rate of variants due to our strict filter criteria, we randomly selected one individual from each population for 10X coverage.

Comment: Lines 381-384: It is not clear where the ducks came from. How were they obtained?

Reply: Many thanks for your questions. PK and ML ducks were obtained from Institute of Pekin Duck with the help of Mr. Fangxi Yang, please see author information section, lines 5 and 25. CV ducks were obtained from Cherry Valley farms Co. Ltd with the help of Dr. Yong He, please see lines 5 and 26. The other domesticated ducks were obtained from different duck breeding farms under the help of Dr. Huifang Li, please see lines 5 and 23.

Comment: Line 506: What tool was used for Fst? Also VCFtools?

Reply: Thanks you very much for your questions. The Fst was calculated by the formula described by Weir BS (1984) under our custom perl script. Our custom perl script have been submitted to GigaDB database.

Weir, B. S. and C. C. Cockerham (1984). "Estimating F-Statistics for the Analysis of Population-Structure." Evolution 38(6): 1358-1370.

Comment: Figure 1b: The circos plot in Figure 1 looks impressive, but is impossible to read. What is it supposed to show?

Reply: Apologies for any problems with our figures. The complicated circos plot is the result of the many scaffolds (78,488) in the current duck reference genome. We have removed the circos plot from our current manuscript, please see figure 1, and line 125-127.

Comment: Throughout methods: Version numbers are missing for some softwares.

Reply: Apologies for this. We have added all this information to our current manuscript, such as NGS QC Toolkit v2.3.3 (line 480), SnpEff v4.0 (line 501), GCTA v1.25 (line

Powered by Editorial Manager® and ProduXion Manager® from Aries Systems Corporation

520), MUSCLE v3.8 (line 532), PSMC v0.6.5 (line 541), ∂a∂i v1.7 (line 550), VCFtools v0.1.13 (line 592), and edgeR v3.6 (line 617).

Reviewer #2:

Zhang et al. sequenced whole genomes of 78 individuals of domesticated and wild mallard populations. The authors find a complex history of domestication, with particular artificial selection of meat and egg production in domesticated lineages. Further, outlier analyses demonstrate that white plumage was the result of selection of MITF transcriptional factors. I believe that the authors are tackling an important question regarding variation between domesticates and wild populations, and with an extensive genomic dataset. However, I think the authors fall short in introducing the subject and discussing their results. Moreover, the manuscript requires editing prior to publication, particularly the introduction.

Comment: Introduction.
The introduction requires extensive editing. I would also encourage the authors to add another sentence as the relevance (the why) of looking for outliers between domesticated and wild stocks. What exactly are you trying to learn? Instead of results, I would like to see hypotheses regarding what the authors may expect when comparing the genomes of domesticated and wild populations.

Reply: Many thanks for your comments. The most important reason we identified outliers between wild and domesticated ducks was to identify putative sites associates with the genetic basis of phenotypic differences between wild and domestic populations. We have added this explanation to our manuscript, and have also extensively revised our introduction section according to your suggestions, please see lines 51-85.

We had two primary hypotheses regarding duck domestication given the deep divergence between meat and egg breeds. Were ducks domesticated once from wild mallards and subsequently selected for separate egg and meat traits, or were egg and meat populations domesticated in two independent events. We have add the hypotheses of duck domestication scenarios to introduction section, please see lines 75-79.

Comment: The whole first paragraph requires editing.
For example -- Line 50-52: Suggest change sentence to: "Mallards (Anas platyrhynchos) are the world's most widely distributed and agriculturally important waterfowl species, and are especially of economic importance in Asia [1]."

Reply: Many thanks for this suggestion. We had revised the sentence accordingly, please see lines 63-64. And we have also extensively revised the first paragraph as suggested, please see lines 52-71.

Comment: Results
1. Line 79 - is this 535 billion mappable reads per sample or across samples?

Reply: Apologies for any confusion. The 535 billion is the total mapped reads across samples. We have added this explanation to our revised manuscript, please see line 117.

Comment: 2. Lines 115-121- how did the authors pick the optimum K in FRAPPE analyses? Did the authors explore additional K values? Where separate analyses done within wild and domesticated populations? Please explain.

Reply: Many thanks for your comments. We analyzed the population structure with K =2, 3 and 4 because there are four duck types across the nine duck populations, shown below, and explained in lines161-165. When K=4, a clear division was found between egg type ducks (JD, SM, and SX) and dual-purpose type ducks (GY) (supplemental figure S6). The most important reason we focused on K=3 as the optimum value for further analysis is due to the results of both the phylogenic and PCA analyses, which convergently showed the nine duck populations clustered into 3 major groups.

Powered by Editorial Manager® and ProduXion Manager® from Aries Systems Corporation

Comment: 2a. What do the authors make of domesticated admixture in wild populations? Is this hybridization, ancestry, a combination of both…? I would encourage the authors to explore this further as hybridization between domesticated and wild breeds is a serious concern for conservation of wild populations.

Reply: We agree with the reviewer that this is a very interesting area, and an area of great conservation importance. Unfortunately, given the recent domestication and high levels of diversity we observe, it is not in fact possible to accurately differentiate hybridization from incomplete lineage sorting with our current data, as complex models with these alternative scenarios failed to converge. We agree that this is an interesting area for further study, and have added this explanation to our current manuscript, please see lines 377-381.

Comment: 2b. The PCA analyses seem to suggest that there is structure within wild populations. Running a FRAPPE analyses on wild populations could help tease out whether they are 1 population and PCA analyses are just separating samples as there is so much variation.

Reply: Thank you very much for your comments. Of course, the PCA result showed there is a structure within wild populations, because the two wild populations come from two different provinces in China separated by nearly 2,000 km, (please see line 446). However, the PCA result also showed extensive overlap of these two wild populations, please see fig 2B. Additionally, our FRAPPE analyses were based on all 78 duck individuals rather than pooled population information. Thus, we apologize if we have missed something intended by the reviewer, but we think the structural analysis suggested with recover the same result as our current analysis.

Comment: 3. Lines 139-141 - consider revising the sentence into a more formal hypothesis. I would also like to see such hypotheses in the introduction.

Reply: Thank you so much for your kind suggestion. We had two primary hypotheses regarding duck domestication given the deep divergence between meat and egg breeds. Were ducks domesticated once from wild mallards and subsequently selected for separate egg and meat traits, or were egg and meat populations domesticated in two independent events. We have added the hypotheses of duck domestication scenarios to introduction section, please see lines 75-79.

Comment: 4. Outside of outlier tests by calculating FST, the authors should consider more formal testing of these putative outliers (e.g., BayeScan).

Reply: Thank you very much for this suggestion. We have recalculated our FST with BayeScan, and the results are statistically similar to our current analysis, based on Weir, B. S. (1984). Thus, we have kept our previous FST method in our revised manuscript, as this method is a classical and formal method for calculating FST, and has been widely implemented in many organisms, including rice (Meyer, R. S., et al. 2016), sheep (Yang, J., et al. 2016), dog (Gou, X., et al. 2014, Axelsson, E., et al. 2013), and pigeon (Shapiro, M. D., et al. 2013).

Weir, B. S. and C. C. Cockerham (1984). "Estimating F-Statistics for the Analysis of Population-Structure." Evolution 38(6): 1358-1370.
Meyer, R. S., et al. (2016). "Domestication history and geographical adaptation inferred from a SNP map of African rice." Nat Genet 48(9): 1083-1088.
Yang, J., et al. (2016). "Whole-Genome Sequencing of Native Sheep Provides Insights into Rapid Adaptations to Extreme Environments." Mol Biol Evol 33(10): 2576-2592.
Gou, X., et al. (2014). "Whole-genome sequencing of six dog breeds from continuous altitudes reveals adaptation to high-altitude hypoxia." Genome Res 24(8): 1308-1315.
Axelsson, E., et al. (2013). "The genomic signature of dog domestication reveals adaptation to a starch-rich diet." Nature 495(7441): 360-364.
Shapiro, M. D., et al. (2013). "Genomic diversity and evolution of the head crest in the rock pigeon." Science 339(6123): 1063-1067.

Powered by Editorial Manager® and ProduXion Manager® from Aries Systems Corporation

Comment: 5. Although I like the idea of RNA-seq data here. I think that this is largely overlooked in the manuscript and may detract from the main (genome) focus. I would encourage the authors to consider taking the RNA-seq out or sufficiently expanding on methods, reasoning, etc. of the RNA-seq data.

Reply: Thank you so much for your suggestion. We respectfully suggest that the RNA-seq is a key component of our manuscript, as it represents functional phenotypic differentiation of wild mallards and domesticated ducks, and helps connect the genomic variation to phenotypic differences. We have revised the methods and reasoning of including this data RNA-seq as suggested, please see lines 324-328, 470-475, and 603-615.

Comment: 6. I would like to see global Fst estimates among breeds, wild locations

Reply: Many thanks for your comment. The global FST between were showed in below, and we also add this table to our current manuscript, please see lines 267-268, and supplemental table S4.

Comment: Discussion
I have no issues with the discussion and find it the best written. I think that a section on domesticate and wild hybridization may broaden the appeal of this paper.

Reply: Thanks for this suggestion. As we mentioned above, given the recent domestication and high levels of diversity we observe, it is not possible to accurately differentiate hybridization from incomplete lineage sorting with our current data, as complex models with these alternative scenarios failed to converge. We agree that this is an interesting area for further study, and have added material to the discussion as suggested, please see lines 377-381.

Comment: Methods
Please add additional information regarding FRAPPE analyses, K selection,etc.

Reply: Apologies for any omissions. We have added the method of FRAPPE analyses and K selection to our current manuscript, please see lines 523-529.

Comment: Figures
Figure 1: Consider re-moving statistical tests as these are presented in the results.

Reply: Thanks for your helpful comment. We have moved the statistical tests to the results section as suggested, please see lines 129-133, 144-147.

Reviewer #3:

Overall a very nice paper, detailed comments to the authors:

Comment: Line 35: 45X coverage is misleading since the individual coverage was much smaller, please make a clearer statement here

Reply: Thank you for this helpful suggestion. We have revised the population coverage information to individual information, please see line 36.

Comment: L40: Our FST analysis also indicates for the first time ...

Reply: Thanks for this suggestion. We have revised our manuscript according to your suggestion, please see lines 41-43.

Comment: L52: of particular economic importance ...

Reply: Many thanks for your comment. Done! Please see line 65.

Comment: L60-72: This is not introduction, but actually another summary, which I think is obsolete, a slightly more extended real introduction discussing backgraound

Powered by Editorial Manager® and ProduXion Manager® from Aries Systems Corporation

prior knowledge, and aims of the study, would be preferred

Reply: Many thanks. We have moved this section of our previous version to Data Description according to GigaScience author guidelines and your suggestions, please lines 91-109. Meanwhile, we have revised our Introduction section, please see lines 52-85.

Comment: Figure 1B: this panel is nice, but not very informative, what exact information is retrieved from the graph?

Reply: Apologies for any problems with our figures. The complicated circos plot is the result of the many scaffolds (78,488) in the current duck reference genome. We have removed the circos plot from our current manuscript, please see Figure 1.

Comment: L95: The number of deletions was higher than the number of insertions in all nine populations

Reply: Done! Please see line 134.

Comment: L105: Move the sentence "Single base-pair INDELs were the predominant form, accounting for 38.63% of all detected INDELs (Supplemental Table S3)." before the sentence "Both the number of SNPs ..."

Reply: Thank you so much for your kind suggestion. We revised our manuscript accordingly, please see lines 142-143.

Comment: L111: ... clustered together, the three ...

Reply: Done! Please see line 155.

Comment: L117: Show figure for K=2?

Reply: Thanks for your question. Both K=2 and K=3 were showed in fig 2C, please see line 166.

Comment: L155: ... had the lowest Akaike Information Criteria (AIC) value, ...

Reply: Done! Please see lines 200-201.

Comment: L166: ... are lower than in wild mallards ...

Reply: Done! Please see line 213.

Comment: Table 1: is it possible to report standard errors or confidence intervals of the reported estimates?

Reply: Many thanks for your question. To answer the reviewer's question we added 95% confidence intervals to all estimates. We reanalyzed the demographic history of duck domestication based on mutation rates of both zebra finch and chicken. Using the mutation rate of zebra finch (Jarvis et al. 2014), the time of duck domestication is estimated at 2,128 (+- 421) years ago. With estimates of mutation rate from chicken (Nam et al. 2010), we estimate domestication 2,228 (+- 441) years ago. Considering the genetic relationship of duck to chicken is much closer than to zebra finch (Jarvis, E. D., et al. 2014), we revised the PSMC and δaδi results of our current manuscript, please see Fig 2D, Table 1, and lines 203-211, 547-549.

Comment: L197: ... white plumage phenotype suggesting a causative mutation. Our result indicates for the first time the duck white plumage associated with selection at ...

Reply: Done! Please see lines 245-247.

Comment: L213: of 10kb size.

Powered by Editorial Manager® and ProduXion Manager® from Aries Systems Corporation

Reply: Done! Please see line 267.

Comment: L224: "... scaffolds longer than 10-kb by 10-kb windows with 5-kb steps." This is not clear to me, please describe better.

Reply: Apologies for any confusion. In our study, both FST and π were calculated for each 10kb size window, with 5kb size steps. However, of the 78,488 scaffolds in the duck reference genome, there are many scaffolds < 10kb. These short scaffolds were removed, and we only calculated FST for scaffolds > 10kb. We have added this to our revised manuscript, please see lines 279-281.

Comment: L237 was shown

Reply: Done! Please see lines 293-294.

Comment: L240 level differs between domesticated and wild duck.

Reply: Done! Please see line 296.

Comment: L245 I understand that you limited the GO analysis to certain processes, what happened if you included other processes as well?

Reply: Many thanks for this suggestion. In this study, all 292 genes located in the 5% FST regions (supplementary table S5) were used for the GO analysis, resulting in a total of 57 GO enrichment terms, which have now all been added to our current manuscript, please see lines 300-301, and supplementary table S6. This high number of GO terms presents a hopelessly difficult and complicated analyses, therefore we selected a subset of GO terms for further analysis based on P-value (supplementary table S7) combined the phenotypic differences between wild mallard and domestic duck. We do agree with the reviewer that a more inclusive analysis would be preferable, but the large number of GO terms makes it impossible to obtain meaningful results.

Comment: L252 identified as being under positive selection

Reply: Corrected! Please see line 311.

Comment: L258 Is "neuronal genes" the right term?

Reply: Apologies for any confusion. "Neuronal genes" is not in fact a GO term, rather a simplification of "25 neuro-synapse-axon genes" in line 310. To be more understandable, we have removed this simplification in our revision, please see line 317.

Comment: L260 fatty acid

Reply: Apologies and corrected! Please see line 319.

Comment: L269 and no gene in breast muscle

Reply: Done! Please see line 329.

Comment: L273 The results suggest that the PDC gene is of substantial functional importance in phenotypic differentiation among wild and domestic ducks.

Reply: Many thanks. We have revised this sentence according to your suggestion, please see lines 333-335.

Comment: L289 catalogued 36.1M SNPs and 3.1M INDELs,

Reply: Corrected! Please see line 349.

Comment: L333 ... showed particularly strong signs of selective sweep s presumably associated with domestication.

	Reply: We have corrected our manuscript according to your suggestion, please see lines 398-399.<br><br>Comment: L340 brain and liver of domesticated ducks compared to ...<br><br>Reply: Corrected! Please see line 405.<br><br>Comment: L351 differential selection? Do you mean directional selection?<br><br>Reply: Apologies for any confusion. We also revised our current manuscript, please see lines 416-418.<br><br>Comment: L362 Taken together, our results show that duck domestication was a relatively recent and ...<br><br>Reply: We have corrected our manuscript according to your suggestion, please see line 430.<br><br>Comment: L440 From the 28,199,227 SNPs not confirmed by dbSNPs, 390 randomly chosen (?) nucleotide sites<br><br>Reply: Many thanks for your question. Of course, all nucleotide sites were randomly selected. We have added this explain to our current manuscript, please see lines 510-513.<br><br>Comment: L448 Principal Component Analysis (PCA), first by generating the genetic relationship matrix (GRM) from which the first 20 eigenvectors were extracted.<br><br>Reply: We have corrected our manuscript according to your suggestion, please see line 520-522.<br><br>--<br>Please also take a moment to check our website at http://giga.edmgr.com/l.asp?i=25723&l=YHKU51UQ for any additional comments that were saved as attachments. Please note that as GigaScience has a policy of open peer review, you will be able to see the names of the reviewers.
**Additional Information:**	
**Question**	**Response**
Are you submitting this manuscript to a special series or article collection?	No
**Experimental design and statistics**<br><br>Full details of the experimental design and statistical methods used should be given in the Methods section, as detailed in our Minimum Standards Reporting Checklist. Information essential to interpreting the data presented should be made available in the figure legends.<br><br>Have you included all the information requested in your manuscript?	Yes
**Resources**<br><br>A description of all resources used,	Yes

Powered by Editorial Manager® and ProduXion Manager® from Aries Systems Corporation

including antibodies, cell lines, animals and software tools, with enough information to allow them to be uniquely identified, should be included in the Methods section. Authors are strongly encouraged to cite Research Resource Identifiers (RRIDs) for antibodies, model organisms and tools, where possible.<br><br>Have you included the information requested as detailed in our Minimum Standards Reporting Checklist?	
**Availability of data and materials**<br><br>All datasets and code on which the conclusions of the paper rely must be either included in your submission or deposited in publicly available repositories (where available and ethically appropriate), referencing such data using a unique identifier in the references and in the “Availability of Data and Materials” section of your manuscript.<br><br>Have you have met the above requirement as detailed in our Minimum Standards Reporting Checklist?	Yes

Manuscript

Click here to download Manuscript Manuscript_of_Duck_Domestication_revise1.docx

Whole-genome resequencing reveals signatures of selection and timing of duck domestication

Zebin Zhang [†,1], Yaxiong Jia[†,2], Pedro Almeida[3], Judith E Mank[3,4], Marcel van Tuinen[5], Qiong Wang[1], Zhihua Jiang[6], Yu Chen[7], Kai Zhan[8], Shuisheng Hou[2], Zhengkui Zhou[2], Huifang Li[9] Fangxi Yang[10], Yong He[11], Zhonghua Ning[1], Ning Yang[1], and Lujiang Qu[1*]

[1]Department of Animal Genetics and Breeding, National Engineering Laboratory for Animal Breeding, College of Animal Science and Technology, China Agricultural University, Beijing, China

[2]Institute of Animal Science, Chinese Academy of Agricultural Sciences, Beijing, China

[3]Department of Genetics, Evolution and Environment, University College London, London, UK

[4]Department of Organismal Biology, Evolutionary Biology Centre, Uppsala University, Uppsala, Sweden

[5]Centre of Evolutionary and Ecological Studies, Marine Evolution and Conservation Group, University of Groningen, Groningen, The Netherlands

[6]Department of Animal Sciences, Center for Reproductive Biology, Veterinary and Biomedical Research Building, Washington State University, Pullman, United States

[7]Beijing Municipal General Station of Animal Science, Beijing, China

[8]Institute of Animal Husbandry and Veterinary Medicine, Anhui Academy of Agricultural Sciences, Hefei, China

[9]Poultry Institute, Chinese Academy of Agriculture Science, Yangzhou, China

[10]Institute of Pekin Duck，Beijing, China

[11]Cherry Valley farms (xianghe) Co.,Ltd, Langfang, China

[†]These authors contributed equally to this work.

*Corresponding authors: quluj@163.com

Abstract

Background: The genetic basis of animal domestication remains poorly understood, and systems with substantial phenotypic differences between wild and domestic populations are useful for elucidating the genetic basis of adaptation to new environments as well as the genetic basis of rapid phenotypic change. Here, we sequenced the whole genome of 78 individual ducks, from two wild ~~populations~~ and seven domesticated populations, with an average sequencing depth of 6.42X per individual~~> 45X for each population~~.

Results: Our population and demographic analyse~~i~~s indicate~~s~~ a complex history of domestication, with early selection for separate meat and egg lineages. Genomic comparison of wild to domesticated populations suggest that genes affecting brain and neuronal development have undergone strong positive selection during domestication. Our F_{ST} analysis also indicates ~~for the first time of indicates~~that ~~–~~the duck white plumage is ~~–associated~~ the result of ~~with~~ selection at the *melanogenesis associated transcription factor* locus.

Conclusions: Our results advance the understanding of animal domestication and selection for complex phenotypic traits.

Keywords: duck, domestication, intensive selection, neuronal development, energy metabolism, plumage colouration.

~~Introduction~~Background

Animal domestication was one of the major contributory factors ~~of~~to the agricultural revolution during the Neolithic period, which resulted in a shift in human lifestyle from hunting to farming [1]. Compared with their wild progenitors, domesticated animals showed notable changes in behavior, morphology, physiology, and reproduction [2]. Detecting domestication mediated selective signatures is important for understanding the genetic basis of ~~animal~~ both adaptation to ~~a~~ new environments and rapid phenotype change~~s~~ ~~in a short period of time~~ [3, 4]. In recent years, to characterize signatures of domestication, whole genome resequencing studies have been performed on a wide range of agricultural important organisms, such as observed in pig [5], sheep [6], rabbit [7] and chicken [8, 9].

Mallards (*Anas platyrhynchos*) ~~(ducks or mallards)~~ are the world's most widely distributed and agriculturally important waterfowl species, and are of particular economic ~~and~~ importance in Asia [10]. Southeast Asia, particularly southern China~~,~~, is the major center of duck domestication, with records indicating duck farming in the region dating at least 2,000 years [11, 12], particularly in wet environments [13] associated with rice crops [14]. In the absence of archaeological evidence, the exact timing of domestication and the time of meat and egg type ducks split remains unknown, with the first written records indicating domestic ducks in central China shortly after 500 BC [15].

It is clear that the domesticated duck originated from mallards (*Anas*

Formatted: Font: Not Italic

Formatted: Font: Not Italic

platyrhynchos) [16], and domestic ducks can be classified as those produced primarily for meat (similar to chicken broilers) or eggs~~–~~ (similar to chicken layer lines). Together with the timing of duck domestication, the relative separation of duck meat and egg lines is also unknown. It is unclear whether ducks were domesticated once, and subsequently selected for divergent meat and egg production traits, or whether meat and egg populations were derived independently in two domestication events from wild mallards.

Moreover, domesticated mallards show many important behavioral [17] and morphological [18-20] differences from their wild ancestors, particularly related to plumage and neuroanatomy. However, the genetic basis of these phenotypic differences are still poorly understood.

~~, offering an important opportunity to understand the genetic basis of these phenotypic differences.~~

Data Description

In order to determine the timing of duck domestication in China, as well as identify the genomic regions under selection during domestication, we performed whole genome resequencing from 78 individuals belonging to seven different duck breeds (three for meat breeds, three for egg breeds, and one dual-purpose breed) and two geographically distinct wild populations. A total of 613.37 Gb high quality paired end sequence data were rec~~c~~overed after initial quality control. Using the large number of ~~36.1 million~~ single nucleotide

polymorphisms (SNPs) ~~and 3.1 million~~as well as small insertions and deletions (INDELs) we detected, we analyzed the structure of these populations and signatures of selection associated with domestication. We inferred the demographic scenarios with the~~by~~ pairwise sequential~~ly~~ —Markovian coalescent method combined with the diffusion approximation method. ~~We identified two distinct domesticated populations, originating from a single domestication event roughly 2000 years ago. We also identified signatures of selection on genes associated with neuronal development, energy metabolism, vision and plumage during domestication. Together, our results reveal a complex pattern of selection associated with the domestication of the duck.~~

The whole genome resequencing data and SNP and INDEL variant datasets are valuable resources ~~to~~for researchers studying evolution, domestication or trait discovery, and ~~to~~for breeders of *Anas platyrhynchos*~~s.~~*s.* Furthermore, the data represent a foundation for development of new, ultrahigh density variant screening arrays for duck population level trait analysis and genomic selection.

Formatted: Font: Italic

~~Results~~Analyses

Genetic variation

We individually sequenced 22 wild and 56 domestic ducks, from two wild populations and seven domestic breeds (three meat breeds, three egg breeds

and one dual-purpose breed), from across China (Fig. 1A) to an average of 6.42X coverage per individual after filtering and quality control, resulting in total 535 billion mappable reads across 78 duck individuals (Supplemental Table S1).

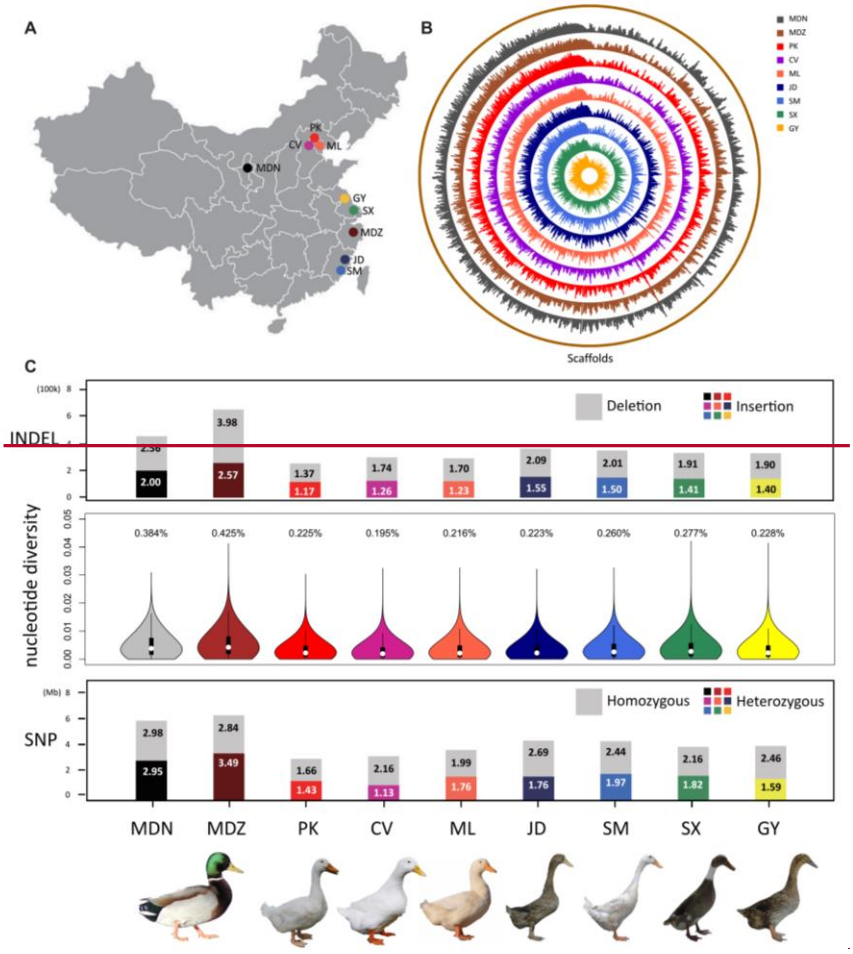

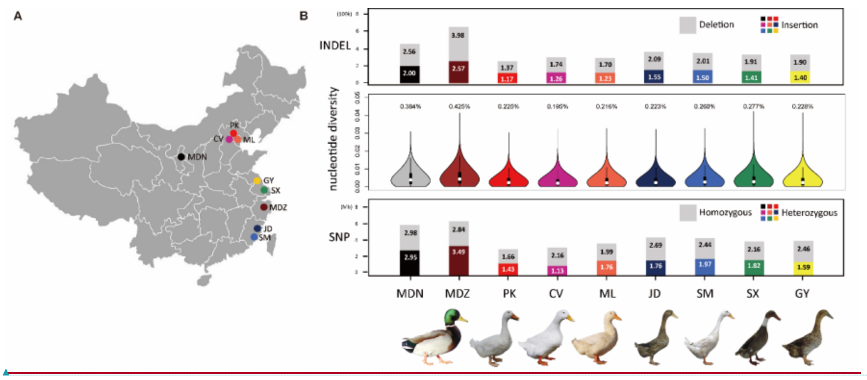

Figure. 1 Experimental design and variants statistics

(A) Sampling sites in this study. A total of 78 ducks from two wild populations (Mallard Ningxia (MDN) n=8; Mallard Zhejiang (MDZ) n=14), three meat breeds (Pekin (PK) n=8; Cherry Valley (CV) n=8; Maple Leaf (ML) n=8), three egg breeds (Jin Ding (JD) n=8; Shan Ma (SM) n=8; Shao Xing (SX) n=8), and one dual purpose breed (Gao You (GY) n=8) were selected.

~~**(B)** Circos plot of SNP distribution and density of seven domestic breeds and two wild populations across the genome. The duck whole genome reference is shown in the outermost circle (non-overlapping, window size = 1 Mb).~~

(~~C~~B) Genomic variation of nine populations ~~ducks~~. Mean number of SNPs, heterozygous and homozygous SNP ratio in the nine populations ~~as~~ are shown at the bottom. ~~Homozygous SNP ratios in domesticated ducks are significantly higher than ratios in wild mallards ($p = 1.35 \times 10^{-10}$).~~ Nucleotide diversity ratios of ~~in~~ the nine populations are shown at the middle. The nucleotide diversity ratios in wild mallards are dramatically higher than ratios in domesticated ducks ~~($p = 2.20 \times 10^{-16}$)~~. Number of insertions and deletions in the nine populations are shown at the top. The number of deletions was higher than the number of insertions in all nine populations.

Formatted: Font:

We detected 36.1 million (M) SNPs in total, with an average for each individual of 4.5M SNPs (range = 2.34 – 9.52M), ~~which covered~~ covering 96.2% of the duck dbSNP database deposited in the Genome Variation Map (GVM) (http://bigd.big.ac.cn/gvm/). We also identified 3.1M INDELs, with an average of 0.4M INDELs (range = 0.21 – 0.89M) (Fig. ~~1C~~1B, Supplemental Figs. S1 - S2, Supplemental Table S2). Single base-pair INDELs were the predominant form, accounting for 38.63% of all detected INDELs (Supplemental Table S3). Both the number of SNPs (t test, $p = 3.13 \times 10^{-12}$) and nucleotide diversity (t test, $p = 2.20 \times 10^{-16}$) are lower in domesticated compared to wild mallards (Fig. 1B~~ - C~~), and homozygous SNP ratios in domesticated ducks are significantly higher than ratios in wild mallards (t test, $p = 1.35 \times 10^{-10}$) consistent with the larger panmictic wild population. ~~Single base-pair INDELs were the predominant form, accounting for 38.63% of all detected INDELs (Supplemental Table S3).~~

Population structure and domestication

Phylogenetic relationships, based on a neighbor-joining (NJ) of pairwise genetic distances of whole genome SNPs (Fig. 2A) and Principal Component Analysis (PCA, Fig. 2B) revealed strong clustering into three distinct genetic groups. The two wild populations (MDN and MDZ) clustered together, ~~with~~ the three meat type population ducks (PK, CV, and ML) clustered together into a

Formatted: Font: 12 pt

Formatted: Font: 12 pt

Formatted: Font: 12 pt

Formatted: Font: 12 pt

Formatted: Font: 12 pt

second group, and the three egg type populations (JD, SM, and SX) clustered with the dual-purpose type ducks (GY) into a third group.

We further performed population structure analysis using FRAPPE [21], which estimates individual ancestry and admixture proportions assuming K ancestral populations (Fig. 2C). With K = 2, a clear division was found between wild type ducks (MDN and MDZ) and domesticated ducks (PK, CV, ML, JD, SM, SX, and GY). With K = 3, a clear division was found between meat type ducks (PK, CV, and ML) and egg type ducks mixed with dual-purpose type ducks (JD, SM, SX, and GY).

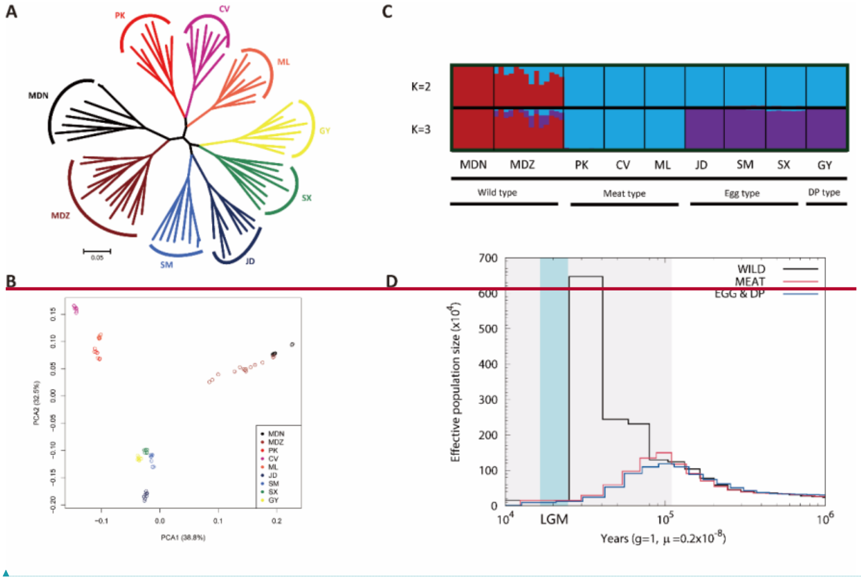

Formatted: Font: Bold

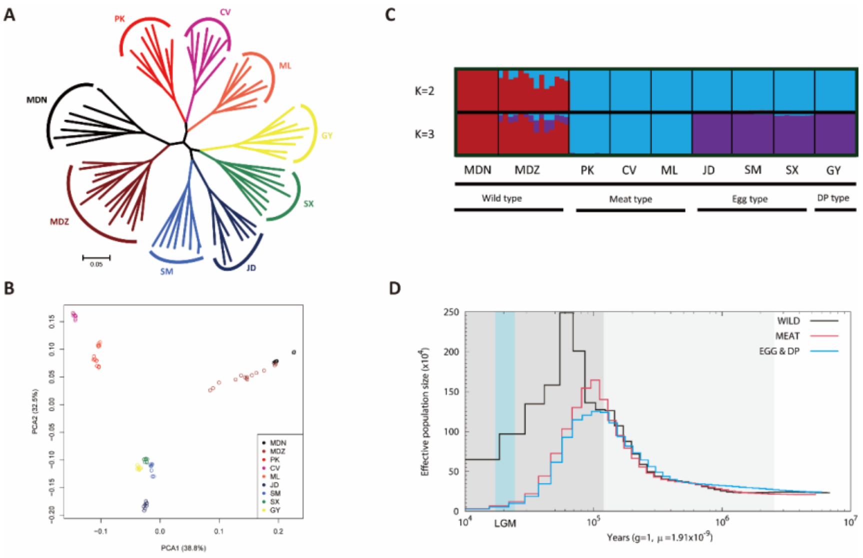

Figure. 2 Population genetic structure and demographic history of nine duck populations

(A) Neighbor-joining phylogenetic tree of nine duck populations. The scale bar is proportional to genetic differentiation (p dist ance).

(B) PCA plot of duck populations. Eigenvector 1 and 2 explained 38.8% and 32.5% of the observed variance, respectively.

(C) Population genetic structure of 78 ducks. The length of each colored segment represents the proportion of the individual genome inferred from ancestral populations (K = 2-3). The population names and production type are at the bottom. DP type means dual-purpose type.

(D) Demographic history of duck populations. Examples of PSMC estimate changes in the effective population size over time, representing variation in inferred Ne dynamics. The lines represent inferred population sizes and the gray shaded areas indicate the Pleistocene period,

with Last Glacial Period (LGP) shown in darker gray, and Last Glacial Maximum (LGM) shown in light blue areas.

Together, these results indicate two genetic clusters of domesticated breeds, either domesticated once with subsequent subdivision due to divergent selection, or domesticated twice independently. In order to differentiate these alternatives, we explored the demographic history of our samples, first estimating changes in effective population size (N_e) in our three genetic clusters in a pairwise sequential~~ly~~ Markovian coalescent (PSMC) framework [22]. The meat type ducks (PK, CV, and ML) showed concordant demographic trajectories with egg and ~~mixture~~ dual-purpose~~type~~ populations (JD, SM, SX, and GY) with one apparent expansion around the Penultimate Glaciation Period (PGP, 0.30-0.13 Mya) [4, 23] and Last Glacial Period (LGP, 110-12 kya) [24, 25], followed by a subsequent contraction (Fig. 2D).

We tested multiple demographic scenarios related to domestication using a diffusion approximation method for the allele frequency spectrum (∂a∂i) (Supplemental Fig. S3 and S4). Among the four isolation models tested (models 1 - 4), the model of a single domestication with subsequent divergence of the domesticated breeds (Model 2) was both consistent with our population structure results (Fig. 2) and had the lowest Akaike Information Criteria (AIC) value, indicating a better overall fit to the data (log-likelihood = -33,388.43; AIC = 66,788) (Supplemental Fig. S3).

Demographic parameters estimated from the single domestication model (Model 2) indicated that domestication occurred ~~approximately 2,200~~2,228, with 95% confidence intervals (CI) ± 441 years ago, followed by a rapid subsequent divergence of the meat breed from the egg/dual purpose breeds roughly 100 years after the initial domestication event (Table 1). Our results suggest that following an initial bottleneck associated with domestication, with an estimated N_e of ~~305~~ 320 (95% CI ± 3) individuals for the ancestral domesticated population, the population has expanded to the current N_e of 5,597 (95% CI ± 1,195) ~~5,345~~ and 12,988 (95% CI ± 2,877) ~~12,404~~ in the meat type and egg/dual purpose breeds respectively. N_e estimates for domesticated breeds are lower than ~~that~~ in wild mallards, consistent with the large panmictic wild population.

Table 1. Maximum likelihood population demographic parameters. Best fit parameter estimates for the model of a single domestication event followed by divergence of the domesticated breeds, including changes in population size. 95% confidence intervals were obtained from 100 bootstrap data sets. Time estimates are given in years and migration are in units of number of migrants per generation.

**Parameter**	**ML estimate**	**95% CI**
N_e of ancestral population after size change	663,439~~633,584~~	644,726 – 68[illegible]
N_e of the wild population	88,842~~84,845~~	70,778 – 106,[illegible]
N_e of the ancestral domesticated population	~~305~~320	316 – 323
N_e of the meat breed	5,597~~5,345~~	4,402 – 6,792[illegible]
N_e of the egg/dual purpose	12,988~~12,404~~	10,111 – 15,8[illegible]
Time of size change in the ancestral population	249,944~~238,696~~	227,912 – 26[illegible]
Time of domestication	2,228~~2,128~~	1,787 – 2,669[illegible]
Time of breed divergence	2,126~~2,030~~	1,686 – 2,567[illegible]
Migration $_{wild \leftarrow meat}$	1.~~2~~12	1.00 – 1.24

Formatted: Indent: First line: 0 ch
Formatted Table
Formatted: Indent: First line: 0 ch
Formatted: Indent: First line: 0 ch
Formatted: Indent: First line: 0 ch
Formatted: Indent: First line: 0 ch
Formatted: Indent: First line: 0 ch
Formatted: Indent: First line: 0 ch
Formatted: Indent: First line: 0 ch
Formatted: Indent: First line: 0 ch
Formatted: Indent: First line: 0 ch

Migration $_{wild \leftarrow egg/dp}$	3.92	3.11 – 4.73

Formatted: Indent: First line: 0 ch

Gene flow estimates were relatively high, and were 1 and 4 migrants per generation from the meat and egg/dual purpose breeds, respectively, into the wild population. Difficulty in differentiating between very recent divergence and high migration rates in the frequency spectrum prevented convergence between independent runs when trying to fit other migration parameters to our model.

Selection for plumage color

Derived traits in domesticated animals tend to evolve in a predictable order, with color variation appearing in the earliest stages of domestication, followed by coat or plumage and structural (skeletal and soft tissue) variation, and finally behavioral differences [26, 27]. One of the simplest and most visible derived traits of ducks is white plumage color. In order to detect the signature of selection associated with white feathers, we searched the duck genome for regions with high F_{ST} among the populations of white feather (PK, CV, and ML) and non-white feather (MDN, MDZ, JD, SX, and GY) based on sliding windows of 10kb windows. We identified a region of high differentiation between white plumage and non-white plumage ducks overlapping the *melanogenesis associated transcription factor* (*MITF*; F_{ST}=0.69) (Fig. 3A). In the intronic region of *MITF*, we identified 13 homozygous SNPs and 2 homozygous INDELs present in all white plumage breeds (n=24). These ~~SNPS~~ variants were absent in all non-white plumage breeds (n=46) (Fig. 3B). These mutations were

completely consistent with ~~the~~ white plumage phenotype suggesting a~~s~~ causative mutation. Our result ~~first~~ indicate~~s for the first time~~ the duck white plumage is completely associated with selection at the *MITF* locus. Moreover, to validate the reliability of variants detected in MITF gene, we amplified the first three SNPs (SNP817793, SNP817818, and SNP818004) and all INDELs by diagnostic PCR combined with Sanger sequencing in the 78 white and non-white plumage ducks. The results show that the three SNPs and INDEL817958 completely match our NGS analysis (supplemental Fig. S5), For INDEL818495, we were unable to design a suitable PCR primer.

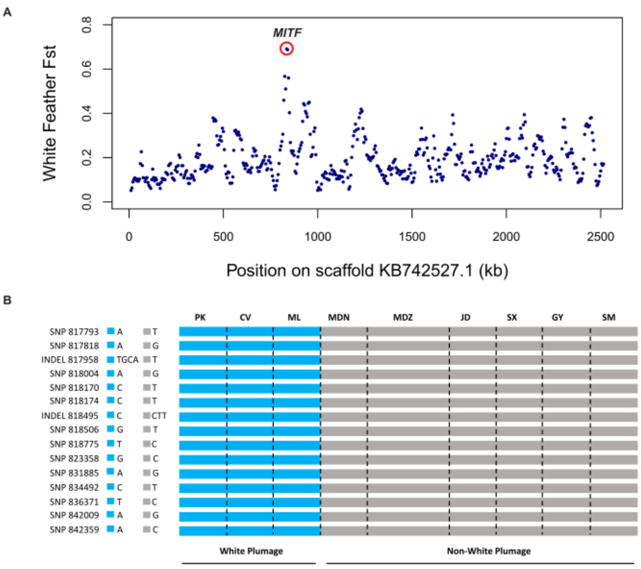

Figure. 3 MITF shows different genetic signature between white plumage and non-white plumage ducks.

(A) FST plot around the MITF locus. The FST value of MITF is highest for scaffold KB742527.1, circled in red. Each plot represent a 10 kb windows.

(B) 13 homozygous SNPs and 2 homozygous INDELs were identified in white plumage ducks and absent in non-white plumage ducks. SNPs and INDELs were named according to their position on scaffold.

Selection for other domestication traits

In order to detect the signature of selection for other traits associated with duck domestication, we scanned the duck genome for regions with a high coefficient of nucleotide differentiation (F_{ST}) among the populations of wild types (MDN and MDZ) and domesticated types (PK, CV, ML, JD, SM, SX, and GY) based on sliding windows of 10kb size windows, as well as global F_{ST} between each population (Supplemental Tables S4). Owing to the complex and partly unresolved demography of these populations, it is difficult to define a strict threshold that distinguishes true sweeps from regions of homozygosity caused by drift. We therefore also calculated pairwise diversity ratio (θ_π(wild/domesticated)). We identified 292 genes in the top 5% of both F_{ST} and θ_π scores, putatively under positive selection during domestication (Fig. 4A, Supplemental Tables ~~S4~~S5).

Formatted: Subscript

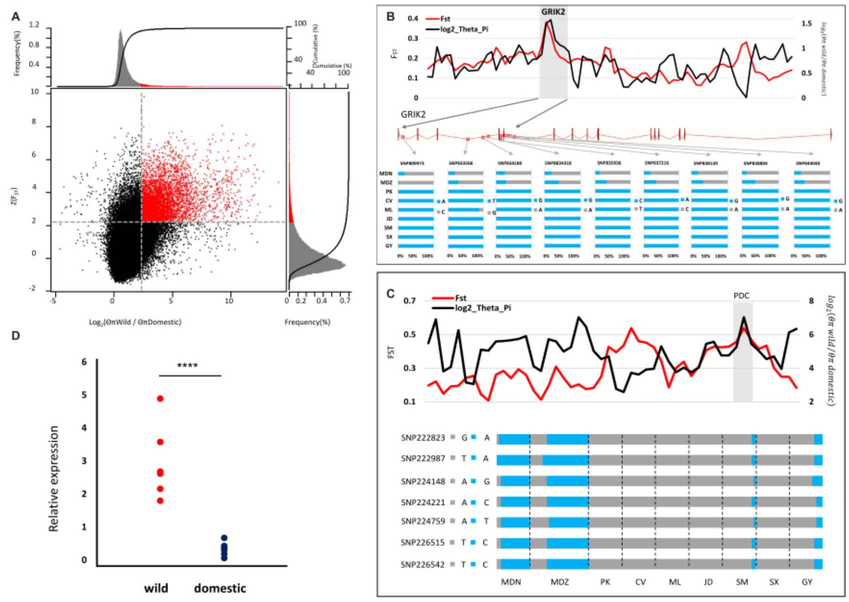

Figure. 4 Genomic regions with strong selective sweep signals in wild population ducks and domesticated population ducks.

(A) Distribution of θπ ratios θπ(wild/domesticated) and Z(F_{ST}) values, which are calculated ~~using scaffolds longer than 10 kb~~ by 10-kb windows with 5-kb steps. Only scaffolds > 10kb were used for our calculation, as F_{ST} result calculated on small scaffold are unlikely to be accurate. Red data points located to the top-right regions correspond to the 5% right tails of empirical $log_2(\theta\pi\, wild/\theta\pi\, domestic)$ ratio distribution and the top 5% empirical Z(F_{ST}) distribution are genomic regions under selection during duck domestication. The two horizontal and vertical gray lines represented the top 5% value of Z(F_{ST}) (2.216) and $log_2(\theta\pi\, wild/\theta\pi\, domestic)$ (2.375), respectively.

(B) $log_2(\theta\pi)$ ratios and F_{ST} values around the *GRIK2* locus and allele frequencies of nine SNPs within the *GRIK2* gene across nine duck populations. The black and red lines represent $log_2(\theta\pi\, wild/\theta\pi\, domestic)$ ratios and F_{ST} values, respectively. The gray bar

Formatted: Subscript

showed the region of under strong selection in *GRIK2* gene. The nine red rectangular frame corresponding to the locus on gene of nine SNPs. The SNPs were named according to their position on scaffold.

(C) The PDC gene showed different genetic signature in domesticated and wild duck. $log_2(\theta\pi)$ ratios and F_{ST} values around the *PDC* locus. The *PDC* gene region i~~ wa~~s ~~showed~~ shown in gray~~ par~~. Allele frequencies of seven SNPs within the *PDC* gene across nine duck populations. The SNPs are~~were~~ named according to their scaffold p~~p~~ositio~~n on scaffold~~n.

(D) The PDC gene expression level ~~different~~ differs between~~in~~ domesticated and wild duck. PDC mRNA expression levels in brain of wild (MDN, n=3; MDZ, n=4) and domesticated (PK, n=1; CV, n=1; ML, n=1; JD, n=1; SM, n=1; SX, n=1; GY, n=1) ducks. ****P value from *t*-test (P<0.0001).

All 292 genes located in the 5% FST regions were used for the GO analysis, resulting in a total of 57 GO enrichment terms (supplementary table S6). Because domesticated ducks are known to differ from wild ducks in body size, body fat percentage, behavior, egg productivity, growth speed, and flight capability, we focused our analysis on GO annotations of neural related processes, lipid metabolism and energy metabolism, reproduction, and skeletal muscle contraction for our 292 putative positively selection genes. In this reduced data set, the neuro-synapse-axon and lipid-energy metabolism pathways were over-represented (Supplemental Table ~~S5~~S7) in our list of genes under selection.

From the highlighted GO terms, a total of 25 neuro-synapse-axon genes

were identified as being under positive selection, with six (*ADGRB3*, *EFNA5*, *GRIN3A*, *GRIK2*, *SYNGAP1*, and *HOMER1*) in the top 1% of F_{ST} and θ_π (Supplemental Tables ~~S6~~S8). In particular, *GRIK2* (glutamate receptor, ionotropic kainate 2) and *GRIN3A* (glutamate receptor, subunit 3A) both showed high F_{ST} and θ_π value compared to neighboring regions, suggesting functional importance (Fig. 3B, Supplemental Table ~~S4~~S5, ~~S6~~S8).

Beyond the neuronal-synapse-axon genes, 115 genes were identified in the four lipid and energy related pathways with high F_{ST} and θ_π values, particularly related to ~~gatty~~ fatty acid metabolism. Among these genes, 37 genes were found with both parameters yielding top 1% ranked values (Supplemental Tables ~~S6~~S8), such as phosphatidylinositol 3-kinase catalytic subunit type 3 (*PIK3C3*), and patatin like phospholipase domain containing 8 (*PNPLA8*).

To infer whether selection extends beyond ~~yielding novel~~ allelic variation and ~~by~~ also affects~~ing~~ gene expression, we compared individual gene expression in the brain, liver, and in breast muscle between seven wild mallards and seven domesticated ducks in natural states with RNA-seq (Supplemental Tables ~~S7~~S9). We detected three genes (*PDC*, *MLPH*, and *NID2*) in the brain, two genes (*MAPK12* and *BST1*) in the liver, and ~~zero~~ no genes~~s~~ in breast muscle with significantly different expression between wild and domesticated ducks. Of the five differentially expressed genes, *PDC* was the only gene which also showed evidence of a selective sweep at the genomic level (Supplemental

Tables ~~S4~~S5, Fig. 3C - D). The results ~~imply~~ suggest that the *PDC* gene is of substantial functional importance in phenotypic differentiation among wild and domestic ducks~~ through both allelic and expression differences~~.

Discussion

Animal domestication was one of the major contributory factors of the agricultural revolution during the Neolithic period, which resulted in a shift in human lifestyle from hunting to farming [1]. Since this transition, domesticated animals have contributed greatly to human society and human population growth by provision of stable animal protein, fat, and accessory products such as leather and feathers (including down). Whole genome sequencing has made it possible to illuminate the genetic trajectories of animal domestication such as those observed in pig [5], sheep [6], rabbit [7] and chicken [8, 9].

In this study, we performed whole-genome sequencing of 78 ducks including seven domesticate breeds and two wild populations. This is the first study to characterize the genetic architecture, phylogenetic relationships and domestication history of domesticated ducks and wild mallards. We first catalogued ~~millions of~~ 36.1M SNPs and 3.1M INDELs, and in both types of variants~~cases~~, we observed higher mean variant numbers and nucleotide diversity for the wild mallard populations compared to the domestics, consistent with both a greater panmictic mallard population as well as recent sweeps associated with domestication.

Population structure and domestication

We observed a large expansion of the duck population at the interglacial period, which could be the result of beneficial climatic changes, including rising temperatures and sea levels. In contrast, the glacial maximum coincided with a much reduced duck population size, consistent with harsher conditions and limited access to arctic breeding grounds [4, 28-30]. The demographic pattern we observe in wild ducks is similar to that observed in wild boars [5], wild yaks [31], and wild horses [32]. However, it is worth noting that although PSMC is a powerful method to infer changes in N_e over time, it is also sensitive to deviations from a neutral model. The effects of genetic drift and/or selection could lead to time-dependent estimates of mutation rate, and bias our estimates of population expansion [25].

We observed three genetic clusters, with wild mallard, meat breeds, and egg/dual purpose breeds each representing unique groups. These results suggest either a single domestication event followed by subsequent breed-specific selection, or two separate domestication events. In order to distinguish alternative models of domestication, we modeled population demographics and found strong support for a single domestication event roughly 2,~~100~~ 200 years ago, with the rapid subsequent selection for separate meat and egg/dual purpose breeds roughly 100 generations later. We note that the evolutionary history of wild mallards and domesticated duck breeds is likely to be more complex than the simple demographic scenarios modelled here, and further

studies may be needed to fully capture the evolutionary dynamics of duck domestication. Given the recent origin of wild ducks, as well as the high levels of diversity we observe in the wild and domestic duck genomes, it is not possible to differentiate recent admixture from incomplete lineage sorting with our current data. This issue has important conservation implications, and represents an interesting area for future study. ~~-~~Nevertheless, the time estimates obtained with our model are compatible with previous written records from 500 BC [15].

Selection for white plumage

Plumage color is an important domestication trait, and we compared breeds with white plumage to those with colored plumage. We identified high levels of divergence in the intronic region of the *MITF* gene, an important developmental locus with a complex regulation implicated in pigmentation and melanocyte development in sever~~v~~al vertebrate species [33-35], including Japanese quail [36]~~ and~~, dog [37], and duck[38, 39].

Selection for other domestication traits

In order to identify those genomic regions which have been the target of selection during domestication, we used estimates of diversity between wild and domestic samples, retaining those 292 genes in the top 5% of both F_{ST} and θ_π values for further analysis. These genes were over-represented for both neural developmental and lipid metabolism, suggesting that these

functionalities were under strong selection during domestication. Two loci, *GRIK2 and GRIN3A*, showed particularly strong ~~signatures of genetic~~signs of selective sweeps presumably associated with domestication. *GRIK2* encodes a subunit of a glutamate receptor that has a role in synaptic plasticity and is important for learning and memory. *GRIN3A* encodes a subunit of the N-methyl-D-aspartate (NMDAR) receptors, which is expressed abundantly in the human cerebral cortex [40] and is involved in the development of synaptic elements

We also identified five genes with significantly different expression in the brain and liver of ~~domestics~~ domesticated ducks compared to their wild ancestor. One of these, *PDC*, also showed evidence of selective sweeps at the genomic level. *PDC* encodes phosducin, a photoreceptor-specific protein highly expressed in retina and pineal gland [41], as well as the brain [42].

Our results suggest that *PDC*, *GRIK2* and *GRIN3A* may have played a crucial role in duck domestication by altering functional regulation of the developing brain and nervous system. This finding is consistent with theories that behavioral traits are the most critical in the initial steps of animal domestication, allowing animals to tolerate humans and captivity [43, 44]. Indeed, compared to wild mallards, domestic ducks are more docile, less vigilant, and show important differences in brain morphology [17, 18]. Interestingly, ~~differential selection~~ differences~~s~~ between wild and domesticated animals i~~o~~n brain and nervous system functions due to~~by~~ directional selection were~~as~~ also observed in domestication studies of rabbits [7], dogs [45],

chickens [8]. In particular, *GRIK2* was also found to play a crucial role during rabbit domestication [7].

Besides brain and nervous system related genes, we also identified several genes that play an important function in lipid and energy metabolism. For example, *PIK3C3* plays an important role in ATP binding but also regulates brain development and axons of cortical neurons [46-50]. *PNPLA8* is involved in facilitating lipid storage in adipocyte tissue energy mobilization and maintains mitochondrial integrity [51, 52], as well as plays a role in lipid metabolism associated with neurodegenerative diseases [53-55]. *PRKAR2B* is associated with body weight regulation, hyperphagia, and other energy metabolism [56, 57].

Taken together, our results show that duck domestication was a relatively recent and complex process, and the genetic basis of domestication traits show many striking overlaps with other vertebrate domestication events.

Methods

Ethics statement

The entire procedure was carried out in strict accordance with the protocol approved by the Animal Welfare Committee of China Agricultural University (Permit Number: XK622).

Sample selection

78 ducks were chosen for sequencing, seven different populations of domesticated ducks and two population of mallards from different geographic regions. The domesticated ducks include three meat type populations *i.e.*, Pekin duck (PK; n=8); Cherry Valley duck (CV; n=8); Maple Leaf duck (ML; n=8), three egg type populations *i.e.*, Jin Ding duck (JD; n=8); Shao Xing duck (SX; n=8); Shan Ma duck (SM; n=8), one egg and meat dual-purpose type (DP type) population *i.e.*, Gao You duck (GY; n=8), and two wild populations come from two different provinces in China with separated by nearly 2,000 km distance *i.e.*, Mallard from Ningxia province (MDN; n=8); Mallard form Zhejiang province (MDZ; n=14). The classification of production types follow the description of Animal Genetic Resources in China Poultry [58]. PK, CV, and ML ducks originated from Beijing; JD and SM ducks originated from Fujian province while SX and GY ducks originated from Jiangsu province. Whole blood samples were collected from brachial veins of ducks by standard venipuncture.

In addition, 14 male ducks (MDNM, n=3; MDZM, n=4; PKM, n=1; CVM, n=1; MLM, n=1; JDM, n=1; SMM, n=1; SXM, n=1; GYM, n=1) were chosen for RNA-seq.

Sequencing and mapping statistic of individual ducks in genome and transcriptome analysis were detailed in supplementary files (Supplemental Table S1, S7).

Sequencing and library preparation

Genomic DNA was extracted using standard phenol/chloroform extraction method. For each sample, two paired-end libraries (500 bp) were constructed according to manufacturer protocols (Illumina), and sequenced on the Illumina Hiseq 2500 sequencing platform. From each populations, we sequenced seven samples at 5X depth and one at 10X coverage, except for the MDN population, where we sequenced seven individuals at 5X coverage and one at 20X coverage and the MDZ population, where we sequenced all individuals at 10X coverage. We generated a total of 628.37 Gb of paired-end reads of 100 bp (or 150 bp; MDZ) length (Supplemental Table S1).

mRNA from brain, liver, and breast muscle of 14 individual ducks were extracted using standard trizol extraction methods. For each samples, ~~Two~~ two paired-end libraries (500 bp) were constructed according to manufacturer instruction (Illumina). All samples were sequenced by Illumina Hiseq 4000 sequencing platform with the coverage of 6X.~~,~~ ~~with 32M paired-end 150 bp mapped reads~~ We generated total of 278.62 Gb of paired-end reads of 150 bp length (Supplemental Table S9).

~~per sample after QC (Supplemental Table S7).~~

Read alignment and variant calling

To avoid low quality reads, mainly the result of base-calling duplicates and adapter contamination, we filtered out sequences according to the default

parameters of NGS QC Toolkit (v2.3.3) [59]. Those paired reads which passed Illumina's quality control filter were aligned using BWA-MEM (v0.7.12) to version 1.0 of the *Anas platyrhynchos* genome (BGI_duck_1.0) [10]. Duplicate reads were removed from individual samples alignments using Picard tools MarkDuplicates, and reads were merged using MergeSamFiles (http://broadinstitute.github.io/picard/).

The Genome Analysis Toolkit (GATK, v3.5) RealignerTargetCreator and IndelRealigner protocol were used for global realignment of reads around INDELs before variant calling [60, 61]. SNPs and small indels (1-50 bp) were called used the GATK UnifiedGenotyper set for diploids with the parameter of minimum quality score of 20 for both mapped reads and bases to call variants, similarly to previous studies [62-66]. We filtered variants both per population and per individual using GATK according to the stringent filtering criteria. For SNPs of population filter: a.) QUAL > 30.0; b.) QD > 5.0; c.) FS < 60.0; d.) MQ > 40.0; e.) MQRankSum > -12.5; f.) ReadPosRankSum > -8.0; Additionally, if there were more than 3 SNPs clustered in a 10 bp window, all three SNPs were considered as false positives and removed [67].

We used the following population criteria to identify INDELs: QUAL > 30.0, QD > 5.0, FS < 200.0, ReadPosRankSum > -20.0. Of individual filter, we also removed all INDELs and SNPs where the depth of derived variants was less than half the depth of the sequence. All SNPs and INDELs were assigned to specific genomic regions and genes using SnpEff (v4.0) [68] based on the

Ensembl duck annotations. After filtering a total of 36,107,949 SNPs and 3,082,731 INDELs were identified (Supplemental Table S2).

SNP validation

In order to evaluate the reliability of our data, we compared our SNPs to the duck dbSNP database deposited in the Genome Variation Map (GVM) at the Big Data Center in the Beijing Institute of Genomics, Chinese Academy of Science (http://bigd.big.ac.cn/gvm/). 7,908,722 SNPs were validated in the duck dbSNP database, which covered 96.2% of the database (Supplemental Table S2). For the 28,199,227 SNPs not confirmed by dbSNPs, 390 randomly selected nucleotide sites were further validated diagnostic PCR combined with Sanger sequence method described in our previous research [69]. The result showed~~by PCR~~ ~~with~~ 100% accuracy, indicating the high reliability of the called SNP variation identified in this study.

Population structure

We removed all SNPs with a minor allele frequency (MAF) <= 0.1 and kept only SNPs that occurred in more than 90% of individuals. Vcf files were converted to hapmap format with custom perl scripts, and to PLINK format file by GLU v1.0b3 (https://code.google.com/archive/p/glu-genetics/) and PLINK v1.90 [70, 71] when appropriate. We used GCTA (v1.25) [72] for Principle Component Analysis (PCA), first by generating the genetic relationship matrix

(GRM) ~~followed by~~from which the first 20 eigenvectors were extracted.

To estimate individual admixture assuming different numbers of clusters, the population structure was investigated using FRAPPE v1.1 [21] base on all high quality SNPs information, with a maximum likelihood method. We increased the coancestry clusters spanning from 2 to 4 (Supplemental figure S6), because there are four duck types (wild type, meat type, egg type, and dual-purpose type) across the nine duck populations~~We used all high quality SNPs to infer population structure using FRAPPE 1.1~~ [21], with 10,000 iterations per run.

A distance matrix was generated by calculating the pairwise allele sharing distance for each pair of all high quality SNPs. Multiple alignment of the sequences was performed with MUSCLE (v3.8) [73]. A neighbor-joining maximum likelihood phylogenetic tree was constructed with the DNAML program in the PHYLIP package v3.69 [74] and MEGA7 [75, 76]. All implementation was performed according to the recommended manipulations of SNPhylo [77].

Demographic history reconstruction

The demographic history of both wild and domesticated ducks was inferred using a hidden Markov model approach as implemented in Pairwise Sequentially Markovian Coalescence based on SNP distributions [22]. In order to determine which PSMC (v0.6.5) settings were most appropriate for each

population, we reset the number of free atomic time intervals (-p option), upper limit of time to most recent common ancestor (TMRCA) (-t option), and initial value of $r = \theta/\rho$ (-r option) according to previous research [25] and online suggestions by Li and Durbin (https://github.com/lh3/psmc). Based on estimated from the ~~zebra finch~~chicken genome, an average mutation rate (μ) of ~~2~~1.9~~5~~1 $\times 10^{-9}$ per base per generation and a generation time (g) of 1 year were used for analysis~~ [78, 79] ~~[80].

Three-population demographic inference was performed using a diffusion-based approach as implemented in the program ∂a∂i (v1.7) [81]. To minimize potential effects of selection that could interfere with demographic inference, these analyses were performed using the subset of noncoding regions across the whole genome and spanning 750,939,264 bp in length. Noncoding SNPs were then thinned to 1% to alleviate potential linkage between the markers. The final dataset consisted of 95,181 SNPs with an average distance of 7,112 bp (± 18,810 bp) between neighbouring SNPs. To account for missing data, the folded allele frequency spectrum for the three populations (wild, meat and egg/dual purpose breeds) was projected down in ∂a∂i to the projection that maximized the number of segregating SNPs, resulting in 92,966 SNPs.

We tested four different scenarios to reconstruct the demographic history of the domesticated breeds of mallards: simultaneous domestication of the meat and egg and dual purpose breeds (Model 1); a single domestication event followed by divergence of the meat and egg and dual purpose breeds (Model

2); two independent domestication events, with the meat type breed being domesticated first (Model 3); and two independent domestication events, with the egg and dual purpose breed being domesticated first (Model 4). Using the “backbone” of the best model, we then used a step-wise strategy to add parameters related with variation in population sizes and population growth, keeping a new parameter only if the Akaike information criterion (AIC) and log likelihood improved considerably over the previous model with less parameters. In cases where additional parameters resulted in negligibly improved AIC and likelihood, we retained the simpler, less parameterized model. Gene flow was modelled as continuous migration events after population divergence. Each model was run at least ten times from independent starting values to ensure convergence to the same parameter estimates. We rejected models where we failed to obtain convergence across the replicate runs. Scaled parameters for the best-supported model were transformed into real values using the same average mutation rate (μ) and (g) as described above for the PSMC analysis. Parameter uncertainty was obtained using the Godambe Information Matrix (GIM) [82] from 100 non-parametric bootstraps.

Selective-sweep analysis

In order to define candidate regions having undergone directional selection during duck domestication we calculated the coefficient of nucleotide differentiation (F_{ST}) between mallards and domesticated ducks described by

Weir & Cockerham [83]. We calculated the average F_{ST} in 10kb windows with a 5 kb shift for all seven domesticated duck populations combined, and two mallard populations combined. Only scaffolds longer than 10 kb, 2368 of 78488 scaffolds, were chosen for the analysis. We transformed observed F_{ST} values to Z transformation (Z(F_{ST})) with $\mu = 0.1154$ and $\sigma = 0.0678$ according to previously described methods [84].

To estimate levels of nucleotide diversity (π) across all sampled populations we used the VCFtools software (v0.1.13) [85] to calculate $\theta\pi(\mathrm{wild/domesticated})$ [86], computing the average difference per locus over each pair of accessions. As the measurement of F_{ST}, averaged π ratio ($\theta\pi(\mathrm{wild/domesticated})$) was calculated for each scaffold in 10kb sliding windows.

Functional classification of GO categories was performed in Database for Annotation, Visualization and Integrated Discovery (DAVID, ver 6.8) [87]. Statistical significance was accessed by using a modified Fisher's exact test and Benjamini correction for multiple testing.

RNA-seq and data processing

To infer whether novel allelic variants located in the top 5% F_{ST} regions of genome comparison between wild mallards and domesticated ducks could also affecting gene expression, we compared gene expression in brain, liver and in breast muscle between wild mallards and domesticated ducks. To make our

Formatted: Subscript

result more universal, 7 male mallards and 7 male domesticated ducks were choose for RNA-seq. All samples were individually sequenced by Illumina Highseq 4000 sequencing platfrom.

For each sample, adapters and primers of paired end reads were removed by NGSQC Tool kit (v2.3.3) [59]. For each paired end read pair, if one of two reads had an average base quality less than 20 (PHRED quality score), then both reads were removed. If one end of paired end read had percentage of high quality base less than 70%, the two paired reads also removed. After that ~~H~~high-quality reads were mapped to reference genome using STAR (v.2.5.3a) [88]. The *featureCounts* function of the *Rsubread* (v.1.5.2) [89, 90] was used to output the counts of reads aligning to each gene. We detected the differential expression genes with edgeR (v3.6) [91-94] using a $p_{adj} < 0.05$ threshold.

Availability of supporting data and materials ~~Data Access~~

The 78 ducks used in whloe genome resequencing analysis and the 14 ducks used in RNA-seq analysis are accessible at NCBI BioProject (http://www.ncbi.nlm.nih.gov/bioproject) under accession numbers PRJNA419832 and PRJNA419583, respectively. The unassessembled sequencing reads of 78 ducks and RNA-seq reads of 14 ducks have been deposited in NCBI Sequence Read Archive (SRA: http://www.ncbi.nlm.nih.gov/sra) under accession numbers SRP125660 and

SRP125529, respectively. All VCF files of SNPs and INDELs and other supporting data were submitted to *Giga*DB datavase.

~~All duck sequence data had been submitted to Genome Sequence Archive (GSA) database of BIG Data Center in Beijing Institute of Genomics (BIGD) with accession number of CRA000523.~~

Declarations~~Acknowledgments~~

Funding

This work was supported by the earmarked fund for the Beijing Innovation Team of the Modern Agro-industry Technology Research System (BAIC04-2017), European Research Council (grant agreement 680951) and Wolfson Merit Award. We gratefully acknowledge our colleagues in the Poultry Team at the National Engineering Laboratory for Animal Breeding of China Agricultural University, for their assistance on sample collection and helpful comments on the manuscript.

Authors' contributions

Conceived and designed the experiments: Lujiang Qu. Wrote the paper: Zebin Zhang. Revised the paper: Lujiang Qu, Judith E Mank, Marcel van Tuinen. Analyzed the data: Zebin Zhang, Pedro Almeida, Qiong Wang, Yaxiong Jia. Performed the experiments: Zebin Zhang, Yaxiong Jia. Contributed reagents/materials: Zhihua Jiang, Yu Chen, Kai Zhan, Shuisheng Hou,

Formatted: Font: Italic

Formatted: Heading 3, Indent: First line: 0"

Formatted: Underline

Formatted: Heading 3

Formatted: Underline

Zhengkui Zhou, Huifang Li, Fangxi Yang, and Yong He, Zhonghua Ning, and Ning Yang.

References

1. Li J and Zhang Y. Advances in research of the origin and domestication of domestic animals. Biodiversity Science. 2009;17 4:319-29.
2. Darwin C and Mayr E. On the origin of species by means of natural selection, or the preservation of favoured races in the struggle for life. john murray, london. On the Origin of Species by Means of Natural Selection. 1859.
3. Chen C, Liu Z, Pan Q, Chen X, Wang H, Guo H, et al. Genomic Analyses Reveal Demographic History and Temperate Adaptation of the Newly Discovered Honey Bee Subspecies Apis mellifera sinisxinyuan n. ssp. Mol Biol Evol. 2016;33 5:1337-48. doi:10.1093/molbev/msw017.
4. Yang J, Li WR, Lv FH, He SG, Tian SL, Peng WF, et al. Whole-Genome Sequencing of Native Sheep Provides Insights into Rapid Adaptations to Extreme Environments. Mol Biol Evol. 2016;33 10:2576-92. doi:10.1093/molbev/msw129.
5. Li M, Tian S, Jin L, Zhou G, Li Y, Zhang Y, et al. Genomic analyses identify distinct patterns of selection in domesticated pigs and Tibetan wild boars. Nat Genet. 2013;45 12:1431-8. doi:10.1038/ng.2811.
6. Jiang Y, Xie M, Chen W, Talbot R, Maddox JF, Faraut T, et al. The sheep genome illuminates biology of the rumen and lipid metabolism. Science. 2014;344 6188:1168-73. doi:10.1126/science.1252806.
7. Carneiro M, Rubin CJ, Di Palma F, Albert FW, Alfoldi J, Barrio AM, et al. Rabbit genome analysis reveals a polygenic basis for phenotypic change during domestication. Science. 2014;345 6200:1074-9. doi:10.1126/science.1253714.
8. Wang MS, Zhang RW, Su LY, Li Y, Peng MS, Liu HQ, et al. Positive selection rather than relaxation of functional constraint drives the evolution of vision during chicken domestication. Cell Res. 2016;26 5:556-73. doi:10.1038/cr.2016.44.
9. Rubin CJ, Zody MC, Eriksson J, Meadows JR, Sherwood E, Webster MT, et al. Whole-genome resequencing reveals loci under selection during chicken domestication. Nature. 2010;464 7288:587-91. doi:10.1038/nature08832.
10. Huang Y, Li Y, Burt DW, Chen H, Zhang Y, Qian W, et al. The duck genome and transcriptome provide insight into an avian influenza virus reservoir species. Nat Genet. 2013;45 7:776-83.

doi:10.1038/ng.2657.

11. Zeuner FE. A history of domesticated animals. A history of domesticated animals. 1963.

12. Thomson SAL, Ornithologists' Union B and Thomson AL. A new dictionary of birds. Nelson London; 1964.

13. Mason IL and Mason IL. Evolution of domesticated animals. 1984.

14. Bray F and Needham J. Science and Civilization in China, vol. 6, part 1. Agriculture: Cambridge University Press, Cambridge, UK. 1984.

15. Luff R. 2000. Ducks. In Cambridge World History of Food, ed. KF Kiple, KC Ornelas, pp. 517–24. Cambridge, UK: Cambridge University PressKiple KF. The Cambridge world history of food. Cambridge: Cambridge University Press; 2000.

16. Chang H. Conspectus of genetic resources of livestock. Chinese Agriculture Press, Beijing, China, 1995.

17. Miller DB. Social displays of Mallard Ducks (Anas platyrhynchos): effects of domestication. Journal of Comparative and Physiological Psychology. 1977;91 2:221.

18. Ebinger P. Domestication and plasticity of brain organization in mallards (Anas platyrhynchos). Brain, behavior and evolution. 1995;45 5:286-300.

19. Frahm H, Rehkämper G and Werner C. Brain alterations in crested versus non-crested breeds of domestic ducks (Anas platyrhynchos fd). Poultry science. 2001;80 9:1249-57.

20. Duggan BM, Hocking PM, Schwarz T and Clements DN. Differences in hindlimb morphology of ducks and chickens: effects of domestication and selection. Genetics Selection Evolution. 2015;47 1:88.

21. Tang H, Peng J, Wang P and Risch NJ. Estimation of individual admixture: analytical and study design considerations. Genet Epidemiol. 2005;28 4:289-301. doi:10.1002/gepi.20064.

22. Li H and Durbin R. Inference of human population history from individual whole-genome sequences. Nature. 2011;475 7357:493-6. doi:10.1038/nature10231.

23. Ehlers J and Gibbard PL. The extent and chronology of Cenozoic global glaciation. Quaternary International. 2007;164:6-20.

24. Williams MAJ, Dunkerley D, De Deckker P, Kershaw AP and Stokes T. Quaternary environments. Science Press; 1997.

25. Nadachowska-Brzyska K, Li C, Smeds L, Zhang G and Ellegren H. Temporal Dynamics of Avian Populations during Pleistocene Revealed by Whole-Genome Sequences. Curr Biol. 2015;25 10:1375-80. doi:10.1016/j.cub.2015.03.047.

26. Shapiro MD, Kronenberg Z, Li C, Domyan ET, Pan H, Campbell M, et al. Genomic diversity and evolution of the head crest in the rock pigeon. Science. 2013;339 6123:1063-7. doi:10.1126/science.1230422.

27. Price TD. Domesticated birds as a model for the genetics of speciation by sexual selection. Genetica. 2002;116 2-3:311-27. doi:Doi 10.1023/A:1021248913179.

28. Lorenzen ED, Nogués-Bravo D, Orlando L, Weinstock J, Binladen J, Marske KA, et al. Species-specific responses of Late Quaternary megafauna to climate and humans. Nature. 2011;479 7373:359-64.

29. Hewitt G. The genetic legacy of the Quaternary ice ages. Nature. 2000;405 6789:907-13.

30. Hewitt G. Genetic consequences of climatic oscillations in the Quaternary. Philosophical Transactions of the Royal Society of London B: Biological Sciences. 2004;359 1442:183-95.

31. Qiu Q, Wang L, Wang K, Yang Y, Ma T, Wang Z, et al. Yak whole-genome resequencing reveals domestication signatures and prehistoric population expansions. Nature communications. 2015;6:10283. doi:10.1038/ncomms10283.

32. Orlando L, Ginolhac A, Zhang G, Froese D, Albrechtsen A, Stiller M, et al. Recalibrating Equus evolution using the genome sequence of an early Middle Pleistocene horse. Nature. 2013;499 7456:74-8. doi:10.1038/nature12323.

33. Steingrimsson E, Copeland NG and Jenkins NA. Melanocytes and the microphthalmia transcription factor network. Annual review of genetics. 2004;38:365-411. doi:10.1146/annurev.genet.38.072902.092717.

34. Hallsson JH, Haflidadottir BS, Schepsky A, Arnheiter H and Steingrimsson E. Evolutionary sequence comparison of the Mitf gene reveals novel conserved domains. Pigment cell research. 2007;20 3:185-200. doi:10.1111/j.1600-0749.2007.00373.x.

35. Levy C, Khaled M and Fisher DE. MITF: master regulator of melanocyte development and melanoma oncogene. Trends in molecular medicine. 2006;12 9:406-14. doi:10.1016/j.molmed.2006.07.008.

36. Minvielle F, Bed'hom B, Coville JL, Ito S, Inoue-Murayama M and Gourichon D. The "silver" Japanese quail and the MITF gene: causal mutation, associated traits and homology with the "blue" chicken plumage. BMC genetics. 2010;11:15. doi:10.1186/1471-2156-11-15.

37. Karlsson EK, Baranowska I, Wade CM, Salmon Hillbertz NH, Zody MC, Anderson N, et al. Efficient mapping of mendelian traits in dogs through genome-wide association. Nat Genet. 2007;39 11:1321-8. doi:10.1038/ng.2007.10.

38. Li S, Wang C, Yu W, Zhao S and Gong Y. Identification of genes related to white and black plumage formation by RNA-Seq from white and black feather bulbs in ducks. PLoS One. 2012;7 5:e36592. doi:10.1371/journal.pone.0036592.

39. Sultana H, Seo D, Choi NR, Bhuiyan MSA, Lee SH, Heo KN,

et al. Identification of Polymorphisms in MITF and DCT Genes and their Associations with Plumage Colors in Asian Duck Breeds. Asian-Australasian journal of animal sciences. 2017; doi:10.5713/ajas.17.0298.

40. Eriksson M, Nilsson A, Samuelsson H, Samuelsson EB, Mo L, Akesson E, et al. On the role of NR3A in human NMDA receptors. Physiology & behavior. 2007;92 1-2:54-9. doi:10.1016/j.physbeh.2007.05.026.

41. Bauer PH, Muller S, Puzicha M, Pippig S, Obermaier B, Helmreich EJM, et al. Phosducin Is a Protein Kinase-a-Regulated G-Protein Regulator. Nature. 1992;358 6381:73-6. doi:Doi 10.1038/358073a0.

42. Sunayashiki-Kusuzaki K, Kikuchi T, Wawrousek EF and Shinohara T. Arrestin and phosducin are expressed in a small number of brain cells. Brain research Molecular brain research. 1997;52 1:112-20.

43. Mignon-Grasteau S, Boissy A, Bouix J, Faure J-M, Fisher AD, Hinch GN, et al. Genetics of adaptation and domestication in livestock. Livestock Production Science. 2005;93 1:3-14.

44. Dugatkin LA and Trut L. How to Tame a Fox (and Build a Dog): Visionary Scientists and a Siberian Tale of Jump-Started Evolution. University of Chicago Press; 2017.

45. Axelsson E, Ratnakumar A, Arendt ML, Maqbool K, Webster MT, Perloski M, et al. The genomic signature of dog domestication reveals adaptation to a starch-rich diet. Nature. 2013;495 7441:360-4. doi:10.1038/nature11837.

46. Volinia S, Dhand R, Vanhaesebroeck B, MacDougall L, Stein R, Zvelebil M, et al. A human phosphatidylinositol 3-kinase complex related to the yeast Vps34p-Vps15p protein sorting system. The EMBO Journal. 1995;14 14:3339.

47. Inaguma Y, Ito H, Iwamoto I, Matsumoto A, Yamagata T, Tabata H, et al. Morphological characterization of Class III phosphoinositide 3-kinase during mouse brain development. Medical molecular morphology. 2016;49 1:28-33. doi:10.1007/s00795-015-0116-1.

48. Stopkova P, Saito T, Papolos DF, Vevera J, Paclt I, Zukov I, et al. Identification of PIK3C3 promoter variant associated with bipolar disorder and schizophrenia. Biol Psychiatry. 2004;55 10:981-8. doi:10.1016/j.biopsych.2004.01.014.

49. Tang R, Zhao X, Fang C, Tang W, Huang K, Wang L, et al. Investigation of variants in the promoter region of PIK3C3 in schizophrenia. Neuroscience letters. 2008;437 1:42-4. doi:10.1016/j.neulet.2008.03.043.

50. Zhou X, Wang L, Hasegawa H, Amin P, Han B-X, Kaneko S, et al. Deletion of PIK3C3/Vps34 in sensory neurons causes rapid neurodegeneration by disrupting the endosomal but not the autophagic

pathway. Proceedings of the National Academy of Sciences. 2010;107 20:9424-9.

51. Wilson PA, Gardner SD, Lambie NM, Commans SA and Crowther DJ. Characterization of the human patatin-like phospholipase family. Journal of lipid research. 2006;47 9:1940-9.

52. Kienesberger PC, Oberer M, Lass A and Zechner R. Mammalian patatin domain containing proteins: a family with diverse lipolytic activities involved in multiple biological functions. Journal of lipid research. 2009;50 Supplement:S63-S8.

53. Tesson C, Nawara M, Salih MA, Rossignol R, Zaki MS, Al Balwi M, et al. Alteration of fatty-acid-metabolizing enzymes affects mitochondrial form and function in hereditary spastic paraplegia. The American Journal of Human Genetics. 2012;91 6:1051-64.

54. Schuurs-Hoeijmakers JH, Oh EC, Vissers LE, Swinkels ME, Gilissen C, Willemsen MA, et al. Recurrent de novo mutations in PACS1 cause defective cranial-neural-crest migration and define a recognizable intellectual-disability syndrome. The American Journal of Human Genetics. 2012;91 6:1122-7.

55. Martin E, Schüle R, Smets K, Rastetter A, Boukhris A, Loureiro JL, et al. Loss of function of glucocerebrosidase GBA2 is responsible for motor neuron defects in hereditary spastic paraplegia. The American Journal of Human Genetics. 2013;92 2:238-44.

56. Gagliano SA, Tiwari AK, Freeman N, Lieberman JA, Meltzer HY, Kennedy JL, et al. Protein kinase cAMP-dependent regulatory type II beta (PRKAR2B) gene variants in antipsychotic-induced weight gain. Human psychopharmacology. 2014;29 4:330-5. doi:10.1002/hup.2407.

57. Czyzyk TA, Sikorski MA, Yang L and McKnight GS. Disruption of the RIIβ subunit of PKA reverses the obesity syndrome of agouti lethal yellow mice. Proceedings of the National Academy of Sciences. 2008;105 1:276-81.

58. Resources CNCoAG. Animal genetic resources in China poultry. Beijing: China Agriculture Press; 2010.

59. Patel RK and Jain M. NGS QC Toolkit: a toolkit for quality control of next generation sequencing data. PLoS One. 2012;7 2:e30619. doi:10.1371/journal.pone.0030619.

60. McKenna A, Hanna M, Banks E, Sivachenko A, Cibulskis K, Kernytsky A, et al. The Genome Analysis Toolkit: a MapReduce framework for analyzing next-generation DNA sequencing data. Genome Res. 2010;20 9:1297-303. doi:10.1101/gr.107524.110.

61. DePristo MA, Banks E, Poplin R, Garimella KV, Maguire JR, Hartl C, et al. A framework for variation discovery and genotyping using next-generation DNA sequencing data. Nat Genet. 2011;43 5:491-8. doi:10.1038/ng.806.

62. Yan Y, Yi G, Sun C, Qu L and Yang N. Genome-wide

characterization of insertion and deletion variation in chicken using next generation sequencing. PLoS One. 2014;9 8:e104652. doi:10.1371/journal.pone.0104652.

63. Qu Y, Tian S, Han N, Zhao H, Gao B, Fu J, et al. Genetic responses to seasonal variation in altitudinal stress: whole-genome resequencing of great tit in eastern Himalayas. Sci Rep. 2015;5:14256. doi:10.1038/srep14256.

64. Meyer RS, Choi JY, Sanches M, Plessis A, Flowers JM, Amas J, et al. Domestication history and geographical adaptation inferred from a SNP map of African rice. Nat Genet. 2016;48 9:1083-8. doi:10.1038/ng.3633.

65. Russell J, Mascher M, Dawson IK, Kyriakidis S, Calixto C, Freund F, et al. Exome sequencing of geographically diverse barley landraces and wild relatives gives insights into environmental adaptation. Nat Genet. 2016;48 9:1024-30. doi:10.1038/ng.3612.

66. Mascher M, Schuenemann VJ, Davidovich U, Marom N, Himmelbach A, Hubner S, et al. Genomic analysis of 6,000-year-old cultivated grain illuminates the domestication history of barley. Nat Genet. 2016;48 9:1089-93. doi:10.1038/ng.3611.

67. Li H, Ruan J and Durbin R. Mapping short DNA sequencing reads and calling variants using mapping quality scores. Genome Res. 2008;18 11:1851-8. doi:10.1101/gr.078212.108.

68. Cingolani P, Platts A, Wang LL, Coon M, Nguyen T, Wang L, et al. A program for annotating and predicting the effects of single nucleotide polymorphisms, SnpEff: SNPs in the genome of Drosophila melanogaster strain w1118; iso-2; iso-3. Fly. 2012;6 2:80-92.

69. Zhang Z, Nie C, Jia Y, Jiang R, Xia H, Lv X, et al. Parallel Evolution of Polydactyly Traits in Chinese and European Chickens. PloS one. 2016;11 2:e0149010.

70. Purcell S, Neale B, Todd-Brown K, Thomas L, Ferreira MA, Bender D, et al. PLINK: a tool set for whole-genome association and population-based linkage analyses. The American Journal of Human Genetics. 2007;81 3:559-75.

71. Chang CC, Chow CC, Tellier LC, Vattikuti S, Purcell SM and Lee JJ. Second-generation PLINK: rising to the challenge of larger and richer datasets. Gigascience. 2015;4 1:7.

72. Yang J, Lee SH, Goddard ME and Visscher PM. GCTA: a tool for genome-wide complex trait analysis. The American Journal of Human Genetics. 2011;88 1:76-82.

73. Edgar RC. MUSCLE: multiple sequence alignment with high accuracy and high throughput. Nucleic acids research. 2004;32 5:1792-7.

74. Plotree D and Plotgram D. PHYLIP-phylogeny inference package (version 3.2). cladistics. 1989;5 163:6.

75. Tamura K, Dudley J, Nei M and Kumar S. MEGA4: molecular evolutionary genetics analysis (MEGA) software version 4.0. Molecular biology and evolution. 2007;24 8:1596-9.

76. Kumar S, Stecher G and Tamura K. MEGA7: Molecular Evolutionary Genetics Analysis Version 7.0 for Bigger Datasets. Mol Biol Evol. 2016;33 7:1870-4. doi:10.1093/molbev/msw054.

77. Lee TH, Guo H, Wang X, Kim C and Paterson AH. SNPhylo: a pipeline to construct a phylogenetic tree from huge SNP data. BMC genomics. 2014;15 1:162. doi:10.1186/1471-2164-15-162.

78. Zhan X, Pan S, Wang J, Dixon A, He J, Muller MG, et al. Peregrine and saker falcon genome sequences provide insights into evolution of a predatory lifestyle. Nat Genet. 2013;45 5:563-6. doi:10.1038/ng.2588.

79. Balakrishnan CN and Edwards SV. Nucleotide variation, linkage disequilibrium and founder-facilitated speciation in wild populations of the zebra finch (Taeniopygia guttata). Genetics. 2009;181 2:645-60.

80. Nam K, Mugal C, Nabholz B, Schielzeth H, Wolf JB, Backström N, et al. Molecular evolution of genes in avian genomes. Genome biology. 2010;11 6:R68.

81. Gutenkunst RN, Hernandez RD, Williamson SH and Bustamante CD. Inferring the joint demographic history of multiple populations from multidimensional SNP frequency data. PLoS genetics. 2009;5 10:e1000695. doi:10.1371/journal.pgen.1000695.

82. Coffman AJ, Hsieh PH, Gravel S and Gutenkunst RN. Computationally Efficient Composite Likelihood Statistics for Demographic Inference. Molecular Biology and Evolution. 2016;33 2:591-3. doi:10.1093/molbev/msv255.

83. Weir BS and Cockerham CC. Estimating F-Statistics for the Analysis of Population-Structure. Evolution. 1984;38 6:1358-70. doi:Doi 10.2307/2408641.

84. Kreyszig E. Advanced engineering mathematics. John Wiley & Sons; 2007.

85. Danecek P, Auton A, Abecasis G, Albers CA, Banks E, DePristo MA, et al. The variant call format and VCFtools. Bioinformatics. 2011;27 15:2156-8.

86. Tajima F. Evolutionary relationship of DNA sequences in finite populations. Genetics. 1983;105 2:437-60.

87. Huang da W, Sherman BT and Lempicki RA. Systematic and integrative analysis of large gene lists using DAVID bioinformatics resources. Nature protocols. 2009;4 1:44-57. doi:10.1038/nprot.2008.211.

88. Dobin A, Davis CA, Schlesinger F, Drenkow J, Zaleski C, Jha S, et al. STAR: ultrafast universal RNA-seq aligner. Bioinformatics.

2013;29 1:15-21.

89. Liao Y, Smyth GK and Shi W. The Subread aligner: fast, accurate and scalable read mapping by seed-and-vote. Nucleic acids research. 2013;41 10:e108-e.

90. Liao Y, Smyth GK and Shi W. featureCounts: an efficient general purpose program for assigning sequence reads to genomic features. Bioinformatics. 2014;30 7:923-30. doi:10.1093/bioinformatics/btt656.

91. Robinson MD and Smyth GK. Moderated statistical tests for assessing differences in tag abundance. Bioinformatics. 2007;23 21:2881-7. doi:10.1093/bioinformatics/btm453.

92. Robinson MD, McCarthy DJ and Smyth GK. edgeR: a Bioconductor package for differential expression analysis of digital gene expression data. Bioinformatics. 2010;26 1:139-40. doi:10.1093/bioinformatics/btp616.

93. McCarthy DJ, Chen Y and Smyth GK. Differential expression analysis of multifactor RNA-Seq experiments with respect to biological variation. Nucleic acids research. 2012;40 10:4288-97. doi:10.1093/nar/gks042.

94. Lun AT, Chen Y and Smyth GK. It's DE-licious: a recipe for differential expression analyses of RNA-seq experiments using quasi-likelihood methods in edgeR. Statistical Genomics: Methods and Protocols. 2016:391-416.

Figure 1

Click here to download Figure Fig. 1.pdf

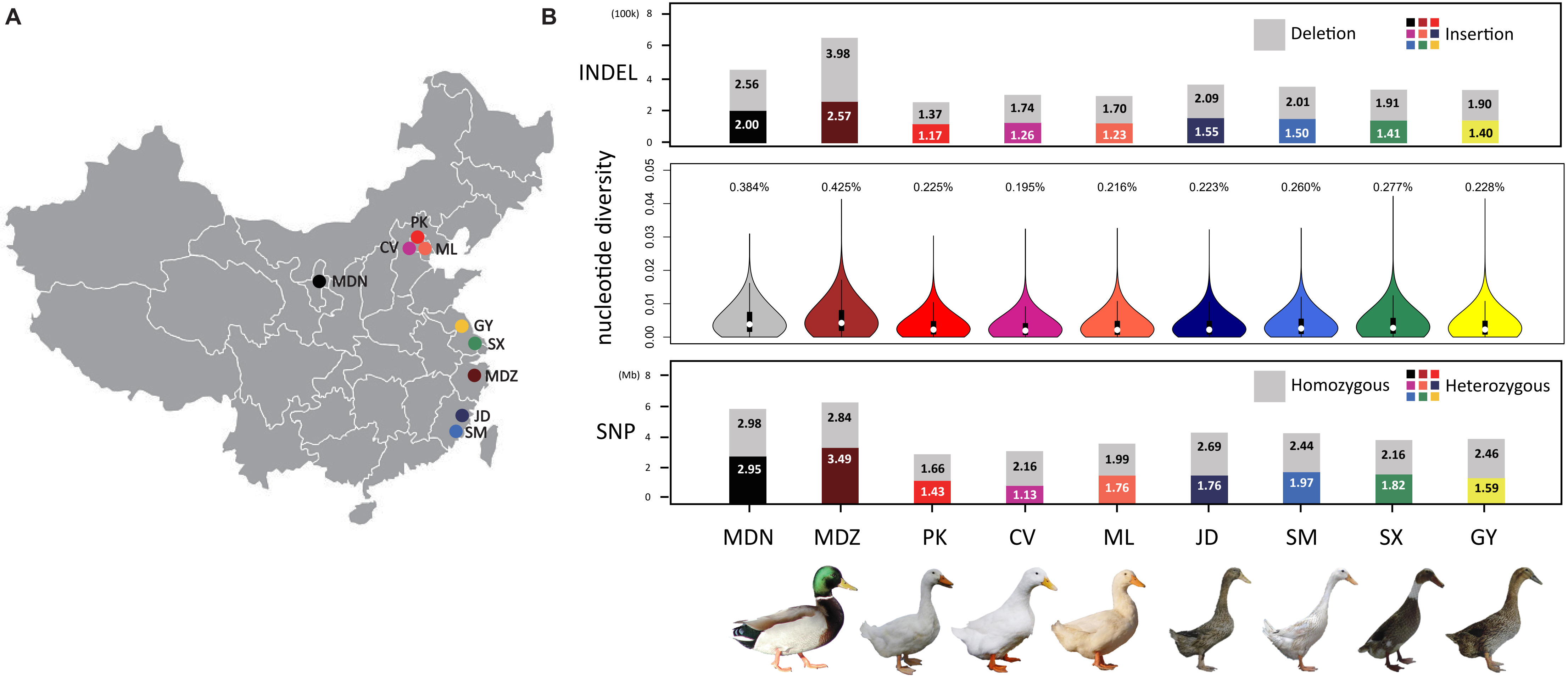

Figure 2

Click here to download Figure Fig. 2.pdf

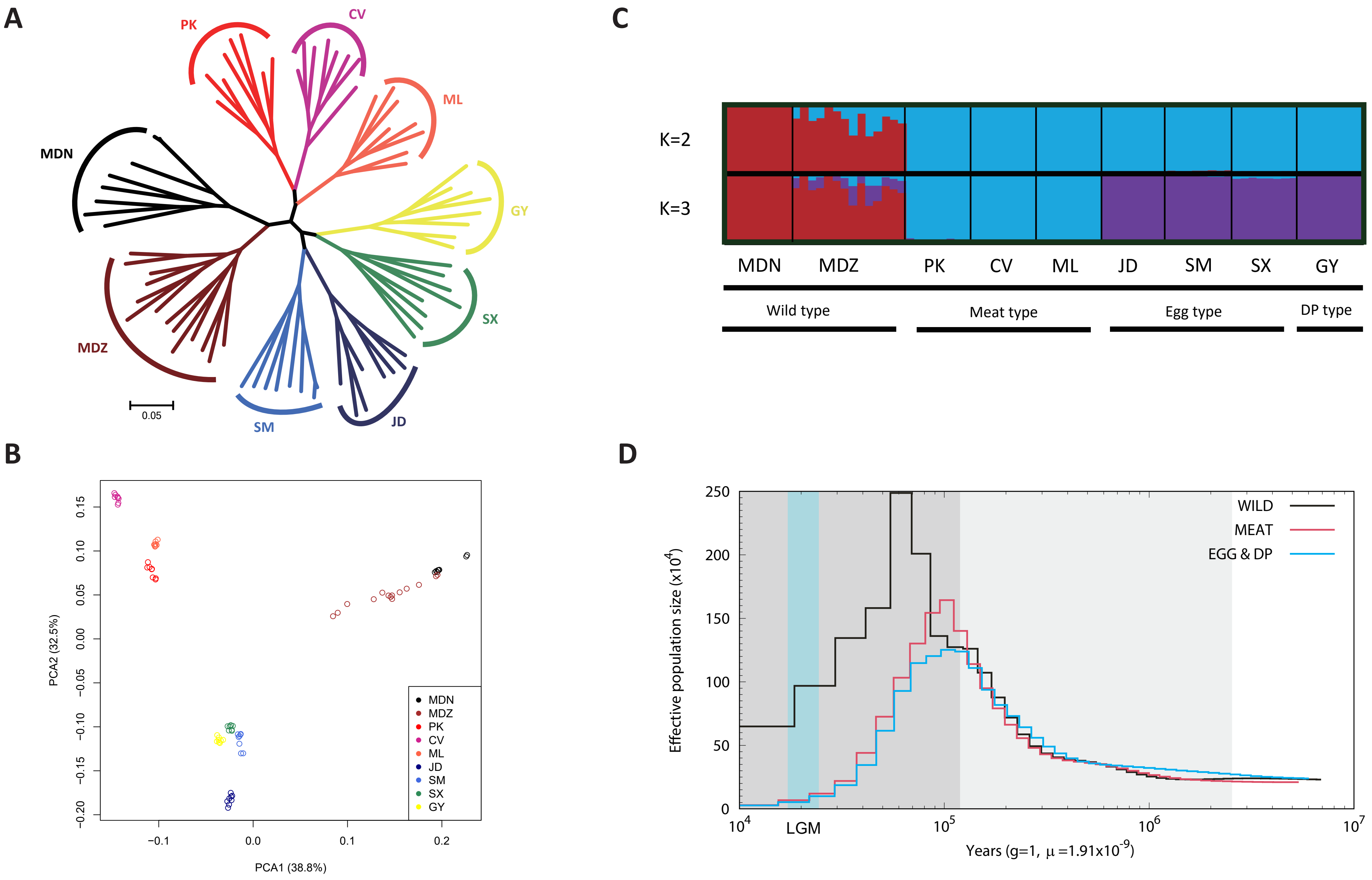

Figure 3

Click here to download Figure Fig. 3.pdf

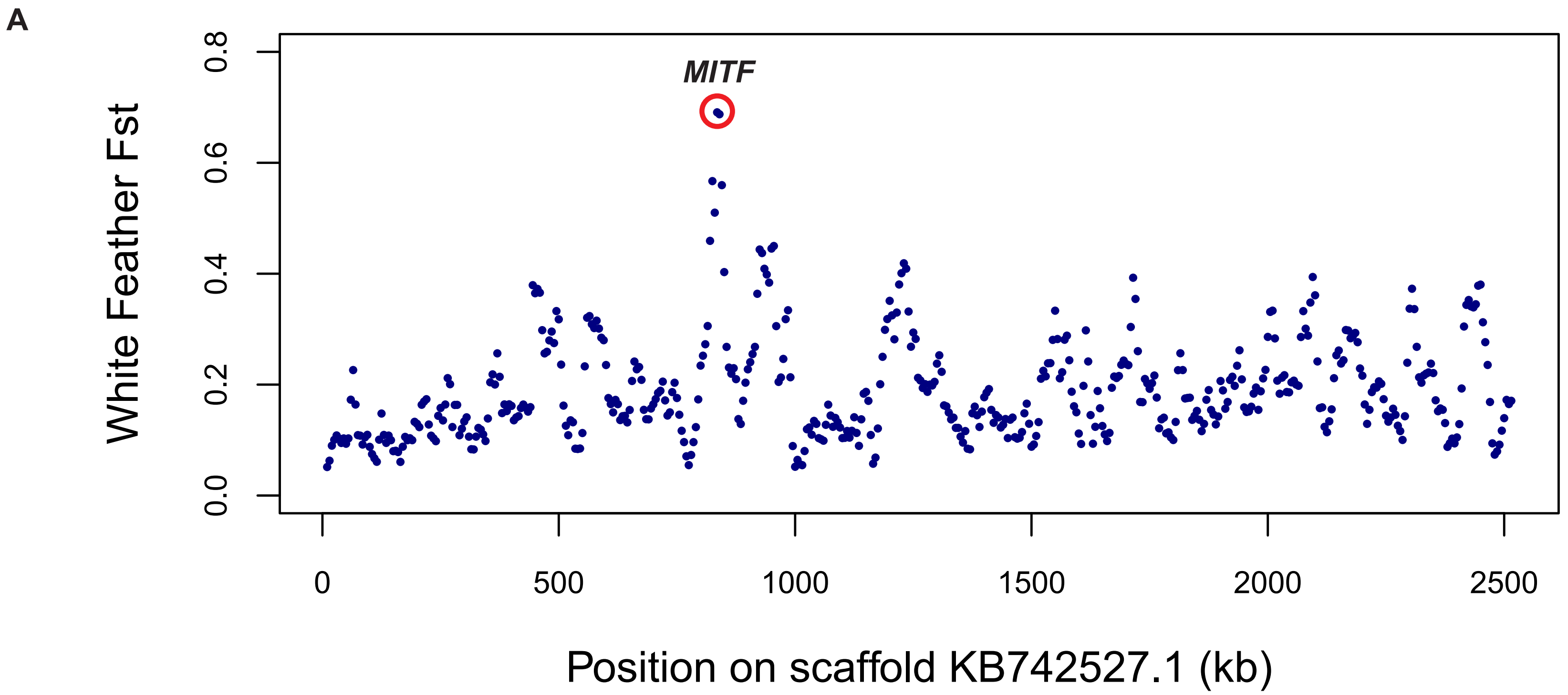

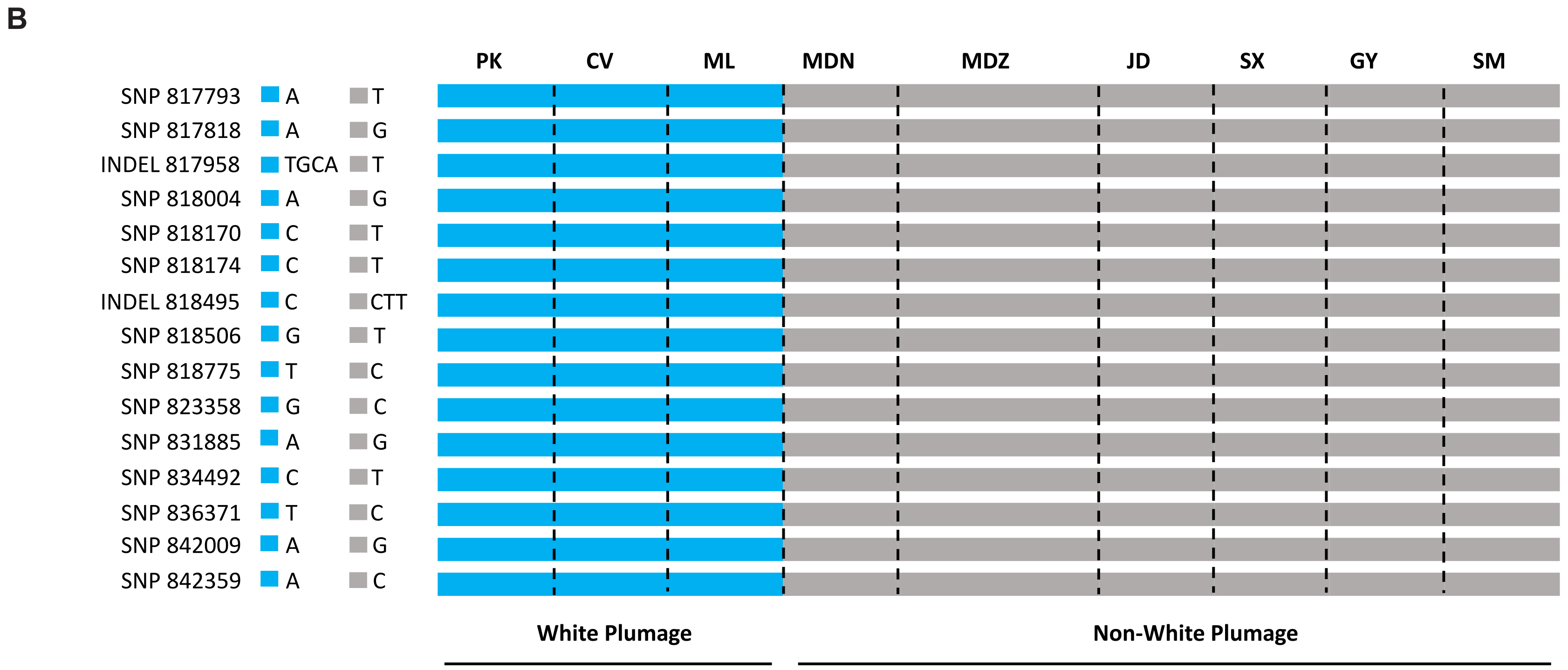

Figure 4

Click here to download Figure fig. 4.pdf

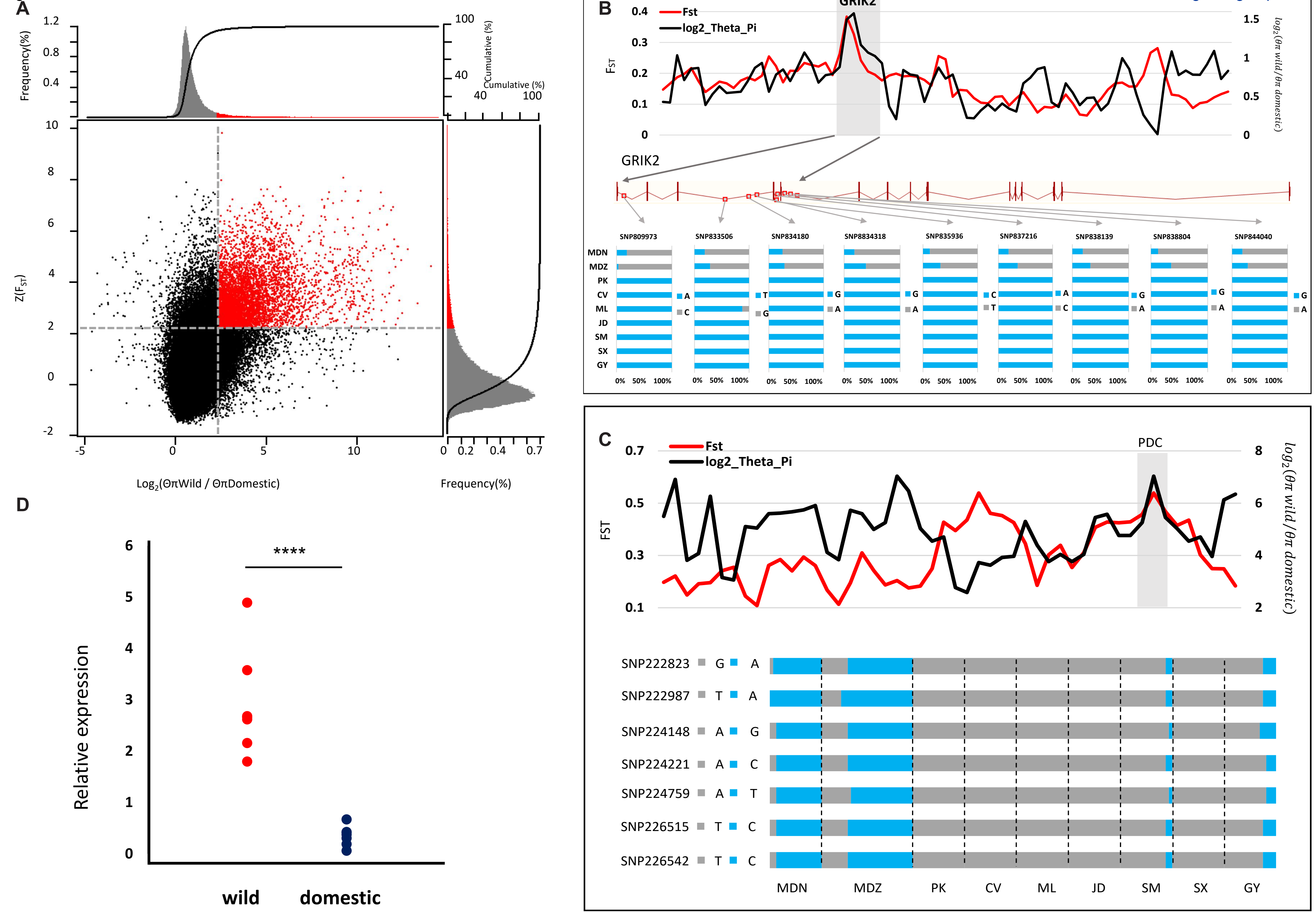

Supplementary Figure S1

Click here to access/download
Supplementary Material
supplemental Figure S1.pdf

Supplementary Figure S2

Click here to access/download
Supplementary Material
supplemental Figure S2.pdf

Supplementary Figure S3

Click here to access/download
Supplementary Material
Supplemental Figure S3.pdf

Supplementary Figure S4

Click here to access/download
Supplementary Material
Supplemental Figure S4.pdf

Supplementary Figure S5

Click here to access/download
Supplementary Material
Supplemental Figure S5.pdf

Supplementary Figure S6

Click here to access/download
Supplementary Material
Supplemental Figure S6.pdf

Supplementary Tables

Click here to access/download
Supplementary Material
Supplementary Tables.xlsx

Click here to download Personal Cover Reponse to Editor.docx

Dear Dr Zauner,

Many thanks for your positive comments about our manuscript, "Whole-genome resequencing reveals signatures of selection and timing of duck domestication" (manuscript number GIGA-D-17-00301). We also thank the reviewers for their thoughtful and constructive suggestions. We have addressed all these comments, detailed below, in our revised manuscript, which we hope is now suitable for publication in GigaScience.

Sincerely,

Lujiang Qu, Ph.D., on behalf of all co-authors.
Email: quluj@163.com
Department of Animal Genetics and Breeding, College of Animal Science and Technology, China Agricultural University, Beijing 100193, China

GIGA-D-17-00301
Whole-genome resequencing reveals signatures of selection and timing of duck domestication
Zebin Zhang; Yaxiong Jia; Pedro Almeida; Judith E Mank; Marcel van Tuinen; Qiong Wang; Zhihua Jiang; Yu Chen; Kai Zhan; Shuisheng Hou; Zhengkui Zhou; Huifang Li; Fangxi Yang; Yong He; Lujiang Qu, Ph.D.
GigaScience

Dear Prof. Qu,

Your manuscript "Whole-genome resequencing reveals signatures of selection and timing of duck domestication" (GIGA-D-17-00301) has been assessed by our reviewers. Although it is of interest, we are unable to consider it for publication in its current form. The reviewers have raised a number of points which we believe would improve the manuscript and may allow a revised version to be published in GigaScience.

Their reports are below.

Comment: All reviewers, but reviewer 2 in particular, provide some suggestions how the submission can be improved, for example by explaining the hypotheses more clearly in the introduction, and also by some additional analyses that may make the paper even stronger.

Reply: Many thanks for your comments. We have more clearly articulated our hypotheses in introduction section according to your and reviewer2's suggestion, please see lines 75-79. Meanwhile, we have done the additional analyses according to your and reviewer2's suggestion, such as FRAPPE analyses by K=4, PSMC and δaδi analyses based on chicken mutation rate, global F_{ST} between each duck population, and F_{ST} recalculated by BayeScan, please see the specific reply to reviewer2.

Comment: An absolutely crucial point for publication in GigaScience is the remark #6 by reviewer 1, regarding sharing of data, code and protocols. GigaScience embraces the FAIR principles (https://www.force11.org/group/fairgroup/fairprinciples) and we ask our authors to document their work according to these principles, to allow full reproducibility and maximum reuse potential of the data, protocols and scripts.

Please include supporting data such as custom scripts, full population genetic statistics and location of sweeps, any software output files, alignments, phylogenetic tree files etc.

Reply: Thank you for this suggestion. The 78 ducks used in our whole genome resequencing analysis and the 14 ducks used in RNA-seq analysis have been submitted to NCBI BioProject (http://www.ncbi.nlm.nih.gov/bioproject) under accession numbers PRJNA419832 and PRJNA419583, respectively. The unassembled sequencing

reads of 78 ducks and RNA-seq reads of 14 ducks have been deposited in NCBI Sequence Read Archive (SRA: http://www.ncbi.nlm.nih.gov/sra) under accession numbers SRP125660 and SRP125529, respectively.

VCF files of SNPs and INDELs, as well as other supporting data, have been submitted to *Giga*DB as suggested. Please check the *Giga*DB servers.

Meanwhile, we also replied to reviewer 1 and have added these description to our current manuscript, please see lines 618-628.

To share your supporting data and scripts, our data curators will be able to help you to make them available via our data repository GigaDB. You can contact them via email: database@gigasciencejournal.com.

We are encouraging our submitters to make use of protocols.io , if you provide your methods (both wet-lab and dry-lab) in the SOP tab on the data spreadsheet we can import those into protocols.io on your behalf.

To share your raw sequencing data, please note that the BIG data repository is not part of the International Nucleotide Sequence Database Collaboration. Please choose a database that is an INSDC member (http://www.insdc.org/) and report accession numbers of the INSDC database in the manuscript.

If you are able to fully address points of our reviewers, we would encourage you to submit a revised manuscript to GigaScience. Once you have made the necessary corrections, please submit online at:

http://giga.edmgr.com/

If you have forgotten your username or password please use the "Send Login Details" link to get your login information. For security reasons, your password will be reset.

Please include a point-by-point within the 'Response to Reviewers' box in the submission system.
Please ensure you describe additional experiments that were carried out and include a detailed rebuttal of any criticisms or requested revisions that you disagreed with.
Please also ensure that your revised manuscript conforms to the journal style, which can be found in the Instructions for Authors on the journal homepage.

The due date for submitting the revised version of your article is 20 Mar 2018.

I look forward to receiving your revised manuscript soon.

Best wishes,

Hans Zauner
GigaScience
www.gigasciencejournal.com

Reviewer reports:

Reviewer #1:
This paper reports sequencing, population history inferences, and selective sweep mapping in ducks using whole genome sequence data of multiple populations.

This is a good paper. It presents a large-scale population genomic dataset of ducks, uses standard methods that seem appropriate to the task, and it is well written.

Despite this, I have a few criticisms and questions:

Comment: **1. The paper repeatedly states that this is the first time MITF is associated with colour in the duck. This seems not to be entirely true (see Li et al 2012, http://journals.plos.org/plosone/article?id=10.1371/journal.pone.0036592, and Sultana et al 2017, https://www.ncbi.nlm.nih.gov/pubmed/28823136, but maybe the latter was not published when the manuscript was written). This study presents a whole-genome scan, which should provide stronger evidence than candidate gene associations. Comparing to other papers would be interesting. Can that help filter the candidate variants?**

Reply: Thank you very much for your positive comments and for the two very helpful citations. Li *et al* (2012) identified that M isoform of *MITF* as expressed in black feather ducks, rather than white feather ducks or other colorful ducks. Sultana *et al* (2017) showed several SNPs and INDEL of *MITF* with different allele frequency in black and white ducks (table 2 - 5), but did not distinguish the correlation of *MITF* to white or other feather colors.
Due linkage effects, it is notoriously difficult to determine which variant is the real causative mutation of white plumage. Thus, we used the strictest variant filter criteria, namely those with fixed genotype differences in white and non-white ducks. We would very much like to implement the reviewer's suggestion of using the variants identified in these two previous studies, however the variants reported in Li *et al* (2012) and Sultana *et al* (2017) do not in fact pass our strict filter criteria.

We have however added these citations to our manuscript and revised the discussion accordingly (please see line 390). Most importantly, in order to distinguish our result from these previous studies, we revised our statement to say that "Our results show that white plumage in the duck is completely associated with selection at the *MITF* locus" in our current manuscript, please see line 42 and line 246-247.

Comment: **2. It would be useful to see the population history results put more into context. In the light of what is known about duck breed history, is it reasonable that meat and egg type ducks split 2100 years ago? In the Discussion, this number is said to be "compatible with previous written records from 500 BC". The reference is to a book with no page numbers given. Would it be possible to be more specific? Given convergence problems with alternative models, how sure are you that the balance between migration and split time is right? I will admit that I am not really the person to evaluate the pairwise sequential Markov coalescent and δaδi results.**

Reply: Many thanks for your comments. As we state in the manuscript, written records note domestic ducks in China as early as 500 BC. Due to the lack of archaeological evidence, we must focus on textual evidence, which indicates duck domestication occurred approximately 2,000 - 2,500 years ago. We have added these historical references regarding duck domestication to our current manuscript, please see lines 63-71, and have added page numbers to the book citations, and below, please see lines 697-700. Meanwhile, we also reran the PSMC and δaδi analyses based on the mutation rate estimate in chicken (1.91×10^{-9} per base per generation, Nam et al. 2010). The chicken is phylogenetically closer to the duck than zebra finch, the source of our previous mutation rate estimate (Jarvis et al. 2014), however the mutation rate estimates in both chicken and duck are qualitatively similar. As a result, our results are similar, and indicate duck domestication occurred 2228 (±441) years ago. We revised the PSMC and δaδi results of our current manuscript, please see Fig 2D, Table 1, and lines 204-219, 546-548.

It is true that the recent divergent time and the high level of diversity in both the domestic and wild populations makes it difficult to differentiate recent admixture from incomplete lineage sorting, however our genetic analysis is largely consistent with these written records, and does not indicate domestication much earlier than this time.

Luff R. 2000. Ducks. In *Cambridge World History of Food*, ed. KF Kiple, KC Ornelas, pp. 517–24. Cambridge, UK: Cambridge University Press
Jarvis, E. D., et al. (2014). "Whole-genome analyses resolve early branches in the tree of life of modern birds." Science 346(6215): 1320-1331.
Nam, K., et al. (2010). "Molecular evolution of genes in avian genomes." Genome Biol 11(6): R68.

Comment: **3. It is nice to see the high overlap between SNPs detected here and those in dbSNP. How many of the indels were already in databases? Was PCR validation only for SNPs? Given that indel detection is harder than SNP detection, are you convinced that the MITF indels are real?**

Reply: Thank you for your comments. Initially, we validated our INDELs in dbINDEL, following a similar protocol to our SNP validation. However, there has been less focus on INDEL annotation in the database, which contains nearly 70 fold fewer INDELs than

we detected. As we used extremely strict filter criteria for INDELs as well as SNPs, we suggest that the difference in variation is due to our greater focus on INDEL annotation please lines 497 – 500.

For the two MITF INDELs discussed, we used diagnostic PCR combined with Sanger sequencing to validate these sites in the 78 white and non-white ducks, as well as the first three SNPs (SNP817793, SNP817818, and SNP818004). The Sanger sequencing results of the three SNPs and INDEL817958 completely match our NGS analysis, please see figure below and supplemental figure S5 in our current manuscript. For INDEL818495, we were unable to identify a suitable PCR primer. We have added this to our revised manuscript, please see lines 247-253.

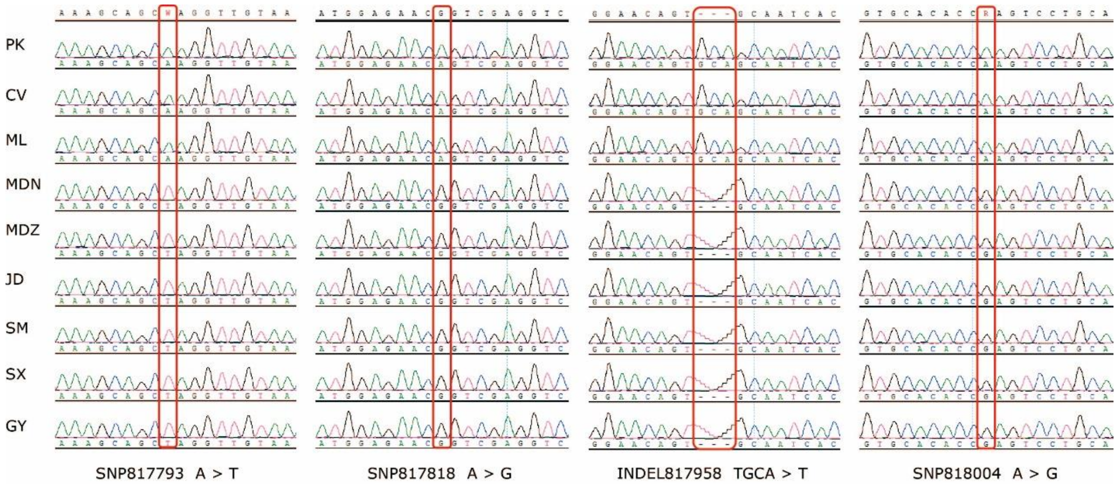

Comment: **4. A protocol for PCR validation seems to be missing (L440-442). It is hard to interpret the 100% accuracy in SNP validation when it is not clear how validation was performed or the accuracy evaluated.**

Reply: Apologies, and many thanks for pointing this out. The SNP validation was performed by diagnostic PCR combined with Sanger sequencing method. We have added this description to our revised manuscript, please see lines 510-513.

Comment: **5. The paper is well written, but the GigaScience author guidelines prescribe a somewhat different structure. It specifies an abstract divided into Background, Results, and Conclusions. The Data Description section is missing and other sections are have different names.**

Reply: Thank you very much for this helpful suggestion. We had separated the abstract section accordingly, please see lines 30-44. We have also added the Data Description section, please see lines 86-109. We also renamed the Results as Analyses, please see line 111, and revised the Availability of Supporting Data and Materials (lines 618-628), and the Declarations section (lines 632, 633, and 641).

Comment:6. **It seems to me that the data and source code availability may not be in line with the journal policies. I am not certain how to interpret the policies, but the**

editors will know better. Overall, the methods are described in text, but protocols and scripts are not provided. The raw sequence data is published in a repository, but little else, not even the full population genetic statistics or location of sweeps, as far as I can tell.

Reply: Apologies for our previous raw data and source code status. The data from the 78 ducks used in whole genome resequencing and the 14 ducks used in RNA-seq analysis have been submitted to NCBI BioProject (http://www.ncbi.nlm.nih.gov/bioproject) under accession numbers PRJNA419832 and PRJNA419583, respectively. The unassessembled sequencing reads of 78 ducks and RNA-seq reads of 14 ducks have been deposited in the NCBI Sequence Read Archive (SRA: http://www.ncbi.nlm.nih.gov/sra) under accession numbers SRP125660 and SRP125529, respectively. VCF files of SNPs and INDELs, as well as other supporting data, have been submitted to *Giga*DB as you suggest, please check the *Giga*DB servers. And, we add these description to our current manuscript, please see lines 618-628.

Minor comments

Comment: **Line 35: The important numbers are the number of individuals sampled and the coverage per individual. Average coverage per breed seems less interesting.**

Reply: Many thanks for your comment, we had revised this to per individual coverage information, please see line 36.

Comment: **Lines 97-101: What do the average numbers of variants detected per individual mean? Are they variants that differ from reference genome, heterozygous variants, or something else?**

Reply: Many thanks for your questions. The number of variants between the reference genome and each individual are different, especially in wild mallard and domesticated ducks, (please see supplementary table S2). The average value is the mean variant count of an individual, which includes both heterozygous variants and homozygous variants.

Comment: **Lines 243-250: Which GO terms were these, and how were they chosen? It seems odd to me to first select a subset of genes based on GO and then perform enrichment analysis on that set. Will this not bias the analysis?**

Reply: Apologies for any confusion. In fact, we observed 292 genes in the top 5% Fst regions, please see supplementary table S5. Our enrichment analysis is based on these 292 genes, and we identified a subset of GO terms for further analyses based on significant GO term P-values, please see supplementary table S7. Moreover, we add the full GO terms to our current manuscript, please see supplementary table S6.

Comment: **Lines 393-400: Is there a reason for this mix of sequencing coverage?**

Reply: We aimed to sequence each individual at 5X coverage. Additionally, in order to reduce the false negative rate of variants due to our strict filter criteria, we randomly selected one individual from each population for 10X coverage.

Comment: **Lines 381-384: It is not clear where the ducks came from. How were they obtained?**

Reply: Many thanks for your questions. PK and ML ducks were obtained from Institute of Pekin Duck with the help of Mr. Fangxi Yang, please see author information section, lines 5 and 25. CV ducks were obtained from Cherry Valley farms Co. Ltd with the help of Dr. Yong He, please see lines 5 and 26. The other domesticated ducks were obtained from different duck breeding farms under the help of Dr. Huifang Li, please see lines 5 and 23.

Comment: **Line 506: What tool was used for Fst? Also VCFtools?**

Reply: Thanks you very much for your questions. The Fst was calculated by the formula described by Weir BS (1984) under our custom perl script. Our custom perl script have been submitted to *Giga*DB database.

Weir, B. S. and C. C. Cockerham (1984). "Estimating F-Statistics for the Analysis of Population-Structure." Evolution **38**(6): 1358-1370.

Comment: **Figure 1b: The circos plot in Figure 1 looks impressive, but is impossible to read. What is it supposed to show?**

Reply: Apologies for any problems with our figures. The complicated circos plot is the result of the many scaffolds (78,488) in the current duck reference genome. We have removed the circos plot from our current manuscript, please see figure 1, and line 125-127 .

Comment: **Throughout methods: Version numbers are missing for some softwares.**

Reply: Apologies for this. We have added all this information to our current manuscript, such as NGS QC Toolkit v2.3.3 (line 480), SnpEff v4.0 (line 501), GCTA v1.25 (line 520), MUSCLE v3.8 (line 532), PSMC v0.6.5 (line 541), ∂a∂i v1.7 (line 550), VCFtools v0.1.13 (line 592), and edgeR v3.6 (line 617).

Reviewer #2:

Zhang et al. sequenced whole genomes of 78 individuals of domesticated and wild

mallard populations. The authors find a complex history of domestication, with particular artificial selection of meat and egg production in domesticated lineages. Further, outlier analyses demonstrate that white plumage was the result of selection of MITF transcriptional factors. I believe that the authors are tackling an important question regarding variation between domesticates and wild populations, and with an extensive genomic dataset. However, I think the authors fall short in introducing the subject and discussing their results. Moreover, the manuscript requires editing prior to publication, particularly the introduction.

Comment: Introduction.
The introduction requires extensive editing. I would also encourage the authors to add another sentence as the relevance (the why) of looking for outliers between domesticated and wild stocks. What exactly are you trying to learn? Instead of results, I would like to see hypotheses regarding what the authors may expect when comparing the genomes of domesticated and wild populations.

Reply: Many thanks for your comments. The most important reason we identified outliers between wild and domesticated ducks was to identify putative sites associates with the genetic basis of phenotypic differences between wild and domestic populations. We have added this explanation to our manuscript, and have also extensively revised our introduction section according to your suggestions, please see lines 51-85.

We had two primary hypotheses regarding duck domestication given the deep divergence between meat and egg breeds. Were ducks domesticated once from wild mallards and subsequently selected for separate egg and meat traits, or were egg and meat populations domesticated in two independent events. We have add the hypotheses of duck domestication scenarios to introduction section, please see lines 75-79.

Comment: The whole first paragraph requires editing.
For example -- Line 50-52: Suggest change sentence to: "Mallards (Anas platyrhynchos) are the world's most widely distributed and agriculturally important waterfowl species, and are especially of economic importance in Asia [1]."

Reply: Many thanks for this suggestion. We had revised the sentence accordingly, please see lines 63-64. And we have also extensively revised the first paragraph as suggested, please see lines 52-71.

Comment: Results

1. Line 79 - is this 535 billion mappable reads per sample or across samples?

Reply: Apologies for any confusion. The 535 billion is the total mapped reads across samples. We have added this explanation to our revised manuscript, please see line

117.

Comment: 2. Lines 115-121- how did the authors pick the optimum K in FRAPPE analyses? Did the authors explore additional K values? Where separate analyses done within wild and domesticated populations? Please explain.

Reply: Many thanks for your comments. We analyzed the population structure with K =2, 3 and 4 because there are four duck types across the nine duck populations, shown below, and explained in lines161-165. When K=4, a clear division was found between egg type ducks (JD, SM, and SX) and dual-purpose type ducks (GY) (supplemental figure S6). The most important reason we focused on K=3 as the optimum value for further analysis is due to the results of both the phylogenic and PCA analyses, which convergently showed the nine duck populations clustered into 3 major groups.

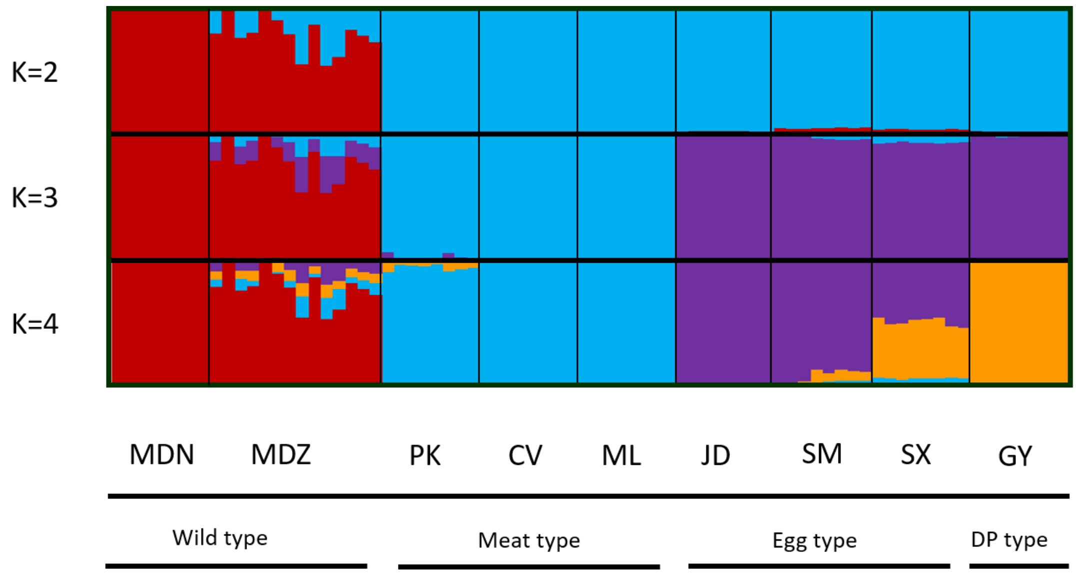

Comment: 2a. What do the authors make of domesticated admixture in wild populations? Is this hybridization, ancestry, a combination of both…? I would encourage the authors to explore this further as hybridization between domesticated and wild breeds is a serious concern for conservation of wild populations.

Reply: We agree with the reviewer that this is a very interesting area, and an area of great conservation importance. Unfortunately, given the recent domestication and high levels of diversity we observe, it is not in fact possible to accurately differentiate hybridization from incomplete lineage sorting with our current data, as complex models with these alternative scenarios failed to converge. We agree that this is an interesting area for further study, and have added this explanation to our current manuscript, please see lines 377-381.

Comment: 2b. The PCA analyses seem to suggest that there is structure within wild

populations. Running a FRAPPE analyses on wild populations could help tease out whether they are 1 population and PCA analyses are just separating samples as there is so much variation.

Reply: Thank you very much for your comments. Of course, the PCA result showed there is a structure within wild populations, because the two wild populations come from two different provinces in China separated by nearly 2,000 km, (please see line 446). However, the PCA result also showed extensive overlap of these two wild populations, please see fig 2B. Additionally, our FRAPPE analyses were based on all 78 duck individuals rather than pooled population information. Thus, we apologize if we have missed something intended by the reviewer, but we think the structural analysis suggested with recover the same result as our current analysis.

Comment: **3. Lines 139-141 - consider revising the sentence into a more formal hypothesis. I would also like to see such hypotheses in the introduction.**

Reply: Thank you so much for your kind suggestion. We had two primary hypotheses regarding duck domestication given the deep divergence between meat and egg breeds. Were ducks domesticated once from wild mallards and subsequently selected for separate egg and meat traits, or were egg and meat populations domesticated in two independent events. We have added the hypotheses of duck domestication scenarios to introduction section, please see lines 75-79.

Comment: **4. Outside of outlier tests by calculating FST, the authors should consider more formal testing of these putative outliers (e.g., BayeScan).**

Reply: Thank you very much for this suggestion. We have recalculated our F_{ST} with BayeScan, and the results are statistically similar to our current analysis, based on Weir, B. S. (1984). Thus, we have kept our previous F_{ST} method in our revised manuscript, as this method is a classical and formal method for calculating $F_{ST,}$ and has been widely implemented in many organisms, including rice (Meyer, R. S., et al. 2016), sheep (Yang, J., et al. 2016), dog (Gou, X., et al. 2014, Axelsson, E., et al. 2013), and pigeon (Shapiro, M. D., et al. 2013).

Weir, B. S. and C. C. Cockerham (1984). "Estimating F-Statistics for the Analysis of Population-Structure." Evolution 38(6): 1358-1370.
Meyer, R. S., et al. (2016). "Domestication history and geographical adaptation inferred from a SNP map of African rice." Nat Genet 48(9): 1083-1088.
Yang, J., et al. (2016). "Whole-Genome Sequencing of Native Sheep Provides Insights into Rapid Adaptations to Extreme Environments." Mol Biol Evol 33(10): 2576-2592.
Gou, X., et al. (2014). "Whole-genome sequencing of six dog breeds from continuous altitudes reveals adaptation to high-altitude hypoxia." Genome Res 24(8): 1308-1315.
Axelsson, E., et al. (2013). "The genomic signature of dog domestication reveals adaptation to a starch-rich diet." Nature 495(7441): 360-364.

Shapiro, M. D., et al. (2013). "Genomic diversity and evolution of the head crest in the rock pigeon." Science 339(6123): 1063-1067.

Comment: **5. Although I like the idea of RNA-seq data here. I think that this is largely overlooked in the manuscript and may detract from the main (genome) focus. I would encourage the authors to consider taking the RNA-seq out or sufficiently expanding on methods, reasoning, etc. of the RNA-seq data.**

Reply: Thank you so much for your suggestion. We respectfully suggest that the RNA-seq is a key component of our manuscript, as it represents functional phenotypic differentiation of wild mallards and domesticated ducks, and helps connect the genomic variation to phenotypic differences. We have revised the methods and reasoning of including this data RNA-seq as suggested, please see lines 324-328, 470-475, and 603-615.

Comment: **6. I would like to see global Fst estimates among breeds, wild locations**

Reply: Many thanks for your comment. The global F_{ST} between were showed in below, and we also add this table to our current manuscript, please see lines 267-268, and supplemental table S4.

	MDN	MDZ	PK	CV	ML	JD	SX	SM	GY
**MDN**	-	1.00E-01	2.73E-01	3.13E-01	2.68E-01	2.73E-01	2.13E-01	2.30E-01	2.68E-01
**MDZ**	-	-	1.97E-01	2.40E-01	1.88E-01	1.99E-01	1.32E-01	1.54E-01	1.90E-01
**PK**	-	-	-	2.23E-01	1.84E-01	2.84E-01	1.96E-01	2.32E-01	2.57E-01
**CV**	-	-	-	-	2.05E-01	3.41E-01	2.57E-01	2.90E-01	3.20E-01
**ML**	-	-	-	-	-	2.86E-01	2.07E-01	2.35E-01	2.72E-01
**JD**	-	-	-	-	-	-	1.71E-01	1.97E-01	2.63E-01
**SX**	-	-	-	-	-	-	-	1.27E-01	1.52E-01
**SM**	-	-	-	-	-	-	-	-	2.15E-01
**GY**	-	-	-	-	-	-	-	-	-

Comment: **Discussion**

I have no issues with the discussion and find it the best written. I think that a section on domesticate and wild hybridization may broaden the appeal of this paper.

Reply: Thanks for this suggestion. As we mentioned above, given the recent domestication and high levels of diversity we observe, it is not possible to accurately differentiate hybridization from incomplete lineage sorting with our current data, as complex models with these alternative scenarios failed to converge. We agree that this is an interesting area for further study, and have added material to the discussion as suggested, please see lines 377-381.

Comment: **Methods**

Please add additional information regarding FRAPPE analyses, K selection,etc.

Reply: Apologies for any omissions. We have added the method of FRAPPE analyses and K selection to our current manuscript, please see lines 523-529.

Comment: **Figures**
Figure 1: Consider re-moving statistical tests as these are presented in the results.

Reply: Thanks for your helpful comment. We have moved the statistical tests to the results section as suggested, please see lines 129-133, 144-147.

Reviewer #3:

Overall a very nice paper, detailed comments to the authors:

Comment: **Line 35: 45X coverage is misleading since the individual coverage was much smaller, please make a clearer statement here**

Reply: Thank you for this helpful suggestion. We have revised the population coverage information to individual information, please see line 36.

Comment: **L40: Our FST analysis also indicates for the first time ...**

Reply: Thanks for this suggestion. We have revised our manuscript according to your suggestion, please see lines 41-43.

Comment: **L52: of particular economic importance ...**

Reply: Many thanks for your comment. Done! Please see line 65.

Comment: **L60-72: This is not introduction, but actually another summary, which I think is obsolete, a slightly more extended real introduction discussing backgraound prior knowledge, and aims of the study, would be preferred**

Reply: Many thanks. We have moved this section of our previous version to Data Description according to GigaScience author guidelines and your suggestions, please lines 91-109. Meanwhile, we have revised our Introduction section, please see lines 52-85.

Comment: **Figure 1B: this panel is nice, but not very informative, what exact information is retrieved from the graph?**

Reply: Apologies for any problems with our figures. The complicated circos plot is the result of the many scaffolds (78,488) in the current duck reference genome. We have removed the circos plot from our current manuscript, please see Figure 1.

Comment: **L95: The number of deletions was higher than the number of insertions in all nine populations**

Reply: Done! Please see line 134.

Comment: **L105: Move the sentence "Single base-pair INDELs were the predominant form, accounting for 38.63% of all detected INDELs (Supplemental Table S3)." before the sentence "Both the number of SNPs ..."**

Reply: Thank you so much for your kind suggestion. We revised our manuscript accordingly, please see lines 142-143.

Comment: **L111: ... clustered together, the three ...**

Reply: Done! Please see line 155.

Comment: **L117: Show figure for K=2?**

Reply: Thanks for your question. Both K=2 and K=3 were showed in fig 2C, please see line 166.

Comment: **L155: ... had the lowest Akaike Information Criteria (AIC) value, ...**

Reply: Done! Please see lines 200-201.

Comment: **L166: ... are lower than in wild mallards ...**

Reply: Done! Please see line 213.

Comment: **Table 1: is it possible to report standard errors or confidence intervals of the reported estimates?**

Reply: Many thanks for your question. To answer the reviewer's question we added 95% confidence intervals to all estimates. We reanalyzed the demographic history of duck domestication based on mutation rates of both zebra finch and chicken. Using the mutation rate of zebra finch (Jarvis et al. 2014), the time of duck domestication is estimated at 2,128 (+- 421) years ago. With estimates of mutation rate from chicken (Nam et al. 2010), we estimate domestication 2,228 (+- 441) years ago. Considering the genetic relationship of duck to chicken is much closer than to zebra finch (Jarvis, E. D., et al. 2014), we revised the PSMC and δaδi results of our current manuscript, please see Fig 2D, Table 1, and lines 203-211, 547-549.

Comment: **L197: ... white plumage phenotype suggesting a causative mutation.**

Our result indicates for the first time the duck white plumage associated with selection at ...

Reply: Done! Please see lines 245-247.

Comment: **L213: of 10kb size.**

Reply: Done! Please see line 267.

Comment: **L224: "... scaffolds longer than 10-kb by 10-kb windows with 5-kb steps." This is not clear to me, please describe better.**

Reply: Apologies for any confusion. In our study, both F_{ST} and π were calculated for each 10kb size window, with 5kb size steps. However, of the 78,488 scaffolds in the duck reference genome, there are many scaffolds < 10kb. These short scaffolds were removed, and we only calculated F_{ST} for scaffolds > 10kb. We have added this to our revised manuscript, please see lines 279-281.

Comment: **L237 was shown**

Reply: Done! Please see lines 293-294.

Comment: **L240 level differs between domesticated and wild duck.**

Reply: Done! Please see line 296.

Comment: **L245 I understand that you limited the GO analysis to certain processes, what happened if you included other processes as well?**

Reply: Many thanks for this suggestion. In this study, all 292 genes located in the 5% F_{ST} regions (supplementary table S5) were used for the GO analysis, resulting in a total of 57 GO enrichment terms, which have now all been added to our current manuscript, please see lines 300-301, and supplementary table S6. This high number of GO terms presents a hopelessly difficult and complicated analyses, therefore we selected a subset of GO terms for further analysis based on P-value (supplementary table S7) combined the phenotypic differences between wild mallard and domestic duck. We do agree with the reviewer that a more inclusive analysis would be preferable, but the large number of GO terms makes it impossible to obtain meaningful results.

Comment: **L252 identified as being under positive selection**

Reply: Corrected! Please see line 311.

Comment: **L258 Is "neuronal genes" the right term?**

Reply: Apologies for any confusion. "Neuronal genes" is not in fact a GO term, rather a simplification of "25 neuro-synapse-axon genes" in line 310. To be more understandable, we have removed this simplification in our revision, please see line 317.

Comment: L260 fatty acid

Reply: Apologies and corrected! Please see line 319.

Comment: L269 and no gene in breast muscle

Reply: Done! Please see line 329.

Comment: L273 The results suggest that the PDC gene is of substantial functional importance in phenotypic differentiation among wild and domestic ducks.

Reply: Many thanks. We have revised this sentence according to your suggestion, please see lines 333-335.

Comment: L289 catalogued 36.1M SNPs and 3.1M INDELs,

Reply: Corrected! Please see line 349.

Comment: L333 ... showed particularly strong signs of selective sweep s presumably associated with domestication.

Reply: We have corrected our manuscript according to your suggestion, please see lines 398-399.

Comment: L340 brain and liver of domesticated ducks compared to ...

Reply: Corrected! Please see line 405.

Comment: L351 differential selection? Do you mean directional selection?

Reply: Apologies for any confusion. We also revised our current manuscript, please see lines 416-418.

Comment: L362 Taken together, our results show that duck domestication was a relatively recent and ...

Reply: We have corrected our manuscript according to your suggestion, please see line 430.

Comment: **L440 From the 28,199,227 SNPs not confirmed by dbSNPs, 390 randomly chosen (?) nucleotide sites**

Reply: Many thanks for your question. Of course, all nucleotide sites were randomly selected. We have added this explain to our current manuscript, please see lines 510-513.

Comment: **L448 Principal Component Analysis (PCA), first by generating the genetic relationship matrix (GRM) from which the first 20 eigenvectors were extracted.**

Reply: We have corrected our manuscript according to your suggestion, please see line 520-522.

--

Please also take a moment to check our website at http://giga.edmgr.com/l.asp?i=25723&l=YHKU51UQ for any additional comments that were saved as attachments. Please note that as GigaScience has a policy of open peer review, you will be able to see the names of the reviewers.
