# Supplementary material for: Whole-genome resequencing reveals signatures of selection and timing of duck domestication
Source: Gigascience. 2018 Apr 9;7(4):giy027. doi: 10.1093/gigascience/giy027 (PMC6007426; doi:10.1093/gigascience/giy027)
Supplement: GIGA-D-17-00301_Revision_2.pdf [file giy027_giga-d-17-00301_revision_2.pdf]

GigaScience

# Whole-genome resequencing reveals signatures of selection and timing of duck domestication

--Manuscript Draft--

| | |
|---|---|
| **Manuscript Number:** | GIGA-D-17-00301R2 |
| **Full Title:** | Whole-genome resequencing reveals signatures of selection and timing of duck domestication |
| **Article Type:** | Research |
| **Funding Information:** | Beijing Innovation Team of the Modern Agro-industry Technology Research System (BAIC04-2017) — Prof. Lujiang Qu<br>European Research Council (680951) — Dr. Judith E Mank<br>Wolfson Merit Award — Dr. Judith E Mank |
| **Abstract:** | Background: The genetic basis of animal domestication remains poorly understood, and systems with substantial phenotypic differences between wild and domestic populations are useful for elucidating the genetic basis of adaptation to new environments as well as the genetic basis of rapid phenotypic change. Here, we sequenced the whole genome of 78 individual ducks, from two wild and seven domesticated populations, with an average sequencing depth of 6.42X per individual.<br>Results: Our population and demographic analyses indicate a complex history of domestication, with early selection for separate meat and egg lineages. Genomic comparison of wild to domesticated populations suggest that genes affecting brain and neuronal development have undergone strong positive selection during domestication. Our FST analysis also indicates that the duck white plumage is the result of selection at the melanogenesis associated transcription factor locus.<br>Conclusions: Our results advance the understanding of animal domestication and selection for complex phenotypic traits. |
| **Corresponding Author:** | Lujiang Qu, Ph.D.<br>China Agricultural University<br>Beijing, CHINA |
| **Corresponding Author Secondary Information:** | |
| **Corresponding Author's Institution:** | China Agricultural University |
| **Corresponding Author's Secondary Institution:** | |
| **First Author:** | Zebin Zhang |
| **First Author Secondary Information:** | |
| **Order of Authors:** | Zebin Zhang<br>Yaxiong Jia<br>Pedro Almeida<br>Judith E Mank<br>Marcel van Tuinen<br>Qiong Wang<br>Zhihua Jiang<br>Yu Chen<br>Kai Zhan<br>Shuisheng Hou |

Powered by Editorial Manager® and ProduXion Manager® from Aries Systems Corporation

| | |
|---|---|
| | Zhengkui Zhou |
| | Huifang Li |
| | Fangxi Yang |
| | Yong He |
| | Zhonghua Ning |
| | Ning Yang |
| | Lujiang Qu, Ph.D. |
| **Order of Authors Secondary Information:** | |

**Response to Reviewers:**

Dear Dr Zauner,

Many thanks for your positive comments about our manuscript, “Whole-genome resequencing reveals signatures of selection and timing of duck domestication” (manuscript number GIGA-D-17-00301R1). We also thank the reviewers for their thoughtful and constructive suggestions. We have addressed all these comments, detailed below, in our revised manuscript, which we hope is now suitable for publication in GigaScience.

Sincerely,
Lujiang Qu, Ph.D., on behalf of all co-authors.
Email: quluj@163.com
Department of Animal Genetics and Breeding, College of Animal Science and Technology, China Agricultural University, Beijing 100193, China

GIGA-D-17-00301R1
Whole-genome resequencing reveals signatures of selection and timing of duck domestication
Zebin Zhang; Yaxiong Jia; Pedro Almeida; Judith E Mank; Marcel van Tuinen; Qiong Wang; Zhihua Jiang; Yu Chen; Kai Zhan; Shuisheng Hou; Zhengkui Zhou; Huifang Li; Fangxi Yang; Yong He; Zhonghua Ning; Ning Yang; Lujiang Qu, Ph.D.
GigaScience

Dear Prof. Qu,

Your revised manuscript "Whole-genome resequencing reveals signatures of selection and timing of duck domestication" (GIGA-D-17-00301R1) has been assessed again by our reviewers.

I am happy that the reviewers feel that many of their previous comments have been addressed and the manuscript has improved. However, some issues remain to be clarified, and I urge you to fully address the latest comments in a second revised manuscript.

Please see the reviewers' reports below.

Comment: Please pay particular attention to the comments of reviewer 1 regarding availability of population genetics raw data, coordinates of sweeps, scripts, etc, as well as full step-by-step description of all wet and dry lab protocols.  As I explained in my previous decision letter, reproducibility of methods and full data availability are of utmost importance for acceptance in GigaScience.

Reply: Many thanks for your comment. All population genetic raw data and command scripts have been submitted to the GigaDB database according to reviewer 1 and your suggestion. We also add the description of all wet and dry protocols to our current manuscript, please see specific replies below.

As mentioned previously, the protocols.io platform is a very convenient way to share experimental protocols, and I recommend you to consider this option. Please do let me know if you have questions regarding how we can integrate protocols.io entries with your manuscript.

Powered by Editorial Manager® and ProduXion Manager® from Aries Systems Corporation

Our data curators will contact you to prepare the GigaDB set that will be posted alongside your manuscript, if it is accepted.

Please include a citation to your GigaDB dataset (including the DOI link) to your reference list, and cite this in the data availability section and elsewhere in the manuscript, where appropriate.

Please follow this example format for the reference:

[xx] Author1 N, Author2 N, AuthorX N. Supporting data for "Title of your manuscript". GigaScience Database 2018. http://dx.doi.orgxxxxxxxxxxxx

(We will replace the dummy doi (xxxx) with the final version prior to acceptance).

Once you have made the necessary corrections, please submit a revised manuscript online at:

https://giga.editorialmanager.com/

If you have forgotten your username or password please use the "Send Login Details" link to get your login information. For security reasons, your password will be reset.

Please include a point-by-point within the 'Response to Reviewers' box in the submission system.
Please also ensure that your revised manuscript conforms to the journal style, which can be found in the Instructions for Authors on the journal homepage.

The due date for submitting the revised version of your article is 15 May 2018.

We look forward to receiving your revised manuscript soon.

Best wishes,

Hans Zauner
GigaScience
www.gigasciencejournal.com

Reviewer reports:
Reviewer #1: In my opinion, this revision adequately answers most of my comments. The manuscript has also improved with the answers to the other reviewer.

I have only a few remaining comments. The most serious one is about data availability and protocols.

Comment: The revision comes with better data availability. VCF files of variants are included, plus a couple of perl scripts used to process them. However, full population genetic statistics and sweep locations still seem to be missing. Scripts for running the bioinformatic tools are not included. The description of the PCR follow-up of variants has been expanded. However, the description does not include the full protocol, and neither does the description of any of the other laboratory methods. This level of detail is about the standard in the field, but it does not seem to live up to the policies of the journal.

Reply: Many thanks for your positive comments and apologies for any inadequate descriptions. All population genetic raw data and command scripts have been submitted to the GigaDB database.
We used a sliding windows method for FST calculation in our sweep analysis, as this approach is more robust and informative for genome-wide evaluation. This approach means that one window might have several genes, and some very long genes may be present in multiple overlapping windows. Thus, we substituted sweep locations for gene locations, and added this information to our current manuscript, please see supplemental tables S5 and S8.

We have provided a citation for the specific PCR validation methods (Van et al 2008), which has been widely used in previous studies (Wang et al 2016, Yan et al 2014), please see line 536.

Van Tassell, C. P., et al. (2008). "SNP discovery and allele frequency estimation by deep sequencing of reduced representation libraries." Nat Methods 5(3): 247-252.
Wang, M. S., et al. (2016). "Positive selection rather than relaxation of functional constraint drives the evolution of vision during chicken domestication." Cell Res 26(5): 556-573.
Yan, Y., et al. (2014). "Genome-wide characterization of insertion and deletion variation in chicken using next generation sequencing." PLoS One 9(8): e104652.

Comment: A couple of times (the justification for the mix of sequence coverages, and the detail about the origin of the ducks), the reply to reviewers contain useful information that was not incorporated in the manuscript. In my opinion, the Methods should include this information, and in particular as much detail as possible about the origin of the animals.

Reply: Many thanks for your suggestion. We have add the justification of coverage to the Methods section of our current manuscript, please see lines 486-490. We have also detailed the point of origin for our samples, please see lines 468-474.

Minor comments

Comment: The reply to reviewers describe the variant filtering as "extremely strict". In fact, it seems to be mostly the default starting criteria suggested by GATK developers in their "best practices" (with a "QUAL" cutoff and a higher "QD" cutoff). How were these filter settings chosen? Are they actually "extremely strict"?

Reply: Many thanks for your questions. Of course, all variants were filtered with “hard filter” criteria suggested by GATK developers. However, to identify variants associated with white plumage traits, the “extremely strict” criteria were used, where variant allele frequency must be 0 in all white duck individuals and be 1 in all non-white duck individuals. Or, 1 in all white duck individuals and 0 in all non-white duck individuals. In other words, the variant had to be completely associated with the phenotype to pass our strictest threshold.

Comment: Line 247: What does "completely associated with selection" mean in this context?

Reply: Thanks for your question. “The duck white plumage is completely associated with selection at the MITF locus” means the mutations were completely associated with white plumage phenotype.

Comment: Lines 252-253: In what sense did the PCR primer design fail? Were you unable to amplify the region, amplify specifically, or unable to find primers that lived up to your quality criteria? I fully understand that PCR primer design fails occasionally, but I think a more specific description would be useful.

Reply: We were unable to design suitable primers to amplify this region, and we add this explanation to our current manuscript, please see line 270.

Reviewer #2: The revised version of the manuscript entitled, "Whole-genome resequencing reveals signatures of selection and timing of duck domestication" tackles the genomic question of domestication. The authors have done much to improve the manuscript. While most of my comments are now minor, there are a few additional requests that would be nice to see incorporated in order to strengthen the manuscript. I believe that the paper will be ready for submission if the authors incorporate all/most comments (See below).

Comment: INTRODUCTION/DATA DESCRIPTION: I think the introduction is much improved. In addition to minor comments below, I would still like to see the authors develop at least one hypothesis as to what genes/genetic regions may be playing a

role in the meat/egg domestication process of these ducks. Alternatively (or in addition to), I would like to see a hypothesis regarding what they think some of the differences may be between wild and domesticated populations.

Reply: Thank you very much for your positive comments. Respectfully, the advantage of comparative genomic studies such as ours is that they are agnostic screens of the entire genome without a priori need to develop specific hypotheses. Previous similar studies of domestication (including Rubin et al. Nature 2010; Vonholdt et al. Nature 2010; Montague et al. PNAS 2014, among many others) have used these approaches to identify regions of the genome affected by artificial selection without a priori hypotheses. We adapted these approaches to the study of ducks here, with the broad aim of identifying whether ducks were domesticated once (null hypothesis) or separately for egg and meat breeds (alternative hypothesis). Moreover, we assess the role of domestication on genes related to plumage and neuroanatomy. We respectfully suggest that to develop further post hoc hypotheses to fit our results at this point would be disingenuous, and defeat the purpose of these sorts of agnostic screens.

Rubin, C. J., et al. (2010). "Whole-genome resequencing reveals loci under selection during chicken domestication." Nature 464(7288): 587-591.
Vonholdt, B. M., et al. (2010). "Genome-wide SNP and haplotype analyses reveal a rich history underlying dog domestication." Nature 464(7290): 898-902.
Montague, M. J., et al. (2014). "Comparative analysis of the domestic cat genome reveals genetic signatures underlying feline biology and domestication." Proceedings of the National Academy of Sciences 111(48): 17230-17235.

Comment: Line 63: remove scientific name as you already introduced mallards in the previous paragraph.

Reply: Done! Please see line 72.

Comment: Line 92: insert "of" - "….613.37 [of] Gb high….". I would also advise the authors to move any kind of findings of this type to RESULTS.

Reply: Done! Please see lines 89-91, 111-112.

Comment: Lines 94: Delete "we detected"

Reply: Done! Please see line 92.

Comment: Line 94: consider change " …,we tested for population structure between domesticated and wild populations, as well as assessed for signatures of selection associated with domestication."

Reply: Many thanks for your helpful suggestion. We have revised our manuscript accordingly, please see lines 92-96.

Comment: Line 96-98: Either delete the sentence starting with "We inferred…" or add another 1-2 sentence explaining what exactly you tested.

Reply: Deleted! Please see line 95-97.

Comment: Lines 104-109: This seems forced and out of place. Either delete it and put it to the discussion OR expand/edit it to be more streamlined.

Reply: This paragraph have been moved to discussion section, please see lines 100-105, and 449-454.

ANALYSIS:

Comment: Line 117: end with "…78 ducks."

Reply: Done! Please see line 113.

Comment: 2nd Paragraph: "Across samples, a total of 36.1 million (M) SNPs (average

per sample = 4.5 M SNPs; range = 2.34 - 9.52 M SNPs) and 3.1M INDELs (average per sample = 0.4M INDELs; range = 0.21 - 0.89M INDELs) were detected (Fig. 1C1B, Supplemental Figs. S1-S2, Supplemental Table S2). ingle base-pair INDELs were the predominant form, and accounting for 38.63% of all detected INDELs (Supplemental Table S3). Our dataset covers 96.2% of the duck dbSNP database deposited in the Genome Variation Map (GVM) (http://bigd.big.ac.cn/gvm/)." In general, domesticated stock showed lower number of SNPs (t test, $p = 3.13 \times 10^{-12}$) and nucleotide diversity (ttest, $p = 2.20 \times 10^{-16}$) as compared to wild mallards (Fig. 1B - C). Moreover, homozygousity in domesticated ducks was significantly higher than ratios in wild mallards (t test, $p = 1.35 \times 10^{-10}$ ) consistent with the larger panmictic wild population.

Reply: Thank you so much for your helpful suggestion. This paragraph was revised accordingly, please see lines 126-151.

Comment: Line 137: does 36.1 million SNPs include indels? If not, I would just include the 2 in one summation of total diversity.

Reply: Many thanks for your question and helpful suggestion, the 36.1 million SNPs did not include INDELs. These two variation types are summed together according to your suggestion in our current manuscript, please see line 127.

Comment: Line 142 - 143: The sentence "Single base-pair INDELs were the predominant form, accounting for 38.63% of all detected INDELs (Supplemental Table S3)."

Reply: Revised! Please see lines 131-132.

Comment: Line 148: Are you sure that your data is "consistent with larger panmictic wild population" ? What about artificial selection and inbreeding within domesticated stock? Maybe both? Consider revising.

Reply: Apologies for any confusion. We had revised our manuscript accordingly, please see lines 138-140.

Comment: Lines 155 - 158: Consider changing the sentence to: "In general, clustering among samples corresponded with their source, that included wild ducks (MDN and MDZ), ducks domesticated for meat production (PK, CV, and ML), and ducks domesticated for egg production (JD, 157 SM, and SX). The dual-purpose domesticate clustered with ducks domesticated for egg production (Fig. 2B-C)."

Reply: Done! Please see lines 156-160.

Comment: Lines 184-202: Consider revising to 1 paragraph: "Next, we explored the demographic history of our samples to differentiate whether domestication of meat and egg producing ducks was the result of one or multiple events. First, we estimated changes in effective population size (Ne) in our three genetic clusters in a pairwise sequentially Markovian coalescent (PSMC) framework [22]. The meat type ducks (PK, CV, and ML) showed concordant demographic trajectories with egg and mixture dual-purpose type populations (JD, SM, SX, and GY) with one apparent expansion around the Penultimate Glaciation Period (PGP, 0.30-0.13 Mya) [4, 23] and Last Glacial Period (LGP, 110-12 kya) [24, 25], followed by a subsequent contraction (Fig. 2D). Next, we tested multiple demographic scenarios …."

Reply: Done! Please see lines 187-208.

Comment: Line 214: What is the Ne for the wild population. Please make clear by at least referencing Table 1.

Reply: Thank you for this helpful suggestion. We have had add the Ne estimate of the wild population to our main text, please see line 225.

Comment: Lines 224-229: Please cite sources for some of your statements here. Better to make the statement of your findings and save lines 226-229 for discussion.

Powered by Editorial Manager® and ProduXion Manager® from Aries Systems Corporation

| | |
|---|---|
| | Reply: Many thanks for your comments. We have moved lines 226-229 to the discussion section according as suggested, please see lines 387-390.<br><br>Comment: Line 241: I would like to know if any other region showed deviation/outliers? Or was there only 1 region across the entire genome? Please clarify.<br><br>Reply: Many thanks for your questions. This region is the fourth ranked region across the entire genome, but the only one region correlated with coloration. We also revised our current manuscript, please see lines 251-261.<br><br>Comment: DISCUSSION: Overall, the discussion is well written, organized, and I find the topics of broad appeal.<br><br>I believe the introduction of the Discussion can be combined into a single paragraph and a bit streamlined as it is just reiterating the results.<br><br>Reply: Thank you so much for your positive comment and your helpful suggestion. The introduction of the Discussion have been revised and redundant material deleted as you suggest, please see lines 348-363.<br><br>Comment: Lines 348 - 353: Consider splitting into at least 2 sentences.<br><br>Reply: Done! Please see lines 357-363.<br><br>Comment: Line 419: add "and": "dogs [45], and…"<br><br>Reply: Done! Please see line 433. |
| **Additional Information:** | |
| **Question** | **Response** |
| Are you submitting this manuscript to a special series or article collection? | No |
| **Experimental design and statistics**<br><br>Full details of the experimental design and statistical methods used should be given in the Methods section, as detailed in our Minimum Standards Reporting Checklist. Information essential to interpreting the data presented should be made available in the figure legends.<br><br>Have you included all the information requested in your manuscript? | Yes |
| **Resources**<br><br>A description of all resources used, including antibodies, cell lines, animals and software tools, with enough information to allow them to be uniquely identified, should be included in the Methods section. Authors are strongly encouraged to cite Research Resource Identifiers (RRIDs) for antibodies, model organisms and tools, where possible. | Yes |

| | |
|---|---|
| Have you included the information requested as detailed in our Minimum Standards Reporting Checklist? | |
| **Availability of data and materials**<br><br>All datasets and code on which the conclusions of the paper rely must be either included in your submission or deposited in publicly available repositories (where available and ethically appropriate), referencing such data using a unique identifier in the references and in the "Availability of Data and Materials" section of your manuscript.<br><br>Have you have met the above requirement as detailed in our Minimum Standards Reporting Checklist? | Yes |

Powered by Editorial Manager® and ProduXion Manager® from Aries Systems Corporation

Manuscript

Click here to download Manuscript Manuscript_of_Duck_Domestication_17_3.docx

# Whole-genome resequencing reveals signatures of selection and timing of duck domestication

Zebin Zhang [†,1], Yaxiong Jia[†,2], Pedro Almeida[3], Judith E Mank[3,4], Marcel van Tuinen[5], Qiong Wang[1], Zhihua Jiang[6], Yu Chen[7], Kai Zhan[8], Shuisheng Hou[2], Zhengkui Zhou[2], Huifang Li[9] Fangxi Yang[10], Yong He[11], Zhonghua Ning[1], Ning Yang[1], and Lujiang Qu[1*]

[1]Department of Animal Genetics and Breeding, National Engineering Laboratory for Animal Breeding, College of Animal Science and Technology, China Agricultural University, Beijing, China

[2]Institute of Animal Science, Chinese Academy of Agricultural Sciences, Beijing, China

[3]Department of Genetics, Evolution and Environment, University College London, London, UK

[4]Department of Organismal Biology, Evolutionary Biology Centre, Uppsala University, Uppsala, Sweden

[5]Centre of Evolutionary and Ecological Studies, Marine Evolution and Conservation Group, University of Groningen, Groningen, The Netherlands

[6]Department of Animal Sciences, Center for Reproductive Biology, Veterinary and Biomedical Research Building, Washington State University, Pullman, United States

[7]Beijing Municipal General Station of Animal Science, Beijing, China

[8]Institute of Animal Husbandry and Veterinary Medicine, Anhui Academy of Agricultural Sciences, Hefei, China

[9]Poultry Institute, Chinese Academy of Agriculture Science, Yangzhou, China

[10]Institute of Pekin Duck，Beijing, China

[11]Cherry Valley farms (xianghe) Co.,Ltd, Langfang, China

[†]These authors contributed equally to this work.

*Corresponding authors: quluj@163.com

# Abstract

**Background:** The genetic basis of animal domestication remains poorly understood, and systems with substantial phenotypic differences between wild and domestic populations are useful for elucidating the genetic basis of adaptation to new environments as well as the genetic basis of rapid phenotypic change. Here, we sequenced the whole genome of 78 individual ducks, from two wild and seven domesticated populations, with an average sequencing depth of 6.42X per individual.

**Results:** Our population and demographic analyses indicate a complex history of domestication, with early selection for separate meat and egg lineages. Genomic comparison of wild to domesticated populations suggest that genes affecting brain and neuronal development have undergone strong positive selection during domestication. Our $F_{ST}$ analysis also indicates that the duck white plumage is the result of selection at the *melanogenesis associated transcription factor* locus.

**Conclusions:** Our results advance the understanding of animal domestication and selection for complex phenotypic traits.

**Keywords:** duck, domestication, intensive selection, neuronal development, energy metabolism, plumage colouration.

## Background

Animal domestication was one of the major contributory factors to the agricultural revolution during the Neolithic period, which resulted in a shift in human lifestyle from hunting to farming [1]. Compared with their wild progenitors, domesticated animals showed notable changes in behavior, morphology, physiology, and reproduction [2]. Detecting domestication-mediated selective signatures is important for understanding the genetic basis of both adaptation to new environments and rapid phenotype change [3, 4]. In recent years, to characterize signatures of domestication, whole genome resequencing studies have been performed on a wide range of agricultural animals, including pig [5], sheep [6], rabbit [7] and chicken [8, 9].

Mallards (*Anas platyrhynchos*) are the world's most widely distributed and agriculturally important waterfowl species, and are of particular economic importance in Asia [10]. Southeast Asia, particularly southern China, is the major center of duck domestication, with records indicating duck farming in the region dating at least 2,000 years [11, 12], particularly in wet environments [13] associated with rice crops [14]. In the absence of archaeological evidence, the exact timing of domestication and the time of meat and egg type ducks split remains unknown, with the first written records indicating domestic ducks in central China shortly after 500 BC [15].

It is clear that the domesticated duck originated from mallards [16], and domestic ducks can be classified as those produced primarily for meat (similar

to chicken broilers) or eggs (similar to chicken layer lines). Together with the timing of duck domestication, the relative separation of duck meat and egg lines is also unknown. It is unclear whether ducks were domesticated once, and subsequently selected for divergent meat and egg production traits, or whether meat and egg populations were derived independently in two domestication events from wild mallards.

Moreover, domesticated mallards show many important behavioral [17] and morphological [18-20] differences from their wild ancestors, particularly related to plumage and neuroanatomy. However, the genetic basis of these phenotypic differences are still poorly understood.

## Data Description

In order to determine the timing of duck domestication in China, as well as identify the genomic regions under selection during domestication, we performed whole genome resequencing from 78 individuals belonging to seven different duck breeds (three for meat breeds, three for egg breeds, and one dual-purpose breed) and two geographically distinct wild populations. Using the large number of single nucleotide polymorphisms (SNPs) as well as small insertions and deletions (INDELs), we tested for population structure between domesticated and wild populations, as well as assessed the genome for signatures of selection associated with domestication. We tested alternative demographic scenarios with the pairwise sequential Markovian coalescent

method combined with the diffusion approximation method.

# Analyses

## Genetic variation

We individually sequenced 22 wild and 56 domestic ducks, from two wild populations and seven domestic breeds (three meat breeds, three egg breeds and one dual-purpose breed), from across China (Fig. 1A) to an average of 6.42X coverage per individual (a total of 613.37 of Gb high quality paired end sequence data) after filtering and quality control, resulting in total 535 billion mappable reads across 78 ducks (Supplemental Table S1).

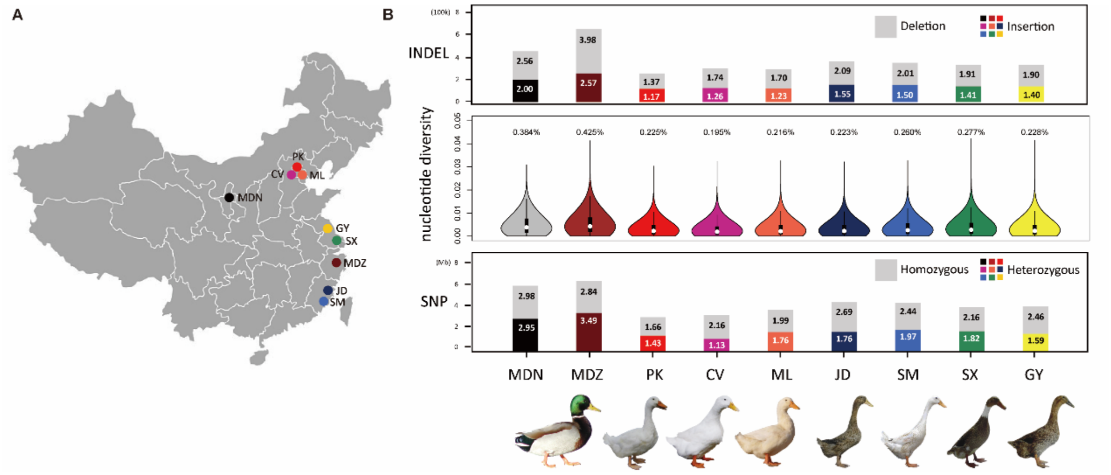


**Figure. 1 Experimental design and variants statistics**

**(A)** Sampling sites in this study. A total of 78 ducks from two wild populations (Mallard Ningxia (MDN) n=8; Mallard Zhejiang (MDZ) n=14), three meat breeds (Pekin (PK) n=8; Cherry Valley (CV) n=8; Maple Leaf (ML) n=8), three egg breeds (Jin Ding (JD) n=8; Shan Ma (SM) n=8; Shao Xing (SX) n=8), and one dual purpose breed (Gao You (GY) n=8) were selected.

**(B)** Genomic variation of nine populations. Mean number of SNPs, heterozygous and

homozygous SNP ratio in the nine populations are shown at the bottom. Nucleotide diversity ratios of the nine populations are shown at the middle. The nucleotide diversity ratios in wild mallards are dramatically higher than ratios in domesticated ducks. Number of insertions and deletions in the nine populations are shown at the top. The number of deletions was higher than the number of insertions in all nine populations.

Across samples, we identified a total of 39.2 million (M) variants, consisting of 36.1 M SNPs (average per sample = 4.5 M SNPs; range = 2.34 - 9.52 M SNPs) and 3.1 M INDELs (average per sample = 0.4 M INDELs; range = 0.21 - 0.89 M INDELs) (Fig. 1B, Supplemental Figs. S1 - S2, Supplemental Table S2). Single base-pair INDELs were the most common, accounting for 38.63% of all detected INDELs (Supplemental Table S3). Our dataset covers 96.2% of the duck dbSNP database deposited in the Genome Variation Map (GVM) (http://bigd.big.ac.cn/gvm/). In general, domesticated populations showed lower number of SNPs (t test, $p = 3.13 \times 10^{-12}$) and nucleotide diversity (t test, $p = 2.20 \times 10^{-16}$ ) as compared to wild mallards (Fig. 1B). Moreover, homozygosity in domesticated ducks was significantly higher than ratios in wild mallards (t test, $p = 1.35 \times 10^{-10}$) consistent with the larger panmictic wild population or with the higher artificial selection and inbreeding within domesticated stocks.

## Population structure and domestication

Phylogenetic relationships, based on a neighbor-joining (NJ) of pairwise genetic distances of whole genome SNPs (Fig. 2A) and Principal Component Analysis (PCA, Fig. 2B) revealed strong clustering into three distinct genetic groups. In general, we observed separate clusters corresponding to wild ducks (MDN and MDZ), ducks domesticated for meat production (PK, CV, and ML), and ducks domesticated for egg production (JD, SM, and SX). The dual-purpose domesticate (GY) clustered with ducks domesticated for egg production (Fig. 2B-C).

We further performed population structure analysis using FRAPPE [21], which estimates individual ancestry and admixture proportions assuming K ancestral populations (Fig. 2C). With K = 2, a clear division was found between wild type ducks (MDN and MDZ) and domesticated ducks (PK, CV, ML, JD, SM, SX, and GY). With K = 3, a clear division was found between meat type ducks (PK, CV, and ML) and egg type ducks mixed with dual-purpose type ducks (JD, SM, SX, and GY).

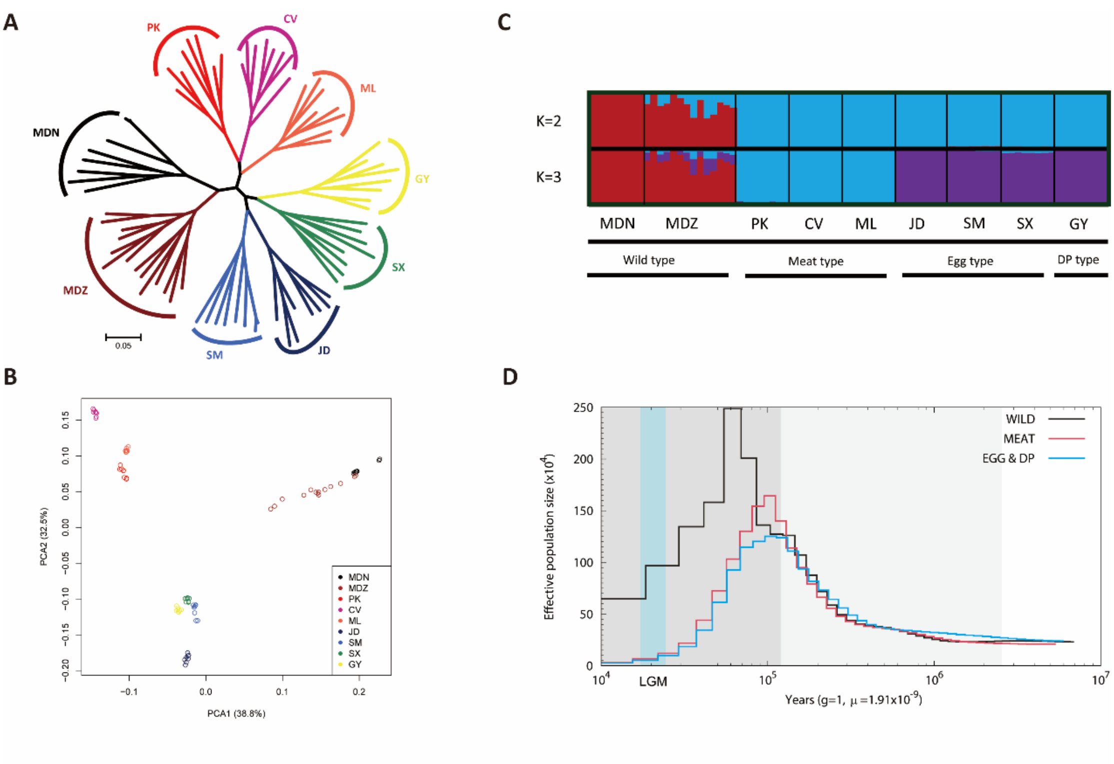


**Figure. 2 Population genetic structure and demographic history of nine duck populations**

**(A)** Neighbor-joining phylogenetic tree of nine duck populations. The scale bar is proportional to genetic differentiation (p dist ance).

**(B)** PCA plot of duck populations. Eigenvector 1 and 2 explained 38.8% and 32.5% of the observed variance, respectively.

**(C)** Population genetic structure of 78 ducks. The length of each colored segment represents the proportion of the individual genome inferred from ancestral populations (K = 2-3). The population names and production type are at the bottom. DP type means dual-purpose type.

**(D)** Demographic history of duck populations. Examples of PSMC estimate changes in the effective population size over time, representing variation in inferred Ne dynamics. The lines represent inferred population sizes and the gray shaded areas indicate the Pleistocene period,

with Last Glacial Period (LGP) shown in darker gray, and Last Glacial Maximum (LGM) shown in light blue areas.

Next, we explored the demographic history of our samples to differentiate whether domestication of meat and egg producing ducks was the result of one or multiple events. First, we estimated changes in effective population size ($N_e$) in our three genetic clusters in a pairwise sequentially Markovian coalescent (PSMC) framework [22]. The meat type ducks (PK, CV, and ML) showed concordant demographic trajectories with egg and mixture dual-purpose type populations (JD, SM, SX, and GY) with one apparent expansion around the Penultimate Glaciation Period (PGP, 0.30-0.13 Mya) [4, 23] and Last Glacial Period (LGP, 110-12 kya) [24, 25], followed by a subsequent contraction (Fig. 2D). Next, we tested multiple demographic scenarios related to domestication using a diffusion approximation method for the allele frequency spectrum (∂a∂i) (Supplemental Fig. S3 and S4). Among the four isolation models tested (models 1 - 4), the model of a single domestication with subsequent divergence of the domesticated breeds (Model 2) was both consistent with our population structure results (Fig. 2) and had the lowest Akaike Information Criteria (AIC) value, indicating a better overall fit to the data (log-likelihood = -33,388.43; AIC = 66,788) (Supplemental Fig. S3).

Demographic parameters estimated from the single domestication model (Model 2) indicated that domestication occurred 2,228, with 95% confidence intervals (CI) ± 441 years ago, followed by a rapid subsequent divergence of

the meat breed from the egg/dual purpose breeds roughly 100 years after the initial domestication event (Table 1). Our results suggest that following an initial bottleneck associated with domestication, with an estimated $N_e$ of 320 (95% CI ± 3) individuals for the ancestral domesticated population, the population has expanded to the current $N_e$ of 5,597 (95% CI ± 1,195) and 12,988 (95% CI ± 2,877) in the meat type and egg/dual purpose breeds respectively. $N_e$ estimates for domesticated breeds are lower than Ne of 88,842 (95% CI ±18,065) in wild mallards, consistent with the large panmictic wild population.

**Table 1**. Maximum likelihood population demographic parameters. Best fit parameter estimates for the model of a single domestication event followed by divergence of the domesticated breeds, including changes in population size. 95% confidence intervals were obtained from 100 bootstrap data sets. Time estimates are given in years and migration are in units of number of migrants per generation.

| Parameter | ML estimate | 95% CI |
|---|---|---|
| $N_e$ of ancestral population after size change | 663,439 | 644,726 – 682,152 |
| $N_e$ of the wild population | 88,842 | 70,778 – 106,907 |
| $N_e$ of the ancestral domesticated population | 320 | 316 – 323 |
| $N_e$ of the meat breed | 5,597 | 4,402 – 6,792 |
| $N_e$ of the egg/dual purpose | 12,988 | 10,111 – 15,865 |
| Time of size change in the ancestral population | 249,944 | 227,912 – 267,518 |
| Time of domestication | 2,228 | 1,787 – 2,669 |
| Time of breed divergence | 2,126 | 1,686 – 2,567 |
| Migration $_{wild \leftarrow meat}$ | 1.12 | 1.00 – 1.24 |
| Migration $_{wild \leftarrow egg/dp}$ | 3.92 | 3.11 – 4.73 |

Gene flow estimates were relatively high, with 1 and 4 migrants per generation from the meat and egg/dual purpose breeds, respectively, into the wild population. Our results suggested duck domestication was a recent single

domestication event followed by rapid subsequent selection for separate meat and egg/dual purpose breeds.

## Selection for plumage color

Derived traits in domesticated animals tend to evolve in a predictable order, with color variation appearing in the earliest stages of domestication, followed by coat or plumage and structural (skeletal and soft tissue) variation, and finally behavioral differences [26, 27]. One of the simplest and most visible derived traits of ducks is white plumage color. In order to detect the signature of selection associated with white feathers, we searched the duck genome for regions with high $F_{ST}$ between the populations of white feather (PK, CV, and ML) and non-white feather (MDN, MDZ, JD, SX, and GY) birds based on sliding 10kb windows. We identified a region of high differentiation between white plumage and non-white plumage ducks overlapping the *melanogenesis associated transcription factor* (*MITF*; $F_{ST}$=0.69) (Fig. 3A). In the intronic region of *MITF*, we identified 13 homozygous SNPs and 2 homozygous INDELs present in all white plumage breeds (n=24) and absent in all non-white plumage breeds (n=46) (Fig. 3B). These mutations were completely associated with the white plumage phenotype, suggesting a causative mutation at the *MITF* locus. Moreover, to validate the reliability of variants detected in MITF gene, we amplified the first three SNPs (SNP817793, SNP817818, and SNP818004) and all INDELs by diagnostic PCR combined with Sanger sequencing in the 78 white and non-white plumage ducks. The results show that the three SNPs and

INDEL817958 completely match our NGS analysis (supplemental Fig. S5), For INDEL818495, we were unable to design a suitable PCR primer to amplify this region.

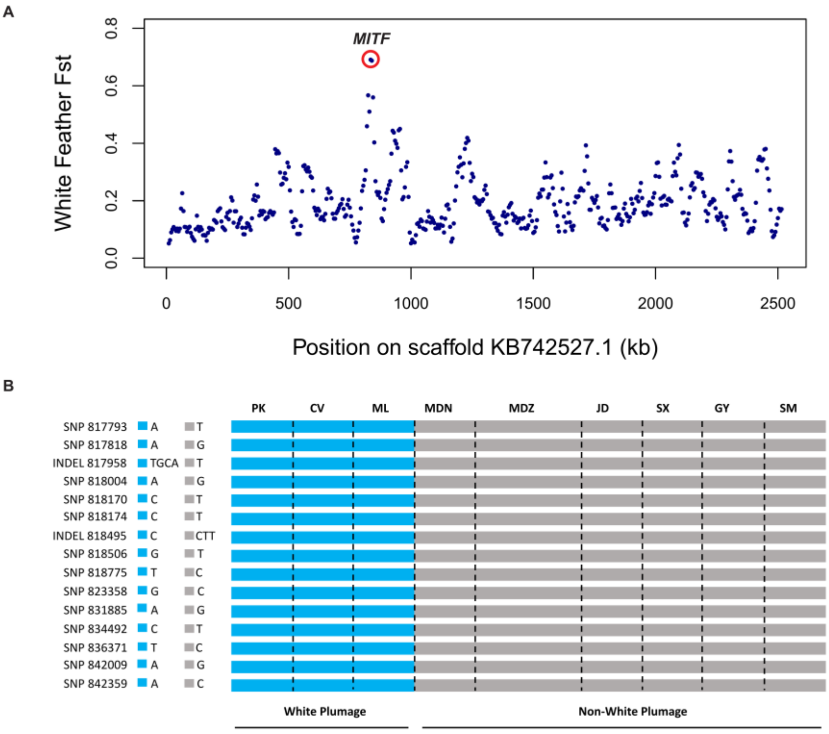


**Figure. 3 MITF shows different genetic signature between white plumage and non-white plumage ducks.**

**(A)** FST plot around the MITF locus. The $F_{ST}$ value of MITF is highest for scaffold KB742527.1, circled in red. Each plot represent a 10 kb windows.

**(B)** 13 homozygous SNPs and 2 homozygous INDELs were identified in white plumage ducks and absent in non-white plumage ducks. SNPs and INDELs were named according to their position on scaffold.

## Selection for other domestication traits

In order to detect the signature of selection for other traits associated with

duck domestication, we scanned the duck genome for regions with a high coefficient of nucleotide differentiation ($F_{ST}$) among the populations of wild (MDN and MDZ) and domesticated (PK, CV, ML, JD, SM, SX, and GY) ducks based on 10kb sliding windows, as well as global $F_{ST}$ between each population (Supplemental Tables S4). Owing to the complex and partly unresolved demographic history of these populations, it is difficult to define a strict threshold that distinguishes true sweeps from regions of homozygosity caused by drift. We therefore also calculated the pairwise diversity ratio ($\theta_\pi$(wild/domesticated)). We identified 292 genes in the top 5% of both $F_{ST}$ and $\theta_\pi$ scores, putatively under positive selection during domestication (Fig. 4A, Supplemental Tables S5).

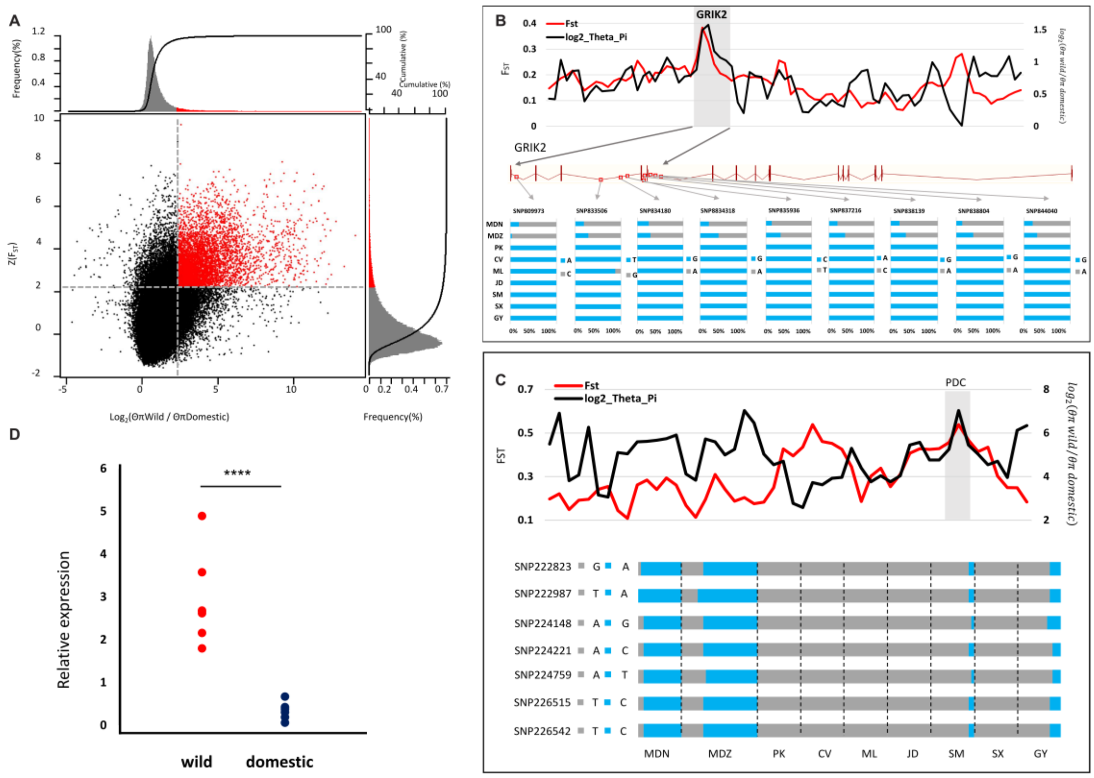


**Figure. 4 Genomic regions with strong selective sweep signals in wild population ducks and domesticated population ducks.**

**(A)** Distribution of θπ ratios θπ(wild/domesticated)) and Z($F_{ST}$) values, which are calculated by 10kb windows with 5kb steps. Only scaffolds > 10kb were used for our calculation, as $F_{ST}$ result calculated on small scaffold are unlikely to be accurate. Red data points located to the top-right regions correspond to the 5% right tails of empirical $log_2(\theta\pi\ wild/\theta\pi\ domestic)$ ratio distribution and the top 5% empirical Z($F_{ST}$) distribution are genomic regions under selection during duck domestication. The two horizontal and vertical gray lines represented the top 5% value of Z($F_{ST}$) (2.216) and $log_2(\theta\pi\ wild/\theta\pi\ domestic)$ (2.375), respectively.

**(B)** $log_2(\theta\pi)$ ratios and $F_{ST}$ values around the *GRIK2* locus and allele frequencies of nine SNPs within the *GRIK2* gene across nine duck populations. The black and red lines represent $log_2(\theta\pi\ wild/\theta\pi\ domestic)$ ratios and $F_{ST}$ values, respectively. The gray bar showed the region of under strong selection in *GRIK2* gene. The nine red rectangular frame corresponding to the locus on gene of nine SNPs. The SNPs were named according to their position on scaffold.

**(C)**The PDC gene showed different genetic signature in domesticated and wild duck. $log_2(\theta\pi)$ ratios and $F_{ST}$ values around the *PDC* locus. The *PDC* gene region is shown in gray. Allele frequencies of seven SNPs within the *PDC* gene across nine duck populations. The SNPs are named according to their scaffold position.

**(D)** The PDC gene expression level differs between domesticated and wild duck. PDC mRNA expression levels in brain of wild (MDN, n=3; MDZ, n=4) and domesticated (PK, n=1; CV, n=1; ML, n=1; JD, n=1; SM, n=1; SX, n=1; GY, n=1) ducks. ****$P$ value from *t*-test ($P$<0.0001).

All 292 genes located in the top 5% FST regions were used for the GO

analysis, resulting in a total of 57 GO enrichment terms (supplementary table S6). Because domesticated ducks are known to differ from wild ducks in body size, body fat percentage, behavior, egg productivity, growth speed, and flight capability, we focused our analysis on GO annotations of neural related processes, lipid metabolism and energy metabolism, reproduction, and skeletal muscle contraction for our 292 putative positively selection genes. In this reduced data set, the neuro-synapse-axon and lipid-energy metabolism pathways were over-represented (Supplemental Table S7) in our list of genes under selection.

From the highlighted GO terms, a total of 25 neuro-synapse-axon genes were identified as being under positive selection, with six (*ADGRB3*, *EFNA5*, *GRIN3A*, *GRIK2*, *SYNGAP1*, and *HOMER1*) in the top 1% of $F_{ST}$ and $\theta_\pi$ (Supplemental Tables S8). In particular, *GRIK2* (glutamate receptor, ionotropic kainate 2) and *GRIN3A* (glutamate receptor, subunit 3A) both showed high $F_{ST}$ and $\theta_\pi$ value compared to neighboring regions, suggesting functional importance (Fig. 3B, Supplemental Table S5, S8).

Beyond the neuronal-synapse-axon genes, 115 genes were identified in the four lipid and energy related pathways with high $F_{ST}$ and $\theta_\pi$ values, particularly related to fatty acid metabolism. Among these genes, 37 genes were found with both parameters yielding top 1% ranked values (Supplemental Tables S8), such as phosphatidylinositol 3-kinase catalytic subunit type 3 (*PIK3C3*), and patatin like phospholipase domain containing 8 (*PNPLA8*).

To infer whether selection extends beyond allelic variation and also affects gene expression, we compared individual gene expression in the brain, liver, and in breast muscle between seven wild mallards and seven domesticated ducks in natural states with RNA-seq (Supplemental Tables S9). We detected three genes (*PDC*, *MLPH*, and *NID2*) in the brain, two genes (*MAPK12* and *BST1*) in the liver, and no genes in breast muscle with significantly different expression between wild and domesticated ducks. Of the five differentially expressed genes, *PDC* was the only gene which also showed evidence of a selective sweep at the genomic level (Supplemental Tables S5, Fig. 3C - D). The results suggest that the *PDC* gene is of substantial functional importance in phenotypic differentiation among wild and domestic ducks.

## Discussion

Domesticated animals have contributed greatly to human society and human population growth by providing a stable source of animal protein, fat, and accessory products such as leather and feathers (including down).To illuminate the genetic trajectories of duck domestication, we performed whole-genome sequencing of 78 ducks including seven domesticate breeds and two wild populations. This is the first study to characterize the genetic architecture, phylogenetic relationships and domestication history of domesticated ducks and wild mallards.

Using this powerful dataset and a suite of cutting-edge population genomic

and functional genetic analyses, we observed higher mean variant numbers and nucleotide diversity for the wild mallard populations compared to the domestics, consistent with both a greater panmictic mallard population as well as recent sweeps associated with domestication.

## Population structure and domestication

We observed a large expansion of the duck population at the interglacial period, which could be the result of beneficial climatic changes, including rising temperatures and sea levels. In contrast, the glacial maximum coincided with a reduction in population size, consistent with harsher conditions and limited access to arctic breeding grounds [4, 28-30]. The demographic pattern we observe in wild ducks is similar to that observed in wild boars [5], wild yaks [31], and wild horses [32]. However, it is worth noting that although PSMC is a powerful method to infer changes in $N_e$ over time, it is also sensitive to deviations from a neutral model. The effects of genetic drift and/or selection could lead to time-dependent estimates of mutation rate, and bias our estimates of population expansion [25].

We observed three genetic clusters, with wild mallard, meat breeds, and egg/dual purpose breeds each representing unique groups. These results suggest either a single domestication event followed by subsequent breed-specific selection, or two separate domestication events. In order to distinguish alternative models of domestication, we modeled population demographics and found strong support for a single domestication event roughly 2,200 years ago,

with the rapid subsequent selection for separate meat and egg/dual purpose breeds roughly 100 generations later. Difficulty in differentiating between very recent divergence and high migration rates in the frequency spectrum prevented convergence between independent runs when trying to fit other migration parameters to our model. We note that the evolutionary history of wild mallards and domesticated duck breeds is likely to be more complex than the simple demographic scenarios modelled here, and further studies may be needed to fully capture the evolutionary dynamics of duck domestication. Given the recent origin of wild ducks, as well as the high levels of diversity we observe in the wild and domestic duck genomes, it is not possible to differentiate recent admixture from incomplete lineage sorting with our current data. This issue has important conservation implications, and represents an interesting area for future study. Nevertheless, the time estimates obtained with our model are compatible with previous written records from 500 BC [15].

## Selection for white plumage

Plumage color is an important domestication trait, and we compared breeds with white plumage to those with colored plumage. We identified high levels of divergence in the intronic region of the *MITF* gene, an important developmental locus with a complex regulation implicated in pigmentation and melanocyte development in several vertebrate species [33-35], including Japanese quail [36], dog [37], and duck [38, 39].

## Selection for other domestication traits

In order to identify those genomic regions which have been the target of selection during domestication, we used estimates of diversity between wild and domestic samples, retaining those 292 genes in the top 5% of both $F_{ST}$ and $\theta_\pi$ values for further analysis. These genes were over-represented for both neural developmental and lipid metabolism, suggesting that these functionalities were under strong selection during domestication. Two loci, *GRIK2 and GRIN3A*, showed particularly strong signs of selective sweeps presumably associated with domestication. *GRIK2* encodes a subunit of a glutamate receptor that has a role in synaptic plasticity and is important for learning and memory. *GRIN3A* encodes a subunit of the N-methyl-D-aspartate (NMDAR) receptors, which is expressed abundantly in the human cerebral cortex [40] and is involved in the development of synaptic elements

We also identified five genes with significantly different expression in the brain and liver of domesticated ducks compared to their wild ancestor. One of these, *PDC*, also showed evidence of selective sweeps at the genomic level. *PDC* encodes phosducin, a photoreceptor-specific protein highly expressed in retina and pineal gland [41], as well as the brain [42].

Our results suggest that *PDC*, *GRIK2* and *GRIN3A* may have played a crucial role in duck domestication by altering functional regulation of the developing brain and nervous system. This finding is consistent with theories that behavioral traits are the most critical in the initial steps of animal

domestication, allowing animals to tolerate humans and captivity [43, 44]. Indeed, compared to wild mallards, domestic ducks are more docile, less vigilant, and show important differences in brain morphology [17, 18]. Interestingly, differences between wild and domesticated animals in brain and nervous system functions due to directional selection were also observed in domestication studies of rabbits [7], dogs [45], and chickens [8]. In particular, *GRIK2* was also found to play a crucial role during rabbit domestication [7].

Besides brain and nervous system related genes, we also identified several genes that play an important function in lipid and energy metabolism. For example, *PIK3C3* plays an important role in ATP binding but also regulates brain development and axons of cortical neurons [46-50]. *PNPLA8* is involved in facilitating lipid storage in adipocyte tissue energy mobilization and maintains mitochondrial integrity [51, 52], as well as plays a role in lipid metabolism associated with neurodegenerative diseases [53-55]. *PRKAR2B* is associated with body weight regulation, hyperphagia, and other energy metabolism [56, 57].

Taken together, our results show that duck domestication was a relatively recent and complex process, and the genetic basis of domestication traits show many striking overlaps with other vertebrate domestication events. And, the whole genome resequencing data and SNP and INDEL variant datasets are valuable resources for researchers studying evolution, domestication or trait discovery, and for breeders of *Anas platyrhynchos.* Furthermore, the data

represent a foundation for development of new, ultrahigh density variant screening arrays for duck population level trait analysis and genomic selection.

# Methods

## Ethics statement

The entire procedure was carried out in strict accordance with the protocol approved by the Animal Welfare Committee of China Agricultural University (Permit Number: XK622).

## Sample selection

78 ducks were chosen for sequencing, seven different populations of domesticated ducks and two population of mallards from different geographic regions. The domesticated ducks include three meat type populations *i.e.*, Pekin duck (PK; n=8); Cherry Valley duck (CV; n=8); Maple Leaf duck (ML; n=8), three egg type populations *i.e.*, Jin Ding duck (JD; n=8); Shao Xing duck (SX; n=8); Shan Ma duck (SM; n=8), one egg and meat dual-purpose type (DP type) population *i.e.*, Gao You duck (GY; n=8), and two wild populations come from two different provinces in China with separated by nearly 2,000 km distance *i.e.*, Mallard from Ningxia province (MDN; n=8); Mallard form Zhejiang province (MDZ; n=14). The classification of production types follow the description of Animal Genetic Resources in China Poultry [58]. PK, CV, and ML ducks originated from Beijing; JD and SM ducks originated from Fujian province while

SX and GY ducks originated from Jiangsu province. Whole blood samples were collected from brachial veins of ducks by standard venipuncture.

In addition, 14 male ducks (MDNM, n=3; MDZM, n=4; PKM, n=1; CVM, n=1; MLM, n=1; JDM, n=1; SMM, n=1; SXM, n=1; GYM, n=1) were chosen for RNA-seq.

Sequencing and mapping statistic of individual ducks in genome and transcriptome analysis were detailed in supplementary files (Supplemental Table S1, S7).

## Sequencing and library preparation

Genomic DNA was extracted using standard phenol/chloroform extraction method. For each sample, two paired-end libraries (500 bp) were constructed according to manufacturer protocols (Illumina), and sequenced on the Illumina Hiseq 2500 sequencing platform. We sequenced each samples at 5X depth, in order to reduce the false negative rate of variants due to our strict filter criteria, we randomly selected one individual for 10X coverage, except for the MDN population, where we sequenced seven individuals at 5X coverage and random one at 20X coverage and the MDZ population, where we sequenced all individuals at 10X coverage. We generated a total of 628.37 Gb of paired-end reads of 100 bp (or 150 bp; MDZ) length (Supplemental Table S1).

mRNA from brain, liver, and breast muscle of 14 individual ducks were extracted using standard trizol extraction methods. For each samples, two

paired-end libraries (500 bp) were constructed according to manufacturer instruction (Illumina). All samples were sequenced by Illumina Hiseq 4000 sequencing platform with the coverage of 6X. We generated total of 278.62 Gb of paired-end reads of 150 bp length (Supplemental Table S9).

## Read alignment and variant calling

To avoid low quality reads, mainly the result of base-calling duplicates and adapter contamination, we filtered out sequences according to the default parameters of NGS QC Toolkit (v2.3.3) [59]. Those paired reads which passed Illumina's quality control filter were aligned using BWA-MEM (v0.7.12) to version 1.0 of the *Anas platyrhynchos* genome (BGI_duck_1.0) [10]. Duplicate reads were removed from individual samples alignments using Picard tools MarkDuplicates, and reads were merged using MergeSamFiles (http://broadinstitute.github.io/picard/).

The Genome Analysis Toolkit v3.5 (GATK, RRID:SCR_001876) RealignerTargetCreator and IndelRealigner protocol were used for global realignment of reads around INDELs before variant calling [60, 61]. SNPs and small indels (1-50 bp) were called used the GATK UnifiedGenotyper set for diploids with the parameter of minimum quality score of 20 for both mapped reads and bases to call variants, similarly to previous studies [62-66]. We filtered variants both per population and per individual using GATK according

to the stringent filtering criteria. For SNPs of population filter: a.) QUAL > 30.0; b.) QD > 5.0; c.) FS < 60.0; d.) MQ > 40.0; e.) MQRankSum > -12.5; f.) ReadPosRankSum > -8.0; Additionally, if there were more than 3 SNPs clustered in a 10 bp window, all three SNPs were considered as false positives and removed [67].

We used the following population criteria to identify INDELs: QUAL > 30.0, QD > 5.0, FS < 200.0, ReadPosRankSum > -20.0. Of individual filter, we also removed all INDELs and SNPs where the depth of derived variants was less than half the depth of the sequence. All SNPs and INDELs were assigned to specific genomic regions and genes using SnpEff v4.0 (SnpEff, RRID:SCR_005191) [68] based on the Ensembl duck annotations. After filtering a total of 36,107,949 SNPs and 3,082,731 INDELs were identified (Supplemental Table S2).

## SNP validation

In order to evaluate the reliability of our data, we compared our SNPs to the duck dbSNP database deposited in the Genome Variation Map (GVM) at the Big Data Center in the Beijing Institute of Genomics, Chinese Academy of Science (http://bigd.big.ac.cn/gvm/). 7,908,722 SNPs were validated in the duck dbSNP database, which covered 96.2% of the database (Supplemental Table S2). For the 28,199,227 SNPs not confirmed by dbSNPs, 390 randomly selected nucleotide sites were further validated diagnostic PCR combined with

Sanger sequence method described in previous researchs [8, 69, 70]. The result showed 100% accuracy, indicating the high reliability of the called SNP variation identified in this study.

## Population structure

We removed all SNPs with a minor allele frequency (MAF) <= 0.1 and kept only SNPs that occurred in more than 90% of individuals. Vcf files were converted to hapmap format with custom perl scripts, and to PLINK format file by GLU v1.0b3 (https://code.google.com/archive/p/glu-genetics/) and PLINK v1.90 (PLINK, RRID:SCR_001757) [71, 72] when appropriate. We used GCTA (v1.25) [73] for Principle Component Analysis (PCA), first by generating the genetic relationship matrix (GRM) from which the first 20 eigenvectors were extracted.

To estimate individual admixture assuming different numbers of clusters, the population structure was investigated using FRAPPE v1.1 [21] base on all high quality SNPs information, with a maximum likelihood method. We increased the coancestry clusters spanning from 2 to 4 (Supplemental figure S6), because there are four duck types (wild type, meat type, egg type, and dual-purpose type) across the nine duck populations, with 10,000 iterations per run.

A distance matrix was generated by calculating the pairwise allele sharing distance for each pair of all high quality SNPs. Multiple alignment of the

sequences was performed with MUSCLE v3.8 (MUSCLE, RRID:SCR_011812) [74]. A neighbor-joining maximum likelihood phylogenetic tree was constructed with the DNAML program in the PHYLIP package v3.69 (PHYLIP, RRID:SCR_006244) [75] and MEGA7 [76, 77]. All implementation was performed according to the recommended manipulations of SNPhylo [78].

## Demographic history reconstruction

The demographic history of both wild and domesticated ducks was inferred using a hidden Markov model approach as implemented in Pairwise Sequentially Markovian Coalescence based on SNP distributions [22]. In order to determine which PSMC (v0.6.5) settings were most appropriate for each population, we reset the number of free atomic time intervals (-p option), upper limit of time to most recent common ancestor (TMRCA) (-t option), and initial value of $r = \theta/\rho$ (-r option) according to previous research [25] and online suggestions by Li and Durbin (https://github.com/lh3/psmc). Based on estimated from the chicken genome, an average mutation rate (μ) of $1.91 \times 10^{-9}$ per base per generation and a generation time (g) of 1 year were used for analysis [79].

Three-population demographic inference was performed using a diffusion-based approach as implemented in the program ∂a∂i (v1.7) [80]. To minimize potential effects of selection that could interfere with demographic inference, these analyses were performed using the subset of noncoding regions across

the whole genome and spanning 750,939,264 bp in length. Noncoding SNPs were then thinned to 1% to alleviate potential linkage between the markers. The final dataset consisted of 95,181 SNPs with an average distance of 7,112 bp (± 18,810 bp) between neighbouring SNPs. To account for missing data, the folded allele frequency spectrum for the three populations (wild, meat and egg/dual purpose breeds) was projected down in *∂a∂i* to the projection that maximized the number of segregating SNPs, resulting in 92,966 SNPs.

We tested four different scenarios to reconstruct the demographic history of the domesticated breeds of mallards: simultaneous domestication of the meat and egg and dual purpose breeds (Model 1); a single domestication event followed by divergence of the meat and egg and dual purpose breeds (Model 2); two independent domestication events, with the meat type breed being domesticated first (Model 3); and two independent domestication events, with the egg and dual purpose breed being domesticated first (Model 4). Using the "backbone" of the best model, we then used a step-wise strategy to add parameters related with variation in population sizes and population growth, keeping a new parameter only if the Akaike information criterion (AIC) and log likelihood improved considerably over the previous model with less parameters. In cases where additional parameters resulted in negligibly improved AIC and likelihood, we retained the simpler, less parameterized model. Gene flow was modelled as continuous migration events after population divergence. Each model was run at least ten times from independent starting values to ensure

convergence to the same parameter estimates. We rejected models where we failed to obtain convergence across the replicate runs. Scaled parameters for the best-supported model were transformed into real values using the same average mutation rate ($\mu$) and ($g$) as described above for the PSMC analysis. Parameter uncertainty was obtained using the Godambe Information Matrix (GIM) [81] from 100 non-parametric bootstraps.

## Selective-sweep analysis

In order to define candidate regions having undergone directional selection during duck domestication we calculated the coefficient of nucleotide differentiation ($F_{ST}$) between mallards and domesticated ducks described by Weir & Cockerham [82]. We calculated the average $F_{ST}$ in 10kb windows with a 5 kb shift for all seven domesticated duck populations combined, and two mallard populations combined. Only scaffolds longer than 10 kb, 2368 of 78488 scaffolds, were chosen for the analysis. We transformed observed $F_{ST}$ values to Z transformation ($Z(F_{ST})$) with $\mu = 0.1154$ and $\sigma = 0.0678$ according to previously described methods [83].

To estimate levels of nucleotide diversity ($\pi$) across all sampled populations we used the VCFtools software (v0.1.13) [84] to calculate $\theta\pi(\mathrm{wild/domesticated})$ [85], computing the average difference per locus over each pair of accessions. As the measurement of $F_{ST}$, averaged $\pi$ ratio ($\theta\pi(\mathrm{wild/domesticated})$) was calculated for each scaffold in 10kb sliding

windows.

Functional classification of GO categories was performed in Database for Annotation, Visualization and Integrated Discovery (DAVID, v6.8) [86]. Statistical significance was accessed by using a modified Fisher's exact test and Benjamini correction for multiple testing.

## RNA-seq and data processing

To infer whether novel allelic variants located in the top 5% $F_{ST}$ regions of genome comparison between wild mallards and domesticated ducks could also affecting gene expression, we compared gene expression in brain, liver and in breast muscle between wild mallards and domesticated ducks. To make our result more universal, 7 male mallards and 7 male domesticated ducks were choose for RNA-seq. All samples were individually sequenced by Illumina Highseq 4000 sequencing platfrom.

For each sample, adapters and primers of paired end reads were removed by NGSQC Tool kit (v2.3.3) [59]. For each paired end read pair, if one of two reads had an average base quality less than 20 (PHRED quality score), then both reads were removed. If one end of paired end read had percentage of high quality base less than 70%, the two paired reads also removed. After that high-quality reads were mapped to reference genome using STAR (v.2.5.3a) [87]. The *featureCounts* function of the *Rsubread* (v.1.5.2) [88, 89] was used to output the counts of reads aligning to each gene. We detected the differential

expression genes with edgeR (v3.6) [90-93] using a $p_{adj} < 0.05$ threshold.

**Availability of supporting data and materials**

The 78 ducks used in whloe genome resequencing analysis and the 14 ducks used in RNA-seq analysis are accessible at NCBI under BioProject accession numbers PRJNA419832 and PRJNA419583, respectively. The unassessembled sequencing reads of 78 ducks and RNA-seq reads of 14 ducks have been deposited in NCBI Sequence Read Archive (SRA) under accession numbers SRP125660 and SRP125529, respectively. All VCF files of SNPs and INDELs and other supporting data, such as scripts, alignments for phylogenetic trees and sweep regions, are available via the *GigaScience* database *Giga*DB[94].

# Declarations

## Funding

This work was supported by the earmarked fund for the Beijing Innovation Team of the Modern Agro-industry Technology Research System (BAIC04-2017), European Research Council (grant agreement 680951) and Wolfson Merit Award. We gratefully acknowledge our colleagues in the Poultry Team at the National Engineering Laboratory for Animal Breeding of China Agricultural University, for their assistance on sample collection and helpful comments on the manuscript.

## Authors’ contributions

Conceived and designed the experiments: Lujiang Qu. Wrote the paper: Zebin Zhang. Revised the paper: Lujiang Qu, Judith E Mank, Marcel van Tuinen. Analyzed the data: Zebin Zhang, Pedro Almeida, Qiong Wang, Yaxiong Jia. Performed the experiments: Zebin Zhang, Yaxiong Jia. Contributed reagents/materials: Zhihua Jiang, Yu Chen, Kai Zhan, Shuisheng Hou, Zhengkui Zhou, Huifang Li, Fangxi Yang, Yong He, Zhonghua Ning, and Ning Yang.

# References

1. Li J and Zhang Y. Advances in research of the origin and domestication of domestic animals. Biodiversity Science. 2009;17 4:319-29.
2. Darwin C and Mayr E. On the origin of species by means of natural selection, or the preservation of favoured races in the struggle for life. john murray, london. On the Origin of Species by Means of Natural Selection. 1859.
3. Chen C, Liu Z, Pan Q, Chen X, Wang H, Guo H, et al. Genomic Analyses Reveal Demographic History and Temperate Adaptation of the Newly Discovered Honey Bee Subspecies Apis mellifera sinisxinyuan n. ssp. Mol Biol Evol. 2016;33 5:1337-48. doi:10.1093/molbev/msw017.
4. Yang J, Li WR, Lv FH, He SG, Tian SL, Peng WF, et al. Whole-Genome Sequencing of Native Sheep Provides Insights into Rapid Adaptations to Extreme Environments. Mol Biol Evol. 2016;33 10:2576-92. doi:10.1093/molbev/msw129.
5. Li M, Tian S, Jin L, Zhou G, Li Y, Zhang Y, et al. Genomic analyses identify distinct patterns of selection in domesticated pigs and Tibetan wild boars. Nat Genet. 2013;45 12:1431-8. doi:10.1038/ng.2811.
6. Jiang Y, Xie M, Chen W, Talbot R, Maddox JF, Faraut T, et al. The sheep genome illuminates biology of the rumen and lipid metabolism. Science. 2014;344 6188:1168-73. doi:10.1126/science.1252806.
7. Carneiro M, Rubin CJ, Di Palma F, Albert FW, Alfoldi J, Barrio AM, et al. Rabbit genome analysis reveals a polygenic basis for

phenotypic change during domestication. Science. 2014;345 6200:1074-9. doi:10.1126/science.1253714.

8. Wang MS, Zhang RW, Su LY, Li Y, Peng MS, Liu HQ, et al. Positive selection rather than relaxation of functional constraint drives the evolution of vision during chicken domestication. Cell Res. 2016;26 5:556-73. doi:10.1038/cr.2016.44.

9. Rubin CJ, Zody MC, Eriksson J, Meadows JR, Sherwood E, Webster MT, et al. Whole-genome resequencing reveals loci under selection during chicken domestication. Nature. 2010;464 7288:587-91. doi:10.1038/nature08832.

10. Huang Y, Li Y, Burt DW, Chen H, Zhang Y, Qian W, et al. The duck genome and transcriptome provide insight into an avian influenza virus reservoir species. Nat Genet. 2013;45 7:776-83. doi:10.1038/ng.2657.

11. Zeuner FE. A history of domesticated animals. A history of domesticated animals. 1963.

12. Thomson SAL, Ornithologists' Union B and Thomson AL. A new dictionary of birds. Nelson London; 1964.

13. Mason IL and Mason IL. Evolution of domesticated animals. 1984.

14. Bray F and Needham J. Science and Civilization in China, vol. 6, part 1. Agriculture: Cambridge University Press, Cambridge, UK. 1984.

15. Kiple KF. The Cambridge world history of food. Cambridge: Cambridge University Press; 2000.

16. Chang H. Conspectus of genetic resources of livestock. Chinese Agriculture Press, Beijing, China, 1995.

17. Miller DB. Social displays of Mallard Ducks (Anas platyrhynchos): effects of domestication. Journal of Comparative and Physiological Psychology. 1977;91 2:221.

18. Ebinger P. Domestication and plasticity of brain organization in mallards (Anas platyrhynchos). Brain, behavior and evolution. 1995;45 5:286-300.

19. Frahm H, Rehkämper G and Werner C. Brain alterations in crested versus non-crested breeds of domestic ducks (Anas platyrhynchos fd). Poultry science. 2001;80 9:1249-57.

20. Duggan BM, Hocking PM, Schwarz T and Clements DN. Differences in hindlimb morphology of ducks and chickens: effects of domestication and selection. Genetics Selection Evolution. 2015;47 1:88.

21. Tang H, Peng J, Wang P and Risch NJ. Estimation of individual admixture: analytical and study design considerations. Genet Epidemiol. 2005;28 4:289-301. doi:10.1002/gepi.20064.

22. Li H and Durbin R. Inference of human population history from individual whole-genome sequences. Nature. 2011;475 7357:493-6. doi:10.1038/nature10231.

23. Ehlers J and Gibbard PL. The extent and chronology of Cenozoic global glaciation. Quaternary International. 2007;164:6-20.

24. Williams MAJ, Dunkerley D, De Deckker P, Kershaw AP and Stokes T. Quaternary environments. Science Press; 1997.

25. Nadachowska-Brzyska K, Li C, Smeds L, Zhang G and Ellegren H. Temporal Dynamics of Avian Populations during Pleistocene Revealed by Whole-Genome Sequences. Curr Biol. 2015;25 10:1375-80. doi:10.1016/j.cub.2015.03.047.

26. Shapiro MD, Kronenberg Z, Li C, Domyan ET, Pan H, Campbell M, et al. Genomic diversity and evolution of the head crest in the rock pigeon. Science. 2013;339 6123:1063-7. doi:10.1126/science.1230422.

27. Price TD. Domesticated birds as a model for the genetics of speciation by sexual selection. Genetica. 2002;116 2-3:311-27. doi:Doi 10.1023/A:1021248913179.

28. Lorenzen ED, Nogués-Bravo D, Orlando L, Weinstock J, Binladen J, Marske KA, et al. Species-specific responses of Late Quaternary megafauna to climate and humans. Nature. 2011;479 7373:359-64.

29. Hewitt G. The genetic legacy of the Quaternary ice ages. Nature. 2000;405 6789:907-13.

30. Hewitt G. Genetic consequences of climatic oscillations in the Quaternary. Philosophical Transactions of the Royal Society of London B: Biological Sciences. 2004;359 1442:183-95.

31. Qiu Q, Wang L, Wang K, Yang Y, Ma T, Wang Z, et al. Yak whole-genome resequencing reveals domestication signatures and prehistoric population expansions. Nature communications. 2015;6:10283. doi:10.1038/ncomms10283.

32. Orlando L, Ginolhac A, Zhang G, Froese D, Albrechtsen A, Stiller M, et al. Recalibrating Equus evolution using the genome sequence of an early Middle Pleistocene horse. Nature. 2013;499 7456:74-8. doi:10.1038/nature12323.

33. Steingrimsson E, Copeland NG and Jenkins NA. Melanocytes and the microphthalmia transcription factor network. Annual review of genetics. 2004;38:365-411. doi:10.1146/annurev.genet.38.072902.092717.

34. Hallsson JH, Haflidadottir BS, Schepsky A, Arnheiter H and Steingrimsson E. Evolutionary sequence comparison of the Mitf gene reveals novel conserved domains. Pigment cell research. 2007;20 3:185-200. doi:10.1111/j.1600-0749.2007.00373.x.

35. Levy C, Khaled M and Fisher DE. MITF: master regulator of melanocyte development and melanoma oncogene. Trends in molecular medicine. 2006;12 9:406-14. doi:10.1016/j.molmed.2006.07.008.

36. Minvielle F, Bed'hom B, Coville JL, Ito S, Inoue-Murayama M and Gourichon D. The "silver" Japanese quail and the MITF gene: causal

mutation, associated traits and homology with the "blue" chicken plumage. BMC genetics. 2010;11:15. doi:10.1186/1471-2156-11-15.

37. Karlsson EK, Baranowska I, Wade CM, Salmon Hillbertz NH, Zody MC, Anderson N, et al. Efficient mapping of mendelian traits in dogs through genome-wide association. Nat Genet. 2007;39 11:1321-8. doi:10.1038/ng.2007.10.

38. Li S, Wang C, Yu W, Zhao S and Gong Y. Identification of genes related to white and black plumage formation by RNA-Seq from white and black feather bulbs in ducks. PLoS One. 2012;7 5:e36592. doi:10.1371/journal.pone.0036592.

39. Sultana H, Seo D, Choi NR, Bhuiyan MSA, Lee SH, Heo KN, et al. Identification of Polymorphisms in MITF and DCT Genes and their Associations with Plumage Colors in Asian Duck Breeds. Asian-Australasian journal of animal sciences. 2017; doi:10.5713/ajas.17.0298.

40. Eriksson M, Nilsson A, Samuelsson H, Samuelsson EB, Mo L, Akesson E, et al. On the role of NR3A in human NMDA receptors. Physiology & behavior. 2007;92 1-2:54-9. doi:10.1016/j.physbeh.2007.05.026.

41. Bauer PH, Muller S, Puzicha M, Pippig S, Obermaier B, Helmreich EJM, et al. Phosducin Is a Protein Kinase-a-Regulated G-Protein Regulator. Nature. 1992;358 6381:73-6. doi:Doi 10.1038/358073a0.

42. Sunayashiki-Kusuzaki K, Kikuchi T, Wawrousek EF and Shinohara T. Arrestin and phosducin are expressed in a small number of brain cells. Brain research Molecular brain research. 1997;52 1:112-20.

43. Mignon-Grasteau S, Boissy A, Bouix J, Faure J-M, Fisher AD, Hinch GN, et al. Genetics of adaptation and domestication in livestock. Livestock Production Science. 2005;93 1:3-14.

44. Dugatkin LA and Trut L. How to Tame a Fox (and Build a Dog): Visionary Scientists and a Siberian Tale of Jump-Started Evolution. University of Chicago Press; 2017.

45. Axelsson E, Ratnakumar A, Arendt ML, Maqbool K, Webster MT, Perloski M, et al. The genomic signature of dog domestication reveals adaptation to a starch-rich diet. Nature. 2013;495 7441:360-4. doi:10.1038/nature11837.

46. Volinia S, Dhand R, Vanhaesebroeck B, MacDougall L, Stein R, Zvelebil M, et al. A human phosphatidylinositol 3-kinase complex related to the yeast Vps34p-Vps15p protein sorting system. The EMBO Journal. 1995;14 14:3339.

47. Inaguma Y, Ito H, Iwamoto I, Matsumoto A, Yamagata T, Tabata H, et al. Morphological characterization of Class III phosphoinositide 3-kinase during mouse brain development. Medical molecular morphology. 2016;49 1:28-33. doi:10.1007/s00795-015-0116-1.

48. Stopkova P, Saito T, Papolos DF, Vevera J, Paclt I, Zukov I, et al. Identification of PIK3C3 promoter variant associated with bipolar disorder and schizophrenia. Biol Psychiatry. 2004;55 10:981-8. doi:10.1016/j.biopsych.2004.01.014.

49. Tang R, Zhao X, Fang C, Tang W, Huang K, Wang L, et al. Investigation of variants in the promoter region of PIK3C3 in schizophrenia. Neuroscience letters. 2008;437 1:42-4. doi:10.1016/j.neulet.2008.03.043.

50. Zhou X, Wang L, Hasegawa H, Amin P, Han B-X, Kaneko S, et al. Deletion of PIK3C3/Vps34 in sensory neurons causes rapid neurodegeneration by disrupting the endosomal but not the autophagic pathway. Proceedings of the National Academy of Sciences. 2010;107 20:9424-9.

51. Wilson PA, Gardner SD, Lambie NM, Commans SA and Crowther DJ. Characterization of the human patatin-like phospholipase family. Journal of lipid research. 2006;47 9:1940-9.

52. Kienesberger PC, Oberer M, Lass A and Zechner R. Mammalian patatin domain containing proteins: a family with diverse lipolytic activities involved in multiple biological functions. Journal of lipid research. 2009;50 Supplement:S63-S8.

53. Tesson C, Nawara M, Salih MA, Rossignol R, Zaki MS, Al Balwi M, et al. Alteration of fatty-acid-metabolizing enzymes affects mitochondrial form and function in hereditary spastic paraplegia. The American Journal of Human Genetics. 2012;91 6:1051-64.

54. Schuurs-Hoeijmakers JH, Oh EC, Vissers LE, Swinkels ME, Gilissen C, Willemsen MA, et al. Recurrent de novo mutations in PACS1 cause defective cranial-neural-crest migration and define a recognizable intellectual-disability syndrome. The American Journal of Human Genetics. 2012;91 6:1122-7.

55. Martin E, Schüle R, Smets K, Rastetter A, Boukhris A, Loureiro JL, et al. Loss of function of glucocerebrosidase GBA2 is responsible for motor neuron defects in hereditary spastic paraplegia. The American Journal of Human Genetics. 2013;92 2:238-44.

56. Gagliano SA, Tiwari AK, Freeman N, Lieberman JA, Meltzer HY, Kennedy JL, et al. Protein kinase cAMP-dependent regulatory type II beta (PRKAR2B) gene variants in antipsychotic-induced weight gain. Human psychopharmacology. 2014;29 4:330-5. doi:10.1002/hup.2407.

57. Czyzyk TA, Sikorski MA, Yang L and McKnight GS. Disruption of the RIIβ subunit of PKA reverses the obesity syndrome of agouti lethal yellow mice. Proceedings of the National Academy of Sciences. 2008;105 1:276-81.

58. Resources CNCoAG. Animal genetic resources in China poultry. Beijing: China Agriculture Press; 2010.

59. Patel RK and Jain M. NGS QC Toolkit: a toolkit for quality

control of next generation sequencing data. PLoS One. 2012;7 2:e30619. doi:10.1371/journal.pone.0030619.

60. McKenna A, Hanna M, Banks E, Sivachenko A, Cibulskis K, Kernytsky A, et al. The Genome Analysis Toolkit: a MapReduce framework for analyzing next-generation DNA sequencing data. Genome Res. 2010;20 9:1297-303. doi:10.1101/gr.107524.110.

61. DePristo MA, Banks E, Poplin R, Garimella KV, Maguire JR, Hartl C, et al. A framework for variation discovery and genotyping using next-generation DNA sequencing data. Nat Genet. 2011;43 5:491-8. doi:10.1038/ng.806.

62. Yan Y, Yi G, Sun C, Qu L and Yang N. Genome-wide characterization of insertion and deletion variation in chicken using next generation sequencing. PLoS One. 2014;9 8:e104652. doi:10.1371/journal.pone.0104652.

63. Qu Y, Tian S, Han N, Zhao H, Gao B, Fu J, et al. Genetic responses to seasonal variation in altitudinal stress: whole-genome resequencing of great tit in eastern Himalayas. Sci Rep. 2015;5:14256. doi:10.1038/srep14256.

64. Meyer RS, Choi JY, Sanches M, Plessis A, Flowers JM, Amas J, et al. Domestication history and geographical adaptation inferred from a SNP map of African rice. Nat Genet. 2016;48 9:1083-8. doi:10.1038/ng.3633.

65. Russell J, Mascher M, Dawson IK, Kyriakidis S, Calixto C, Freund F, et al. Exome sequencing of geographically diverse barley landraces and wild relatives gives insights into environmental adaptation. Nat Genet. 2016;48 9:1024-30. doi:10.1038/ng.3612.

66. Mascher M, Schuenemann VJ, Davidovich U, Marom N, Himmelbach A, Hubner S, et al. Genomic analysis of 6,000-year-old cultivated grain illuminates the domestication history of barley. Nat Genet. 2016;48 9:1089-93. doi:10.1038/ng.3611.

67. Li H, Ruan J and Durbin R. Mapping short DNA sequencing reads and calling variants using mapping quality scores. Genome Res. 2008;18 11:1851-8. doi:10.1101/gr.078212.108.

68. Cingolani P, Platts A, Wang LL, Coon M, Nguyen T, Wang L, et al. A program for annotating and predicting the effects of single nucleotide polymorphisms, SnpEff: SNPs in the genome of Drosophila melanogaster strain w1118; iso-2; iso-3. Fly. 2012;6 2:80-92.

69. Zhang Z, Nie C, Jia Y, Jiang R, Xia H, Lv X, et al. Parallel Evolution of Polydactyly Traits in Chinese and European Chickens. PloS one. 2016;11 2:e0149010.

70. Van Tassell CP, Smith TP, Matukumalli LK, Taylor JF, Schnabel RD, Lawley CT, et al. SNP discovery and allele frequency estimation by deep sequencing of reduced representation libraries. Nature methods. 2008;5 3:247-52. doi:10.1038/nmeth.1185.

71. Purcell S, Neale B, Todd-Brown K, Thomas L, Ferreira MA, Bender D, et al. PLINK: a tool set for whole-genome association and population-based linkage analyses. The American Journal of Human Genetics. 2007;81 3:559-75.

72. Chang CC, Chow CC, Tellier LC, Vattikuti S, Purcell SM and Lee JJ. Second-generation PLINK: rising to the challenge of larger and richer datasets. Gigascience. 2015;4 1:7.

73. Yang J, Lee SH, Goddard ME and Visscher PM. GCTA: a tool for genome-wide complex trait analysis. The American Journal of Human Genetics. 2011;88 1:76-82.

74. Edgar RC. MUSCLE: multiple sequence alignment with high accuracy and high throughput. Nucleic acids research. 2004;32 5:1792-7.

75. Plotree D and Plotgram D. PHYLIP-phylogeny inference package (version 3.2). cladistics. 1989;5 163:6.

76. Tamura K, Dudley J, Nei M and Kumar S. MEGA4: molecular evolutionary genetics analysis (MEGA) software version 4.0. Molecular biology and evolution. 2007;24 8:1596-9.

77. Kumar S, Stecher G and Tamura K. MEGA7: Molecular Evolutionary Genetics Analysis Version 7.0 for Bigger Datasets. Mol Biol Evol. 2016;33 7:1870-4. doi:10.1093/molbev/msw054.

78. Lee TH, Guo H, Wang X, Kim C and Paterson AH. SNPhylo: a pipeline to construct a phylogenetic tree from huge SNP data. BMC genomics. 2014;15 1:162. doi:10.1186/1471-2164-15-162.

79. Nam K, Mugal C, Nabholz B, Schielzeth H, Wolf JB, Backström N, et al. Molecular evolution of genes in avian genomes. Genome biology. 2010;11 6:R68.

80. Gutenkunst RN, Hernandez RD, Williamson SH and Bustamante CD. Inferring the joint demographic history of multiple populations from multidimensional SNP frequency data. PLoS genetics. 2009;5 10:e1000695. doi:10.1371/journal.pgen.1000695.

81. Coffman AJ, Hsieh PH, Gravel S and Gutenkunst RN. Computationally Efficient Composite Likelihood Statistics for Demographic Inference. Molecular Biology and Evolution. 2016;33 2:591-3. doi:10.1093/molbev/msv255.

82. Weir BS and Cockerham CC. Estimating F-Statistics for the Analysis of Population-Structure. Evolution. 1984;38 6:1358-70. doi:Doi 10.2307/2408641.

83. Kreyszig E. Advanced engineering mathematics. John Wiley & Sons; 2007.

84. Danecek P, Auton A, Abecasis G, Albers CA, Banks E, DePristo MA, et al. The variant call format and VCFtools. Bioinformatics. 2011;27 15:2156-8.

85. Tajima F. Evolutionary relationship of DNA sequences in finite

populations. Genetics. 1983;105 2:437-60.

86. Huang da W, Sherman BT and Lempicki RA. Systematic and integrative analysis of large gene lists using DAVID bioinformatics resources. Nature protocols. 2009;4 1:44-57. doi:10.1038/nprot.2008.211.

87. Dobin A, Davis CA, Schlesinger F, Drenkow J, Zaleski C, Jha S, et al. STAR: ultrafast universal RNA-seq aligner. Bioinformatics. 2013;29 1:15-21.

88. Liao Y, Smyth GK and Shi W. The Subread aligner: fast, accurate and scalable read mapping by seed-and-vote. Nucleic acids research. 2013;41 10:e108-e.

89. Liao Y, Smyth GK and Shi W. featureCounts: an efficient general purpose program for assigning sequence reads to genomic features. Bioinformatics. 2014;30 7:923-30. doi:10.1093/bioinformatics/btt656.

90. Robinson MD and Smyth GK. Moderated statistical tests for assessing differences in tag abundance. Bioinformatics. 2007;23 21:2881-7. doi:10.1093/bioinformatics/btm453.

91. Robinson MD, McCarthy DJ and Smyth GK. edgeR: a Bioconductor package for differential expression analysis of digital gene expression data. Bioinformatics. 2010;26 1:139-40. doi:10.1093/bioinformatics/btp616.

92. McCarthy DJ, Chen Y and Smyth GK. Differential expression analysis of multifactor RNA-Seq experiments with respect to biological variation. Nucleic acids research. 2012;40 10:4288-97. doi:10.1093/nar/gks042.

93. Lun AT, Chen Y and Smyth GK. It’s DE-licious: a recipe for differential expression analyses of RNA-seq experiments using quasi-likelihood methods in edgeR. Statistical Genomics: Methods and Protocols. 2016:391-416.

94. Zhang Z, Jia Y, Almeida P, Mank JE, Tuinen MV, Wang Q et al. Supporting data for "Whole-genome resequencing reveals signatures of selection and timing of duck domestication". GigaScience database 2018. http://dx.doi.org/10.5524/100417

Figure 1

Click here to download Figure Fig. 1.pdf

A

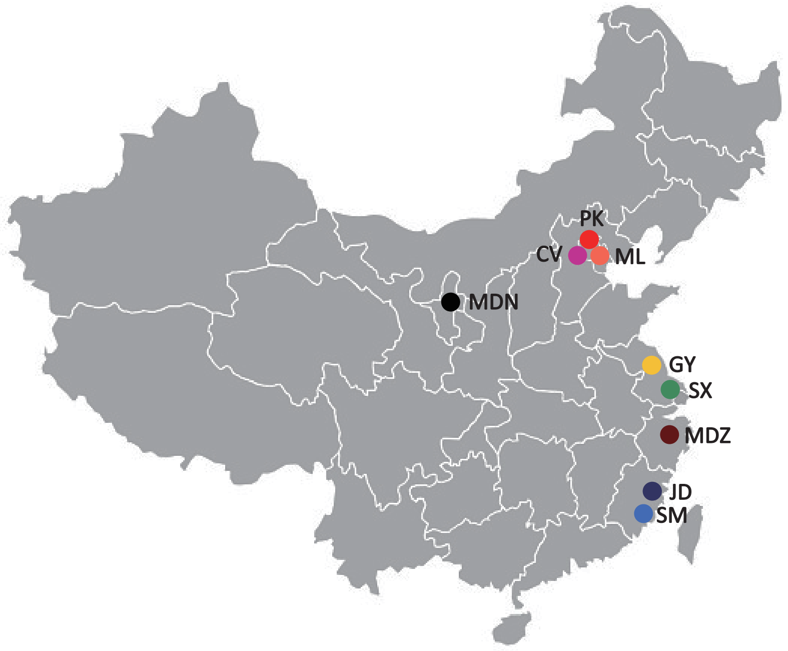


B

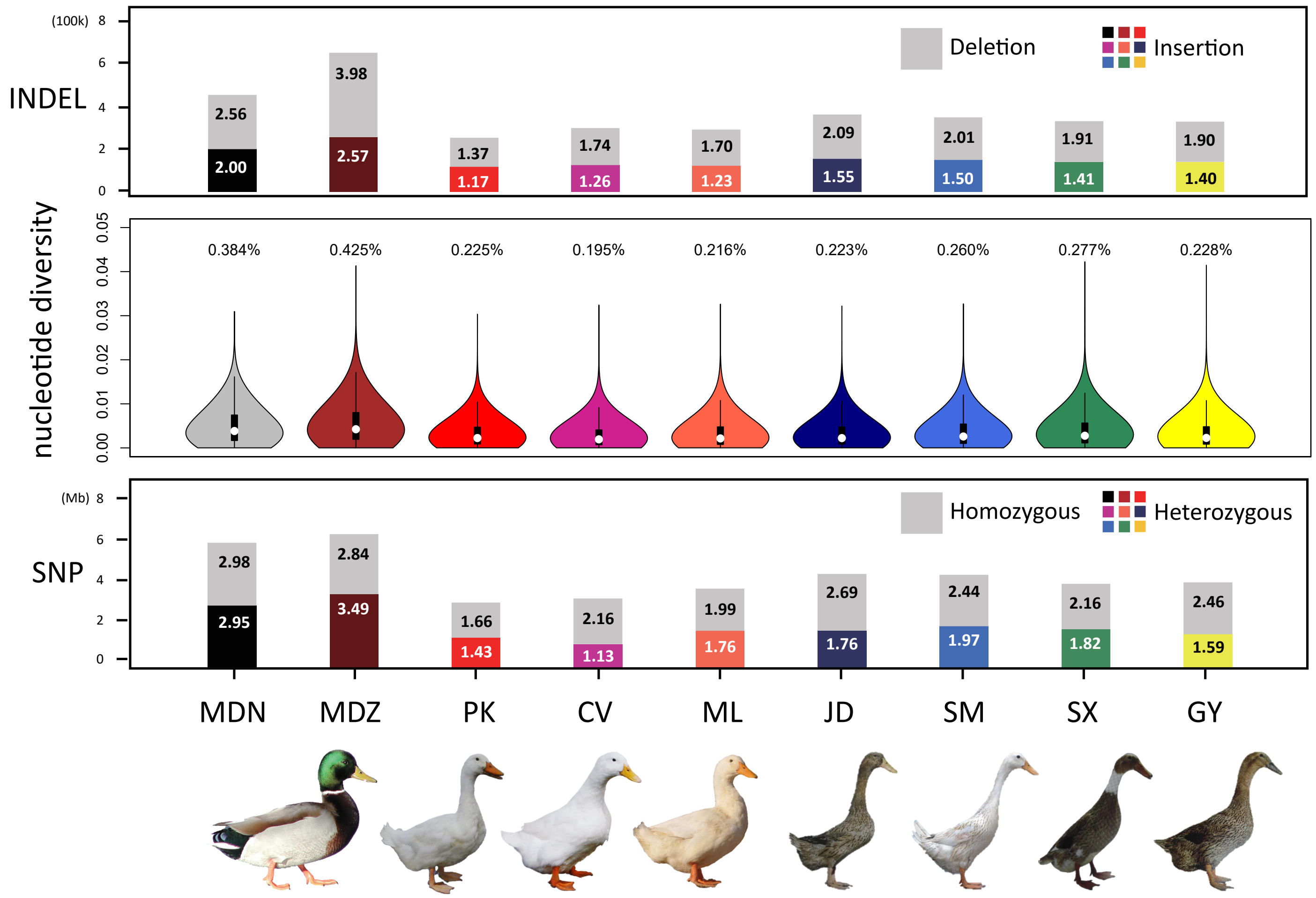

Figure 2

Click here to download Figure Fig. 2.pdf

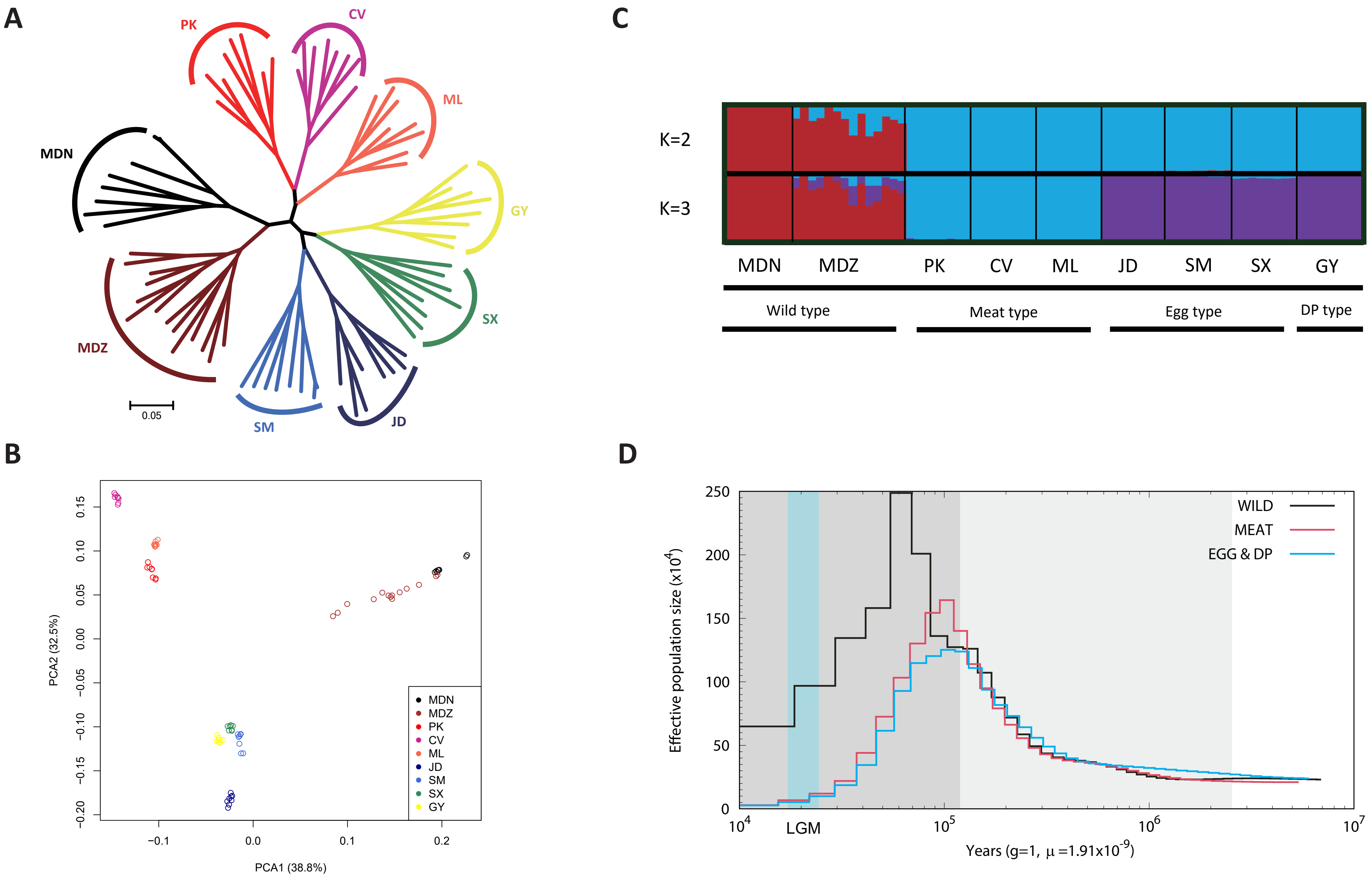

Figure 3

Click here to download Figure Fig. 3.pdf

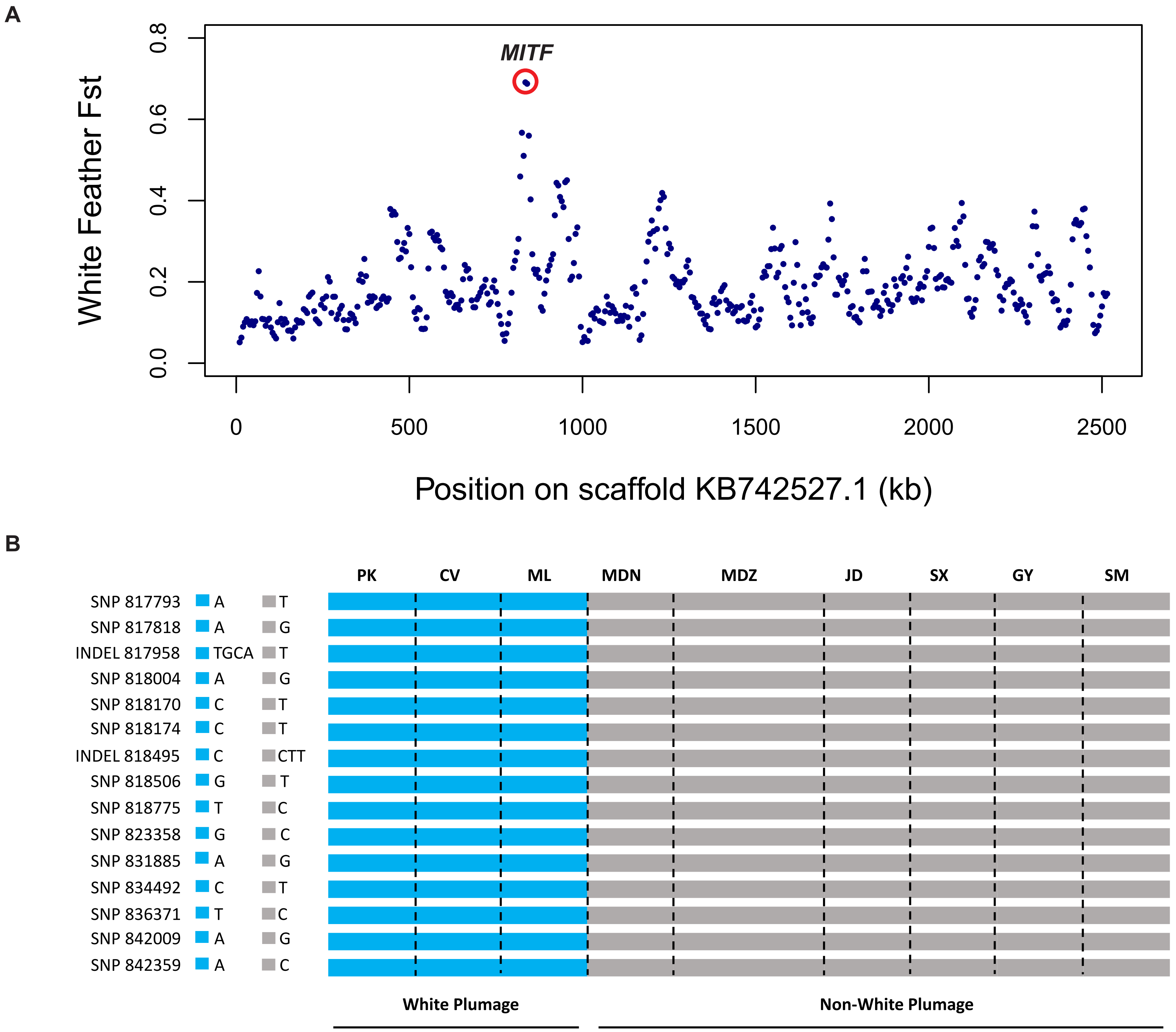

Figure 4

Click here to download Figure fig. 4.pdf

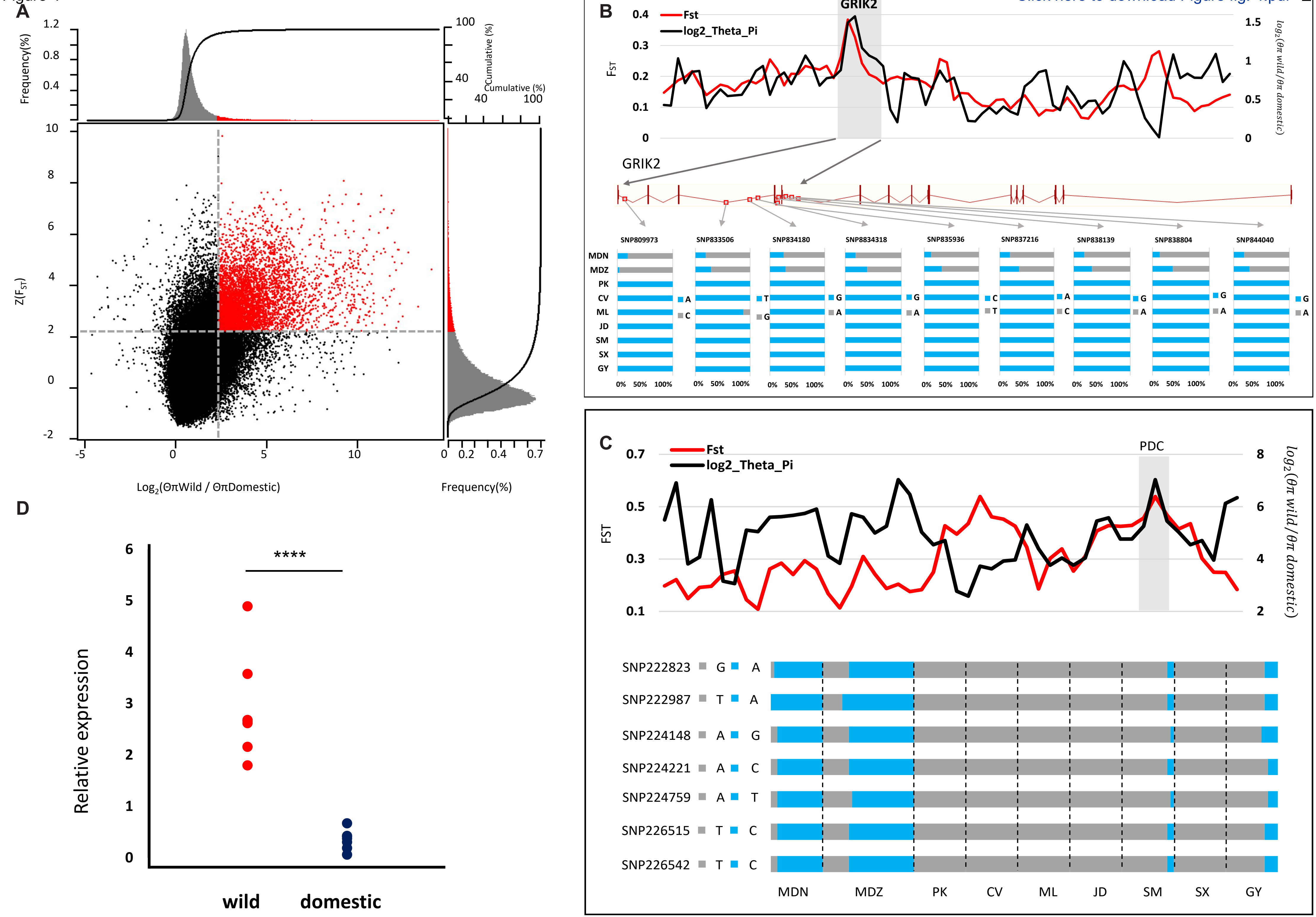

Supplementary Figure S1

Click here to access/download
**Supplementary Material**
supplemental Figure S1.pdf

Supplementary Figure S2

Click here to access/download
**Supplementary Material**
supplemental Figure S2.pdf

Supplementary Figure S3

Click here to access/download
**Supplementary Material**
Supplemental Figure S3.pdf

Supplementary Figure S4

Click here to access/download
**Supplementary Material**
Supplemental Figure S4.pdf

Supplementary Figure S5

Click here to access/download
**Supplementary Material**
Supplemental Figure S5.pdf

Supplementary Figure S6

Click here to access/download
**Supplementary Material**
Supplemental Figure S6.pdf

Click here to access/download
**Supplementary Material**
Supplementary Tables.xlsx
