# Supplementary material for: Whole-genome resequencing reveals signatures of selection and timing of duck domestication
Source: Gigascience. 2018 Apr 9;7(4):giy027. doi: 10.1093/gigascience/giy027 (PMC6007426; doi:10.1093/gigascience/giy027)

Figure S1: Distribution of variants in functional regions.
SNPs distribution were showed on the left, and INDELs were showed on right. Most variants were synonymous mutations both in SNPs and in INDELs at genome wide across all populations.

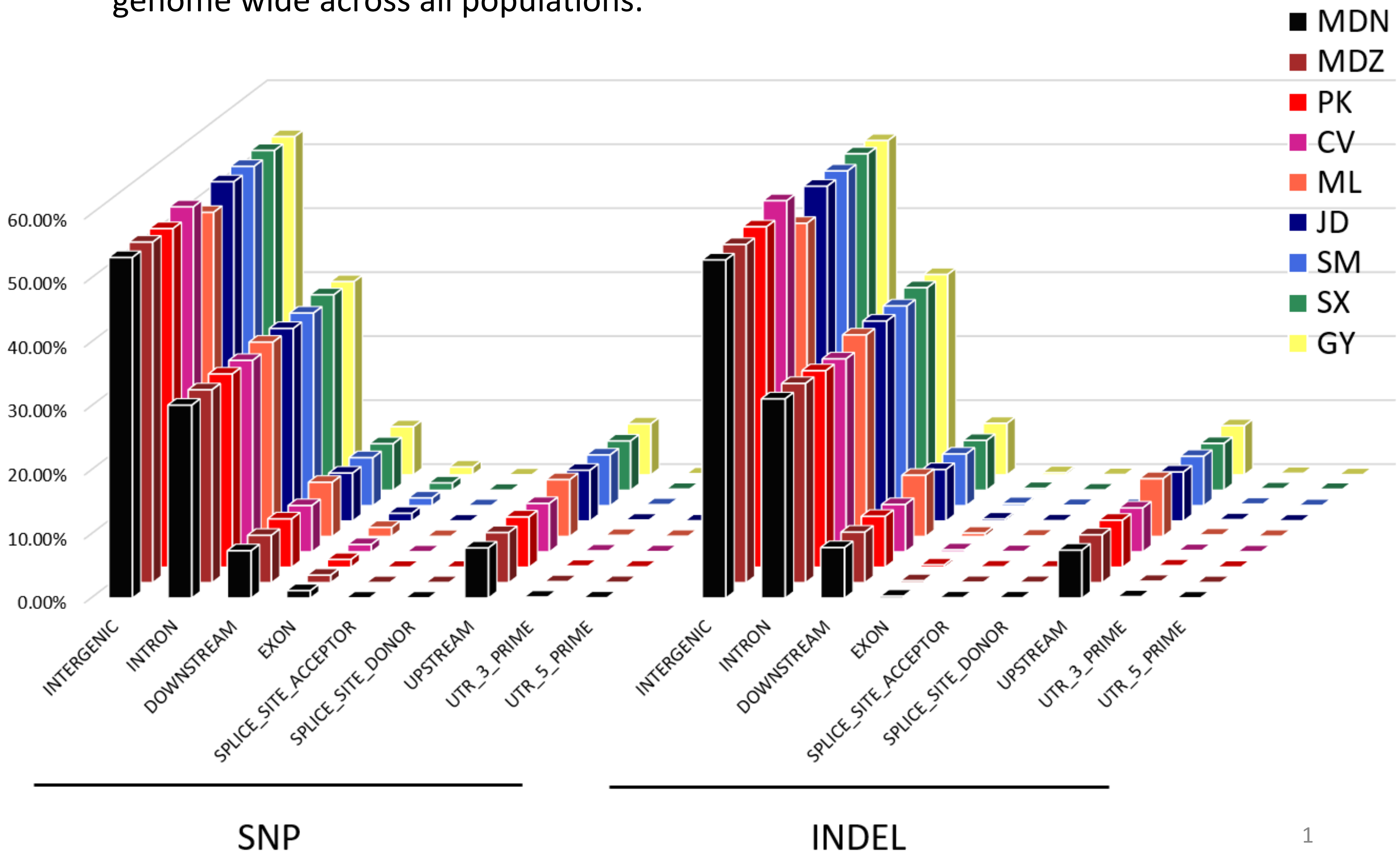

Supplement: Supplemental material [file giy027_supp.zip › supplemental Figure S1.pdf]
