# Supplementary material for: Whole-genome resequencing reveals signatures of selection and timing of duck domestication
Source: Gigascience. 2018 Apr 9;7(4):giy027. doi: 10.1093/gigascience/giy027 (PMC6007426; doi:10.1093/gigascience/giy027)

Figure S2: INDELs statistics of 9 population ducks.

The largest INDEL detected in this study was 50 bp, and the majority of INDELs were less than 10 bp. Single base-pair INDEL was the predominant form and accounted for 38.63% of all detected INDELs. Both count and percentage were mean value of 9 population ducks.

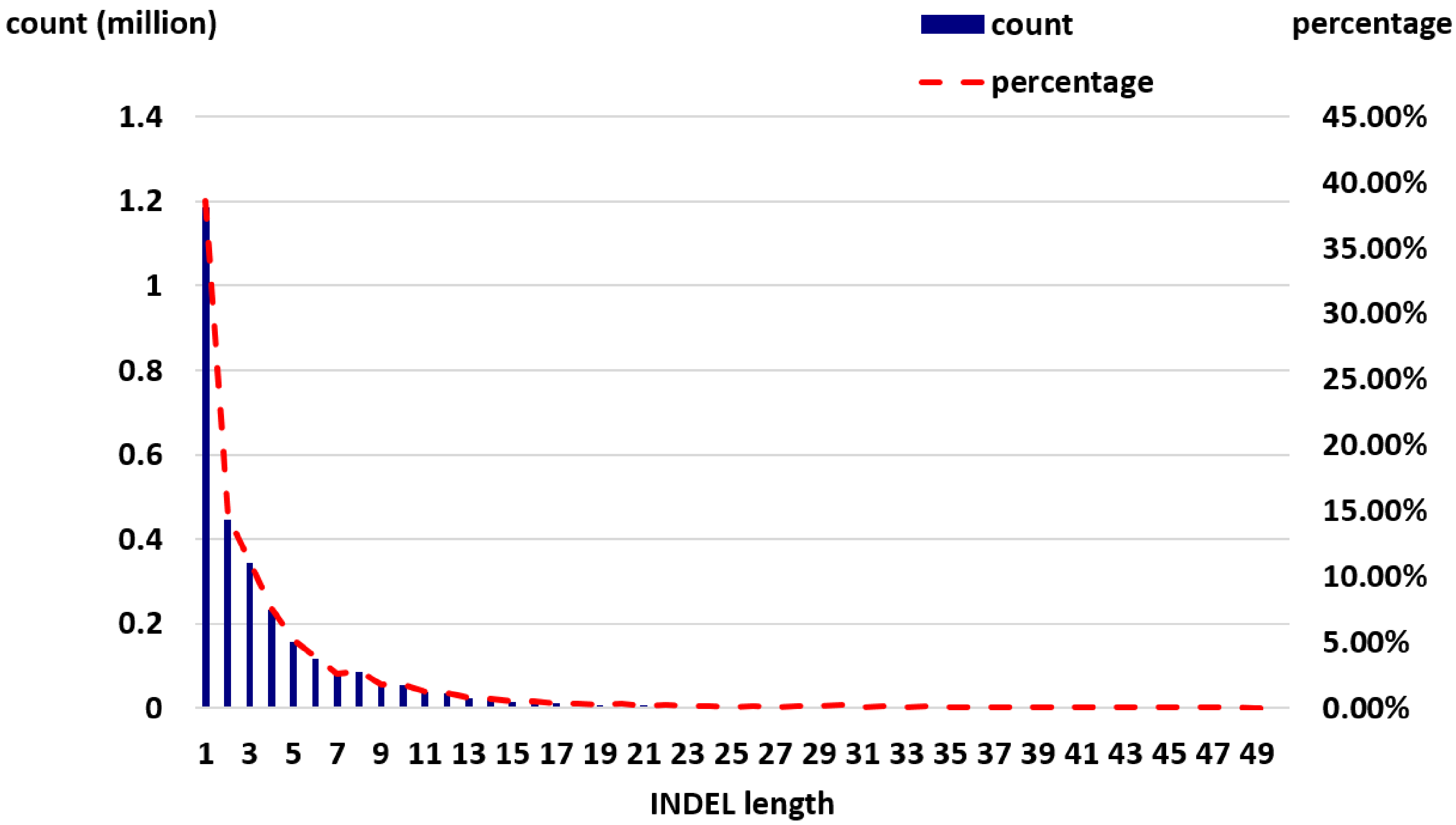

Supplement: Supplemental material [file giy027_supp.zip › supplemental Figure S2.pdf]
