# Supplementary material for: Whole-genome resequencing reveals signatures of selection and timing of duck domestication
Source: Gigascience. 2018 Apr 9;7(4):giy027. doi: 10.1093/gigascience/giy027 (PMC6007426; doi:10.1093/gigascience/giy027)
Supplement: Supplemental material [file giy027_supp.zip › Supplemental Figure S3.pdf]

Supplemental Figure S3

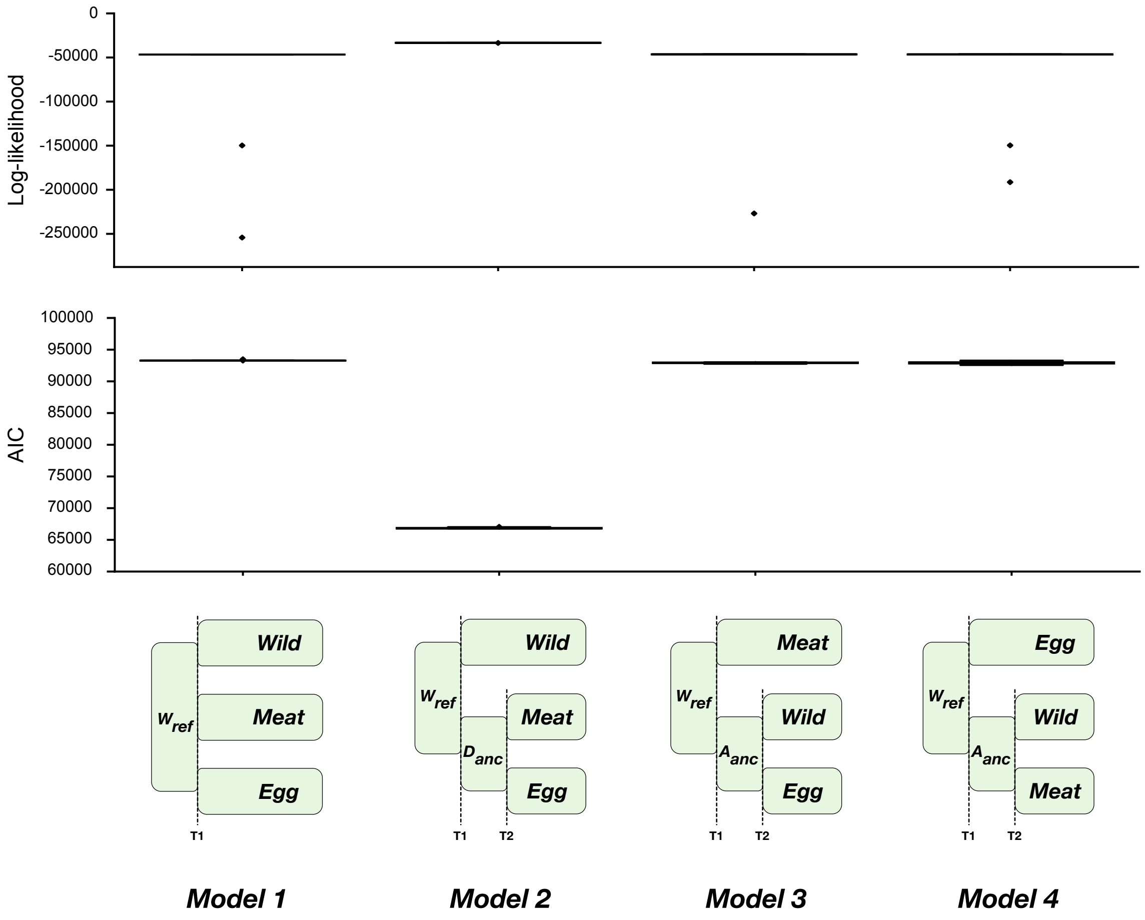

Supplemental Figure S3. Comparison of four demographic models for the domestication of meat and egg/dual purpose breeds of mallards using ∂a∂i. The top panel shows the distribution of the log-likelihood for each one of the tested models and the middle panel the distribution of the Akaike information criterion (AIC) with outliers excluded. *Model 1*: simultaneous domestication of the meat and egg and dual purpose breeds; *Model 2*: a single domestication event followed by divergence of the meat and egg and dual purpose breeds; *Model 3*: two independent domestication events, with the meat type breed being domesticated first; *Model 4*: two independent domestication events, with the egg and dual purpose breed being domesticated first.
