# Supplementary material for: Whole-genome resequencing reveals signatures of selection and timing of duck domestication
Source: Gigascience. 2018 Apr 9;7(4):giy027. doi: 10.1093/gigascience/giy027 (PMC6007426; doi:10.1093/gigascience/giy027)
Supplement: Supplemental material [file giy027_supp.zip › Supplemental Figure S4.pdf]

Supplemental Figure S4

(A)

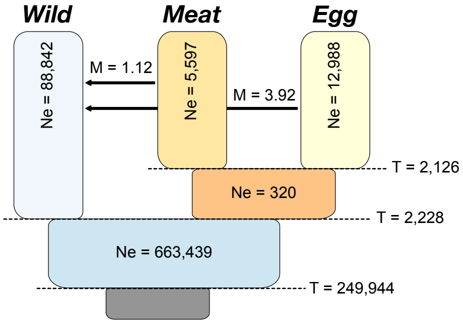

(B)

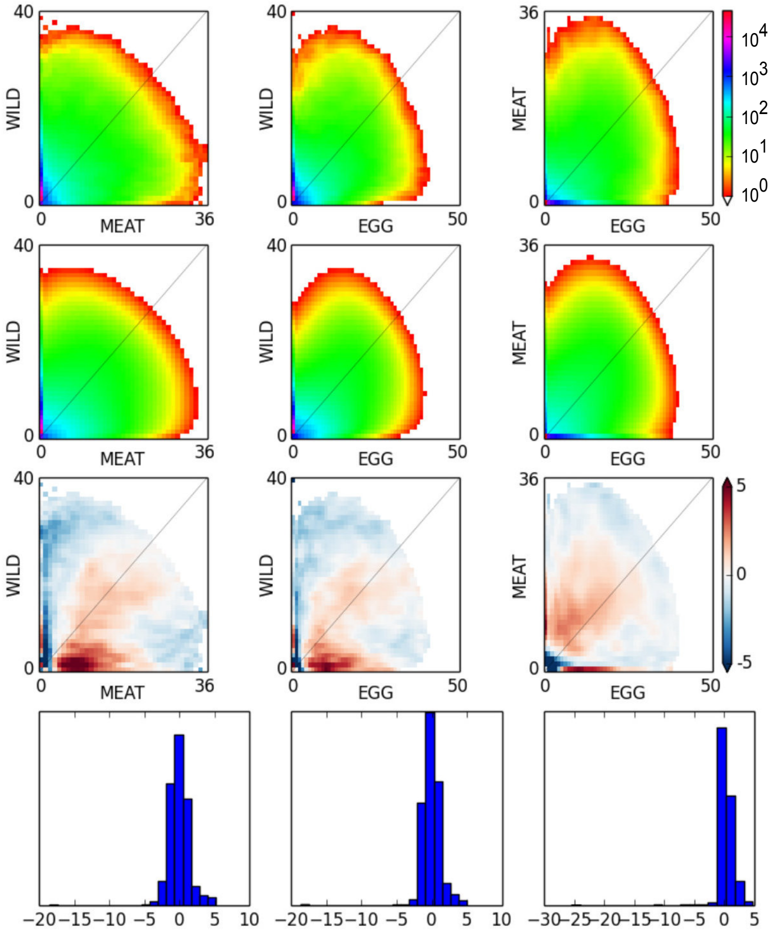

Supplemental Figure S4. Demographic history of meat and egg/dual purpose breed domestication using the best fit model inferred by ∂a∂i. **(A)** Model of single domestication event with changes in population sizes and migration. Time units are in years before present and migration are in units of number of migrants per generation. **(B)** Site frequency spectrum for the three populations of domesticated and wild mallards. The frequency spectrum is shown for the data (first row) and for the best fit model (second row). The last two rows show the normalized difference (*i.e.* residuals) between model and data for each bin in the spectrum.
