# Supplementary material for: Whole-genome resequencing reveals signatures of selection and timing of duck domestication
Source: Gigascience. 2018 Apr 9;7(4):giy027. doi: 10.1093/gigascience/giy027 (PMC6007426; doi:10.1093/gigascience/giy027)
Supplement: Supplemental material [file giy027_supp.zip › Supplemental Figure S5.pdf]

Supplemental Figure S5

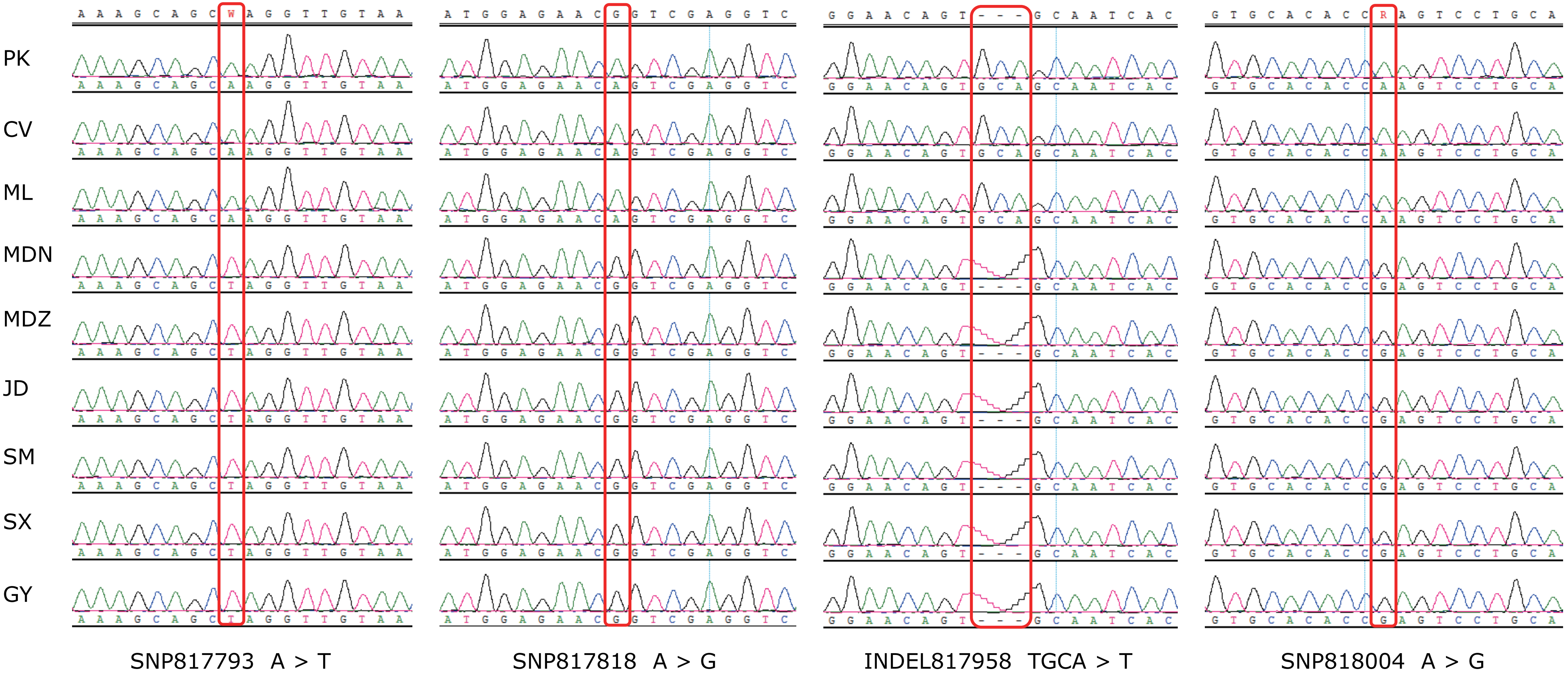

supplemental Figure S5. White plumage related variants of *MITF* validation by Sanger sequence in 78 ducks. Three SNPs and one INDEL of *MITF* was amplified by diagnostic PCR and sequenced by Sanger method, resulted completely matched with the analysis rusult of NGS. White plumage ducks contains PK, CV, and ML; non-white plumage ducks contains MDN, MDZ, JD, SM, SX, and GY.
