# Supplementary material for: Whole-genome resequencing reveals signatures of selection and timing of duck domestication
Source: Gigascience. 2018 Apr 9;7(4):giy027. doi: 10.1093/gigascience/giy027 (PMC6007426; doi:10.1093/gigascience/giy027)
Supplement: Supplemental material [file giy027_supp.zip › Supplemental Figure S6.pdf]

Supplemental Figure S6

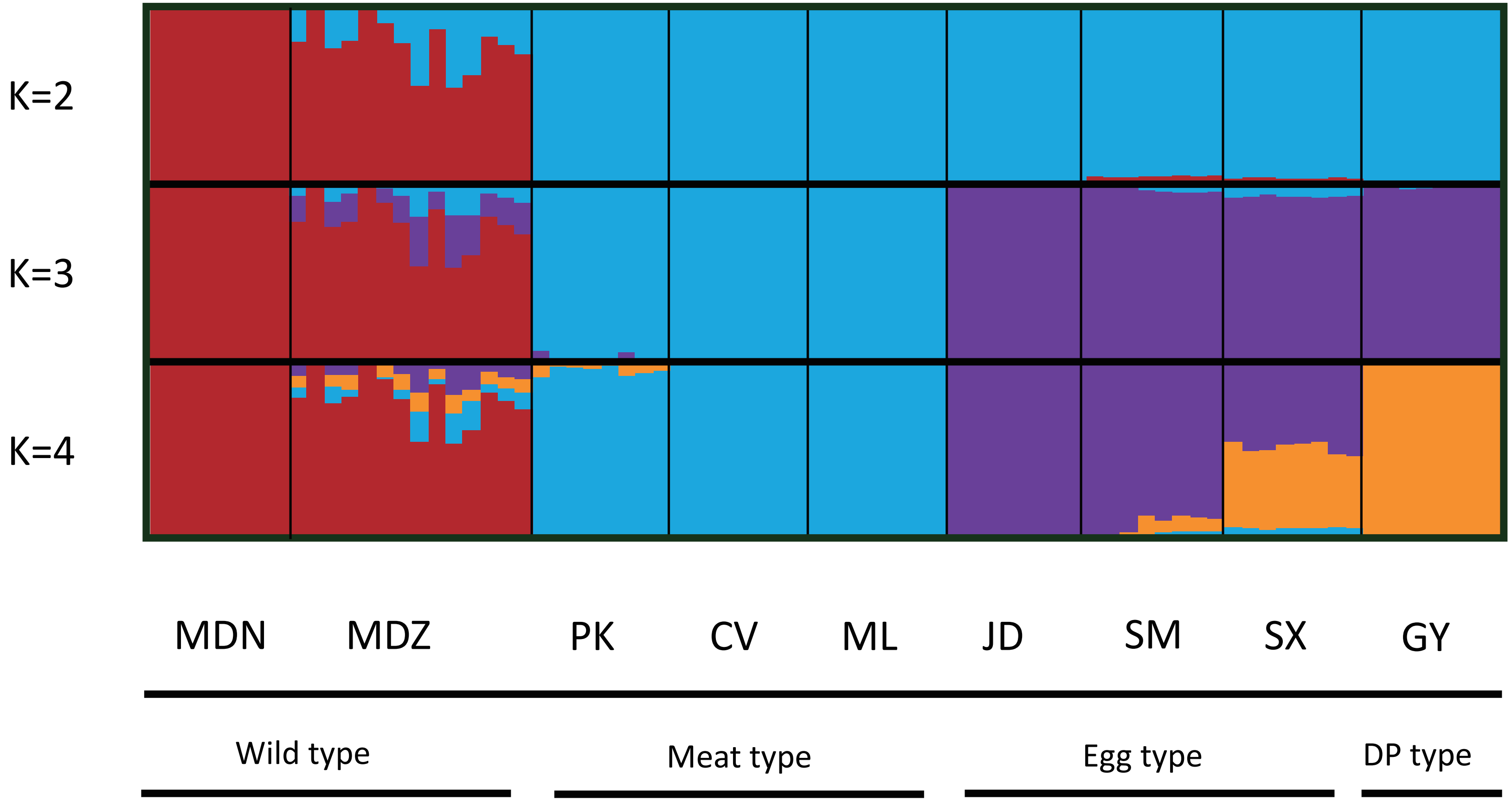

supplemental Figure S6. Population genetic structure of 78 ducks. The length of each colored segment represents the proportion of the individual genome inferred from ancestral populations (K = 2-4). The population names and production type are at the bottom. DP type means dual-purpose type. With K = 2, a clear division was found between wild type ducks (MDN and MDZ) and domesticated ducks (PK, CV, ML, JD, SM, SX, and GY). With K = 3, a clear division was found between meat type ducks (PK, CV, and ML) and egg type ducks mixed with dual-purpose type ducks (JD, SM, SX, and GY). With K=4, a clear division was found between egg type ducks (JD, SM, and SX) and dual-purpose type ducks (GY).
