# Supplementary material for: Whole-genome resequencing reveals signatures of selection and timing of duck domestication
Source: Gigascience. 2018 Apr 9;7(4):giy027. doi: 10.1093/gigascience/giy027 (PMC6007426; doi:10.1093/gigascience/giy027)
Supplement: Response_to_Reviewer_Comments_Original_Submission.pdf [file giy027_response_to_reviewer_comments_original_submission.pdf]

Author's Response To Reviewer Comments

Dear Dr Zauner,

Many thanks for your positive comments about our manuscript, “Whole-genome resequencing reveals signatures of selection and timing of duck domestication” (manuscript number GIGA-D-17-00301). We also thank the reviewers for their thoughtful and constructive suggestions. We have addressed all these comments, detailed below, in our revised manuscript, which we hope is now suitable for publication in GigaScience.

Sincerely,
Lujiang Qu, Ph.D., on behalf of all co-authors.
Email: quluj@163.com
Department of Animal Genetics and Breeding, College of Animal Science and Technology, China Agricultural University, Beijing 100193, China

GIGA-D-17-00301
Whole-genome resequencing reveals signatures of selection and timing of duck domestication
Zebin Zhang; Yaxiong Jia; Pedro Almeida; Judith E Mank; Marcel van Tuinen; Qiong Wang; Zhihua Jiang; Yu Chen; Kai Zhan; Shuisheng Hou; Zhengkui Zhou; Huifang Li; Fangxi Yang; Yong He; Lujiang Qu, Ph.D.
GigaScience

Dear Prof. Qu,

Your manuscript "Whole-genome resequencing reveals signatures of selection and timing of duck domestication" (GIGA-D-17-00301) has been assessed by our reviewers. Although it is of interest, we are unable to consider it for publication in its current form. The reviewers have raised a number of points which we believe would improve the manuscript and may allow a revised version to be published in GigaScience.

Their reports are below.

Comment: All reviewers, but reviewer 2 in particular, provide some suggestions how the submission can be improved, for example by explaining the hypotheses more clearly in the introduction, and also by some additional analyses that may make the paper even stronger.

Reply: Many thanks for your comments. We have more clearly articulated our hypotheses in introduction section according to your and reviewer2’s suggestion, please see lines 75-79. Meanwhile, we have done the additional analyses according to your and reviewer2’s suggestion, such as FRAPPE analyses by K=4, PSMC and δaδi analyses based on chicken mutation rate, global FST between each duck population, and FST recalculated by BayeScan, please see the specific reply to reviewer2.

Comment: An absolutely crucial point for publication in GigaScience is the remark #6 by reviewer 1, regarding sharing of data, code and protocols. GigaScience embraces the FAIR

principles (https://www.force11.org/group/fairgroup/fairprinciples) and we ask our authors to document their work according to these principles, to allow full reproducibility and maximum reuse potential of the data, protocols and scripts.

Please include supporting data such as custom scripts, full population genetic statistics and location of sweeps, any software output files, alignments, phylogenetic tree files etc.

Reply: Thank you for this suggestion. The 78 ducks used in our whole genome resequencing analysis and the 14 ducks used in RNA-seq analysis have been submitted to NCBI BioProject (http://www.ncbi.nlm.nih.gov/bioproject) under accession numbers PRJNA419832 and PRJNA419583, respectively. The unassembled sequencing reads of 78 ducks and RNA-seq reads of 14 ducks have been deposited in NCBI Sequence Read Archive (SRA: http://www.ncbi.nlm.nih.gov/sra) under accession numbers SRP125660 and SRP125529, respectively.

VCF files of SNPs and INDELs, as well as other supporting data, have been submitted to GigaDB as suggested. Please check the GigaDB servers.

Meanwhile, we also replied to reviewer 1 and have added these description to our current manuscript, please see lines 618-628.

To share your supporting data and scripts, our data curators will be able to help you to make them available via our data repository GigaDB. You can contact them via email: database@gigasciencejournal.com.

We are encouraging our submitters to make use of protocols.io , if you provide your methods (both wet-lab and dry-lab) in the SOP tab on the data spreadsheet we can import those into protocols.io on your behalf.

To share your raw sequencing data, please note that the BIG data repository is not part of the International Nucleotide Sequence Database Collaboration. Please choose a database that is an INSDC member (http://www.insdc.org/) and report accession numbers of the INSDC database in the manuscript.

If you are able to fully address points of our reviewers, we would encourage you to submit a revised manuscript to GigaScience. Once you have made the necessary corrections, please submit online at:

http://giga.edmgr.com/

If you have forgotten your username or password please use the "Send Login Details" link to get your login information. For security reasons, your password will be reset.

Please include a point-by-point within the 'Response to Reviewers' box in the submission system. Please ensure you describe additional experiments that were carried out and include a detailed rebuttal of any criticisms or requested revisions that you disagreed with. Please also ensure that

your revised manuscript conforms to the journal style, which can be found in the Instructions for Authors on the journal homepage.

The due date for submitting the revised version of your article is 20 Mar 2018.

I look forward to receiving your revised manuscript soon.

Best wishes,

Hans Zauner
GigaScience
www.gigasciencejournal.com

Reviewer reports:

Reviewer #1:
This paper reports sequencing, population history inferences, and selective sweep mapping in ducks using whole genome sequence data of multiple populations.

This is a good paper. It presents a large-scale population genomic dataset of ducks, uses standard methods that seem appropriate to the task, and it is well written.

Despite this, I have a few criticisms and questions:

Comment: 1. The paper repeatedly states that this is the first time MITF is associated with colour in the duck. This seems not to be entirely true (see Li et al 2012, http://journals.plos.org/plosone/article?id=10.1371/journal.pone.0036592, and Sultana et al 2017, https://www.ncbi.nlm.nih.gov/pubmed/28823136, but maybe the latter was not published when the manuscript was written). This study presents a whole-genome scan, which should provide stronger evidence than candidate gene associations. Comparing to other papers would be interesting. Can that help filter the candidate variants?

Reply: Thank you very much for your positive comments and for the two very helpful citations. Li et al (2012) identified that M isoform of MITF as expressed in black feather ducks, rather than white feather ducks or other colorful ducks. Sultana et al (2017) showed several SNPs and INDEL of MITF with different allele frequency in black and white ducks (table 2 - 5), but did not distinguish the correlation of MITF to white or other feather colors.
Due linkage effects, it is notoriously difficult to determine which variant is the real causative mutation of white plumage. Thus, we used the strictest variant filter criteria, namely those with fixed genotype differences in white and non-white ducks. We would very much like to implement the reviewer's suggestion of using the variants identified in these two previous studies, however the variants reported in Li et al (2012) and Sultana et al (2017) do not in fact pass our strict filter criteria.

We have however added these citations to our manuscript and revised the discussion accordingly (please see line 390). Most importantly, in order to distinguish our result from these previous

studies, we revised our statement to say that “Our results show that white plumage in the duck is completely associated with selection at the MITF locus” in our current manuscript, please see line 42 and line 246-247.

Comment: 2. It would be useful to see the population history results put more into context. In the light of what is known about duck breed history, is it reasonable that meat and egg type ducks split 2100 years ago? In the Discussion, this number is said to be "compatible with previous written records from 500 BC". The reference is to a book with no page numbers given. Would it be possible to be more specific? Given convergence problems with alternative models, how sure are you that the balance between migration and split time is right? I will admit that I am not really the person to evaluate the pairwise sequential Markov coalescent and δaδi results.

Reply: Many thanks for your comments. As we state in the manuscript, written records note domestic ducks in China as early as 500 BC. Due to the lack of archaeological evidence, we must focus on textual evidence, which indicates duck domestication occurred approximately 2,000 - 2,500 years ago. We have added these historical references regarding duck domestication to our current manuscript, please see lines 63-71, and have added page numbers to the book citations, and below, please see lines 697-700. Meanwhile, we also reran the PSMC and δaδi analyses based on the mutation rate estimate in chicken (1.91 x 10-9 per base per generation, Nam et al. 2010). The chicken is phylogenetically closer to the duck than zebra finch, the source of our previous mutation rate estimate (Jarvis et al. 2014), however the mutation rate estimates in both chicken and duck are qualitatively similar. As a result, our results are similar, and indicate duck domestication occurred 2228 441) years ago. We revised the PSMC and δaδi results of our current±(manuscript, please see Fig 2D, Table 1, and lines 204-219, 546-548.

It is true that the recent divergent time and the high level of diversity in both the domestic and wild populations makes it difficult to differentiate recent admixture from incomplete lineage sorting, however our genetic analysis is largely consistent with these written records, and does not indicate domestication much earlier than this time.

Luff R. 2000. Ducks. In Cambridge World History of Food, ed. KF Kiple, KC Ornelas, pp. 517–24. Cambridge, UK: Cambridge University Press
Jarvis, E. D., et al. (2014). "Whole-genome analyses resolve early branches in the tree of life of modern birds." Science 346(6215): 1320-1331.
Nam, K., et al. (2010). "Molecular evolution of genes in avian genomes." Genome Biol 11(6): R68.

Comment: 3. It is nice to see the high overlap between SNPs detected here and those in dbSNP. How many of the indels were already in databases? Was PCR validation only for SNPs? Given that indel detection is harder than SNP detection, are you convinced that the MITF indels are real?

Reply: Thank you for your comments. Initially, we validated our INDELs in dbINDEL, following a similar protocol to our SNP validation. However, there has been less focus on INDEL annotation in the database, which contains nearly 70 fold fewer INDELs than we detected. As we used extremely strict filter criteria for INDELs as well as SNPs, we suggest that

the difference in variation is due to our greater focus on INDEL annotation please lines 497 – 500.
For the two MITF INDELs discussed, we used diagnostic PCR combined with Sanger sequencing to validate these sites in the 78 white and non-white ducks, as well as the first three SNPs (SNP817793, SNP817818, and SNP818004). The Sanger sequencing results of the three SNPs and INDEL817958 completely match our NGS analysis, please see figure below and supplemental figure S5 in our current manuscript. For INDEL818495, we were unable to identify a suitable PCR primer. We have added this to our revised manuscript, please see lines 247-253.

Comment: 4. A protocol for PCR validation seems to be missing (L440-442). It is hard to interpret the 100% accuracy in SNP validation when it is not clear how validation was performed or the accuracy evaluated.

Reply: Apologies, and many thanks for pointing this out. The SNP validation was performed by diagnostic PCR combined with Sanger sequencing method. We have added this description to our revised manuscript, please see lines 510-513.

Comment: 5. The paper is well written, but the GigaScience author guidelines prescribe a somewhat different structure. It specifies an abstract divided into Background, Results, and Conclusions. The Data Description section is missing and other sections are have different names.

Reply: Thank you very much for this helpful suggestion. We had separated the abstract section accordingly, please see lines 30-44. We have also added the Data Description section, please see lines 86-109. We also renamed the Results as Analyses, please see line 111, and revised the Availability of Supporting Data and Materials (lines 618-628), and the Declarations section (lines 632, 633, and 641).

Comment:6. It seems to me that the data and source code availability may not be in line with the journal policies. I am not certain how to interpret the policies, but the editors will know better. Overall, the methods are described in text, but protocols and scripts are not provided. The raw sequence data is published in a repository, but little else, not even the full population genetic statistics or location of sweeps, as far as I can tell.

Reply: Apologies for our previous raw data and source code status. The data from the 78 ducks used in whole genome resequencing and the 14 ducks used in RNA-seq analysis have been submitted to NCBI BioProject (http://www.ncbi.nlm.nih.gov/bioproject) under accession numbers PRJNA419832 and PRJNA419583, respectively. The unassessembled sequencing reads of 78 ducks and RNA-seq reads of 14 ducks have been deposited in the NCBI Sequence Read Archive (SRA: http://www.ncbi.nlm.nih.gov/sra) under accession numbers SRP125660 and SRP125529, respectively. VCF files of SNPs and INDELs, as well as other supporting data, have been submitted to GigaDB as you suggest, please check the GigaDB servers. And, we add these description to our current manuscript, please see lines 618-628.

Minor comments

Comment: Line 35: The important numbers are the number of individuals sampled and the coverage per individual. Average coverage per breed seems less interesting.

Reply: Many thanks for your comment, we had revised this to per individual coverage information, please see line 36.

Comment: Lines 97-101: What do the average numbers of variants detected per individual mean? Are they variants that differ from reference genome, heterozygous variants, or something else?

Reply: Many thanks for your questions. The number of variants between the reference genome and each individual are different, especially in wild mallard and domesticated ducks, (please see supplementary table S2). The average value is the mean variant count of an individual, which includes both heterozygous variants and homozygous variants.

Comment: Lines 243-250: Which GO terms were these, and how were they chosen? It seems odd to me to first select a subset of genes based on GO and then perform enrichment analysis on that set. Will this not bias the analysis?

Reply: Apologies for any confusion. In fact, we observed 292 genes in the top 5% Fst regions, please see supplementary table S5. Our enrichment analysis is based on these 292 genes, and we identified a subset of GO terms for further analyses based on significant GO term P-values, please see supplementary table S7. Moreover, we add the full GO terms to our current manuscript, please see supplementary table S6.

Comment: Lines 393-400: Is there a reason for this mix of sequencing coverage?

Reply: We aimed to sequence each individual at 5X coverage. Additionally, in order to reduce the false negative rate of variants due to our strict filter criteria, we randomly selected one individual from each population for 10X coverage.

Comment: Lines 381-384: It is not clear where the ducks came from. How were they obtained?

Reply: Many thanks for your questions. PK and ML ducks were obtained from Institute of Pekin Duck with the help of Mr. Fangxi Yang, please see author information section, lines 5 and 25. CV ducks were obtained from Cherry Valley farms Co. Ltd with the help of Dr. Yong He, please see lines 5 and 26. The other domesticated ducks were obtained from different duck breeding farms under the help of Dr. Huifang Li, please see lines 5 and 23.

Comment: Line 506: What tool was used for Fst? Also VCFtools?

Reply: Thanks you very much for your questions. The Fst was calculated by the formula described by Weir BS (1984) under our custom perl script. Our custom perl script have been submitted to GigaDB database.

Weir, B. S. and C. C. Cockerham (1984). "Estimating F-Statistics for the Analysis of Population-Structure." Evolution 38(6): 1358-1370.

Comment: Figure 1b: The circos plot in Figure 1 looks impressive, but is impossible to read. What is it supposed to show?

Reply: Apologies for any problems with our figures. The complicated circos plot is the result of the many scaffolds (78,488) in the current duck reference genome. We have removed the circos plot from our current manuscript, please see figure 1, and line 125-127 .

Comment: Throughout methods: Version numbers are missing for some softwares.

Reply: Apologies for this. We have added all this information to our current manuscript, such as NGS QC Toolkit v2.3.3 (line 480), SnpEff v4.0 (line 501), GCTA v1.25 (line 520), MUSCLE v3.8 (line 532), PSMC v0.6.5 (line 541), ∂a∂i v1.7 (line 550), VCFtools v0.1.13 (line 592), and edgeR v3.6 (line 617).

Reviewer #2:

Zhang et al. sequenced whole genomes of 78 individuals of domesticated and wild mallard populations. The authors find a complex history of domestication, with particular artificial selection of meat and egg production in domesticated lineages. Further, outlier analyses demonstrate that white plumage was the result of selection of MITF transcriptional factors. I believe that the authors are tackling an important question regarding variation between domesticates and wild populations, and with an extensive genomic dataset. However, I think the authors fall short in introducing the subject and discussing their results. Moreover, the manuscript requires editing prior to publication, particularly the introduction.

Comment: Introduction.
The introduction requires extensive editing. I would also encourage the authors to add another sentence as the relevance (the why) of looking for outliers between domesticated and wild stocks. What exactly are you trying to learn? Instead of results, I would like to see hypotheses regarding what the authors may expect when comparing the genomes of domesticated and wild populations.

Reply: Many thanks for your comments. The most important reason we identified outliers between wild and domesticated ducks was to identify putative sites associates with the genetic basis of phenotypic differences between wild and domestic populations. We have added this explanation to our manuscript, and have also extensively revised our introduction section according to your suggestions, please see lines 51-85.

We had two primary hypotheses regarding duck domestication given the deep divergence between meat and egg breeds. Were ducks domesticated once from wild mallards and subsequently selected for separate egg and meat traits, or were egg and meat populations domesticated in two independent events. We have add the hypotheses of duck domestication scenarios to introduction section, please see lines 75-79.

Comment: The whole first paragraph requires editing.
For example -- Line 50-52: Suggest change sentence to: "Mallards (Anas platyrhynchos) are the world's most widely distributed and agriculturally important waterfowl species, and are especially of economic importance in Asia [1]."

Reply: Many thanks for this suggestion. We had revised the sentence accordingly, please see lines 63-64. And we have also extensively revised the first paragraph as suggested, please see lines 52-71.

Comment: Results
1. Line 79 - is this 535 billion mappable reads per sample or across samples?

Reply: Apologies for any confusion. The 535 billion is the total mapped reads across samples. We have added this explanation to our revised manuscript, please see line 117.

Comment: 2. Lines 115-121- how did the authors pick the optimum K in FRAPPE analyses? Did the authors explore additional K values? Where separate analyses done within wild and domesticated populations? Please explain.

Reply: Many thanks for your comments. We analyzed the population structure with K =2, 3 and 4 because there are four duck types across the nine duck populations, shown below, and explained in lines161-165. When K=4, a clear division was found between egg type ducks (JD, SM, and SX) and dual-purpose type ducks (GY) (supplemental figure S6). The most important reason we focused on K=3 as the optimum value for further analysis is due to the results of both the phylogenic and PCA analyses, which convergently showed the nine duck populations clustered into 3 major groups.

Comment: 2a. What do the authors make of domesticated admixture in wild populations? Is this hybridization, ancestry, a combination of both…? I would encourage the authors to explore this further as hybridization between domesticated and wild breeds is a serious concern for conservation of wild populations.

Reply: We agree with the reviewer that this is a very interesting area, and an area of great conservation importance. Unfortunately, given the recent domestication and high levels of diversity we observe, it is not in fact possible to accurately differentiate hybridization from incomplete lineage sorting with our current data, as complex models with these alternative scenarios failed to converge. We agree that this is an interesting area for further study, and have added this explanation to our current manuscript, please see lines 377-381.

Comment: 2b. The PCA analyses seem to suggest that there is structure within wild populations. Running a FRAPPE analyses on wild populations could help tease out whether they are 1 population and PCA analyses are just separating samples as there is so much variation.

Reply: Thank you very much for your comments. Of course, the PCA result showed there is a structure within wild populations, because the two wild populations come from two different provinces in China separated by nearly 2,000 km, (please see line 446). However, the PCA result also showed extensive overlap of these two wild populations, please see fig 2B. Additionally, our FRAPPE analyses were based on all 78 duck individuals rather than pooled population information. Thus, we apologize if we have missed something intended by the reviewer, but we think the structural analysis suggested with recover the same result as our current analysis.

Comment: 3. Lines 139-141 - consider revising the sentence into a more formal hypothesis. I would also like to see such hypotheses in the introduction.

Reply: Thank you so much for your kind suggestion. We had two primary hypotheses regarding duck domestication given the deep divergence between meat and egg breeds. Were ducks domesticated once from wild mallards and subsequently selected for separate egg and meat traits, or were egg and meat populations domesticated in two independent events. We have added the hypotheses of duck domestication scenarios to introduction section, please see lines 75-79.

Comment: 4. Outside of outlier tests by calculating FST, the authors should consider more formal testing of these putative outliers (e.g., BayeScan).

Reply: Thank you very much for this suggestion. We have recalculated our FST with BayeScan, and the results are statistically similar to our current analysis, based on Weir, B. S. (1984). Thus, we have kept our previous FST method in our revised manuscript, as this method is a classical and formal method for calculating FST, and has been widely implemented in many organisms, including rice (Meyer, R. S., et al. 2016), sheep (Yang, J., et al. 2016), dog (Gou, X., et al. 2014, Axelsson, E., et al. 2013), and pigeon (Shapiro, M. D., et al. 2013).

Weir, B. S. and C. C. Cockerham (1984). "Estimating F-Statistics for the Analysis of Population-Structure." Evolution 38(6): 1358-1370.
Meyer, R. S., et al. (2016). "Domestication history and geographical adaptation inferred from a SNP map of African rice." Nat Genet 48(9): 1083-1088.
Yang, J., et al. (2016). "Whole-Genome Sequencing of Native Sheep Provides Insights into Rapid Adaptations to Extreme Environments." Mol Biol Evol 33(10): 2576-2592.
Gou, X., et al. (2014). "Whole-genome sequencing of six dog breeds from continuous altitudes reveals adaptation to high-altitude hypoxia." Genome Res 24(8): 1308-1315.
Axelsson, E., et al. (2013). "The genomic signature of dog domestication reveals adaptation to a starch-rich diet." Nature 495(7441): 360-364.
Shapiro, M. D., et al. (2013). "Genomic diversity and evolution of the head crest in the rock pigeon." Science 339(6123): 1063-1067.

Comment: 5. Although I like the idea of RNA-seq data here. I think that this is largely overlooked in the manuscript and may detract from the main (genome) focus. I would encourage the authors to consider taking the RNA-seq out or sufficiently expanding on methods, reasoning, etc. of the RNA-seq data.

Reply: Thank you so much for your suggestion. We respectfully suggest that the RNA-seq is a

key component of our manuscript, as it represents functional phenotypic differentiation of wild mallards and domesticated ducks, and helps connect the genomic variation to phenotypic differences. We have revised the methods and reasoning of including this data RNA-seq as suggested, please see lines 324-328, 470-475, and 603-615.

Comment: 6. I would like to see global Fst estimates among breeds, wild locations

Reply: Many thanks for your comment. The global FST between were showed in below, and we also add this table to our current manuscript, please see lines 267-268, and supplemental table S4.

Comment: Discussion
I have no issues with the discussion and find it the best written. I think that a section on domesticate and wild hybridization may broaden the appeal of this paper.

Reply: Thanks for this suggestion. As we mentioned above, given the recent domestication and high levels of diversity we observe, it is not possible to accurately differentiate hybridization from incomplete lineage sorting with our current data, as complex models with these alternative scenarios failed to converge. We agree that this is an interesting area for further study, and have added material to the discussion as suggested, please see lines 377-381.

Comment: Methods
Please add additional information regarding FRAPPE analyses, K selection,etc.

Reply: Apologies for any omissions. We have added the method of FRAPPE analyses and K selection to our current manuscript, please see lines 523-529.

Comment: Figures
Figure 1: Consider re-moving statistical tests as these are presented in the results.

Reply: Thanks for your helpful comment. We have moved the statistical tests to the results section as suggested, please see lines 129-133, 144-147.

Reviewer #3:

Overall a very nice paper, detailed comments to the authors:

Comment: Line 35: 45X coverage is misleading since the individual coverage was much smaller, please make a clearer statement here

Reply: Thank you for this helpful suggestion. We have revised the population coverage information to individual information, please see line 36.

Comment: L40: Our FST analysis also indicates for the first time ...

Reply: Thanks for this suggestion. We have revised our manuscript according to your suggestion, please see lines 41-43.

Comment: L52: of particular economic importance ...

Reply: Many thanks for your comment. Done! Please see line 65.

Comment: L60-72: This is not introduction, but actually another summary, which I think is obsolete, a slightly more extended real introduction discussing backgraound prior knowledge, and aims of the study, would be preferred

Reply: Many thanks. We have moved this section of our previous version to Data Description according to GigaScience author guidelines and your suggestions, please lines 91-109. Meanwhile, we have revised our Introduction section, please see lines 52-85.

Comment: Figure 1B: this panel is nice, but not very informative, what exact information is retrieved from the graph?

Reply: Apologies for any problems with our figures. The complicated circos plot is the result of the many scaffolds (78,488) in the current duck reference genome. We have removed the circos plot from our current manuscript, please see Figure 1.

Comment: L95: The number of deletions was higher than the number of insertions in all nine populations

Reply: Done! Please see line 134.

Comment: L105: Move the sentence "Single base-pair INDELs were the predominant form, accounting for 38.63% of all detected INDELs (Supplemental Table S3)." before the sentence "Both the number of SNPs ..."

Reply: Thank you so much for your kind suggestion. We revised our manuscript accordingly, please see lines 142-143.

Comment: L111: ... clustered together, the three ...

Reply: Done! Please see line 155.

Comment: L117: Show figure for K=2?

Reply: Thanks for your question. Both K=2 and K=3 were showed in fig 2C, please see line 166.

Comment: L155: ... had the lowest Akaike Information Criteria (AIC) value, ...

Reply: Done! Please see lines 200-201.

Comment: L166: ... are lower than in wild mallards ...

Reply: Done! Please see line 213.

Comment: Table 1: is it possible to report standard errors or confidence intervals of the reported estimates?

Reply: Many thanks for your question. To answer the reviewer's question we added 95% confidence intervals to all estimates. We reanalyzed the demographic history of duck domestication based on mutation rates of both zebra finch and chicken. Using the mutation rate of zebra finch (Jarvis et al. 2014), the time of duck domestication is estimated at 2,128 (+- 421) years ago. With estimates of mutation rate from chicken (Nam et al. 2010), we estimate domestication 2,228 (+- 441) years ago. Considering the genetic relationship of duck to chicken is much closer than to zebra finch (Jarvis, E. D., et al. 2014), we revised the PSMC and δaδi results of our current manuscript, please see Fig 2D, Table 1, and lines 203-211, 547-549.

Comment: L197: ... white plumage phenotype suggesting a causative mutation. Our result indicates for the first time the duck white plumage associated with selection at ...

Reply: Done! Please see lines 245-247.

Comment: L213: of 10kb size.

Reply: Done! Please see line 267.

Comment: L224: "... scaffolds longer than 10-kb by 10-kb windows with 5-kb steps." This is not clear to me, please describe better.

Reply: Apologies for any confusion. In our study, both FST and π were calculated for each 10kb size window, with 5kb size steps. However, of the 78,488 scaffolds in the duck reference genome, there are many scaffolds < 10kb. These short scaffolds were removed, and we only calculated FST for scaffolds > 10kb. We have added this to our revised manuscript, please see lines 279-281.

Comment: L237 was shown

Reply: Done! Please see lines 293-294.

Comment: L240 level differs between domesticated and wild duck.

Reply: Done! Please see line 296.

Comment: L245 I understand that you limited the GO analysis to certain processes, what happened if you included other processes as well?

Reply: Many thanks for this suggestion. In this study, all 292 genes located in the 5% FST regions (supplementary table S5) were used for the GO analysis, resulting in a total of 57 GO enrichment terms, which have now all been added to our current manuscript, please see lines 300-301, and supplementary table S6. This high number of GO terms presents a hopelessly difficult and complicated analyses, therefore we selected a subset of GO terms for further analysis based on P-value (supplementary table S7) combined the phenotypic differences between wild mallard and domestic duck. We do agree with the reviewer that a more inclusive analysis would be preferable, but the large number of GO terms makes it impossible to obtain meaningful results.

Comment: L252 identified as being under positive selection

Reply: Corrected! Please see line 311.

Comment: L258 Is "neuronal genes" the right term?

Reply: Apologies for any confusion. “Neuronal genes” is not in fact a GO term, rather a simplification of “25 neuro-synapse-axon genes” in line 310. To be more understandable, we have removed this simplification in our revision, please see line 317.

Comment: L260 fatty acid

Reply: Apologies and corrected! Please see line 319.

Comment: L269 and no gene in breast muscle

Reply: Done! Please see line 329.

Comment: L273 The results suggest that the PDC gene is of substantial functional importance in phenotypic differentiation among wild and domestic ducks.

Reply: Many thanks. We have revised this sentence according to your suggestion, please see lines 333-335.

Comment: L289 catalogued 36.1M SNPs and 3.1M INDELs,

Reply: Corrected! Please see line 349.

Comment: L333 ... showed particularly strong signs of selective sweep s presumably associated with domestication.

Reply: We have corrected our manuscript according to your suggestion, please see lines 398-399.

Comment: L340 brain and liver of domesticated ducks compared to ...

Reply: Corrected! Please see line 405.

Comment: L351 differential selection? Do you mean directional selection?

Reply: Apologies for any confusion. We also revised our current manuscript, please see lines 416-418.

Comment: L362 Taken together, our results show that duck domestication was a relatively recent and ...

Reply: We have corrected our manuscript according to your suggestion, please see line 430.

Comment: L440 From the 28,199,227 SNPs not confirmed by dbSNPs, 390 randomly chosen (?) nucleotide sites

Reply: Many thanks for your question. Of course, all nucleotide sites were randomly selected. We have added this explain to our current manuscript, please see lines 510-513.

Comment: L448 Principal Component Analysis (PCA), first by generating the genetic relationship matrix (GRM) from which the first 20 eigenvectors were extracted.

Reply: We have corrected our manuscript according to your suggestion, please see line 520-522.

--
Please also take a moment to check our website at http://giga.edmgr.com/l.asp?i=25723&l=YHKU51UQ for any additional comments that were saved as attachments. Please note that as GigaScience has a policy of open peer review, you will be able to see the names of the reviewers.
