# Supplementary material for: Whole-genome resequencing reveals signatures of selection and timing of duck domestication
Source: Gigascience. 2018 Apr 9;7(4):giy027. doi: 10.1093/gigascience/giy027 (PMC6007426; doi:10.1093/gigascience/giy027)
Supplement: Response_to_Reviewer_Comments_Revision_1.pdf [file giy027_response_to_reviewer_comments_revision_1.pdf]

Author's Response To Reviewer Comments

Dear Dr Zauner,

Many thanks for your positive comments about our manuscript, “Whole-genome resequencing reveals signatures of selection and timing of duck domestication” (manuscript number GIGA-D-17-00301R1). We also thank the reviewers for their thoughtful and constructive suggestions. We have addressed all these comments, detailed below, in our revised manuscript, which we hope is now suitable for publication in GigaScience.

Sincerely,
Lujiang Qu, Ph.D., on behalf of all co-authors.
Email: quluj@163.com
Department of Animal Genetics and Breeding, College of Animal Science and Technology, China Agricultural University, Beijing 100193, China

GIGA-D-17-00301R1
Whole-genome resequencing reveals signatures of selection and timing of duck domestication
Zebin Zhang; Yaxiong Jia; Pedro Almeida; Judith E Mank; Marcel van Tuinen; Qiong Wang; Zhihua Jiang; Yu Chen; Kai Zhan; Shuisheng Hou; Zhengkui Zhou; Huifang Li; Fangxi Yang; Yong He; Zhonghua Ning; Ning Yang; Lujiang Qu, Ph.D.
GigaScience

Dear Prof. Qu,

Your revised manuscript "Whole-genome resequencing reveals signatures of selection and timing of duck domestication" (GIGA-D-17-00301R1) has been assessed again by our reviewers.

I am happy that the reviewers feel that many of their previous comments have been addressed and the manuscript has improved. However, some issues remain to be clarified, and I urge you to fully address the latest comments in a second revised manuscript.

Please see the reviewers' reports below.

Comment: Please pay particular attention to the comments of reviewer 1 regarding availability of population genetics raw data, coordinates of sweeps, scripts, etc, as well as full step-by-step description of all wet and dry lab protocols. As I explained in my previous decision letter, reproducibility of methods and full data availability are of utmost importance for acceptance in GigaScience.

Reply: Many thanks for your comment. All population genetic raw data and command scripts have been submitted to the GigaDB database according to reviewer 1 and your suggestion. We also add the description of all wet and dry protocols to our current manuscript, please see specific replies below.

As mentioned previously, the protocols.io platform is a very convenient way to share

experimental protocols, and I recommend you to consider this option. Please do let me know if you have questions regarding how we can integrate protocols.io entries with your manuscript.

Our data curators will contact you to prepare the GigaDB set that will be posted alongside your manuscript, if it is accepted.

Please include a citation to your GigaDB dataset (including the DOI link) to your reference list, and cite this in the data availability section and elsewhere in the manuscript, where appropriate.

Please follow this example format for the reference:

[xx] Author1 N, Author2 N, AuthorX N. Supporting data for "Title of your manuscript". GigaScience Database 2018. http://dx.doi.orgxxxxxxxxxxxx

(We will replace the dummy doi (xxxx) with the final version prior to acceptance).

Once you have made the necessary corrections, please submit a revised manuscript online at:

https://giga.editorialmanager.com/

If you have forgotten your username or password please use the "Send Login Details" link to get your login information. For security reasons, your password will be reset.

Please include a point-by-point within the 'Response to Reviewers' box in the submission system. Please also ensure that your revised manuscript conforms to the journal style, which can be found in the Instructions for Authors on the journal homepage.

The due date for submitting the revised version of your article is 15 May 2018.

We look forward to receiving your revised manuscript soon.

Best wishes,

Hans Zauner
GigaScience
www.gigasciencejournal.com

Reviewer reports:
Reviewer #1: In my opinion, this revision adequately answers most of my comments. The manuscript has also improved with the answers to the other reviewer.

I have only a few remaining comments. The most serious one is about data availability and protocols.

Comment: The revision comes with better data availability. VCF files of variants are included,

plus a couple of perl scripts used to process them. However, full population genetic statistics and sweep locations still seem to be missing. Scripts for running the bioinformatic tools are not included. The description of the PCR follow-up of variants has been expanded. However, the description does not include the full protocol, and neither does the description of any of the other laboratory methods. This level of detail is about the standard in the field, but it does not seem to live up to the policies of the journal.

Reply: Many thanks for your positive comments and apologies for any inadequate descriptions. All population genetic raw data and command scripts have been submitted to the GigaDB database.
We used a sliding windows method for FST calculation in our sweep analysis, as this approach is more robust and informative for genome-wide evaluation. This approach means that one window might have several genes, and some very long genes may be present in multiple overlapping windows. Thus, we substituted sweep locations for gene locations, and added this information to our current manuscript, please see supplemental tables S5 and S8.

We have provided a citation for the specific PCR validation methods (Van et al 2008), which has been widely used in previous studies (Wang et al 2016, Yan et al 2014), please see line 536.

Van Tassell, C. P., et al. (2008). "SNP discovery and allele frequency estimation by deep sequencing of reduced representation libraries." Nat Methods 5(3): 247-252.
Wang, M. S., et al. (2016). "Positive selection rather than relaxation of functional constraint drives the evolution of vision during chicken domestication." Cell Res 26(5): 556-573.
Yan, Y., et al. (2014). "Genome-wide characterization of insertion and deletion variation in chicken using next generation sequencing." PLoS One 9(8): e104652.

Comment: A couple of times (the justification for the mix of sequence coverages, and the detail about the origin of the ducks), the reply to reviewers contain useful information that was not incorporated in the manuscript. In my opinion, the Methods should include this information, and in particular as much detail as possible about the origin of the animals.

Reply: Many thanks for your suggestion. We have add the justification of coverage to the Methods section of our current manuscript, please see lines 486-490. We have also detailed the point of origin for our samples, please see lines 468-474.

Minor comments

Comment: The reply to reviewers describe the variant filtering as "extremely strict". In fact, it seems to be mostly the default starting criteria suggested by GATK developers in their "best practices" (with a "QUAL" cutoff and a higher "QD" cutoff). How were these filter settings chosen? Are they actually "extremely strict"?

Reply: Many thanks for your questions. Of course, all variants were filtered with “hard filter” criteria suggested by GATK developers. However, to identify variants associated with white plumage traits, the “extremely strict” criteria were used, where variant allele frequency must be 0 in all white duck individuals and be 1 in all non-white duck individuals. Or, 1 in all white duck

individuals and 0 in all non-white duck individuals. In other words, the variant had to be completely associated with the phenotype to pass our strictest threshold.

Comment: Line 247: What does "completely associated with selection" mean in this context?

Reply: Thanks for your question. “The duck white plumage is completely associated with selection at the MITF locus” means the mutations were completely associated with white plumage phenotype.

Comment: Lines 252-253: In what sense did the PCR primer design fail? Were you unable to amplify the region, amplify specifically, or unable to find primers that lived up to your quality criteria? I fully understand that PCR primer design fails occasionally, but I think a more specific description would be useful.

Reply: We were unable to design suitable primers to amplify this region, and we add this explanation to our current manuscript, please see line 270.

Reviewer #2: The revised version of the manuscript entitled, "Whole-genome resequencing reveals signatures of selection and timing of duck domestication" tackles the genomic question of domestication. The authors have done much to improve the manuscript. While most of my comments are now minor, there are a few additional requests that would be nice to see incorporated in order to strengthen the manuscript. I believe that the paper will be ready for submission if the authors incorporate all/most comments (See below).

Comment: INTRODUCTION/DATA DESCRIPTION: I think the introduction is much improved. In addition to minor comments below, I would still like to see the authors develop at least one hypothesis as to what genes/genetic regions may be playing a role in the meat/egg domestication process of these ducks. Alternatively (or in addition to), I would like to see a hypothesis regarding what they think some of the differences may be between wild and domesticated populations.

Reply: Thank you very much for your positive comments. Respectfully, the advantage of comparative genomic studies such as ours is that they are agnostic screens of the entire genome without a priori need to develop specific hypotheses. Previous similar studies of domestication (including Rubin et al. Nature 2010; Vonholdt et al. Nature 2010; Montague et al. PNAS 2014, among many others) have used these approaches to identify regions of the genome affected by artificial selection without a priori hypotheses. We adapted these approaches to the study of ducks here, with the broad aim of identifying whether ducks were domesticated once (null hypothesis) or separately for egg and meat breeds (alternative hypothesis). Moreover, we assess the role of domestication on genes related to plumage and neuroanatomy. We respectfully suggest that to develop further post hoc hypotheses to fit our results at this point would be disingenuous, and defeat the purpose of these sorts of agnostic screens.

Rubin, C. J., et al. (2010). "Whole-genome resequencing reveals loci under selection during chicken domestication." Nature 464(7288): 587-591.
Vonholdt, B. M., et al. (2010). "Genome-wide SNP and haplotype analyses reveal a rich history

underlying dog domestication." Nature 464(7290): 898-902.
Montague, M. J., et al. (2014). "Comparative analysis of the domestic cat genome reveals genetic signatures underlying feline biology and domestication." Proceedings of the National Academy of Sciences 111(48): 17230-17235.

Comment: Line 63: remove scientific name as you already introduced mallards in the previous paragraph.

Reply: Done! Please see line 72.

Comment: Line 92: insert "of" - "….613.37 [of] Gb high….". I would also advise the authors to move any kind of findings of this type to RESULTS.

Reply: Done! Please see lines 89-91, 111-112.

Comment: Lines 94: Delete "we detected"

Reply: Done! Please see line 92.

Comment: Line 94: consider change " …,we tested for population structure between domesticated and wild populations, as well as assessed for signatures of selection associated with domestication."

Reply: Many thanks for your helpful suggestion. We have revised our manuscript accordingly, please see lines 92-96.

Comment: Line 96-98: Either delete the sentence starting with "We inferred…" or add another 1-2 sentence explaining what exactly you tested.

Reply: Deleted! Please see line 95-97.

Comment: Lines 104-109: This seems forced and out of place. Either delete it and put it to the discussion OR expand/edit it to be more streamlined.

Reply: This paragraph have been moved to discussion section, please see lines 100-105, and 449-454.

ANALYSIS:

Comment: Line 117: end with "…78 ducks."

Reply: Done! Please see line 113.

Comment: 2nd Paragraph: "Across samples, a total of 36.1 million (M) SNPs (average per sample = 4.5 M SNPs; range = 2.34 - 9.52 M SNPs) and 3.1M INDELs (average per sample = 0.4M INDELs; range = 0.21 - 0.89M INDELs) were detected (Fig. 1C1B, Supplemental Figs.

S1-S2, Supplemental Table S2). ingle base-pair INDELs were the predominant form, and accounting for 38.63% of all detected INDELs (Supplemental Table S3). Our dataset covers 96.2% of the duck dbSNP database deposited in the Genome Variation Map (GVM) (http://bigd.big.ac.cn/gvm/)." In general, domesticated stock showed lower number of SNPs (t test, $p = 3.13 \times 10{-}12$) and nucleotide diversity (ttest, $p = 2.20 \times 10{-}16$) as compared to wild mallards (Fig. 1B - C). Moreover, homozygousity in domesticated ducks was significantly higher than ratios in wild mallards (t test, $p = 1.35 \times 10{-}10$) consistent with the larger panmictic wild population.

Reply: Thank you so much for your helpful suggestion. This paragraph was revised accordingly, please see lines 126-151.

Comment: Line 137: does 36.1 million SNPs include indels? If not, I would just include the 2 in one summation of total diversity.

Reply: Many thanks for your question and helpful suggestion, the 36.1 million SNPs did not include INDELs. These two variation types are summed together according to your suggestion in our current manuscript, please see line 127.

Comment: Line 142 - 143: The sentence "Single base-pair INDELs were the predominant form, accounting for 38.63% of all detected INDELs (Supplemental Table S3)."

Reply: Revised! Please see lines 131-132.

Comment: Line 148: Are you sure that your data is "consistent with larger panmictic wild population" ? What about artificial selection and inbreeding within domesticated stock? Maybe both? Consider revising.

Reply: Apologies for any confusion. We had revised our manuscript accordingly, please see lines 138-140.

Comment: Lines 155 - 158: Consider changing the sentence to: "In general, clustering among samples corresponded with their source, that included wild ducks (MDN and MDZ), ducks domesticated for meat production (PK, CV, and ML), and ducks domesticated for egg production (JD, 157 SM, and SX). The dual-purpose domesticate clustered with ducks domesticated for egg production (Fig. 2B-C)."

Reply: Done! Please see lines 156-160.

Comment: Lines 184-202: Consider revising to 1 paragraph: "Next, we explored the demographic history of our samples to differentiate whether domestication of meat and egg producing ducks was the result of one or multiple events. First, we estimated changes in effective population size (Ne) in our three genetic clusters in a pairwise sequentially Markovian coalescent (PSMC) framework [22]. The meat type ducks (PK, CV, and ML) showed concordant demographic trajectories with egg and mixture dual-purpose type populations (JD, SM, SX, and GY) with one apparent expansion around the Penultimate Glaciation Period (PGP, 0.30-0.13

Mya) [4, 23] and Last Glacial Period (LGP, 110-12 kya) [24, 25], followed by a subsequent contraction (Fig. 2D). Next, we tested multiple demographic scenarios …."

Reply: Done! Please see lines 187-208.

Comment: Line 214: What is the Ne for the wild population. Please make clear by at least referencing Table 1.

Reply: Thank you for this helpful suggestion. We have had add the Ne estimate of the wild population to our main text, please see line 225.

Comment: Lines 224-229: Please cite sources for some of your statements here. Better to make the statement of your findings and save lines 226-229 for discussion.

Reply: Many thanks for your comments. We have moved lines 226-229 to the discussion section according as suggested, please see lines 387-390.

Comment: Line 241: I would like to know if any other region showed deviation/outliers? Or was there only 1 region across the entire genome? Please clarify.

Reply: Many thanks for your questions. This region is the fourth ranked region across the entire genome, but the only one region correlated with coloration. We also revised our current manuscript, please see lines 251-261.

Comment: DISCUSSION: Overall, the discussion is well written, organized, and I find the topics of broad appeal.

I believe the introduction of the Discussion can be combined into a single paragraph and a bit streamlined as it is just reiterating the results.

Reply: Thank you so much for your positive comment and your helpful suggestion. The introduction of the Discussion have been revised and redundant material deleted as you suggest, please see lines 348-363.

Comment: Lines 348 - 353: Consider splitting into at least 2 sentences.

Reply: Done! Please see lines 357-363.

Comment: Line 419: add "and": "dogs [45], and…"

Reply: Done! Please see line 433.
