# Supplementary material for: Whole-genome resequencing reveals signatures of selection and timing of duck domestication
Source: Gigascience. 2018 Apr 9;7(4):giy027. doi: 10.1093/gigascience/giy027 (PMC6007426; doi:10.1093/gigascience/giy027)
Supplement: Reviewer_1_Report_(Original_Submission) -- Martin Johnsson — 07 Dec 2017 Reviewed [file giy027_reviewer_1_report_(original_submission).pdf]

Reviewer Report

Title: Whole-genome resequencing reveals signatures of selection and timing of duck domestication

Version: Original Submission **Date:** 07 Dec 2017

Reviewer name: Martin Johnsson

Reviewer Comments to Author:

This paper reports sequencing, population history inferences, and selective sweep mapping in ducks using whole genome sequence data of multiple populations.

This is a good paper. It presents a large-scale population genomic dataset of ducks, uses standard methods that seem appropriate to the task, and it is well written.

Despite this, I have a few criticisms and questions:

1. The paper repeatedly states that this is the first time MITF is associated with colour in the duck. This seems not to be entirely true (see Li et al 2012, http://journals.plos.org/plosone/article?id=10.1371/journal.pone.0036592, and Sultana et al 2017, https://www.ncbi.nlm.nih.gov/pubmed/28823136, but maybe the latter was not published when the manuscript was written). This study presents a whole-genome scan, which should provide stronger evidence than candidate gene associations. Comparing to other papers would be interesting. Can that help filter the candidate variants?

2. It would be useful to see the population history results put more into context. In the light of what is known about duck breed history, is it reasonable that meat and egg type ducks split 2100 years ago? In the Discussion, this number is said to be "compatible with previous written records from 500 BC". The reference is to a book with no page numbers given. Would it be possible to be more specific? Given convergence problems with alternative models, how sure are you that the balance between migration and split time is right? I will admit that I am not really the person to evaluate the pairwise sequential Markov coalescent and δaδi results.

3. It is nice to see the high overlap between SNPs detected here and those in dbSNP. How many of the indels were already in databases? Was PCR validation only for SNPs? Given that indel detection is harder than SNP detection, are you convinced that the MITF indels are real?

4. A protocol for PCR validation seems to be missing (L440-442). It is hard to interpret the 100% accuracy in SNP validation when it is not clear how validation was performed or the accuracy evaluated.

5. The paper is well written, but the GigaScience author guidelines prescribe a somewhat different structure. It specifies an abstract divided into Background, Results, and Conclusions. The Data

Description section is missing and other sections are have different names.

6. It seems to me that the data and source code availability may not be in line with the journal policies. I am not certain how to interpret the policies, but the editors will know better. Overall, the methods are described in text, but protocols and scripts are not provided. The raw sequence data is published in a repository, but little else, not even the full population genetic statistics or location of sweeps, as far as I can tell.

Minor comments

Line 35: The important numbers are the number of individuals sampled and the coverage per individual. Average coverage per breed seems less interesting.

Lines 97-101: What do the average numbers of variants detected per individual mean? Are they variants that differ from reference genome, heterozygous variants, or something else?

Lines 243-250: Which GO terms were these, and how were they chosen? It seems odd to me to first select a subset of genes based on GO and then perform enrichment analysis on that set. Will this not bias the analysis?

Lines 393-400: Is there a reason for this mix of sequencing coverage?

Lines 381-384: It is not clear where the ducks came from. How were they obtained?

Line 506: What tool was used for Fst? Also VCFtools?

Figure 1b: The circos plot in Figure 1 looks impressive, but is impossible to read. What is it supposed to show?

Throughout methods: Version numbers are missing for some softwares.

Methods

Are the methods appropriate to the aims of the study, are they well described, and are necessary controls included? Yes

Conclusions

Are the conclusions adequately supported by the data shown? Yes

Reporting Standards

Does the manuscript adhere to the journal's guidelines on [minimum standards of reporting?]() No

Choose an item.

Statistics

Are you able to assess all statistics in the manuscript, including the appropriateness of statistical tests used? Yes, and I have assessed the statistics in my report.

Quality of Written English

Please indicate the quality of language in the manuscript: Acceptable

Declaration of Competing Interests

Please complete a declaration of competing interests, considering the following questions:

- Have you in the past five years received reimbursements, fees, funding, or salary from an organisation that may in any way gain or lose financially from the publication of this manuscript, either now or in the future?
- Do you hold any stocks or shares in an organisation that may in any way gain or lose financially from the publication of this manuscript, either now or in the future?
- Do you hold or are you currently applying for any patents relating to the content of the manuscript?
- Have you received reimbursements, fees, funding, or salary from an organization that holds or has applied for patents relating to the content of the manuscript?
- Do you have any other financial competing interests?
- Do you have any non-financial competing interests in relation to this paper?

If you can answer no to all of the above, write 'I declare that I have no competing interests' below. If your reply is yes to any, please give details below.

I declare that I have no competing interests.

I agree to the open peer review policy of the journal. I understand that my name will be included on my report to the authors and, if the manuscript is accepted for publication, my named report including any attachments I upload will be posted on the website along with the authors' responses. I agree for my report to be made available under an Open Access Creative Commons CC-BY license (http://creativecommons.org/licenses/by/4.0/). I understand that any comments which I do not wish to be included in my named report can be included as confidential comments to the editors, which will not be published.

I agree to the open peer review policy of the journal

To further support our reviewers, we have joined with Publons, where you can gain additional credit to further highlight your hard work (see: https://publons.com/journal/530/gigascience). On publication of this paper, your review will be automatically added to Publons, you can then choose whether or not to claim your Publons credit. I understand this statement.

Yes
