# Supplementary material for: Whole-genome resequencing reveals signatures of selection and timing of duck domestication
Source: Gigascience. 2018 Apr 9;7(4):giy027. doi: 10.1093/gigascience/giy027 (PMC6007426; doi:10.1093/gigascience/giy027)
Supplement: Reviewer_1_Report_(Revision_1) -- Martin Johnsson — 06 Feb 2018 Reviewed [file giy027_reviewer_1_report_(revision_1).pdf]

Reviewer Report

Title: Whole-genome resequencing reveals signatures of selection and timing of duck domestication

Version: Revision 1 **Date:** 06 Feb 2018

Reviewer name: Martin Johnsson

Reviewer Comments to Author:

In my opinion, this revision adequately answers most of my comments. The manuscript has also improved with the answers to the other reviewer.

I have only a few remaining comments. The most serious one is about data availability and protocols.

The revision comes with better data availability. VCF files of variants are included, plus a couple of perl scripts used to process them. However, full population genetic statistics and sweep locations still seem to be missing. Scripts for running the bioinformatic tools are not included. The description of the PCR follow-up of variants has been expanded. However, the description does not include the full protocol, and neither does the description of any of the other laboratory methods. This level of detail is about the standard in the field, but it does not seem to live up to the policies of the journal.

A couple of times (the justification for the mix of sequence coverages, and the detail about the origin of the ducks), the reply to reviewers contain useful information that was not incorporated in the manuscript. In my opinion, the Methods should include this information, and in particular as much detail as possible about the origin of the animals.

Minor comments

The reply to reviewers describe the variant filtering as "extremely strict". In fact, it seems to be mostly the default starting criteria suggested by GATK developers in their "best practices" (with a "QUAL" cutoff and a higher "QD" cutoff). How were these filter settings chosen? Are they actually "extremely strict"?

Line 247: What does "completely associated with selection" mean in this context?

Lines 252-253: In what sense did the PCR primer design fail? Were you unable to amplify the region, amplify specifically, or unable to find primers that lived up to your quality criteria? I fully understand that PCR primer design fails occasionally, but I think a more specific description would be useful.

Methods

Are the methods appropriate to the aims of the study, are they well described, and are necessary controls included? Yes

Conclusions

Are the conclusions adequately supported by the data shown? Yes

Reporting Standards

Does the manuscript adhere to the journal's guidelines on minimum standards of reporting? No

Statistics

Are you able to assess all statistics in the manuscript, including the appropriateness of statistical tests used? Yes, and I have assessed the statistics in my report.

Quality of Written English

Please indicate the quality of language in the manuscript: Acceptable

Declaration of Competing Interests

Please complete a declaration of competing interests, considering the following questions:

- Have you in the past five years received reimbursements, fees, funding, or salary from an organisation that may in any way gain or lose financially from the publication of this manuscript, either now or in the future?
- Do you hold any stocks or shares in an organisation that may in any way gain or lose financially from the publication of this manuscript, either now or in the future?
- Do you hold or are you currently applying for any patents relating to the content of the manuscript?
- Have you received reimbursements, fees, funding, or salary from an organization that holds or has applied for patents relating to the content of the manuscript?
- Do you have any other financial competing interests?
- Do you have any non-financial competing interests in relation to this paper?

If you can answer no to all of the above, write 'I declare that I have no competing interests' below. If your reply is yes to any, please give details below.

I declare that I have no competing interests.

I agree to the open peer review policy of the journal. I understand that my name will be included on my report to the authors and, if the manuscript is accepted for publication, my named report including any attachments I upload will be posted on the website along with the authors' responses. I agree for my report to be made available under an Open Access Creative Commons CC-BY license (http://creativecommons.org/licenses/by/4.0/). I understand that any comments which I do not wish to be included in my named report can be included as confidential comments to the editors, which will not be published.

I agree to the open peer review policy of the journal

To further support our reviewers, we have joined with Publons, where you can gain additional credit to further highlight your hard work (see: https://publons.com/journal/530/gigascience). On publication of this paper, your review will be automatically added to Publons, you can then choose whether or not to claim your Publons credit. I understand this statement.

Yes
