# Supplementary material for: Whole-genome resequencing reveals signatures of selection and timing of duck domestication
Source: Gigascience. 2018 Apr 9;7(4):giy027. doi: 10.1093/gigascience/giy027 (PMC6007426; doi:10.1093/gigascience/giy027)
Supplement: Reviewer_2_Report_(Original_Submission) -- Philip Lavretsky — 11 Dec 2017 Reviewed [file giy027_reviewer_2_report_(original_submission).pdf]

Reviewer Report

Title: Whole-genome resequencing reveals signatures of selection and timing of duck domestication

Version: Original Submission **Date:** 11 Dec 2017

Reviewer name: Philip Lavretsky

Reviewer Comments to Author:

Zhang et al. sequenced whole genomes of 78 individuals of domesticated and wild mallard populations. The authors find a complex history of domestication, with particular artificial selection of meat and egg production in domesticated lineages. Further, outlier analyses demonstrate that white plumage was the result of selection of MITF transcriptional factors. I believe that the authors are tackling an important question regarding variation between domesticates and wild populations, and with an extensive genomic dataset. However, I think the authors fall short in introducing the subject and discussing their results. Moreover, the manuscript requires editing prior to publication, particularly the introduction.

Introduction

The introduction requires extensive editing. I would also encourage the authors to add another sentence as the relevance (the why) of looking for outliers between domesticated and wild stocks. What exactly are you trying to learn? Instead of results, I would like to see hypotheses regarding what the authors may expect when comparing the genomes of domesticated and wild populations. I think the

The whole first paragraph requires editing.

For example -- Line 50-52: Suggest change sentence to: "Mallards (Anas platyrhynchos) are the world's most widely distributed and agriculturally important waterfowl species, and are especially of economic importance in Asia [1]."

Results

1. Line 79 - is this 535 billion mappable reads per sample or across samples?
2. Lines 115-121- how did the authors pick the optimum K in FRAPPE analyses? Did the authors explore additional K values? Where separate analyses done within wild and domesticated populations? Please explain.

2a. What do the authors make of domesticated admixture in wild populations? Is this hybridization, ancestry, a combination of both...? I would encourage the authors to explore this further as hybridization between domesticated and wild breeds is a serious concern for conservation of wild populations.

2b. The PCA analyses seem to suggest that there is structure within wild populations. Running a FRAPPE analyses on wild populations could help tease out whether they are 1 population and PCA analyses are just separating samples as there is so much variation.

3. Lines 139-141 - consider revising the sentence into a more formal hypothesis. I would also like to see such hypotheses in the introduction.

4. Outside of outlier tests by calculating FST, the authors should consider more formal testing of these putative outliers (e.g., BayeScan).
5. Although I like the idea of RNA-seq data here. I think that this is largely overlooked in the manuscript and may detract from the main (genome) focus. I would encourage the authors to consider taking the RNA-seq out or sufficiently expanding on methods, reasoning, etc. of the RNA-seq data.
6. I would like to see global Fst estimates among breeds, wild locations
Discussion
I have no issues with the discussion and find it the best written. I think that a section on domesticate and wild hybridization may broaden the appeal of this paper.

Methods
Please add additional information regarding FRAPPE analyses, K selection,etc.
Figures
Figure 1: Consider re-moving statistical tests as these are presented in the results.

Methods

Are the methods appropriate to the aims of the study, are they well described, and are necessary controls included? Yes

Conclusions

Are the conclusions adequately supported by the data shown? Yes

Reporting Standards

Does the manuscript adhere to the journal's guidelines on minimum standards of reporting? Yes

Statistics

Are you able to assess all statistics in the manuscript, including the appropriateness of statistical tests used? Yes, and I have assessed the statistics in my report.

Quality of Written English

Please indicate the quality of language in the manuscript: Not suitable for publication unless extensively edited

Declaration of Competing Interests

Please complete a declaration of competing interests, considering the following questions:

- Have you in the past five years received reimbursements, fees, funding, or salary from an organisation that may in any way gain or lose financially from the publication of this manuscript, either now or in the future?

- Do you hold any stocks or shares in an organisation that may in any way gain or lose financially from the publication of this manuscript, either now or in the future?

- Do you hold or are you currently applying for any patents relating to the content of the manuscript?

- Have you received reimbursements, fees, funding, or salary from an organization that holds or has applied for patents relating to the content of the manuscript?

- Do you have any other financial competing interests?

- Do you have any non-financial competing interests in relation to this paper?

If you can answer no to all of the above, write 'I declare that I have no competing interests' below. If your reply is yes to any, please give details below.

I declare that I have no competing interests

I agree to the open peer review policy of the journal. I understand that my name will be included on my report to the authors and, if the manuscript is accepted for publication, my named report including any attachments I upload will be posted on the website along with the authors' responses. I agree for my report to be made available under an Open Access Creative Commons CC-BY license (http://creativecommons.org/licenses/by/4.0/). I understand that any comments which I do not wish to be included in my named report can be included as confidential comments to the editors, which will not be published.

I agree to the open peer review policy of the journal

To further support our reviewers, we have joined with Publons, where you can gain additional credit to further highlight your hard work (see: https://publons.com/journal/530/gigascience). On publication of this paper, your review will be automatically added to Publons, you can then choose whether or not to claim your Publons credit. I understand this statement.

Yes
