# Supplementary material for: Whole-genome resequencing reveals signatures of selection and timing of duck domestication
Source: Gigascience. 2018 Apr 9;7(4):giy027. doi: 10.1093/gigascience/giy027 (PMC6007426; doi:10.1093/gigascience/giy027)
Supplement: Reviewer_2_Report_(Revision_1) -- Philip Lavretsky — 12 Feb 2018 Reviewed [file giy027_reviewer_2_report_(revision_1).pdf]

Reviewer Report

Title: Whole-genome resequencing reveals signatures of selection and timing of duck domestication

Version: Revision 1 **Date:** 12 Feb 2018

Reviewer name: Philip Lavretsky

Reviewer Comments to Author:

The revised version of the manuscript entitled, "Whole-genome resequencing reveals signatures of selection and timing of duck domestication" tackles the genomic question of domestication. The authors have done much to improve the manuscript. While most of my comments are now minor, there are a few additional requests that would be nice to see incorporated in order to strengthen the manuscript. I believe that the paper will be ready for submission if the authors incorporate all/most comments (See below).

INTRODUCTION/DATA DESCRIPTION: I think the introduction is much improved. In addition to minor comments below, I would still like to see the authors develop at least one hypothesis as to what genes/genetic regions may be playing a role in the meat/egg domestication process of these ducks. Alternatively (or in addition to), I would like to see a hypothesis regarding what they think some of the differences may be between wild and domesticated populations.

Line 63: remove scientific name as you already introduced mallards in the previous paragraph.

Line 92: insert "of" - "....613.37 [of] Gb high....". I would also advise the authors to move any kind of findings of this type to RESULTS.

Lines 94: Delete "we detected"

Line 94: consider change " ...,we tested for population structure between domesticated and wild populations, as well as assessed for signatures of selection associated with domestication."

Line 96-98: Either delete the sentence starting with "We inferred..." or add another 1-2 sentence explaining what exactly you tested.

Lines 104-109: This seems forced and out of place. Either delete it and put it to the discussion OR expand/edit it to be more streamlined.

ANALYSIS:

Line 117: end with "...78 ducks."

2nd Paragraph: "Across samples, a total of 36.1 million (M) SNPs (average per sample = 4.5 M SNPs; range = 2.34 - 9.52 M SNPs) and 3.1M INDELs (average per sample = 0.4M INDELs; range = 0.21 - 0.89M INDELs) were detected (Fig. 1C1B, Supplemental Figs. S1-S2, Supplemental Table S2). ingle base-pair INDELs were the predominant form, and accounting for 38.63% of all detected INDELs (Supplemental Table S3). Our dataset covers 96.2% of the duck dbSNP database deposited in the Genome Variation Map (GVM) (http://bigd.big.ac.cn/gvm/)." In general, domesticated stock showed lower number of SNPs (t test, p = 3.13 × 10−12) and nucleotide diversity (ttest, p = 2.20 × 10−16) as compared to wild mallards (Fig. 1B - C). Moreover, homozygousity in domesticated ducks was significantly higher than ratios in wild mallards (t test, p = 1.35 × 10−10) consistent with the larger panmictic wild population.

Line 137: does 36.1 million SNPs include indels? If not, I would just include the 2 in one summation of total diversity.

Line 142 - 143: The sentence "Single base-pair INDELs were the predominant form, accounting for 38.63% of all detected INDELs (Supplemental Table S3)."

Line 148: Are you sure that your data is "consistent with larger panmictic wild population" ? What about artificial selection and inbreeding within domesticated stock? Maybe both? Consider revising.

Lines 155 - 158: Consider changing the sentence to: "In general, clustering among samples corresponded with their source, that included wild ducks (MDN and MDZ), ducks domesticated for meat production (PK, CV, and ML), and ducks domesticated for egg production (JD, 157 SM, and SX). The dual-purpose domesticate clustered with ducks domesticated for egg production (Fig. 2B-C)."

Lines 184-202: Consider revising to 1 paragraph: "Next, we explored the demographic history of our samples to differentiate whether domestication of meat and egg producing ducks was the result of one or multiple events. First, we estimated changes in effective population size (Ne) in our three genetic clusters in a pairwise sequentially Markovian coalescent (PSMC) framework [22]. The meat type ducks (PK, CV, and ML) showed concordant demographic trajectories with egg and mixture dual-purpose type populations (JD, SM, SX, and GY) with one apparent expansion around the Penultimate Glaciation Period (PGP, 0.30-0.13 Mya) [4, 23] and Last Glacial Period (LGP, 110-12 kya) [24, 25], followed by a subsequent contraction (Fig. 2D). Next, we tested multiple demographic scenarios …."

Line 214: What is the Ne for the wild population. Please make clear by at least referencing Table 1.

Lines 224-229: Please cite sources for some of your statements here. Better to make the statement of your findings and save lines 226-229 for discussion.

Line 241: I would like to know if any other region showed deviation/outliers? Or was there only 1 region across the entire genome? Please clarify.

DISCUSSION: Overall, the discussion is well written, organized, and I find the topics of broad appeal.

I believe the introduction of the Discussion can be combined into a single paragraph and a bit streamlined as it is just reiterating the results.

Lines 348 - 353: Consider splitting into at least 2 sentences.
Line 419: add "and": "dogs [45], and..."

Methods

Are the methods appropriate to the aims of the study, are they well described, and are necessary controls included? Yes

Conclusions

Are the conclusions adequately supported by the data shown? Yes

Reporting Standards

Does the manuscript adhere to the journal's guidelines on minimum standards of reporting? Yes

Statistics

Are you able to assess all statistics in the manuscript, including the appropriateness of statistical tests used? Yes, and I have assessed the statistics in my report.

Quality of Written English

Please indicate the quality of language in the manuscript: Needs some language corrections before being published

Declaration of Competing Interests

Please complete a declaration of competing interests, considering the following questions:

- Have you in the past five years received reimbursements, fees, funding, or salary from an organisation that may in any way gain or lose financially from the publication of this manuscript, either now or in the future?
- Do you hold any stocks or shares in an organisation that may in any way gain or lose financially from the publication of this manuscript, either now or in the future?
- Do you hold or are you currently applying for any patents relating to the content of the manuscript?

- Have you received reimbursements, fees, funding, or salary from an organization that holds or has applied for patents relating to the content of the manuscript?
- Do you have any other financial competing interests?
- Do you have any non-financial competing interests in relation to this paper?

If you can answer no to all of the above, write 'I declare that I have no competing interests' below. If your reply is yes to any, please give details below.

I declare that I have no competing interests

I agree to the open peer review policy of the journal. I understand that my name will be included on my report to the authors and, if the manuscript is accepted for publication, my named report including any attachments I upload will be posted on the website along with the authors' responses. I agree for my report to be made available under an Open Access Creative Commons CC-BY license (http://creativecommons.org/licenses/by/4.0/). I understand that any comments which I do not wish to be included in my named report can be included as confidential comments to the editors, which will not be published.

I agree to the open peer review policy of the journal

To further support our reviewers, we have joined with Publons, where you can gain additional credit to further highlight your hard work (see: https://publons.com/journal/530/gigascience). On publication of this paper, your review will be automatically added to Publons, you can then choose whether or not to claim your Publons credit. I understand this statement.

Yes
