# Supplementary material for: Whole-genome resequencing reveals signatures of selection and timing of duck domestication
Source: Gigascience. 2018 Apr 9;7(4):giy027. doi: 10.1093/gigascience/giy027 (PMC6007426; doi:10.1093/gigascience/giy027)
Supplement: Reviewer_3_Report_(Original_Submission) -- Henner Simianer, Prof. — 20 Dec 2017 Reviewed [file giy027_reviewer_3_report_(original_submission).pdf]

Reviewer Report

Title: Whole-genome resequencing reveals signatures of selection and timing of duck domestication

Version: Original Submission **Date:** 20 Dec 2017

Reviewer name: Henner Simianer, Prof.

Reviewer Comments to Author:

Overall a very nice paper, detailed comments to the authors:
Line 35: 45X coverage is misleading since the individual coverage was much smaller, please make a clearer statement here
L40: Our FST analysis also indicates for the first time ...
L52: of particular economic importance ...
L60-72: This is not introduction, but actually another summary, which I think is obsolete, a slightly more extended real introduction discussing backgraound prior knowledge, and aims of the study, would be preferred
Figure 1B: this panel is nice, but not very informative, what exact information is retrieved from the graph?
L95: The number of deletions was higher than the number of insertions in all nine populations
L105: Move the sentence "Single base-pair INDELs were the predominant form, accounting for 38.63% of all detected INDELs (Supplemental Table S3)." before the sentence "Both the number of SNPs ..."
L111: ... clustered together, the three ...
L117: Show figure for K=2?
L155: ... had the lowest Akaike Information Criteria (AIC) value, ...
L166: ... are lower than in wild mallards ...
Table 1: is it possible to report standard errors or confidence intervals of the reported estimates?
L197: ... white plumage phenotype suggesting a causative mutation. Our result indicates for the first time the duck white plumage associated with selection at ...

L213: of 10kb size.
L224: "... scaffolds longer than 10-kb by 10-kb windows with 5-kb steps." This is not clear to me, please describe better.
L237 was shown
L240 level differs between domesticated and wild duck.
L245 I understand that you limited the GO analysis to certain processes, what happened if you included other processes as well?
L252 identified as being under positive selection
L258 Is "neuronal genes" the right term?
L260 fatty acid
L269 and no gene in breast muscle
L273 The results suggest that the PDC gene is of substantial functional importance in

phenotypic differentiation among wild and domestic ducks.
L289 catalogued 36.1M SNPs and 3.1M INDELs,
L333 ... showed particularly strong signs of selective sweep s presumably associated with domestication.
L340 brain and liver of domesticated ducks compared to ...
L351 differential selection? Do you mean directional selection?
L362 Taken together, our results show that duck domestication was a relatively recent and ...
L440 From the 28,199,227 SNPs not confirmed by dbSNPs, 390 randomly chosen (?) nucleotide sites
L448 Principal Component Analysis (PCA), first by generating the genetic relationship matrix (GRM) from which the first 20 eigenvectors were extracted.

Methods

Are the methods appropriate to the aims of the study, are they well described, and are necessary controls included? Yes

Conclusions

Are the conclusions adequately supported by the data shown? Yes

Reporting Standards

Does the manuscript adhere to the journal's guidelines on minimum standards of reporting? Yes

Statistics

Are you able to assess all statistics in the manuscript, including the appropriateness of statistical tests used? Yes, and I have assessed the statistics in my report.

Quality of Written English

Please indicate the quality of language in the manuscript: Needs some language corrections before being published

Declaration of Competing Interests

Please complete a declaration of competing interests, considering the following questions:

- Have you in the past five years received reimbursements, fees, funding, or salary from an organisation that may in any way gain or lose financially from the publication of this manuscript, either now or in the future?

- Do you hold any stocks or shares in an organisation that may in any way gain or lose financially from the publication of this manuscript, either now or in the future?
- Do you hold or are you currently applying for any patents relating to the content of the manuscript?
- Have you received reimbursements, fees, funding, or salary from an organization that holds or has applied for patents relating to the content of the manuscript?
- Do you have any other financial competing interests?
- Do you have any non-financial competing interests in relation to this paper?

If you can answer no to all of the above, write 'I declare that I have no competing interests' below. If your reply is yes to any, please give details below.

‘I declare that I have no competing interests’

I agree to the open peer review policy of the journal. I understand that my name will be included on my report to the authors and, if the manuscript is accepted for publication, my named report including any attachments I upload will be posted on the website along with the authors' responses. I agree for my report to be made available under an Open Access Creative Commons CC-BY license (http://creativecommons.org/licenses/by/4.0/). I understand that any comments which I do not wish to be included in my named report can be included as confidential comments to the editors, which will not be published.

I agree to the open peer review policy of the journal

To further support our reviewers, we have joined with Publons, where you can gain additional credit to further highlight your hard work (see: https://publons.com/journal/530/gigascience). On publication of this paper, your review will be automatically added to Publons, you can then choose whether or not to claim your Publons credit. I understand this statement.

Yes
